# Free energy relationship analysis for temperature dependence of hydride kinetic isotope effects of NADH/NAD^+^ model reactions: implication for barrier compression by enzyme dynamics

**DOI:** 10.1039/d6sc01847e

**Published:** 2026-05-06

**Authors:** Ava Austin-Kloppe, Nicholas DeGroot, Bikram Dhakal, Jessica Sager, Lauren Phan, Seyedmehrad Poormoghim, Yun Lu

**Affiliations:** a Department of Chemistry, Southern Illinois University Edwardsville Edwardsville Illinois 62026 USA yulu@siue.edu

## Abstract

The observed shift from temperature (*T*)-independence of hydrogen kinetic isotope effects (KIEs) in wild-type enzymes to *T*-dependence of KIEs in enzyme mutants has been explained as evidence for the role of protein dynamics in compressing donor (Don)–acceptor (Acc) distances (DADs) for catalysis. To test this explanation, correlation analysis of free energy changes (Δ*G*° = −44.3 to 6.7 kcal mol^−1^) that simulate system rigidities and *T*-dependence of KIEs (represented by Δ*E*_a_ = *E*_aD_ − *E*_aH_) was carried out for 34 hydride-tunneling reactions of NADH/NAD^+^ models in acetonitrile. For exergonic reactions, Δ*E*_a_ increases as Δ*G*° approaches zero, with the linear trend appearing to reverse for endergonic reactions. Both Δ*E*_a_ and KIEs reach their maximum near thermoneutral reactions, where the charge-transfer (CT) complexation vibration is the weakest and DAD is the longest. A small portion of the free energy change drives the CT complexation vibrations and thus the DAD sampling that correlates with KIEs and their *T*-dependences. The results support the role of protein dynamics in barrier compression for catalysis. The new physical-organic linear Δ*E*_a_–Δ*G*° relationship will contribute to the development of future H-tunneling models as well as updated theories for enzyme catalysis.

## Abstract 2

The Λ-shaped free energy relationship for temperature dependence of hydride KIEs, peaking at Δ*G*° ∼ 0, is identified in solution, supporting the donor–acceptor distance sampling mechanism and the role of fast protein dynamics in enzyme catalysis.

## Graphical abstract

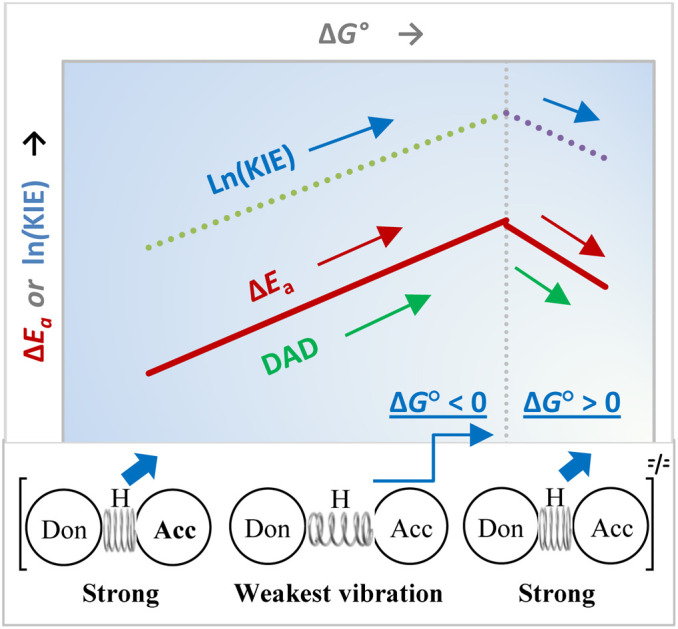

## Introduction

Recent observations of temperature (*T*) independence (or weak dependence) of kinetic isotope effects (KIEs) in various enzyme-catalyzed hydrogen (H) transfer reactions have been ascribed by several research groups to the *T*-independence of narrowly distributed donor–acceptor distances (DADs) for H-tunneling processes.^1–14^ Therefore, such observations are often cited in support of the proposed role of strong protein vibrational dynamics in facilitating short DAD sampling,^1,15–20^ and further promoting enzyme catalysis.^8,12,20–31^ Use of this approach to propose the new physical origin of enzyme catalysis is currently, however, debated.^32–36^ Central to the debate is whether *T*-dependence of KIEs reliably reflects DAD fluctuations or overall system rigidity. To address this question, biochemists have designed experiments to probe enzyme system rigidity and its correlation with the *T*-dependence of KIEs.^6,9,11,12,37–39^ Theoreticians have refined or developed H-tunneling models to simulate the observations in attempts to test whether there is such a correlation.^14,32,33,40–43^ For the same purpose, our group has studied model reactions in solution to explore the potential relationship between the structure and *T*-dependence of hydride KIEs.^24,25,27,29,44–50^ The solution-phase H-transfer reactions permit systematic variation of the molecular structure and solvent environment, enabling more controlled evaluation of how structural rigidity influences the *T*-dependence of KIEs in a broader context.

*T*-Dependence of KIEs reflects the isotopic activation energy difference, represented as Δ*E*_a_ (= *E*_aD_ − *E*_aH_) for hydrogen/deuterium transfer reactions. Semi-classically, Δ*E*_a_ falls within the range between 1.0 and 1.2 kcal mol^−1^, but its relationship with structure is unpredictable. Δ*E*_a_ outside of this range has been used to suggest an H-tunneling mechanism. Notably, a shift from Δ*E*_a_ ∼0 in wild-type enzymes to Δ*E*_a_ > 0 (often exceeding the semi-classical limit) in mutant variants has been frequently observed. This trend has prompted the application of existing H-tunneling models, as well as the development of new theoretical frameworks, to account for these observations and to further elucidate the mechanisms of H-transfer chemistry.^2,5–9,11,12,18,31,42,51–58^ Among these, the recently proposed vibration-assisted activated H-tunneling (VA-AHT) model appears to be able to explain the unusual KIE behaviors.^2,5,8,20,59^

The VA-AHT model incorporates two orthogonal activation processes: (1) heavy atom motions bring the reactants (donor-H and acceptor) and products to degenerate energy states at which H-wave functions overlap ([Don-H ↔ H-Acc]^‡^), *i.e.*, tunnelling ready states (TRS's); and (2) more rapid heavy atom motions, also called promoting vibrations,^19,60^ sample short DADs for efficient tunneling to occur. Since tunneling of a D-isotope requires a smaller average DAD due to its shorter de Broglie wavelength, a higher activation energy is needed leading to *E*_aD_ > *E*_aH_ (assuming that the first activation process is isotope insensitive). In wild-type enzymes, however, strong protein vibrations compress the donor/acceptor closely together, thereby facilitating the formation of short DADs that are extremely densely populated, eliminating the possibility for further short DAD sampling for D-tunneling and making Δ*E*_a_ ∼0. In enzyme mutants, the constructive vibrations are disrupted, and thermal sampling of shorter DADs is allowed so that *T*-dependence of DADs/KIEs (Δ*E*_a_ > 0) emerges. The phenomenological model has been claimed by some researchers to be able to explain all of the hydrogen KIEs.^5,6,8,12^

Computational simulations of *T*-dependence of KIEs followed various theoretical models including the VA-AHT model to investigate the proposed role of fast thermal dynamics in sampling short DADs. Other models include ensemble-averaged variational TS theory with multi-dimensional H-tunneling^32,41,61^ as well as approaches using the empirical valence bond theory.^33,34,62^ While the VA-AHT model has successfully reproduced the Δ*E*_a_ in nonadiabatic reactions to support the thermally activated DAD sampling concept,^63–65^ simulations for more adiabatic hydride/proton transfer reactions have been challenging as there is no direct mathematical relationship yet between DADs and Δ*E*_a_ for this type of reaction.^30,43^ On the other hand, theoretical replications of the observations using other H-tunneling models, especially the huge KIEs, have encountered difficulty.^10,32,36,66^ Nevertheless, even some of the latter computational results sometimes show that Δ*E*_a_ ∼ 0 in some reactions results from the insensitivity of the DAD to temperature,^13,34,41,63^ whereas other researchers have shown that it could also result from the effect of temperature on the microscopic mechanism, for example, on the position of the TS and shape of the potential barrier.^32,34^ The potential relationship between DAD distributions and Δ*E*_a_ magnitudes warrants further investigation to search for a potential mathematical relationship, if it exists, for building future H-tunnelling theoretical frameworks.

To address the “DAD–Δ*E*_a_” relationship, additional data, including from model systems, are needed. Over the years, we have designed hydride transfer reactions of NADH/NAD^+^ models in solution to tackle the problem. Our hypothesis, based on enzyme observations, is that a more rigid system with densely populated DADs gives rise to a smaller Δ*E*_a_.^25^ Systems have been designed to study the electronic, steric, and solvent effects as well as effects of remote heavy group vibrations and mechanisms on the *T*-dependence of KIEs.^25,27,29,44–50^ Current results show that a tighter charge-transfer (CT) complexation between NADH/NAD^+^ model structures exhibits a smaller Δ*E*_a_ value, supporting the hypothesis.

As the project progressed, we seemed to have identified a trend indicating that a reaction with a more negative free energy change (Δ*G*°) tends to show a smaller Δ*E*_a_ value.^29,44,46,48^ This appears consistent with our hypothesis as a stronger hydride donor/acceptor would form stronger CT complexation vibrations and thus more rigid donor–acceptor centers. In this context, a larger negative Δ*G*° corresponds to a tighter CT complexation and, consequently, more densely populated smaller DADs, which in turn result in smaller Δ*E*_a_ values. Based on this reasoning, we envisioned that there might be a free energy relationship with *T*-dependence of KIEs for this type of reaction. As a matter of fact, a VA-AHT-inspired model can be formulated in which a portion of the free energy 
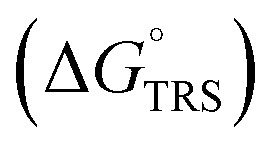
 drives the structural and solvent rearrangement required to reach a TRS, while the remaining portion 
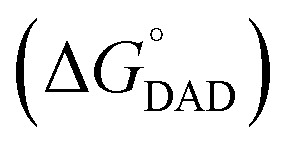
 modulates DAD sampling toward shorter distances; together, both contributions enable H-tunneling. While the overall free energy change 
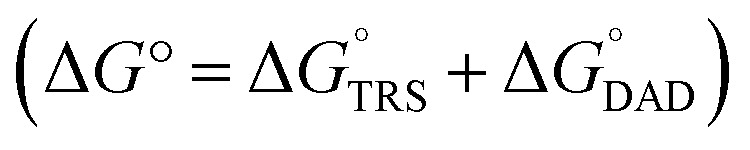
 is expected to linearly correlate with the logarithm of the observed rates (ln(*k*)), Δ*E*_a_ reflects (but is not equal to) the thermal energy required for DAD sampling and is expected to correlate linearly with the 
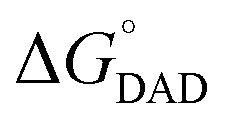
. Although 
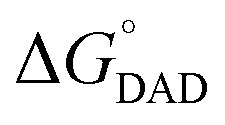
 cannot be directly determined, establishing a Δ*E*_a_–Δ*G*° relationship would provide an indirect means to test our DAD–Δ*E*_a_ hypothesis, clarify the driving force underlying Δ*E*_a_, and deepen our understanding of DAD sampling as well as the enzymatic KIE behavior.

In this work, we examined 34 hydride transfer reactions of NADH/NAD^+^ models in acetonitrile, with Δ*G*° values spanning from −44.3 to 6.7 kcal mol^−1^, to investigate the previously unreported Δ*E*_a_–Δ*G*° relationship and further test our hypothesis concerning the DAD–Δ*E*_a_ relationship. We also compare the Δ*E*_a_–Δ*G*° relationship with the ln(*k*)–Δ*G*° and ln(KIE)–Δ*G*° relationships. Furthermore, this study represents the first application of our hypothesis to endergonic reactions. The dataset reveals how the trends of these relationships evolve when transitioning from exergonic to endergonic regions. These insights enable testing of the DAD sampling mechanism in the VA-AHT(-inspired) model, evaluating other current H-transfer theories, as well as informing the development of future theoretical frameworks for both general H-transfer/tunneling reactions and enzyme catalysis.

## Results

**Fig. 1:**
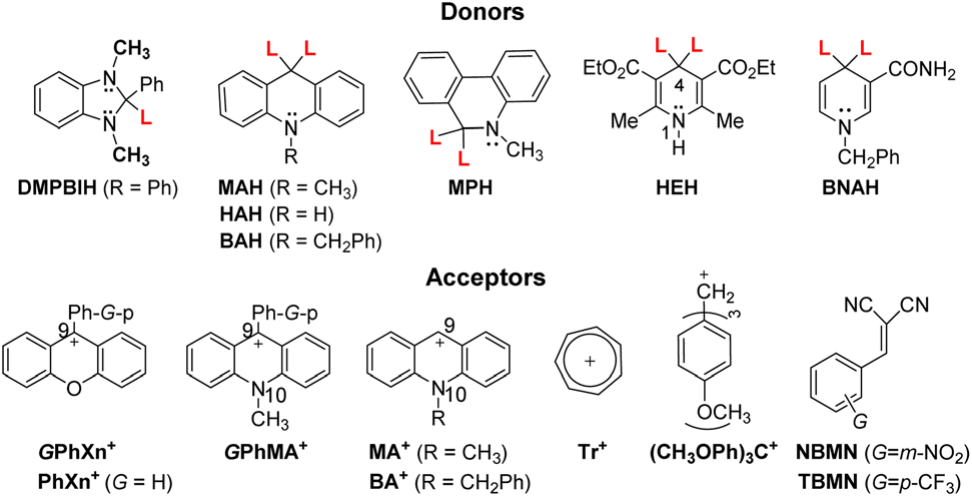
Hydride donors and acceptors (counter-ions: BF_4_^−^) (L = H or D) of the reactions in acetonitrile.

The hydride donor and acceptor structures are shown in Fig. 1. Kinetics were determined spectroscopically on a stopped-flow UV-vis spectrophotometer. The Δ*G*°, second-order rate constants (*k*_2_) and KIEs at 25 °C, as well as the Δ*E*_a_ values are listed in Table 1 (for Rxns 1–34). As noted, some data were taken from our previous publications. Although kinetic study of the endergonic reactions is challenging due to the limited extent of the reaction and slow kinetics, in this work we were able to conduct a study on two such hydride transfer reactions: from Hantzsch 1,4-dihydropyridine (HEH) to benzylidenemalononitriles (BMNs), NBMN (Δ*G*° = 6.3 kcal mol^−1^) and TBMN (6.7 kcal mol^−1^) (Rxns 33 and 34 in Table 1; see structures in Fig. 1). The reactions proceed to completion due to subsequent rapid irreversible transfer of a proton from the N–H site of the HE^+^ product to the dicyanomethanide anion product, as illustrated below,^67^
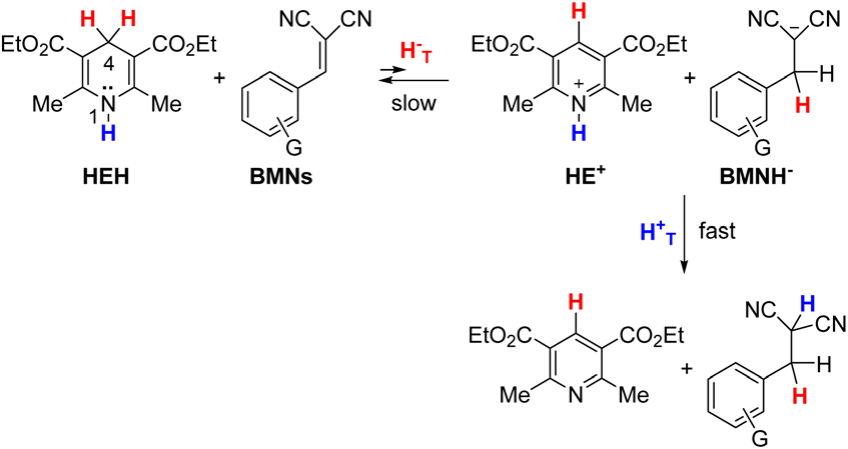


**Table 1:** Hydride affinities 
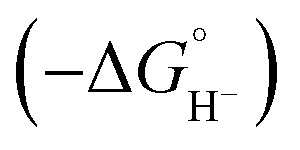
, free energy changes (Δ*G*°), and hydride transfer kinetic data in acetonitrile

Rxns	Donors (Don-H)	Hydride acceptors (Acc^+^BF_4_^−^)	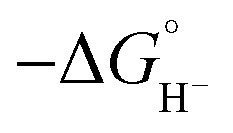 (Don^+^)^l^ (kcal mol^−1^)	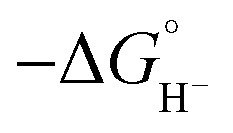 (Acc^+^)^l^ (kcal mol^−1^)	Δ*G*° (kcal mol^−1^)	*k*_2H_ (25 °C)^l^ (M^−1^ s^−1^)	KIE^m^ (25 °C)	Δ*E*_a_^m^ (kcal mol^−1^)
**Exergonic reactions**								
1^a^	DMPBIH	CF_3_PhXn^+^	49.2	93.5	−44.3	1.44 (0.01) × 10^5^	2.56 (0.03)	0.03 (0.07)
2^a^	DMPBIH	BrPhXn^+^	49.2	92.5	−43.3	9.86 (0.09) × 10^4^	2.55 (0.03)	0.07 (0.07)
3^a^	DMPBIH	PhXn^+^	49.2	91.6	−42.4	4.54 (0.05) × 10^4^	2.68 (0.04)	0.27 (0.06)
4^a^	DMPBIH	CH_3_OPhXn^+^	49.2	90.2	−41.0	2.08 (0.02) × 10^4^	2.74 (0.03)	0.55 (0.06)
5^a^	DMPBIH	(CH_3_)_2_NPhXn^+^	49.2	86.7	−37.5	6.34 (0.04) × 10^2^	2.89 (0.06)	0.50 (0.08)
6^b^	DMPBIH	MA^+^	49.2	76.2	−27.0	2.12 (0.01) × 10^2^	3.57 (0.03)	0.43 (0.15)
7^a^	MAH	CF_3_PhXn^+^	76.2	93.5	−17.3	1.03 (0.01) × 10^3^	4.06 (0.04)	0.89 (0.07)
8^a^	MAH	BrPhXn^+^	76.2	92.5	−16.3	6.45 (0.03) × 10^2^	4.04 (0.03)	0.89 (0.07)
9^a^	MAH	PhXn^+^	76.2	91.6	−15.4	4.10 (0.03) × 10^2^	4.08 (0.03)	0.88 (0.05)
10^a^	MAH	CH_3_OPhXn^+^	76.2	90.2	−14.0	1.56 (0.01) × 10^2^	4.18 (0.04)	0.92 (0.16)
11^a^	MAH	(CH_3_)_2_NPhXn^+^	76.2	86.7	−10.5	4.34(0.03)	4.45 (0.05)	0.96 (0.18)
12^c^	MAH	Tr^+^	76.2	83.0	−6.8	4.01 (0.02)	4.95 (0.12)	1.14 (0.10)
13^c^	HAH	Tr^+^	74.9	83.0	−8.1	7.33 (0.05) × 10	5.34 (0.04)	1.27 (0.09)
14^c^	HAH	PhXn^+^	74.9	91.6	−16.7	1.15 (0.01) × 10^3^	4.19 (0.03)	0.88 (0.05)
15^e^	BAH	PhXn^+^	77.4	91.6	−14.2	3.79 (0.02) × 10^2^	4.26 (0.03)	0.89 (0.05)
16^e^	MPH	PhXn^+^	65.7	91.6	−25.9	3.74 (0.02) × 10^3^	3.18 (0.03)	0.71 (0.05)
17^c^	MPH	CH_3_OPhXn^+^	65.7	90.2	−24.5	1.65 (0.01) × 10^3^	3.28 (0.03)	0.63 (0.05)
18^c^^,^^d^	BNAH	MA^+^	59.3	76.2	−16.9	7.16 (0.07) × 10	4.59 (0.06)	1.14 (0.17)
19^c^	BNAH	BA^+^	59.3	77.4	−18.1	2.48 (0.02) × 10^2^	3.75 (0.03)	0.82 (0.07)
20^c^	BNAH	(CH_3_OPh)_3_C^+^	59.3	88.6	−29.3	1.66 (0.01) × 10^5^	2.80 (0.03)	0.75 (0.05)
21^e^	BNAH	(CH_3_)_2_NPhXn^+^	59.3	86.7	−27.4	5.62 (0.04) × 10^4^	3.19 (0.03)	0.82 (0.04)
22^e^	BNAH	(CH_3_)_2_NPhMA^+^	59.3	67.4	−8.1	8.37 (0.08) × 10^−1^	4.79 (0.06)	1.14 (0.21)
23^b^	HEH	MA^+^	64.4	76.2	−11.8	1.56 (0.01) × 10^2^	4.92 (0.04)	0.99 (0.11)
24^f^	HEH	BA^+^	64.4	77.4	−13.0	5.73 (0.03) × 10^2^	4.53 (0.04)	1.01 (0.12)
25^c^	HEH	(CH_3_OPh)_3_C^+^	64.4	88.6	−24.2	5.66 (0.03) × 10^3^	3.64 (0.03)	0.88 (0.06)
26^g^	HEH	CF_3_PhMA^+^	64.4	78.4	−14.0	2.74 (0.03) × 10	5.23 (0.05)	1.13 (0.19)
27^g^	HEH	BrPhMA^+^	64.4	75.8	−11.4	2.09 (0.03) × 10	5.20 (0.08)	1.32 (0.09)
28^g^	HEH	PhMA^+^	64.4	74.1	−9.7	1.37 (0.08) × 10	5.31 (0.04)	1.19 (0.07)
29^g^	HEH	CH_3_PhMA^+^	64.4	72.8	−8.4	1.13 (0.02) × 10	5.30 (0.10)	1.33 (0.10)
30^g^	HEH	CH_3_OPhMA^+^	64.4	71.7	−7.3	1.00 (0.01) × 10	5.11 (0.08)	1.31 (0.10)
31^g^	HEH	(CH_3_)_2_NPhMA^+^	64.4	67.4	−3.0	4.19 (0.03)	5.09 (0.06)	1.27 (0.14)
32^g^	HEH	(CH_3_)_2_NPhXn^+^	64.4	86.7	−22.3	8.87 (0.05) × 10^4^	3.56 (0.02)	0.86 (0.08)

**Endergonic reactions**								
33^c^	HEH	NBMN	64.4	58.1	6.3	1.14 (0.01)	5.13 (0.06)	1.17 (0.15)
34^c^	HEH	TBMN	64.4	57.7	6.7	6.07 (0.02) × 10^−1^	5.27 (0.06)	1.19 (0.16)
35^h^	BnOH	PhXn^+^	—j	91.6	—^j^	6.73 (0.29) × 10^−5^^k^	4.83 (0.21)^k^	1.00 (0.26)
36^i^	i-PrOH	PhXn^+^	—j	91.6	—^j^	2.02 (0.05) × 10^−5^^k^	3.63 (0.23)^k^	0.83 (0.27)
37^i^	i-PrOH-β,β-d_6_	PhXn^+^	—j	91.6	—^j^	2.01 (0.05) × 10^−5^^k^	3.64 (0.16)^k^	0.80 (0.19)
38^i^	*cyclo*-HexOH	PhXn^+^	—j	91.6	—^j^	2.68 (0.01) × 10^−5^^k^	3.68 (0.16)^k^	0.90 (0.27)

a Ref. 44.

b Ref. 25.

c This work.

d Δ*E*_a_ = 1.53 (0.15) was reported by us before.^25^ This was determined with temperatures from 4.5–29.5 °C, using the Hi-Tech Scientific SFA-20 fast kinetic determination kit interfaced to a UV-vis spectrophotometer. The conclusions from that publication are not changed using the numbers obtained in this work.

e Ref. 48.

f Ref. 29.

g Ref. 47.

h Ref. 24.

i Ref. 29.

j Unreported.

k For 22 °C.

l Ref. 68.

m Numbers in parentheses are pooled standard deviations (standard deviations of the average values from different days of measurements in this work are listed in Tables S1 to S10 for comparison).

Table 1 also summarizes the relevant kinetic results for our previous study of the unfavorable hydride transfer reactions from alcohols (R^1^CH(OH)R^2^) to PhXn^+^ in acetonitrile (Rxns 35–38).^24,29^
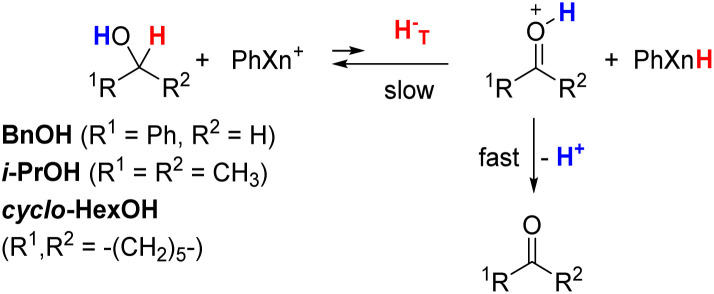


In these reactions, hydride-transfer from the α-position of the alcohol generates an α-hydroxy carbocation intermediate (R^1^C^+^(OH)R^2^). This process is subsequently followed by rapid proton transfer of the OH group to basic species in solution (*e.g.*, excess alcohol substrate or acetonitrile solvent), producing the corresponding oxidized carbonyl product (R^1^C(O)R^2^).^24,29,69–71^

While these extremely slow hydride transfer processes are expected to be endergonic, the corresponding free energy data are not available in the literature, limiting quantitative free energy relationship analysis. Notably, these reactions proceed significantly more slowly than the endergonic reactions of HEH with BMNs (more than 10^4^ times slower, see Table 1), suggesting that they would be even more endergonic. Estimated Δ*G*° values have therefore been obtained by extrapolating the linear ln(*k*_2_)–Δ*G*° correlation derived from reactions 1–34 (see the Discussion section below). These estimates are included to support the discussion of the trends observed across this limited set of endergonic reactions.

## Discussion

Although the KIEs at 25 °C are all below 6, a majority of the Δ*E*_a_ values fall outside of the semiclassical range from 1.0 to 1.2 kcal mol^−1^ (Table 1). These results are consistent with observations from enzyme-catalyzed hydride transfer reactions involving NADH/NAD^+^ coenzymes. In both enzymatic and solution systems, such findings have been analyzed to suggest tunneling mechanisms.^5,48,72–74^

### Free energy relationship analysis for *T*-dependence of KIEs: the Λ-shaped Δ*E*_a_–Δ*G*° correlation

**Fig. 2:**
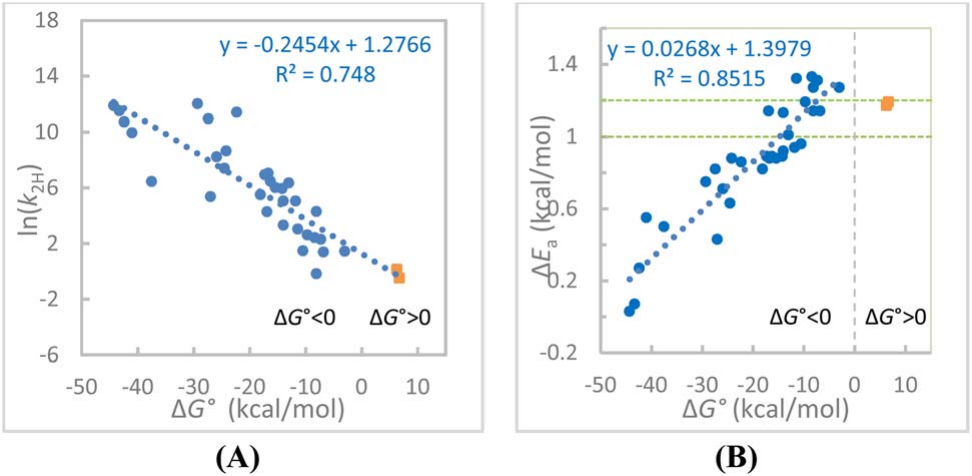
(A) The linear fit of the ln(*k*_2H_)–Δ*G*° data for the 34 hydride transfer reactions (Rxns 1–34). (B) The linear fit of the Δ*E*_a_–Δ*G*° data for the 32 exergonic hydride transfer reactions in acetonitrile (Rxns 1–32). The trend appears to turn to the opposite direction for endergonic reactions of BMNs (Rxns 33–34). Area between the two horizontal green dotted lines represents the semiclassical range. The reactions 35–38 are not included in both plots (see the text).

We first performed a linear correlation analysis of all 34 ln(*k*_2_)–Δ*G*° data points (Fig. 2A). Although the fit is poor (*R*^2^ = 0.748), it reveals a clear trend that the reaction rate decreases as Δ*G*° becomes less negative within the exergonic region and continues to decrease as the reactions become endergonic. Analysis of the Δ*E*_a_–Δ*G*° relationship shows a relatively good linear correlation for the 32 exergonic reactions (Fig. 2B for Rxns 1–32, Δ*G*° = −44.3 to −3.0 kcal mol^−1^, *R*^2^ = 0.852), whereas the two endergonic reactions appear as significant outliers. Within the exergonic region, the Δ*E*_a_ increases as Δ*G*° approaches zero. This new observation is consistent with our expectation; a more negative Δ*G*° corresponds to a more rigid system and a smaller Δ*E*_a_, with a portion of Δ*G*° 
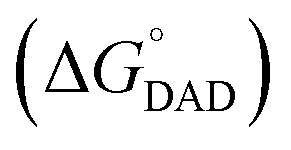
 contributing to the activation process associated with DAD sampling.

**Fig. 3:**
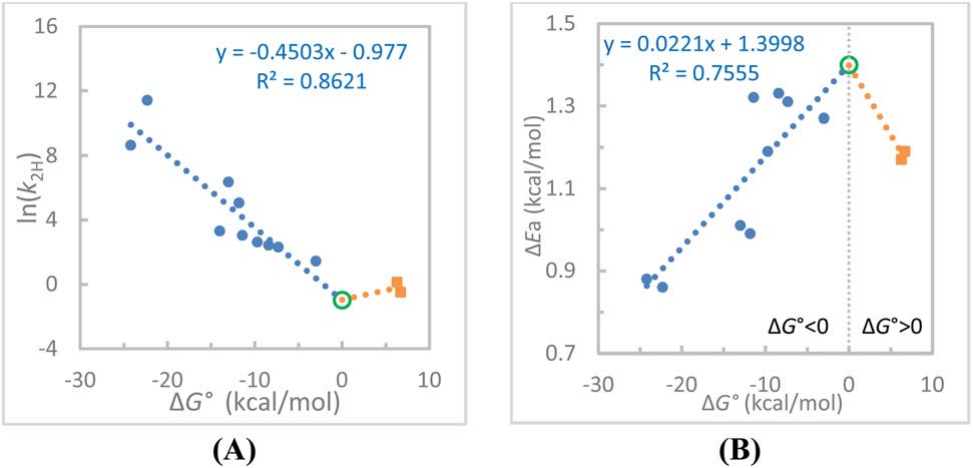
(A) The linear fit of the ln(*k*_2H_)–Δ*G*° data for the 12 hydride transfer reactions with a common HEH hydride donor (Rxns 23–34). (B) The linear fit of the Δ*E*_a_–Δ*G*° data for the same 12 exergonic hydride transfer reactions. In both plots, the trend appears to turn to the opposite direction for endergonic reactions 33–34. The empty green circle point in each plot is an extrapolated point at Δ*G*° = 0 derived from the linear equation from the exergonic reactions.

Therefore, the two endergonic reactions appear to align with the 32 exergonic reactions in the linear ln(*k*_2_)–Δ*G*° correlation, whereas they appear as significant outliers relative to the linear Δ*E*_a_–Δ*G*° correlation for the 32 exergonic reactions. In the latter case, Δ*E*_a_ reaches a maximum when the negative Δ*G*° approaches zero, but upon entering the endergonic region, it begins to decrease. One may think that this behavior resulted from the experimental error or from structural diversity among the reactants, where factors other than electronic effects, such as steric effects, can affect the CT complexation strength and, consequently, the DAD distributions. While we already restricted the structures to rings containing C, N, and O that involve only 2p orbitals in CT complexation and the reactions to only one-step hydride transfers between two carbons, to further minimize the structural variability factor, we replotted the ln(*k*_2_)–Δ*G*° and Δ*E*_a_–Δ*G*° correlations using only the 12 reactions with a common hydride donor, HEH (Fig. 3A and B, respectively). In both plots, the endergonic data points are clearly outliers.

As shown in Fig. 3A, the two endergonic reactions appear to turn the linear ln(*k*_2_)–Δ*G*° correlation derived from the exergonic reactions to the opposite direction. As shown in Fig. 3B, the two endergonic reactions clearly turn the linear Δ*E*_a_–Δ*G*° correlation for the exergonic reactions to the opposite direction. Using the extrapolated Δ*G*° = 0 point from the exergonic reaction data (the circled points in Fig. 3A and B), the corresponding linear correlations with the two data points from the endergonic reactions were drawn, which show a “V-shaped” ln(*k*_2_)–Δ*G*° relationship and a “Λ-shaped” Δ*E*_a_–Δ*G*° relationship.

We first discuss about the Δ*E*_a_–Δ*G*° relationship (Fig. 2B and 3B). Although the current analysis includes only two endergonic reaction data points, this trend could be reinforced by considering additional potential endergonic reactions 35–38. While the Δ*G*° values are not available for these reactions, a qualitative assessment can still be performed. These Δ*G*° values could be approximately derived by substituting the corresponding *k*_2_ values into the linear ln(*k*_2_)–Δ*G*° correlation equation for the reactions 1–34 (Fig. 2A). For one example, the *k*_2_ for the reaction of BnOH with PhXn^+^ in acetonitrile at 22 °C is 6.73 × 10^−5^ M^−1^ s^−1^ (Table 1). Using our reported rate data at other temperatures^24^ for an Arrhenius analysis, the rate constant at 25 °C is estimated to be 8.59 × 10^−5^ M^−1^ s^−1^. Substituting the number in the linear fit equation from Fig. 2A yields an estimated Δ*G*° = 43.4 kcal mol^−1^, indicating a highly endergonic process. We recognize that such an estimate could carry large uncertainty as the fit equation used depends upon many factors, such as dataset size, structural similarity among reactions, as well as experiment errors. Therefore, these estimated Δ*G*° values will not be used for quantitative analysis of the Δ*E*_a_–Δ*G*° relationship. Nevertheless, the Δ*E*_a_ values for reactions 35–38 (0.80–1.00 kcal mol^−1^) are significantly lower than those of the two endergonic reactions (33–34; ∼1.2 kcal mol^−1^). These observations support the conclusion that the Δ*E*_a_–Δ*G*° trend for endergonic reactions is opposite to that observed for exergonic reactions.

The key question is why the clear linear Δ*E*_a_–Δ*G*° plot for the exergonic reactions reverses direction at Δ*G*° ∼ 0. This observation is reminiscent of the Marcus inverted region. In fact, the weakest CT complexation (*i.e.*, the longest average DAD) at the TRS is expected from thermoneutral reactions 
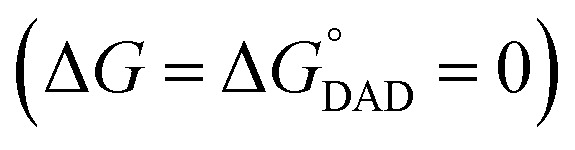
. This inference is based on Hammond's postulate. For an exergonic reaction, a more negative Δ*G*° would correspond with a tighter TRS that resembles more reactive reactant structures, whereas for an endergonic reaction, the more positive Δ*G*° would also correspond with a tighter TRS that, however, resembles more reactive products. Therefore, within the VA-AHT-inspired model, 
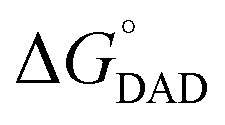
 can be a proxy for TRS rigidity and a Λ-shaped Δ*E*_a_–Δ*G*° relationship is expected, with the maximum Δ*E*_a_ occurring near Δ*G*° = 0 where the DAD is longest. Our experimental observations (Fig. 2B and considering the Δ*E*_a_ values of the endergonic reactions 35–38) are largely consistent with this expectation. Therefore, not only the Δ*E*_a_–Δ*G*° correlation derived from exergonic reactions but the reversed Δ*E*_a_–Δ*G*° relationship for endergonic reactions also supports our proposed DAD–Δ*E*_a_ relationship. That is, a more flexible system with a longer average DAD and a Δ*G*° closer to zero exhibits a larger Δ*E*_a_ value, regardless of whether the reaction is exergonic or endergonic.

It should be noted that steric effects can influence the DAD distributions and, consequently, the Δ*E*_a_ values. Increased steric crowding may enhance system rigidity, leading to a decrease in Δ*E*_a_.^25^ Conversely, steric hindrance may also physically separate the donor and acceptor, enhancing system flexibility and resulting in higher Δ*E*_a_ values. Therefore, variations in steric effects across different systems may contribute to the observed scatter in the correlation, in addition to experimental errors.

### The ln(*k*_2_)–Δ*G*° correlation is consistent with the Λ-shaped Δ*E*_a_–Δ*G*° relationship

**Fig. 4:**
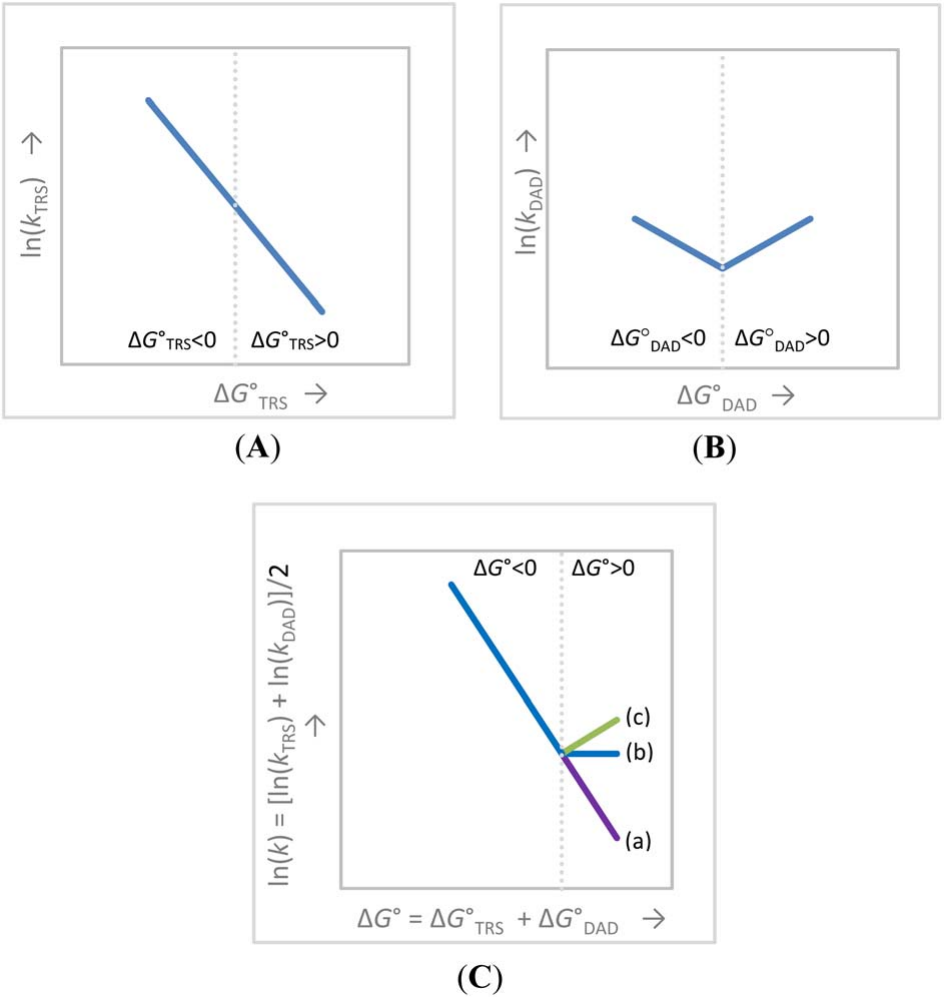
Predictions from the VA-AHT-inspired model for H-tunneling reactions. (A) The linear 
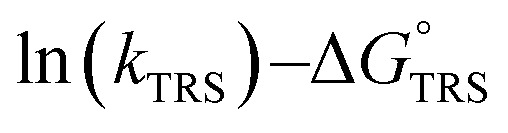
 correlation involving both endergonic and exergonic reactions. (B) The V-shaped 
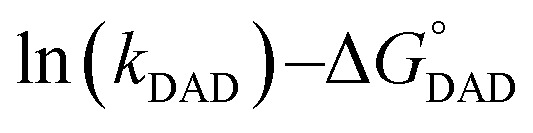
 correlation with the turning point at 
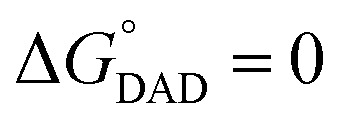
. (C) The overall ln(*k*)–Δ*G*° correlation that reflects the combined assumed equal contributions of the plots (A) and (B); case (a): (A) is a steeper line or (B) is a shallow V-shape or both; from cases (b) to (c): (A) becomes flatter or (B) becomes deeper or both. Note that the Λ-shaped 
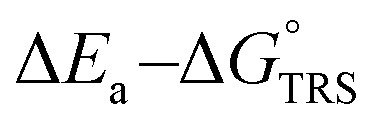
 relationship is another prediction from the model, mirroring the V-shaped 
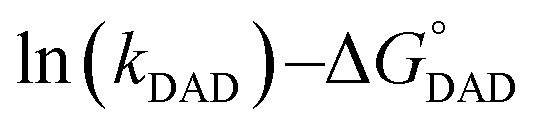
 correlation (but *k*_DAD_ is not directly experimentally accessible).

Another question arises as to why the Δ*E*_a_ values of the two endergonic reactions decrease (Fig. 3A) but their rates increase (Fig. 3B) as compared to their corresponding values at Δ*G*° = 0. The question can also be answered by considering the two orthogonal but coupled activation processes in the VA-AHT-inspired model, the TRS formation and DAD sampling. Note that TRS formation involves both structural (hybridization and charge) and solvation reorganizations. The observed *k*_2_ is determined by both processes (driven by both 
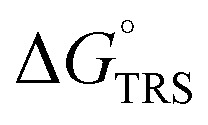
 and 
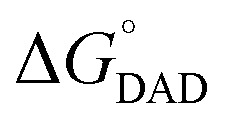
) and can be expressed as *k*_2_ = (*k*_2,TRS_ × *k*_2,DAD_)^1/2^ (or more generally, as a weighted geometric mean), where *k*_2,TRS_ is the rate of reaching the TRS with correct donor–acceptor alignment and *k*_2,DAD_ is the rate of reaching the TRS with short DADs; both structural features of the TRS are required for tunneling. Theoretically, ln(*k*_TRS_) would correlate linearly with the 
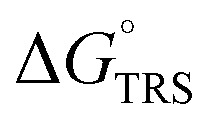
 across both the exergonic and endergonic regions, which is, ln(*k*_TRS_) decreases as 
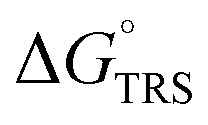
 increases from negative through zero to positive values (Fig. 4A). In contrast, ln(*k*_DAD_) is expected to decrease as exergonic reactions approach thermoneutral reactions due to increasing DADs, but then increase as the reactions become more endergonic due to, however, decreasing DADs (by Hammond's postulate, as discussed above). In other words, a V-shaped 
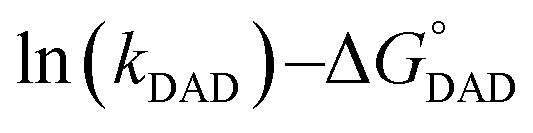
 relationship is expected (Fig. 4B). Since the observed ln(*k*_2_)–Δ*G*° correlation is the “sum” of the two relationships (A and B), the observed ln(*k*_2_)–Δ*G*° correlation is expected to show a broken-line profile with a breaking point at Δ*G*° = 0 (Fig. 4C).

Theoretically, the pattern of the ln(*k*_2_)–Δ*G*° correlation across exergonic and endergonic reactions depends upon the nature of the reaction systems, specifically, the steepness of the linear 
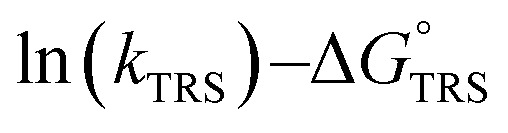
 correlation and the depth of the V-shaped 
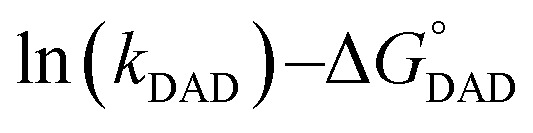
 correlation. Fig. 4C(a)–(c) describe three representative patterns. In (a), the 
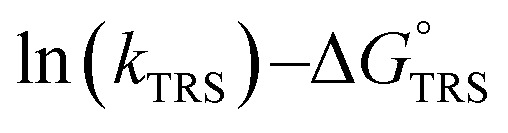
 linear correlation is steep while the V-shaped 
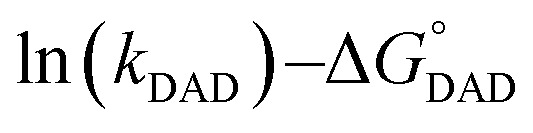
 correlation is shallow, leading to only a slight deviation at Δ*G*° = 0. From (b) to (c), the former correlation becomes less steep and/or the latter becomes deeper, leading to a more evident break and, ultimately a distinctly V-shaped profile. Therefore, all three patterns (a) to (c) are possible. Fig. 3A presents the V-shaped ln(*k*_2_)–Δ*G*° correlation (type (c) pattern). This correlation, along with the Λ-shaped Δ*E*_a_–Δ*G*° correlation in Fig. 3B, appears to support the two-coordinate mechanism proposed in the VA-AHT-inspired model.

It should be noted that patterns (b) and (c) in Fig. 4C also resemble the Marcus inverted region. It is important, however, to note that relying solely on the ln(*k*)–Δ*G*° correlation to identify the DAD sampling mechanism needs caution as the bent or V-shaped 
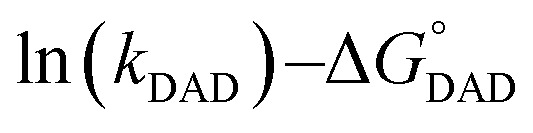
 relationship may be masked by the usually dominant and thus much steeper 
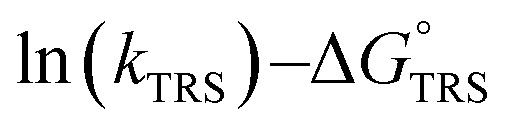
 relationship. The latter relationship reflects structural rehybridization, charge redistribution, and solvent reorganization (pattern (a)), and thus contribute significantly to the activation process. Furthermore, the “bentness” of the ln(*k*)–Δ*G*° correlation depends on the relative linear correlations in between exergonic and endergonic regions. Each slope is influenced by factors such as electronic and steric properties, structural dynamics, the statistical size of the dataset, as well as experimental uncertainties. Consequently, failure to observe a bent or V-shaped ln(*k*)–Δ*G*° correlation cannot preclude the existence of a DAD sampling mechanism. Therefore, the seemingly linear ln(*k*)–Δ*G*° correlation observed for all of the 34 reactions in Fig. 2A cannot be taken as evidence against the existence of the DAD sampling mechanism. Nevertheless, simultaneous observation of both the Λ-shaped Δ*E*_a_–Δ*G*° relationship and V-shaped ln(*k*)–Δ*G*° relationship for the 12 reactions of HEH (Fig. 3B*versus*3A) provides strong support for our DAD–Δ*E*_a_ relationship hypothesis.

Therefore, the Λ-shaped Δ*E*_a_–Δ*G*° correlation and various patterns of the ln(*k*_2_)–Δ*G*° correlation are predicted by the VA-AHT-inspired model, and our experiments are consistent with these predictions. Note that observation of a Λ-shaped Δ*E*_a_–Δ*G*° correlation does not guarantee that an evidently bent or V-shaped ln(*k*)–Δ*G*° correlation will also be observed. Likewise, a single bent or V-shaped ln(*k*_2_)–Δ*G*° correlation should also be used cautiously to determine the DAD sampling mechanism.

### The Λ-shaped ln(KIE)–Δ*G*° correlation is also consistent with the Λ-shaped Δ*E*_a_–Δ*G*° relationship

With the KIE data in hand, we are also able to study the KIE–Δ*G*° correlation. Study of this relationship has had a long history. In 1960, Melander and Westheimer proposed that the maximum KIE should occur at Δ*G*° = 0 where the transition state (TS) is symmetric.^75,76^ Later, in 1980, Kresge deduced a parabolic relationship linking KIEs to (Δ*G*°)^2^ from the Marcus rate theory.^77^ In the 1970s, Bell provided an alternative by introducing a tunnel correction to the one-dimensional energy barrier in the TS theory. In his model, tunneling probability is the greatest at a symmetrical TS, leading to the highest KIE, and it decreases as the TS becomes reactant- or product-like.^78^ Since the 1980s, Kreevoy and coworkers studied the structure–KIE relationship for the hydride transfer reactions of NADH models in isopropanol/water using the Marcus rate theory that attributes a fraction of the KIE to the corner-cutting tunneling mechanism.^72,74,79–81^ Kil and Lee later found that KIEs of several such exergonic hydride transfer reactions increase as Δ*G*° becomes less negative close to zero.^82^ They used Kreevoy's treatment to explain their results and further predicted that the KIE–Δ*G*° relationship would reverse upon entering the endergonic region, which has, however, not yet been experimentally supported due to lack of experiment data. Thus, this latter model also predicts a similar bell-shaped (or “upward/downward”) dependence of KIEs on Δ*G*°, with maximal tunneling and thus the largest KIE occurring near Δ*G*° ∼0, where the effective barrier is highest.

Within the VA-AHT(-inspired) model, the KIE is defined differently. It is primarily dependent upon the difference in isotopic wave-function overlaps at the TRSs over a spectrum of DADs.^6,8,40,59^ Because the vibrational wave-packet of H is more diffuse than that of the D isotope, the H-overlap is more than the D-overlap so that KIE > 1 ^40,83^ (in other words, because D-tunneling requires shorter DADs than H-tunneling,^40,83–86^*k*_D,DAD_ < *k*_H,DAD_). Moreover, since the overlap for D-transfer decreases more rapidly than that for H-transfer with increasing DAD, the KIE increases with DAD (in other words, the difference between *k*_D,DAD_ and *k*_H,DAD_ becomes larger as the DAD increases). Consequently, the KIE is predicted to be the largest at Δ*G*° = 0 where the DAD is the longest, and to decrease as the TRS becomes reactant- or product-like where the DAD shortens. Since the 
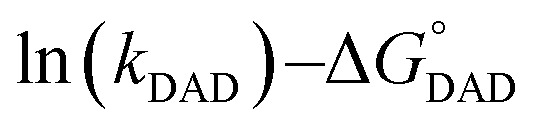
 relationship is V-shaped (Fig. 4B), and *k*_TRS_ is mostly isotope insensitive, the 
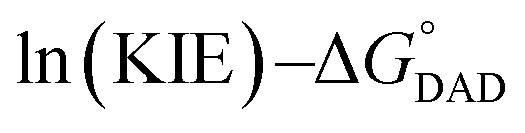
 relationship is expected to be Λ-shaped.

**Fig. 5:**
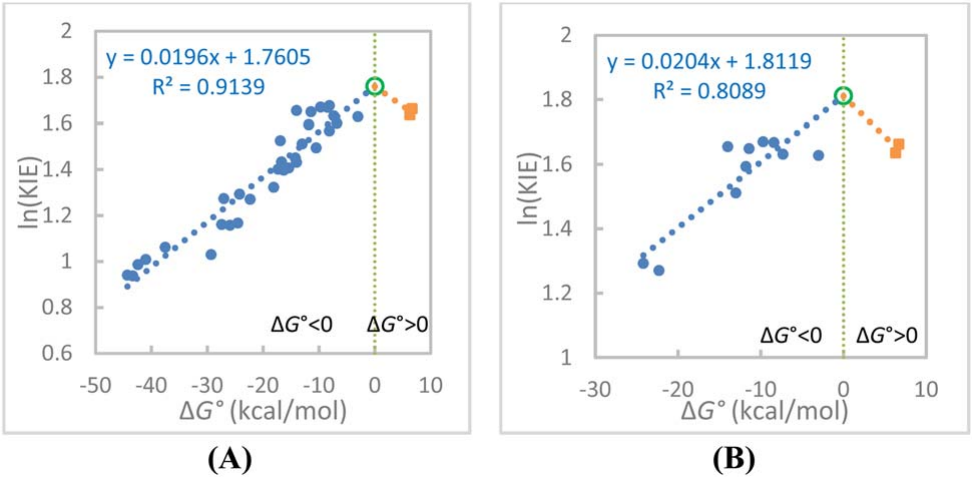
(A) The linear fit of the ln(KIE(25 °C))–Δ*G*° data for the 34 hydride transfer reactions. The reactions 35–38 are not included (see the text). (B) The linear fit of the ln(KIE(25 °C))–Δ*G*° data for the 12 hydride transfer reactions of a common hydride donor HEH (Rxns 23–34). The empty green circle point in each plot is an extrapolated point at Δ*G*° = 0 derived from the linear equation from the exergonic reactions.

We examine the ln(KIE)–Δ*G*° correlation to indirectly test the predicted Λ-shaped 
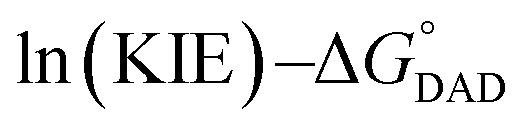
 relationship. The 34 ln(KIE)–Δ*G*° data points and the 12 such data points for the reactions of HEH only, are plotted in Fig. 5A and B, respectively. Linear fits were applied separately to the exergonic and endergonic regions, with the Δ*G*° = 0 point extrapolated from the exergonic data. In both plots, the endergonic reactions appear to reverse the trend observed for the exergonic reactions. The reverse trend could be further supported by including the smaller KIE values (3.63–4.83) from the endergonic reactions between alcohols and PhXn^+^ (Rxns 35–38), but again because of lack of the corresponding Δ*G*° values these KIE data cannot be used for quantitative fitting analysis.

The Λ-shaped ln(KIE)–Δ*G*° relationship is consistent with the Λ-shaped Δ*E*_a_–Δ*G*° relationship in that both Δ*E*_a_s and KIEs reach maxima near thermoneutral conditions (Fig. 5*versus*3B). Importantly, both relationships can be uniformly explained using the DAD coordinate mechanism within the VA-AHT-inspired model. According to this model, the KIE increases with the DAD. Note that previous studies on enzymatic reactions as well as NADH/NAD^+^ model reactions have showcased this KIE–DAD relationship for exergonic reactions (ref. 48 and references cited therein). Therefore, the maxima in both the KIE and Δ*E*_a_ occurring near Δ*G*° = 0 correspond to the longest DADs in H-tunneling mechanisms.

We noticed that the slopes of the Δ*E*_a_–Δ*G*° (0.0268) and ln(KIE)–Δ*G*° (0.0196) correlations for the exergonic reactions are significantly small (Fig. 2B and 5A). This likely partly reflects the fact that Δ*E*_a_ and ln(KIE) correlate only with the 
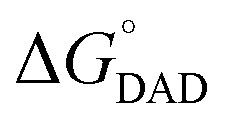
, which constitutes only a small fraction of the overall Δ*G*° in this particular class of reactions, so that they are much less sensitive to the overall Δ*G*°.

## Conclusions

Free-energy dependences of Δ*E*_a_, rates (ln(*k*)), and ln(KIE) were analysed with 34 hydride tunneling reactions of NADH/NAD^+^ models between two carbons in acetonitrile, which span both exergonic and endergonic regions. A clear linear Δ*E*_a_–Δ*G*° relationship was, for the first time, observed for the exergonic reactions, with Δ*E*_a_ increasing as Δ*G*° approaches zero. A quantitative relationship has not yet been obtained for the limited number of endergonic reactions, but reversal of the trend from the exergonic reactions including reactions 35–38 has been clearly shown. This is consistent with our hypothesis regarding the DAD–Δ*E*_a_ relationship. Δ*G*° (strictly, 
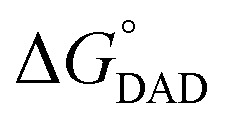
) acts as an indicator of system rigidity (or DAD distributions), where thermoneutral reactions correspond to the most flexible and longest DADs. Overall, a portion of Δ*G*° of the reactions modulates the CT complexation vibrations and thus DAD sampling, which is directly related to the *T*-dependence of KIEs. This resulting Λ-shaped Δ*E*_a_–Δ*G*° relationship agrees with the prediction from the VA-AHT-inspired model that involves both the TRS formation and DAD sampling processes and attributes the KIE to only the latter DAD sampling process.

The second prediction from the same model includes the bent- or V-shaped ln(*k*)–Δ*G*° relationship with the breaking/turning point at Δ*G*° = 0. It was found that the ln(*k*)–Δ*G*° plot is linear across the 34 reactions, but when using the data from the 12 reactions with a common hydride donor HEH, the V-shaped ln(*k*)–Δ*G*° relationship emerges, which mirrors the Λ-shaped Δ*E*_a_–Δ*G*° relationship found from the same series of the reactions. The latter also appears to agree with the DAD sampling mechanism within the VA-AHT-inspired model.

The third prediction from the model includes the Λ-shaped ln(KIE)–Δ*G*° relationship. It was found that the ln(KIE)–Δ*G*° plot is linear across the 32 exergonic reactions, but when using the endergonic reactions, the correlation is reversed. This Λ-shaped trend is the same when using the 12 reactions of HEH. These results are consistent with the prediction. Therefore, all of the three predictions from the VA-AHT-inspired model, including the V-shaped ln(*k*)–Δ*G*°, Λ-shaped Δ*E*_a_–Δ*G*°, and Λ-shaped ln(KIE)–Δ*G*° relationships, were simultaneously found in the 12 reactions of HEH, which support our DAD–Δ*E*_a_ relationship hypothesis.

Caution is required when using the ln(*k*)–Δ*G*° correlation alone to identify the DAD sampling mechanism as the expected bent- or V-shaped correlations can be largely masked by the dominant contribution from TRS formation, as well as by system selection and experimental uncertainties. Conversely, observation of the bent- or V-shaped ln(*k*)–Δ*G*° correlations alone should also be cautiously used to propose the DAD sampling mechanism, since 
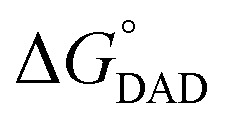
 could account for a small portion of the Δ*G*° so that system selection and experimental uncertainty need to be carefully considered. Also, a Λ-shaped ln(KIE)–Δ*G*° relationship alone should not be considered definitive evidence for DAD sampling either, since similar trends can arise from the Marcus theory combined with the corner-cutting tunneling mechanism process, or even possibly from the classical Melander–Westheimer's “bond-stretching” model. In sharp contrast, the newly identified Λ-shaped Δ*E*_a_–Δ*G*° relationship appearing to arise only from the DAD sampling could provide evidence for the DAD sampling mechanism; however, whether or not this particular relationship is explainable by other tunnelling models remains unclear.

The small slopes of the Λ-shaped Δ*E*_a_–Δ*G*° and ln(KIE)–Δ*G*° relationships may suggest that only a small portion of the Δ*G*° contributes to the activated DAD sampling process. The DAD increases as Δ*G*° of the exergonic reactions becomes less negative, becomes the largest with thermoneutral reactions, and decreases as Δ*G*° becomes more positive in the endergonic reactions. This Λ-shaped Δ*E*_a_–Δ*G*° relationship should be useful for evaluating the existing H-tunneling models and helping develop future H-transfer/tunneling theories.

Overall, our Δ*E*_a_–Δ*G*° correlation results provide support for the DAD–Δ*E*_a_ relationship in both the VA-AHT and VA-AHT-inspired models; systems with more densely distributed small DADs give rise to smaller *T*-dependence of KIEs. Side-by-side comparison of solution-phase and enzymatic reactions on KIEs and their *T*-dependences suggests that frequently observed *T*-independence of KIEs from enzymes can be attributed to strong constructive (fast) protein dynamics (modulated by 
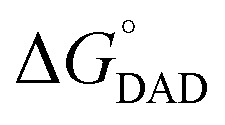
) that compress the donor and acceptor close to each other for H-tunneling to occur. Therefore, there appears a synergy between C–H activation (for TRS formation and DAD sampling) and diverse protein dynamics (encoded in 
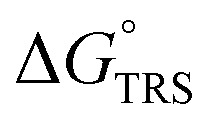
 and
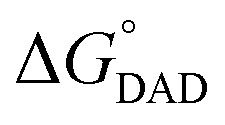
, respectively) cooperatively functioning in enzymatic reactions.

## Author contributions

A. A.-K., N. D., B. D. and J. S. performed organic synthesis and kinetic measurements. A. A.-K. and N. D. wrote part of the paper. B. D. analyzed the data. L. P. and S. P. performed organic synthesis. Y. L. designed research and wrote the manuscript.

## Conflicts of interest

The authors declare no competing financial interests.

## Supplementary Material

SC-OLF-D6SC01847E-s001

## Data Availability

The data underlying this study are available in the published article and its supplementary information (SI). Supplementary information: synthesis, procedures to determine the rate constants, *T*-dependence of KIE plots, tabulated raw rate constant data, and the primary kinetic experiment data. See DOI: https://doi.org/10.1039/d6sc01847e.
