# Supplementary material for: Free energy relationship analysis for temperature dependence of hydride kinetic isotope effects of NADH/NAD+ model reactions: implication for barrier compression by enzyme dynamics
Source: Chem Sci. 2026 May 6;17(25):12440–50. doi: 10.1039/d6sc01847e (PMC13181604; doi:10.1039/d6sc01847e)
Supplement: SC-017-D6SC01847E-s001 [file SC-017-D6SC01847E-s001.pdf]

Supplementary Information (SI) for Chemical Science.
This journal is © The Royal Society of Chemistry 2026

Supplementary Information

Free Energy Relationship Analysis for Temperature Dependence of Hydride Kinetic Isotope Effects of NADH/NAD$^+$ Model Reactions: Implication for Barrier Compression by Enzyme Dynamics

Ava Austin-Kloppe,[†] Nicholas DeGroot,[†] Bikram Dhakal, Jessica Sager, Lauren Phan, Seyedmehrad Poormoghim, Yun Lu*
Department of Chemistry, Southern Illinois University Edwardsville, Edwardsville, Illinois 62026, United States

General Procedures…… S2
Kinetic Procedures…… S2
Plots of temperature dependence of KIEs…… S3
Raw rate constants (Tables S1-S10)…… S5
References …… S8
Data availability statement…… S9

General Procedures

Syntheses of hydride donors of MAH, HAH, MPH, BNAH, HEH and their dideuterio analogues, as well as hydride acceptors of $PhXn^+BF_4^-$, $MA^+BF_4^-$, $BA^+BF_4^-$ and $CH_3OPhXn^+BF_4^-$, have been reported by us.[1-3] The deuterium content in all of the hydride donor compounds but BNAH is generally > 98% by NMR). The deuterium content in BNAH-4,4-d,d is 96% (by NMR). The $Tr^+BF_4^-$ was purchased and purified by recrystallization from acetonitrile-dry ether. $(CH_3OPh)_3C^+BF_4^-$ was synthesized by dehydration of its alcohol precursor (tri(*p*-methoxyphenyl)methanol), which was a gift from Vernon D. Parker's lab from Utah State University. The product was recrystallized from acetonitrile-dry ether. ^1H NMR (CD_3CN, 400 MHz): δ (ppm) 7.57-7.59 (d, 6H), 7.29-7.32 (s, 6H), 4.07 (s, 9H). The *m*-$NO_2PhCH=C(CN)_2$ (NBMN) and *p*-$CF_3PhCH=C(CN)_2$ (TBMN) were prepared from the reaction of malononitrile with corresponding substituted benzaldehyde in ethanol in the presence KOH using a published procedure.[4,5] Melting point of NBMN: 104.0-106.0 °C, ^1H NMR δ (ppm, CD_3CN): 8.72-8.73 (1H, t), 8.41-8.44 (1H, dd), 8.22-8.25 (1H, dd), 8.21 (1H, s), 7.79-7.83 (1H, t). Melting point of TBMN: 109.0-111.0 °C, ^1H NMR δ (ppm, CD_3CN): 8.17 (1H, s), 8.03-8.05 (2H, d), 7.84-7.86 (2H, d).

HPLC grade acetonitrile was redistilled twice under nitrogen, with the presence of $KMnO_4/K_2CO_3$ (to remove the reducing impurity) and P_2O_5 (to remove water) in order, for kinetic measurements.

Kinetic procedures

Kinetic measurements were carried out by following the same procedures from our publications.[1-3] Freshly distilled acetonitrile was used for kinetic measurements. The pseudo-first order rate constants (k^{pfo}'s) were determined on the SF-61DX2 Hi-Tech KinetAsyst double-mixing stopped-flow instrument. The *Abs* - time data of 12-15 half-lives of reaction were collected to derive the initial k^{pfo} by fitting the 12 half-lives data (~99.98% completion of the reaction). Based on this k^{pfo} value, we calculated the half-life time, and ran three to six formal kinetic measurements over a fixed number (typically 12.5) of half-lives. Using the software within the instrument, we fitted 12 half-lives of the *Abs* - time data (Tables 1 to 8). For the Tables S9 and S10 results only, due to slow reactions, data for 1.75 half-lives were collected and the 1.5 half-lives data was fitted to determine the k^{pfo}. If a small spike of the absorbance change is observed at the very beginning due to solution mixing, the data selected for the fit would exclude that part of the data (within first 1% of the reaction). Figure S1 shows one example as to how the fit behaves and how the k^{pfo} is derived.

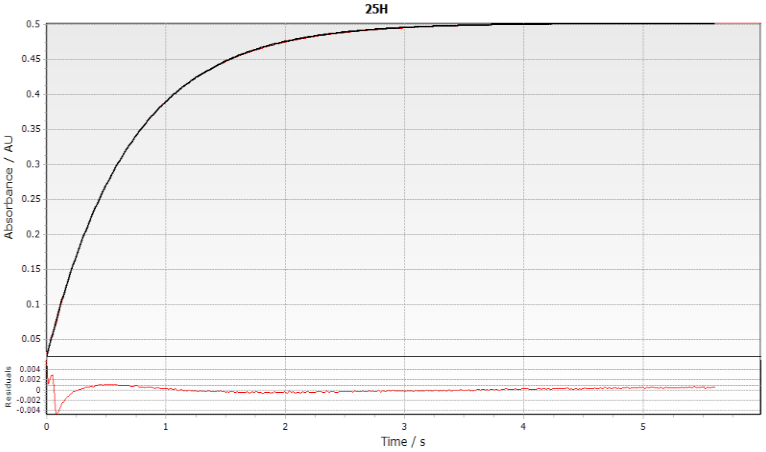

Figure S1. The fit to the Abs - time data for the reaction between HAH (4.50 x 10^{-4} M) and $Tr^+BF_4^-$ (2.00 x 10^{-2} M) in acetonitrile at 25°C at 436 nm (due to HA^+ formation) following the first-order kinetic law (Definition: Y = -A*exp(-R*X) + C; R is k^{pfo}). This is copied from the Kinetic Studio report generated from the fitting software from the instrument. The red Abs - time trace (with 12.5 $t_{1/2}$) is the experimental data, and the black curve (with 12.0 $t_{1/2}$) is the fit.

Measurements of k^{pfo}'s for second-order rate constant (k_2) and KIE derivations were performed over a temperature range of 40 °C at the same day and repeated on two to three additional days (unless otherwise noted in the kinetic data tables (Tables S2 to S11)). A typical kinetic procedure is as follows. Three or six consecutive kinetic runs (each covering 12.5 half-lives) were conducted back-to-back for each isotopic reaction. The procedure was then repeated at other temperatures as quickly as possible (*e.g.*, 15, 25, 35, 45, 55 °C, in order) to maintain the constant instrument settings and minimize possible aging of the reaction solutions. (The reaction solutions were wrapped with aluminum foil and kept in a refrigerator between runs to avoid introducing any unknown impurities with time.) Repetitions of kinetic measurements sometimes used different batches of substrates and sometimes were done by different lab workers, for the purpose to eliminate the possible effect from unknown impurity or human errors on the KIE measurements. The second-order rate constants (k_2) was calculated from k^{pfo}/[excess substrate] to derive KIEs. Therefore, one KIE value was obtained from *at least* 9 repetitions (3 days of measurements with 3 (or 6, in some experiments) repetitions each day, unless otherwise noted in the kinetic data tables).

Arrhenius correlation of k_2's was used to derive E_a's (see examples in Figure S2). The correlation coefficient R^2 values range from 0.9990 to 1.0000 (mostly close to or exactly 1.0000). Standard deviation for each E_a was calculated using the Excel LINEST

formula and the deviation for ΔE_a was derived based on the standard deviations for E_{aH} and E_{aD}, respectively. Pooled standard deviations were reported, and the standard deviations for the average values from different days of measurement are also provided in Tables S1 to S10 for comparison.

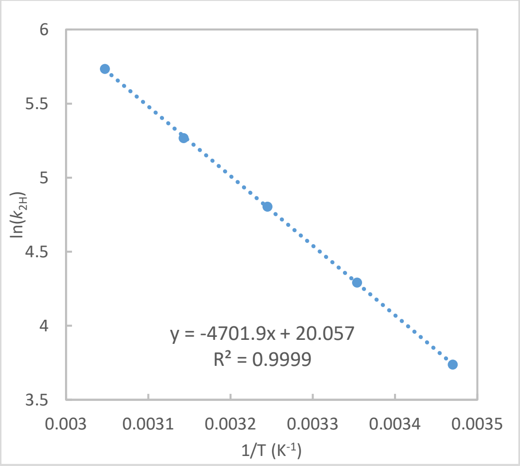

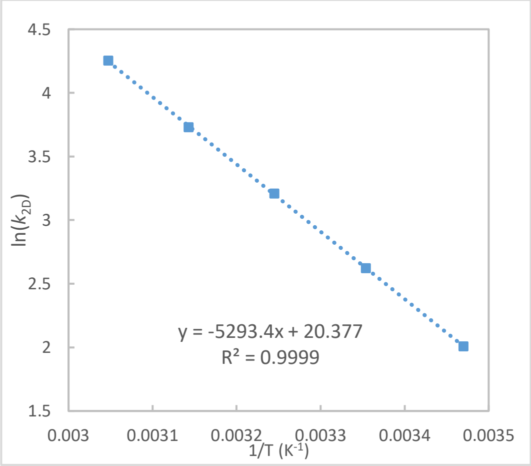

Figure S2. Exemplified Arrhenius plots of rates for hydride (left) and deuteride (right) transfer reactions from HAH and HAH-d_2 to Tr^+ in acetonitrile (temperatures are 15, 25, 35, 45, and 55 °C, respectively). Data are from Day 1 measurement for this reaction. The same plots for other day's measurements or for other reactions can be drawn using the data in the subsequent Data Availability Statement.

The kinetic procedure for the hydride transfer reactions of HEH with NBMN and TBMN at 25 °C has been reported in the literature.[5] Absorbance decay at 380 nm for the reaction of NBMN and 360 nm for the reaction of TBMN due to consumption of HEH was followed for kinetic measurements.

Plots of Temperature Dependence of KIEs

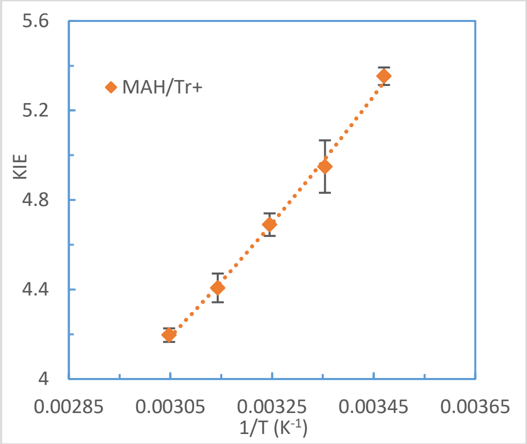

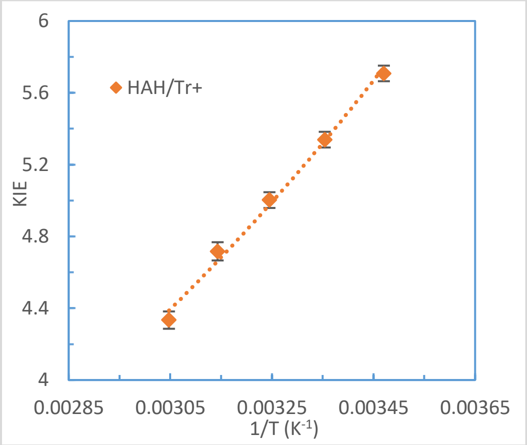

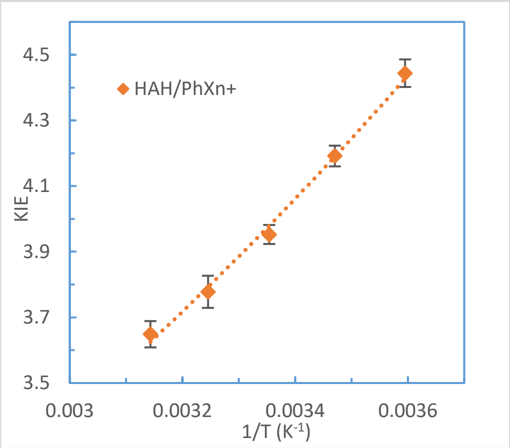
HAH/PhXn+
KIE
4.5
4.3
4.1
3.9
3.7
3.5
0.003
0.0032
0.0034
0.0036
1/T (K-1)

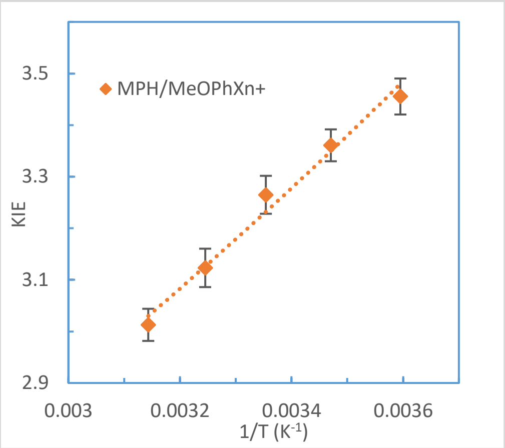
MPH/MeOPhXn+
KIE
3.5
3.3
3.1
2.9
0.003
0.0032
0.0034
0.0036
1/T (K-1)

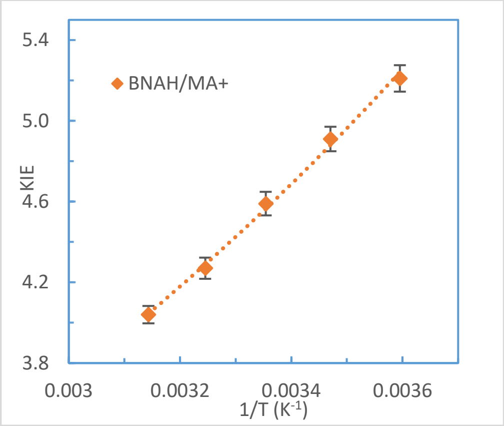
BNAH/MA+
KIE
5.4
5.0
4.6
4.2
3.8
0.003
0.0032
0.0034
0.0036
1/T (K-1)

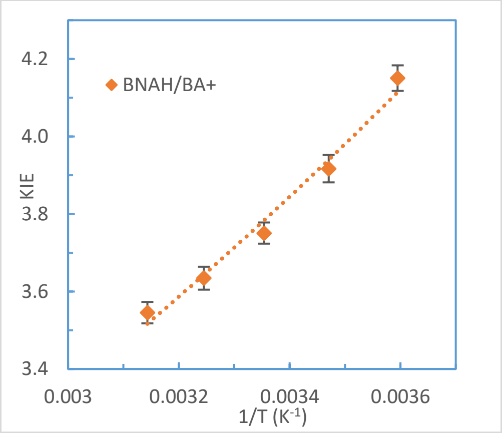
BNAH/BA+
KIE
4.2
4.0
3.8
3.6
3.4
0.003
0.0032
0.0034
0.0036
1/T (K-1)

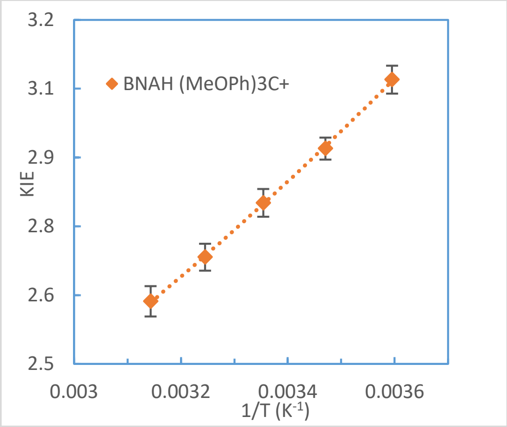
BNAH (MeOPh)3C+
KIE
3.2
3.1
2.9
2.8
2.6
2.5
0.003
0.0032
0.0034
0.0036
1/T (K-1)

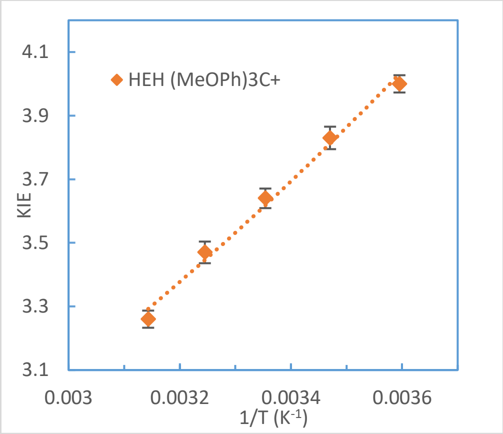
HEH (MeOPh)3C+
KIE
4.1
3.9
3.7
3.5
3.3
3.1
0.003
0.0032
0.0034
0.0036
1/T (K-1)

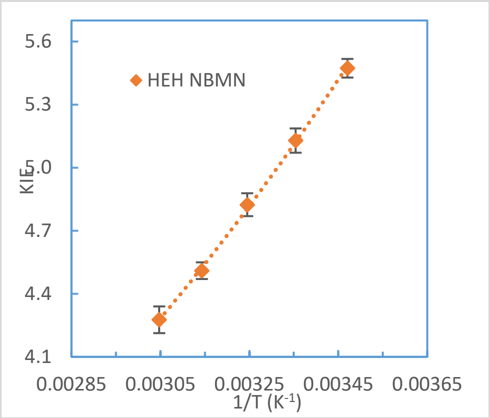

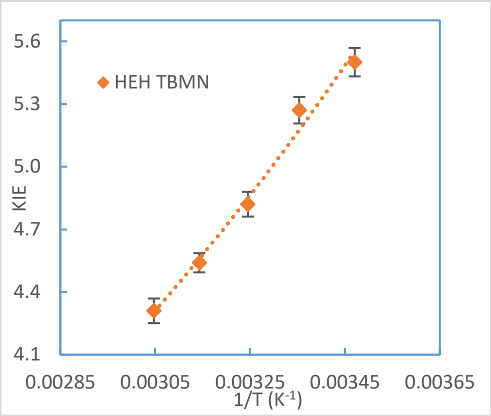

Figure S3. The Arrhenius plots of KIEs for hydride transfer reactions in acetonitrile. Lines represent nonlinear regression using an Arrhenius-type exponential equation (KIE *vs.* EXP(1/T)).

Kinetic Data

Table S1. The temperature effects on the rate constants and 1° KIEs of the hydride transfer reaction from HAH to $Tr^+BF_4^-$ in acetonitrile[a,b]

**Temp (°C)**	k_{2H} **($M^{-1}s^{-1}$)**	k_{2D} **($M^{-1}s^{-1}$)**	**1° KIE**
55.0	3.08(0.03)(0.01)x10^2	7.11(0.05)(0.08)x10	4.33(0.05)(0.05)
45.0	1.97(0.01)(0.03)x10^2	4.18(0.03)(0.04)x10	4.72(0.05)(0.06)
35.0	1.23(0.01)(0.02)x10^2	2.46(0.02)(0.03)x10	5.00(0.04)(0.05)
25.0	7.33(0.05)(0.05)x10	1.37(0.01)(0.01)x10	5.34(0.04)(0.04)
15.0	4.22(0.02)(0.04)x10	7.40(0.05)(0.01)	5.71(0.04)(0.06)

ΔE_a = 1.27(0.09)(0.07)

[a] Repeated on four different days with three repetitions each day. Numbers in the first parenthesis for each value are the pooled standard deviations S(pooled), numbers in the second parenthesis are the standard deviations of the four average values from four days of measurements; [b][HAH] = 0.00045 M, [Tr^+] = 0.020 M. Absorbance growth at 436 nm due to HA^+ was followed for kinetic measurements.

Table S2. The temperature effects on the rate constants and 1° KIEs of the hydride transfer reaction from MAH to $Tr^+BF_4^-$ in acetonitrile [a,b]

**Temp (°C)**	k_{2H} **($M^{-1}s^{-1}$)**	k_{2D} **($M^{-1}s^{-1}$)**	**1° KIE**
55.0	2.41(0.01)(0.01)x10	5.74(0.03)(0.07)	4.20(0.03)(0.06)
45.0	1.38(0.01)(0.02)x10	3.13(0.04)(0.11)	4.41(0.06)(0.11)
35.0	7.60(0.07)(0.01)	1.61(0.00)(0.04)	4.69(0.05)(0.04)
25.0	4.01(0.02)(0.02)	8.10(0.18)(0.14)x10^{-1}	4.95(0.12)(0.07)
15.0	2.03(0.01)(0.01)	3.80(0.02)(0.03)x10^{-1}	5.35(0.04)(0.06)

ΔE_a = 1.14(0.10)(0.07)

[a] Repeated on three different days with three repetitions each day. Numbers in the first parenthesis for each value are the pooled standard deviations S(pooled), numbers in the second parenthesis are the standard deviations of the three average values from three days of measurements; [b][MAH] = 0.000352 M, [Tr^+] = 0.020 M. Absorbance growth at 436 nm due to MA^+ was followed for kinetic measurements.

Table S3. The temperature effects on the rate constants and 1° KIEs of the hydride transfer reaction from HAH to $PhXn^{+}BF_4^{-}$ in acetonitrile [a,b]

Temp (°C)	k_{2H} ($M^{-1}s^{-1}$)	k_{2D} ($M^{-1}s^{-1}$)	1° KIE
45.0	2.39(0.02)(0.07) x 10^3	6.56(0.04)(0.04)x10^2	3.65(0.04)(0.16)
35.0	1.67(0.02)(0.05) x 10^3	4.43(0.03)(0.28)x10^2	3.78(0.05)(0.15)
25.0	1.15(0.01)(0.03) x 10^3	2.91(0.02)(0.17)x10^2	3.95(0.02)(0.17)
15.0	7.68(0.04)(0.23) x 10^2	1.83(0.01)(0.10)x10^2	4.19(0.03)(0.17)
5.0	4.95(0.01)(0.12) x 10^2	1.12(0.01)(0.06)x10^2	4.44(0.04)(0.20)
	ΔE_a = 0.88 (0.05)(0.06)		

[a] Repeated on four different days with six repetitions each day. Numbers in the first parenthesis for each value are the pooled standard deviations S(pooled), numbers in the second parenthesis are the standard deviations of the four average values from four days of measurements; [b][HAH] = 0.002 M, [$PhXn^+$] = 0.0002 M. Absorbance decay at 473 nm due to $PhXn^+$ was followed for kinetic measurements.

Table S4. The temperature effects on the rate constants and 1° KIEs of the hydride transfer reaction from MPH (excess) to $MeOPhXn^{+}BF_4^{-}$ in acetonitrile[a]

Temp (°C)	k_{2H} ($M^{-1}s^{-1}$)	k_{2D} ($M^{-1}s^{-1}$)	1° KIE
45.0[b]	3.37(0.02)(0.13) x10^3	1.09(0.01)(0.08) x10^3	3.10(0.02)(0.11)
35.0[b]	2.30(0.01)(0.08) x10^3	7.15(0.05)(0.50) x10^2	3.22(0.03)(0.11)
25.0[b]	1.52(0.01)(0.06) x10^3	4.54(0.02)(0.33) x10^2	3.34(0.02)(0.10)
15.0[b]	9.81(0.06)(0.34) x10^2	2.83(0.01)(0.21) x10^2	3.47(0.03)(0.13)
5.0[b]	6.09(0.04)(0.21) x10^2	1.70(0.01)(0.12) x10^2	3.59(0.03)(0.14)
	ΔE_a = 0.64(0.03)(0.02)[d]		
45.0[c]	3.97(0.03)(0.02) x10^3	1.34(0.01)(0.01) x10^3	2.96(0.03)(0.01)
35.0[c]	2.67(0.02)(0.02) x10^3	8.69(0.08)(0.19) x10^2	3.07(0.04)(0.04)
25.0[c]	1.78(0.01)(0.00) x10^3	5.54(0.05)(0.06) x10^2	3.21(0.04)(0.04)
15.0[c]	1.15(0.01)(0.01) x10^3	3.46(0.03)(0.05) x10^2	3.31(0.03)(0.02)
5.0[c]	7.03(0.01)(0.00) x10^2	2.07(0.01)(0.01) x10^2	3.41(0.03)(0.02)
	ΔE_a = 0.62(0.06)(0.01)[d]		

[a] Repeated on four different days with six repetitions each day. Absorbance decay was monitored at 496 nm for kinetic measurements due to consumption of $MeOPhXn^+$; [b] For the first two days of collection, [MPH] = 0.623 mM, [$MeOPhXn^+BF_4^+$] = 0.0623 mM was used to measure kinetics (done by Pratichhya Adhikari in 2020); Numbers in the first parenthesis are the pooled standard deviations S(pooled), numbers in the second parenthesis are the standard deviations of the two average values from two days measurements; [c] For the third and fourth days [MPH] = 2 mM, [$MeOPhXn^+BF_4^-$] = 0.0623 mM concentrations were used for kinetics solutions (done by Ava Austin-Kloppe in 2025); Numbers in the first parenthesis are the pooled standard deviations S(pooled), numbers in the second parenthesis are the standard deviations of the two average values from two days measurements; [d] The two workers used different batch of chemicals and different concentrations. Data reported in Table S1 are the average of all measurements by the two workers and the corresponding standard deviation is the pooled standard deviation of all four days measurements.

Table S5. The temperature effects on the rate constants and 1° KIEs of the hydride transfer reaction from BNAH to (p-$CH_3OPh)_3C^{+}BF_4^{-}$ in acetonitrile[a,b]

Temp (°C)	k_{2H} ($M^{-1}s^{-1}$)	k_{2D} ($M^{-1}s^{-1}$)	1° KIE
45.0	2.41(0.02)(0.02) x 10^5	9.34(0.08)(0.28) x 10^4	2.59 (0.03)(0.09)
35.0	2.02(0.02)(0.00) x 10^5	7.52(0.05)(0.23) x 10^4	2.68 (0.03)(0.08)
25.0	1.66(0.01)(0.02) x 10^5	5.93(0.05)(0.15) x 10^4	2.80 (0.03)(0.11)
15.0	1.34(0.01)(0.01) x 10^5	4.58(0.02)(0.11) x 10^4	2.92 (0.02)(0.09)
5.0	1.06(0.01)(0.01) x 10^5	3.47(0.02)(0.07) x 10^4	3.07 (0.03)(0.07)

ΔE_a = 0.75 (0.05)(0.05)

[a] Repeated on two different days with 6 repetitions each day. The last two sets of data were collected in the same day. Numbers in parentheses are the pooled standard deviations S(pooled); numbers in the second parenthesis are the standard deviations of the three average values from three days of measurements; [b] [HEH] = 3.89x10^{-4} M, [$(CH_3OPh)_3C^+$] = 7.79x10^{-6} M. Absorbance decay at 480 nm due to $(CH_3OPh)_3C^+$ was followed for kinetic measurements.

Table S6. The temperature effects on the rate constants and 1° KIEs of the hydride transfer reaction from HEH to (*p*-$CH_3OPh)_3C^+BF_4^-$ in acetonitrile [a,b]

Temp (°C)	k_{2H} ($M^{-1}s^{-1}$)	k_{2D} ($M^{-1}s^{-1}$)	1° KIE
45.0	9.97(0.07)(0.29) x 10^3	3.06(0.01)(0.05) x 10^3	3.26 (0.03)(0.07)
35.0	7.63(0.05)(0.22) x 10^3	2.20(0.02)(0.05) x 10^3	3.47 (0.03)(0.06)
25.0	5.66(0.03)(0.13) x 10^3	1.55(0.01)(0.02) x 10^3	3.64 (0.03)(0.06)
15.0	4.09(0.03)(0.11) x 10^3	1.07(0.01)(0.01) x 10^3	3.83 (0.04)(0.04)
5.0	2.87(0.02)(0.08) x 10^3	7.17(0.02)(0.08) x 10^2	4.00 (0.03)(0.03)

ΔE_a = 0.88 (0.06)(0.01)

[a] Repeated on three different days with 6 repetitions each day. Numbers in parentheses are the pooled standard deviations S(pooled); numbers in the second parenthesis are the standard deviations of the three average values from three days of measurements; [b] [HEH] = 1.17x10^{-3} M, [$(CH_3OPh)_3C^+$] = 7.79x10^{-6} M. Absorbance decay at 480 nm due to $(CH_3OPh)_3C^+$ was followed for kinetic measurements.

Table S7. The temperature effects on the rate constants and 1° KIEs of the hydride transfer reaction from BNAH to $MA^+BF_4^-$ in acetonitrile [a,b]

Temp (°C)	k_{2H} ($M^{-1}s^{-1}$)	k_{2D} ($M^{-1}s^{-1}$)	1° KIE
45.0	1.53(0.01)(0.06) x 10^2	3.78(0.03)(0.14) x 10	4.04 (0.04)(0.16)
35.0	1.05(0.01)(0.02) x 10^2	2.46(0.02)(0.12) x 10	4.27 (0.05)(0.16)
25.0	7.16(0.07)(0.13) x 10	1.57(0.01)(0.12) x 10	4.59 (0.06)(0.27)
15.0	4.47(0.04)(0.25) x 10	9.77(0.08)(1.03)	4.91 (0.06)(0.29)
5.0	3.20(0.03)(0.36) x 10	6.16(0.07)(0.95)	5.21 (0.07)(0.26)

ΔE_a = 1.14 (0.17)(0.12)

[a] Repeated on three different days with six repetitions each day (Day 1 results from Jessica Sager and Days 2 and 3 results are from Ava Austin-Kloppe). Numbers in the first parenthesis for each value are the pooled standard deviations S(pooled), numbers in the second parenthesis are the standard deviations of the three average values from three days of measurements; [b] [BNAH] = 3.00 x10^{-3} M, [MA^+] = 3.00 x10^{-4} M. Absorbance decay at 436 nm due to consumption of MA^+ was followed for kinetic measurements.

Table S8. The temperature effects on the rate constants and 1° KIEs of the hydride transfer reaction from BNAH to $BA^+BF_4^-$ in acetonitrile [a,b]

Temp (°C)	k_{2H} ($M^{-1}s^{-1}$)	k_{2D} ($M^{-1}s^{-1}$)	1° KIE
45.0	4.85(0.03)(0.09) x 10^2	1.22(0.01)(0.01) x 10^2	3.55 (0.03)(0.04)
35.0	3.46(0.02)(0.01) x 10^2	8.48(0.06)(0.01) x 10	3.63 (0.04)(0.01)
25.0	2.48(0.02)(0.03) x 10^2	5.84(0.04)(0.04) x 10	3.75 (0.03)(0.01)
15.0	1.73(0.01)(0.01) x 10^2	3.88(0.03)(0.03) x 10	3.92 (0.04)(0.00)
5.0	1.20(0.01)(0.03) x 10^2	2.51(0.02)(0.04) x 10	4.15 (0.04)(0.04)

ΔE_a = 0.81 (0.09)(0.00)

[a] Repeated on two different days with six repetitions each day. Numbers in the first parenthesis for each value are the pooled standard deviations S(pooled), numbers in the second parenthesis are the standard deviations of the two average values from two days of measurements; [b] [BNAH] = 3.00 x10^{-3} M, [BA^+] = 3.00 x10^{-4} M. Absorbance decay at 436 nm due to consumption of BA^+ was followed for kinetic measurements.

Table S9. The temperature effects on the rate constants and 1° KIEs of the hydride transfer reaction from HEH to NBMN in acetonitrile [a,b]

Temp (°C)	k_{2H} ($M^{-1}s^{-1}$)	k_{2D} ($M^{-1}s^{-1}$)	1° KIE
55.0	4.17(0.06)(0.09)	9.75(0.03)(0.30) x10^{-1}	4.28(0.06)(0.03)
45.0	2.80(0.01)(0.05)	6.20(0.03)(0.17) x10^{-1}	4.51(0.04)(0.06)
35.0	1.83(0.01)(0.06)	3.79(0.03)(0.01) x10^{-1}	4.82(0.05)(0.04)
25.0	1.14(0.01)(0.02)	2.22(0.02)(0.01) x10^{-1}	5.13(0.06)(0.17)
15.0	6.73(0.02)(0.20) x10^{-1}	1.23(0.01)(0.01) x10^{-1}	5.47(0.04)(0.15)
	ΔE_a = 1.17 (0.15)(0.09)		

[a] Repeated on three different days with three repetitions each day. Numbers in the first parenthesis for each value are the pooled standard deviations S(pooled), numbers in the second parenthesis are the standard deviations of the three average values from three days of measurements; [b] [HEH] = 2.00 x 10^{-4} M, [NBMN] = 8.00x10^{-3} M. Absorbance decay at 380 nm due to consumption of HEH was followed for kinetic measurements.

Table S10. The temperature effects on the rate constants and 1° KIEs of the hydride transfer reaction from HEH to TBMN in acetonitrile [a,b]

Temp (°C)	k_{2H} ($M^{-1}s^{-1}$)	k_{2D} ($M^{-1}s^{-1}$)	1° KIE
55.0	2.23(0.03)(0.04)	5.18(0.04)(0.14) x10^{-1}	4.31(0.06)(0.04)
45.0	1.48(0.01)(0.04)	3.27(0.02)(0.08) x10^{-1}	4.54(0.05)(0.01)
35.0	9.65(0.10)(0.24) x10^{-1}	2.00(0.01)(0.06) x10^{-1}	4.82(0.06)(0.02)
25.0	6.07(0.02)(0.23) x10^{-1}	1.15(0.01)(0.03) x10^{-1}	5.27(0.06)(0.24)
15.0	3.55(0.02)(0.12) x10^{-1}	6.45(0.06)(0.28) x10^{-2}	5.50(0.07)(0.14)
	ΔE_a = 1.19 (0.16)(0.02)		

[a] Repeated on three different days with three repetitions each day. Numbers in the first parenthesis for each value are the pooled standard deviations S(pooled), numbers in the second parenthesis are the standard deviations of the three average values from three days of measurements; [b] [HEH] = 2.00 x 10^{-4} M, [TBMN] = 1.20 x 10^{-2} M. Absorbance decay at 360 nm due to consumption of HEH was followed for kinetic measurements.

References

1. Singh, G.; Austin, A.; Bai, M.; Bradshaw, J.; Hammann, B. A.; Kabotso, D. E. K.; Lu, Y. Study of the Effects of Remote Heavy Group Vibrations on the Temperature Dependence of Hydride Kinetic Isotope Effects of the NADH/NAD+ Model Reactions. *ACS Omega* **2024**, *9*, 20593-20600.
2. Beach, A.; Adhikari, P.; Singh, G.; Song, M.; DeGroot, N.; Lu, Y. Structural Effects on the Temperature Dependence of Hydride Kinetic Isotope Effects of the NADH/NAD+ Model Reactions in Acetonitrile: Charge-Transfer Complex Tightness Is a Key. *J. Org. Chem* **2024**, *89*, 3184–3193.
3. Austin, A.; Sager, J.; Phan, L.; Lu, Y. Structural Effects on the Hydride-Tunneling Kinetic Isotope Effects of NADH/NAD+ Model Reactions: Relating to the Donor–Acceptor Distances. *J. Org. Chem.* **2025**, *90*, 3110-3115.
4. Shen, G. B.; Xia, K.; Li, X. T.; Li, J. L.; Fu, Y. H.; Yuan, L.; Zhu, X. Q. Prediction of Kinetic Isotope Effects for Various Hydride Transfer Reactions Using a New Kinetic Model. *J. Phys. Chem. A* **2016**, *120*, 1779−1799.
5. Zhu, X.-Q.; Zou, H.-L.; Yuan, P.-W.; Liu, Y.; Cao, L.; Cheng, J.-P. A Detailed investigation into the oxidation mechanism of Hantzsch 1,4-dihydropyridines by ethyl -cyanocinnamates and benzylidenemalononitriles. *J. Chem. Soc., Perkin Trans. 2* **2000**, 1857–1861.

Data Availability Statement

Primary kinetic data for Tables S1-S10 are presented below. We directly copied the original data from the corresponding excel data file. Due to the decimal point place difference in between the two places, data may slightly differ at the last digit of their numbers. Meanwhile, we provide the *Abs* – time (t) data (plots) and the corresponding fit for the measurements of the pseudo first-order rate constants (k^{pfo}'s).

Primary kinetic data for the rate constants in Table S1 (HAH with Tr^+)

Day 1 data (July 28, 2025)

Pseudo-first-order rate constants (k^{pfo} (s^{-1}))

Temp (°C)	Trial H1	Trial H2	Trial H3	Average k_H^{pfo} (s^{-1})	Stdev	k_{2H} ($M^{-1}s^{-1}$)	Stdev [a]
55	6.23509	6.15531	6.15460	6.18167	0.04627	309.08333	2.31337
45	3.87187	3.86915	3.87527	3.87210	0.00307	193.60483	0.15331
35	2.42494	2.43779	2.44694	2.43656	0.01105	121.82783	0.55259
25	1.46298	1.46489	1.45389	1.46059	0.00588	73.02933	0.29388
15	0.83853	0.83869	0.83936	0.83886	0.00044	41.94300	0.02202

Temp (°C)	Trial D1	Trial D2	Trial D3	Average k_D^{pfo} (s^{-1})	Stdev	k_{2D} ($M^{-1}s^{-1}$)	Stdev [a]
55	1.40029	1.40335	1.40790	1.40385	0.00383	70.19233	0.19146
45	0.82666	0.83394	0.83504	0.83188	0.00455	41.59400	0.22770
35	0.49511	0.49397	0.49281	0.49396	0.00115	24.69817	0.05750
25	0.27400	0.27506	0.27585	0.27497	0.00093	13.74850	0.04641
15	1.40029	1.40335	1.40790	1.40385	0.00383	70.19233	0.19146

[a] = (Stdev(for k^{pfo})/k^{pfo})*k_2

<table>
<tr><td>Three kinetic runs together with the averaged data</td><td>First-order kinetic fit of the averaged data as an example</td></tr>
<tr><td>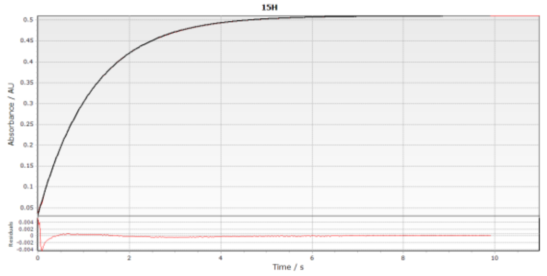
</td><td>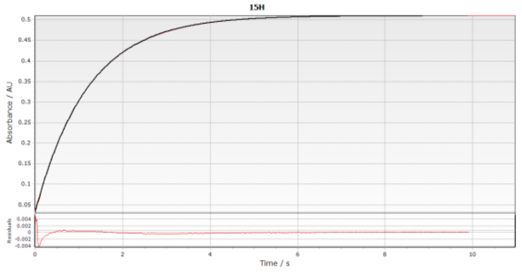
</td></tr>
<tr><td colspan="2">15°C H</td></tr>
<tr><td>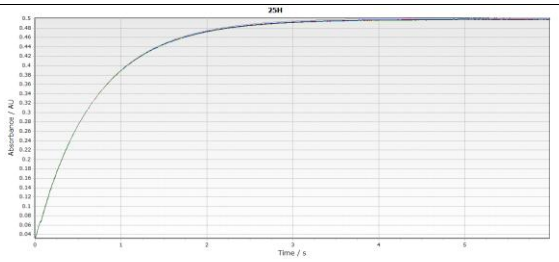
</td><td>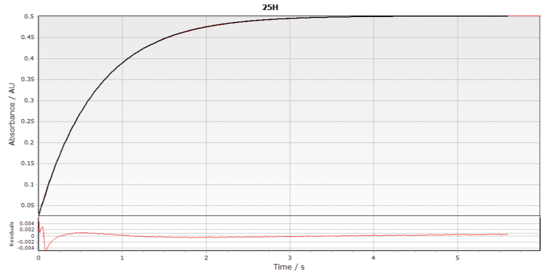
</td></tr>
<tr><td colspan="2">25°C H</td></tr>
</table>

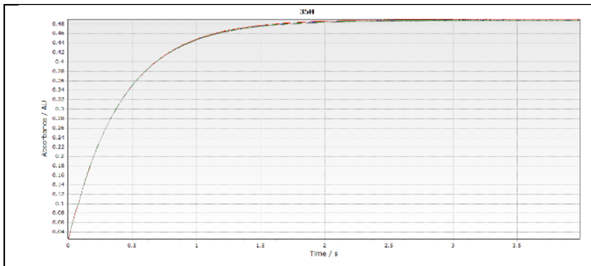

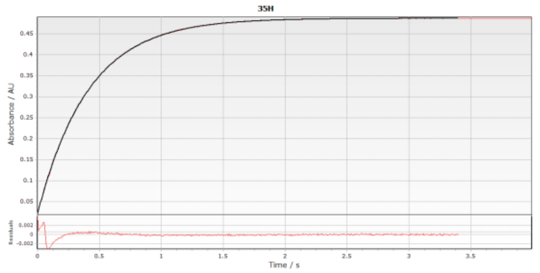

35°C H

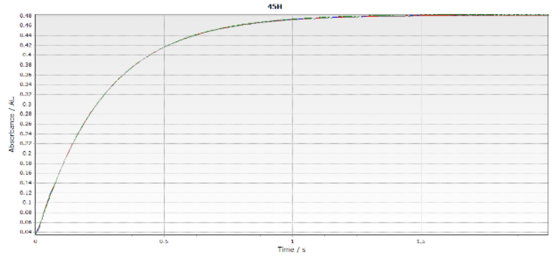

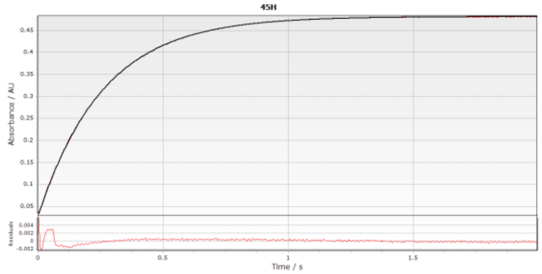

45°C H

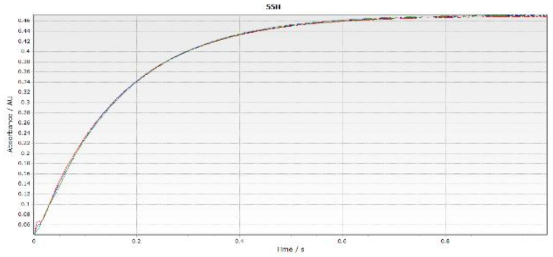

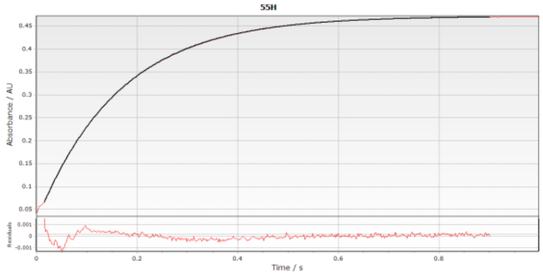

55°C H

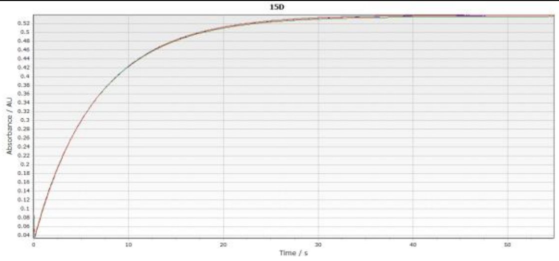

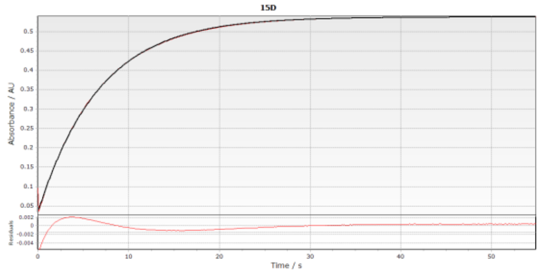

15°C D

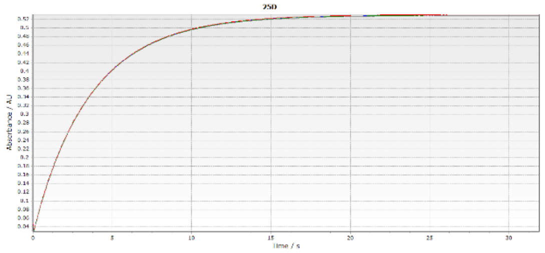

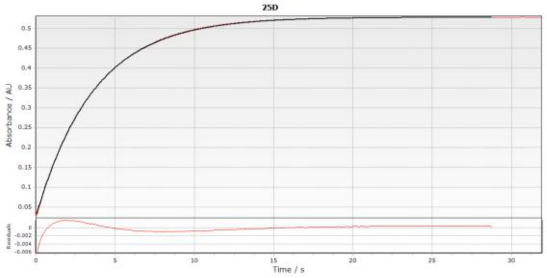

25°C D

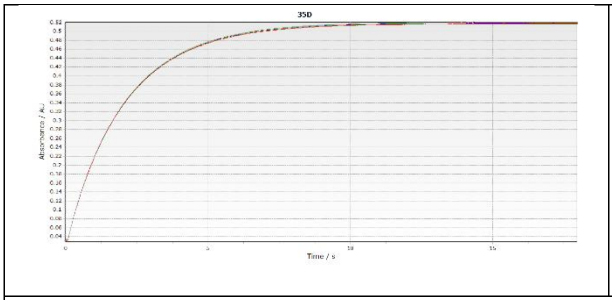

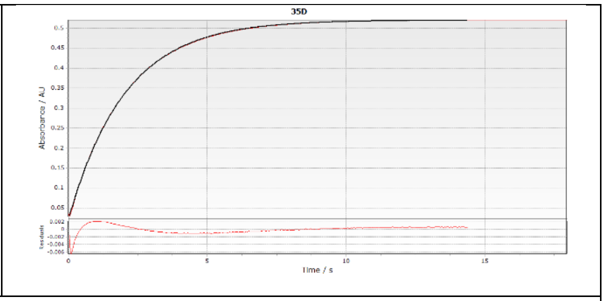

35°C D

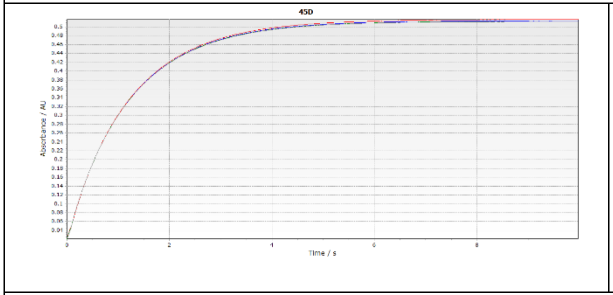

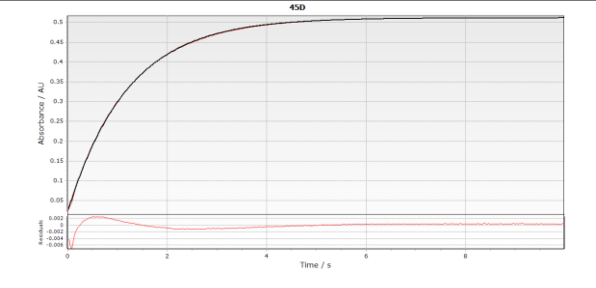

45°C D

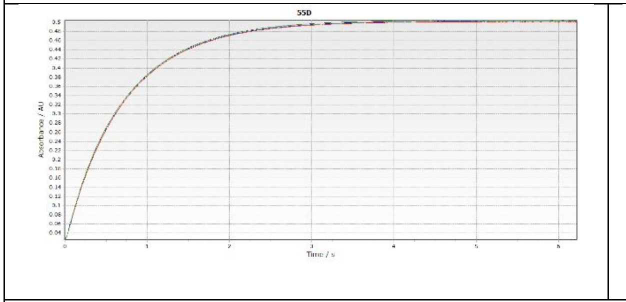

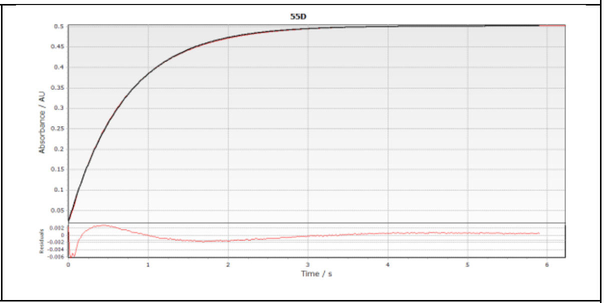

55°C D

Day 2 data (July 29, 2025)

Pseudo-first-order rate constants (k^{pfo} (s^{-1}))

**Temp (°C)**	**Trial H1**	**Trial H2**	**Trial H3**	**Average k_H^{pfo} (s^{-1})**	**Stdev**	**k_{2H} (M^{-1}s^{-1})**	**Stdev [a]**
55	6.12924	6.15305	6.25118	6.17782	0.06463	308.89117	3.23173
45	3.99553	3.99478	4.05057	4.01363	0.03200	200.68133	1.59980
35	2.49651	2.51044	2.50473	2.50389	0.00700	125.19467	0.35013
25	1.46831	1.46337	1.48377	1.47182	0.01064	73.59083	0.53212
15	0.85589	0.85639	0.85106	0.85445	0.00294	42.72233	0.14718

**Temp (°C)**	**Trial D1**	**Trial D2**	**Trial D3**	**Average k_D^{pfo} (s^{-1})**	**Stdev**	**k_{2D} (M^{-1}s^{-1})**	**Stdev [a]**
55	1.42709	1.45379	1.44881	1.44323	0.01420	72.16150	0.70989
45	0.83921	0.82290	0.83556	0.83256	0.00856	41.62783	0.42799
35	0.49490	0.49604	0.50019	0.49704	0.00278	24.85217	0.13920
25	0.27731	0.27621	0.27845	0.27732	0.00112	13.86617	0.05600
15	0.14887	0.14976	0.14977	0.149467	0.000517	7.473333	0.02584

[a] = (Stdev(for k^{pfo})/k^{pfo})*k_2

Three kinetic runs together with the averaged data	First-order kinetic fit of the averaged data as an example
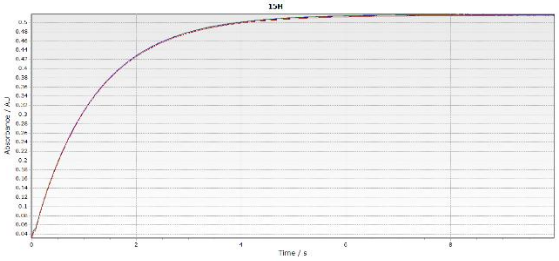	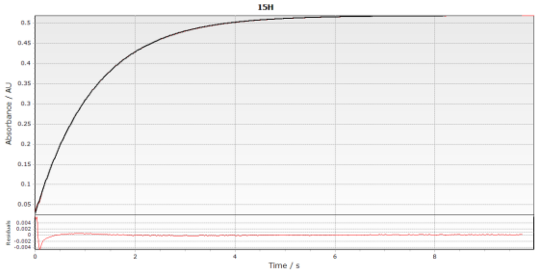
15°C H	
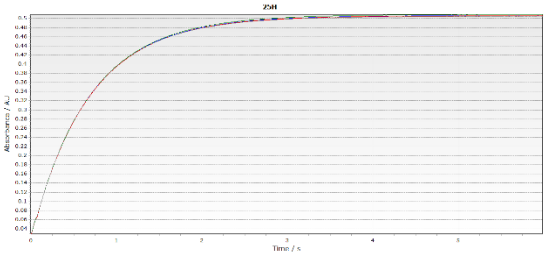	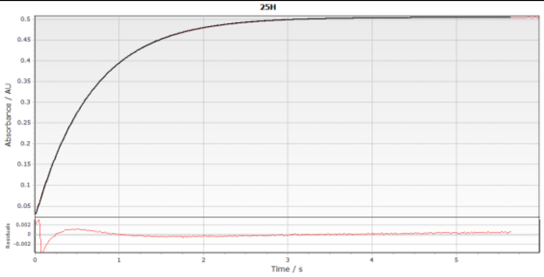
25°C H	
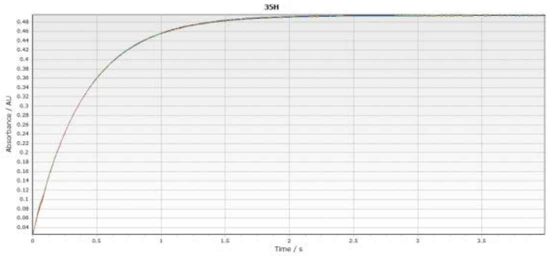	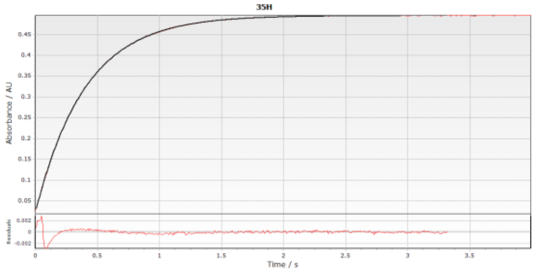
35°C H	
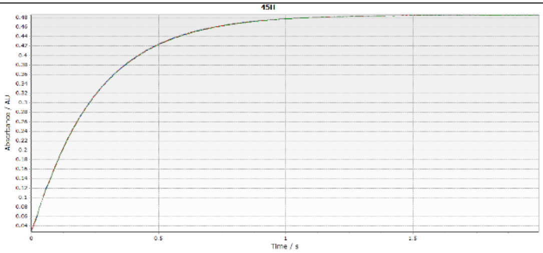	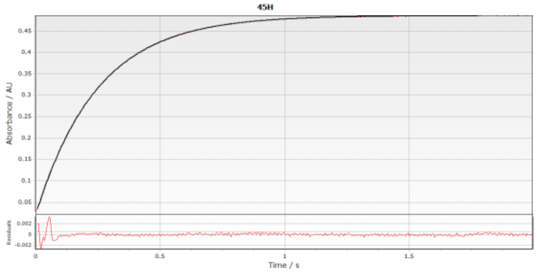
45°C H	
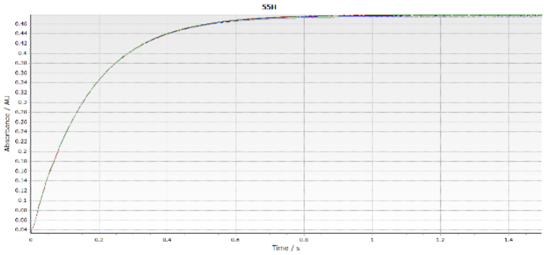	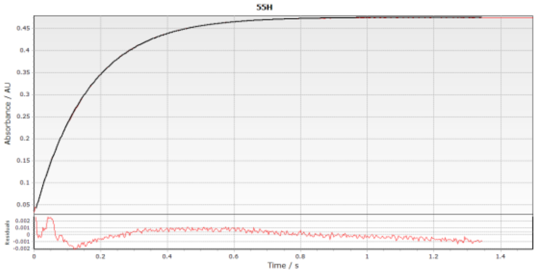
55°C H	

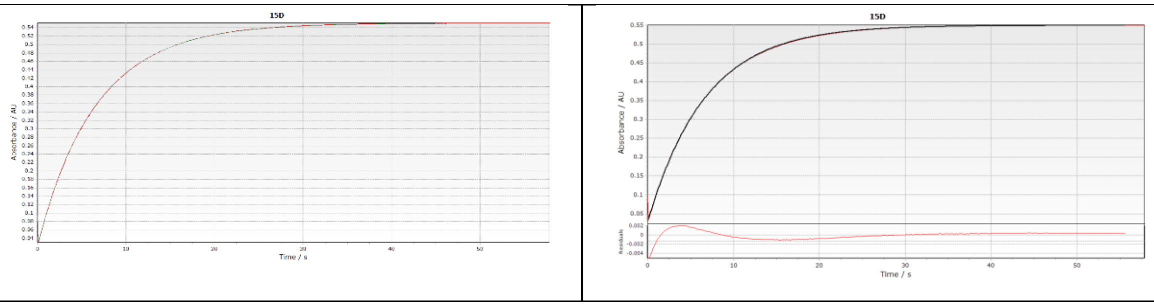

15°C D

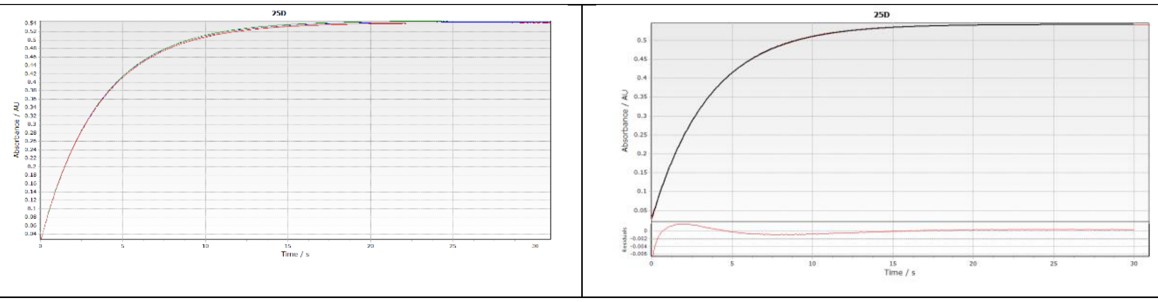

25°C D

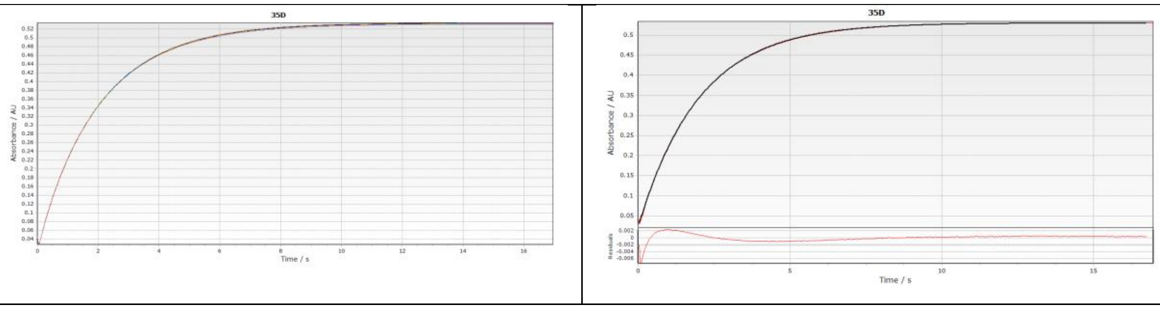

35°C D

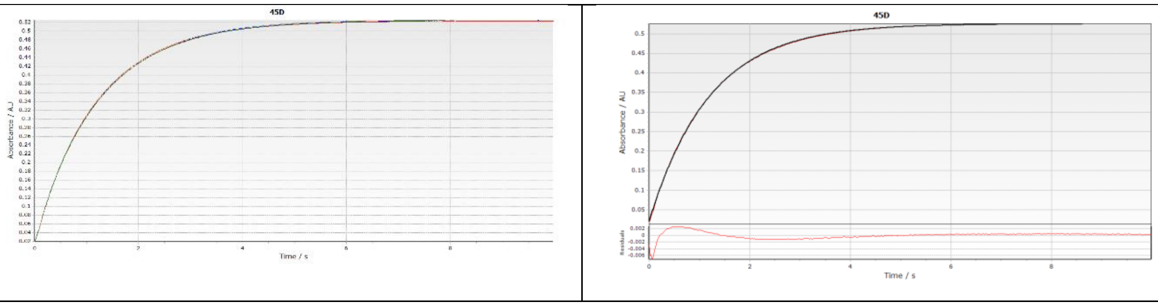

45°C D

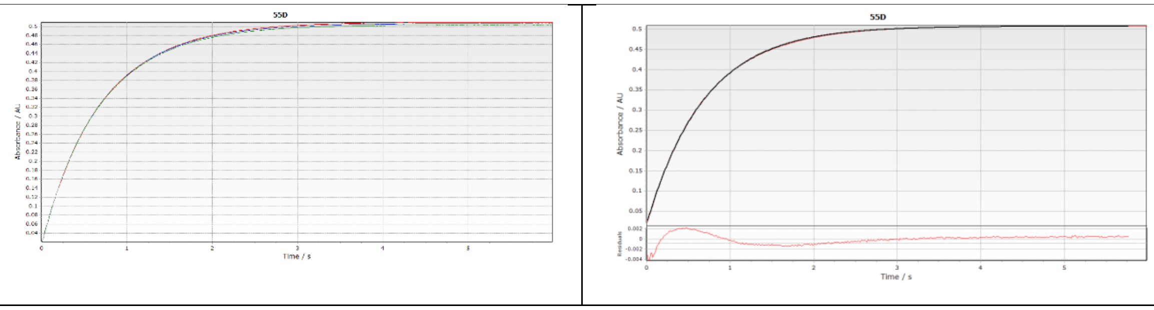

55°C D

Day 3 data (August 1, 2025)

Pseudo-first-order rate constants (k^{pfo} (s^{-1}))

Temp (°C)	Trial H1	Trial H2	Trial H3	Average k_H^{pfo} (s^{-1})	Stdev	k_{2H} (M^{-1}s^{-1})	Stdev [a]
55	6.08736	6.09160	6.17811	6.11902	0.05121	305.95117	2.56072
45	3.95627	3.93184	3.94280	3.94364	0.01224	197.18183	0.61182
35	2.49293	2.48980	2.47281	2.48518	0.01083	124.25900	0.54132
25	1.46553	1.48732	1.47968	1.47751	0.01106	73.87550	0.55279
15	0.84677	0.84320	0.84128	0.84375	0.00279	42.18750	0.13930

Temp (°C)	Trial D1	Trial D2	Trial D3	Average k_D^{pfo} (s^{-1})	Stdev	k_{2D} (M^{-1}s^{-1})	Stdev [a]
55	1.42797	1.41455	1.42886	1.42379	0.00802	71.18967	0.40087
45	0.84777	0.84867	0.85141	0.84928	0.00190	42.46417	0.09480
35	0.49501	0.49095	0.49679	0.49425	0.00299	24.71250	0.14966
25	0.27122	0.27512	0.27471	0.27368	0.00214	13.68417	0.10716
15	0.14659	0.14882	0.14717	0.14753	0.00115	7.37633	0.05784

[a] = (Stdev(for k^{pfo})/k^{pfo})*k_2

Three kinetic runs together with the averaged data	First-order kinetic fit of the averaged data as an example
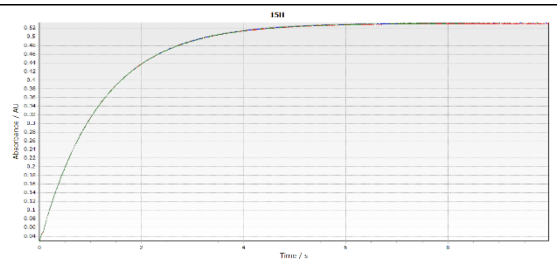	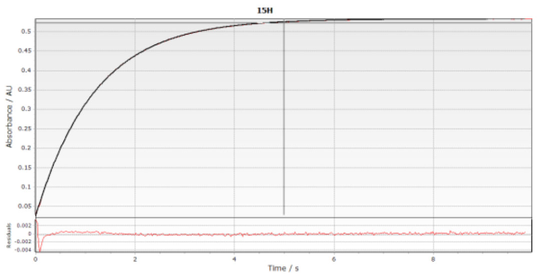
15°C H	
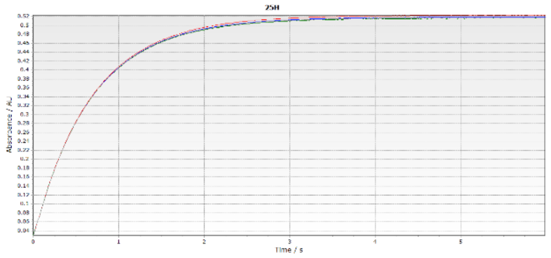	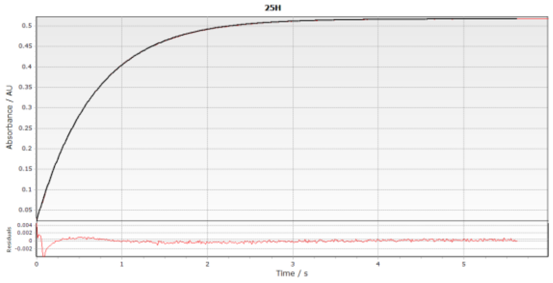
25°C H	
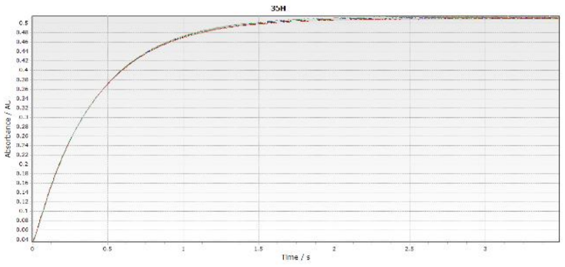	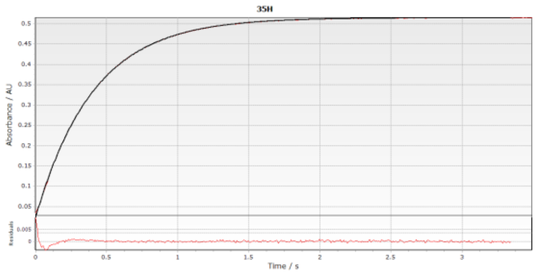
35°C H	

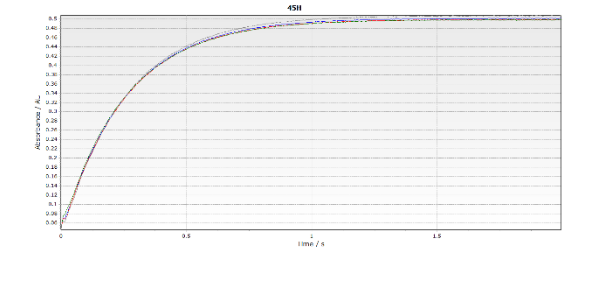

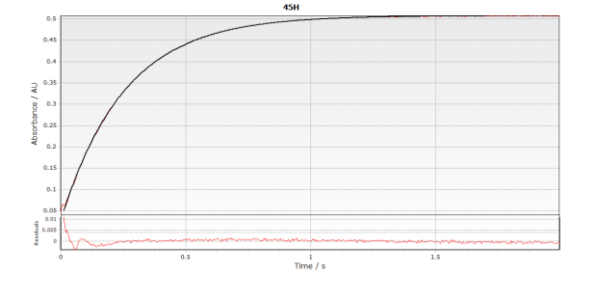

45°C H

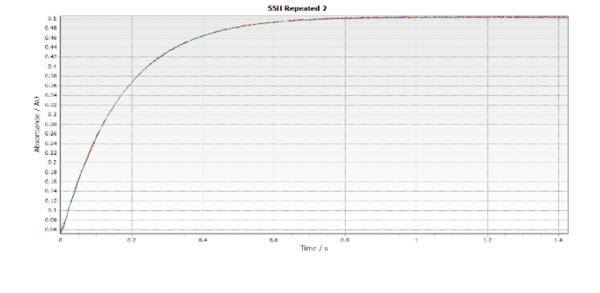

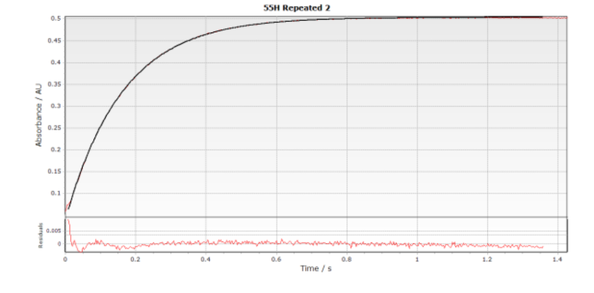

55°C H

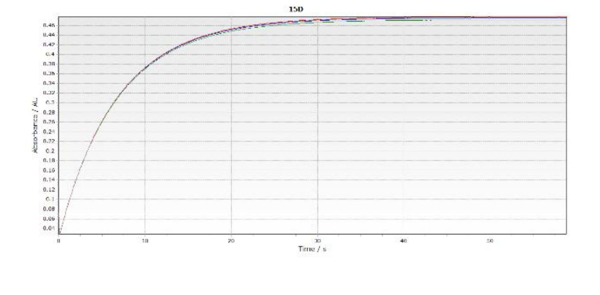

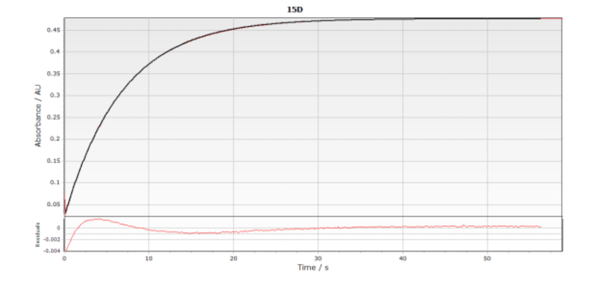

15°C D

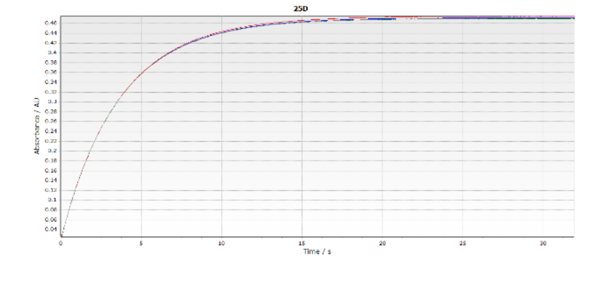

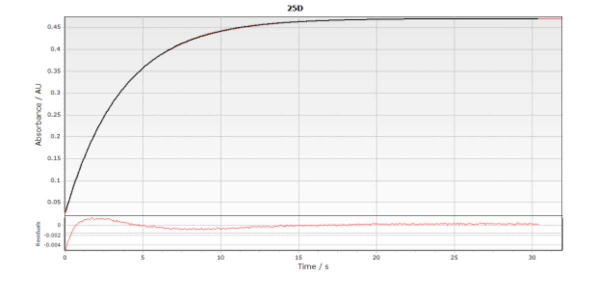

25°C D

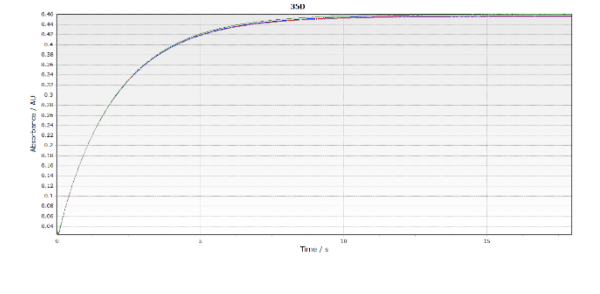

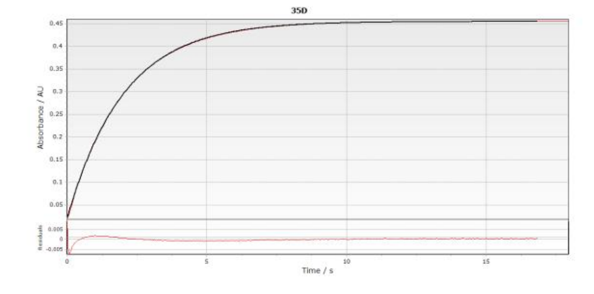

35°C D

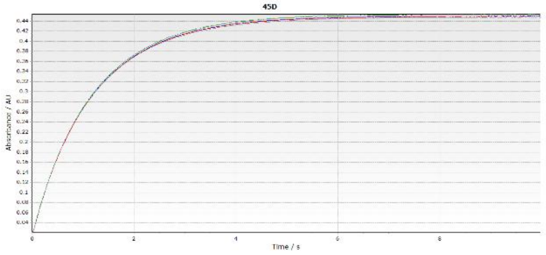

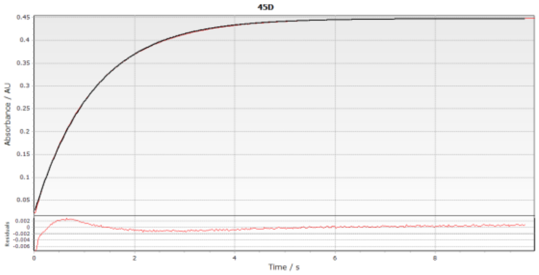

45°C D

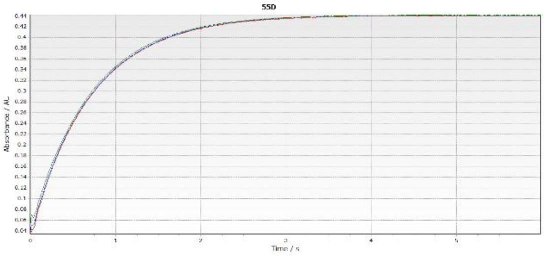

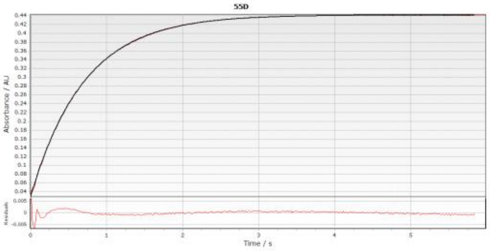

55°C D

Day 4 data (August 6, 2025)

Pseudo-first-order rate constants (k^{pfo} (s^{-1}))

**Temp (°C)**	**Trial H1**	**Trial H2**	**Trial H3**	**Average k_H^{pfo} (s^{-1})**	**Stdev**	**k_{2H} (M^{-1}s^{-1})**	**Stdev [a]**
55	6.14897	6.11595	6.19804	6.15432	0.04131	307.71600	2.06528
45	3.90120	3.98446	3.95765	3.94777	0.04250	197.38850	2.12501
35	2.41969	2.40123	2.40792	2.40961	0.00935	120.48067	0.46729
25	1.46552	1.44581	1.45487	1.45540	0.00987	72.77000	0.49328
15	0.84363	0.84277	0.83349	0.83996	0.00562	41.99817	0.28113

**Temp (°C)**	**Trial D1**	**Trial D2**	**Trial D3**	**Average k_D^{pfo} (s^{-1})**	**Stdev**	**k_{2D} (M^{-1}s^{-1})**	**Stdev [a]**
55	1.41223	1.42695	1.40111	1.41343	0.01296	70.67150	0.64809
45	0.83472	0.83824	0.82075	0.83124	0.00925	41.56183	0.46253
35	0.47395	0.48629	0.48228	0.48084	0.00629	24.04200	0.31474
25	0.27338	0.27247	0.27198	0.27261	0.00071	13.63050	0.03552
15	0.14558	0.14706	0.14477	0.14580	0.00116	7.29017	0.05806

[a] = (Stdev(for k^{pfo})/k^{pfo})*k_2

Three kinetic runs together with the averaged data	First-order kinetic fit of the averaged data as an example
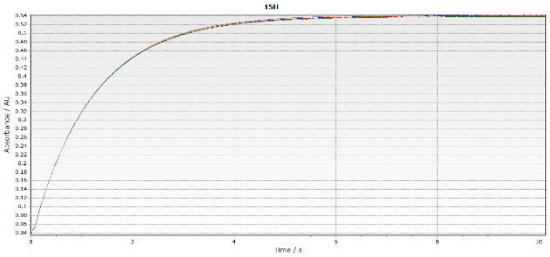	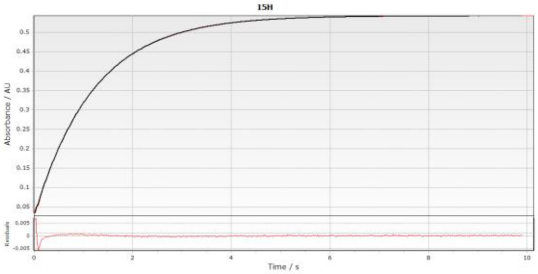

15°C H

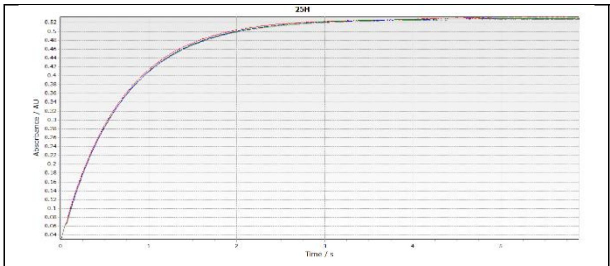	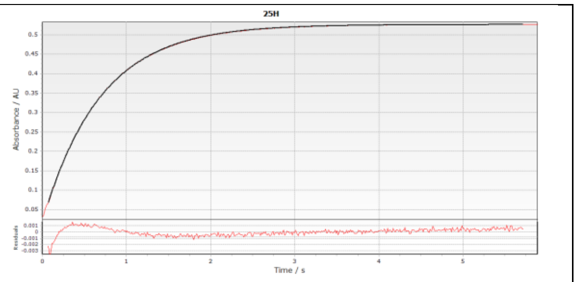
25°C H	
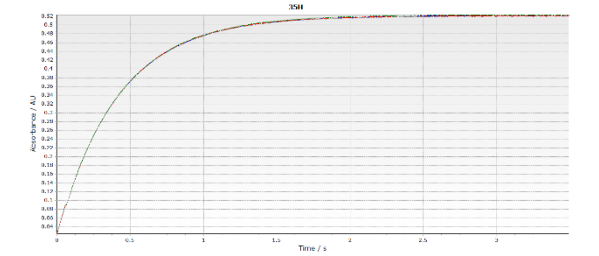	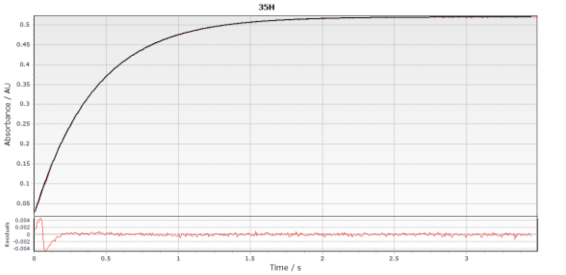
35°C H	
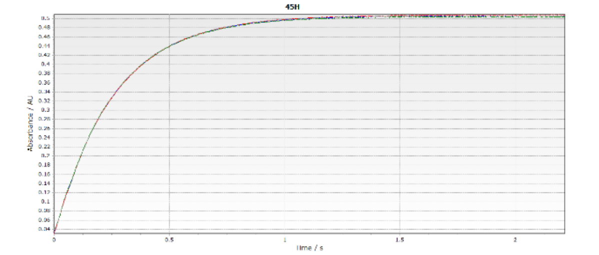	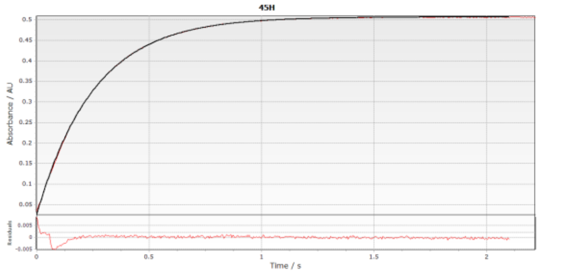
45°C H	
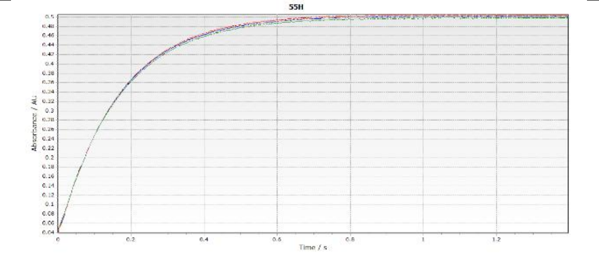	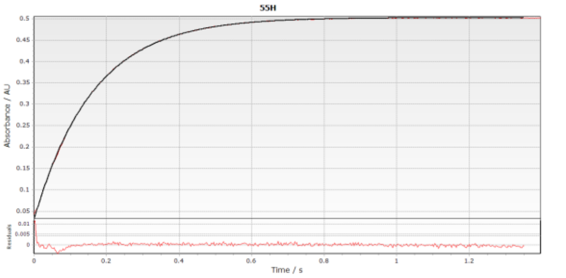
55°C H	
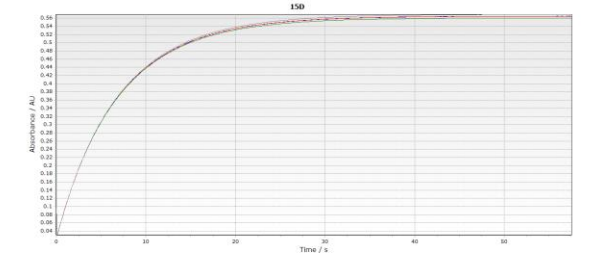	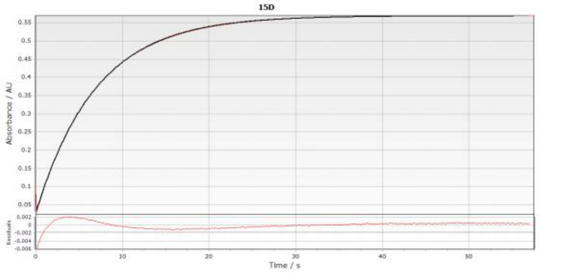
15°C D	

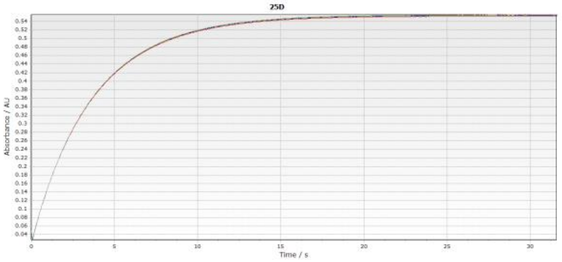

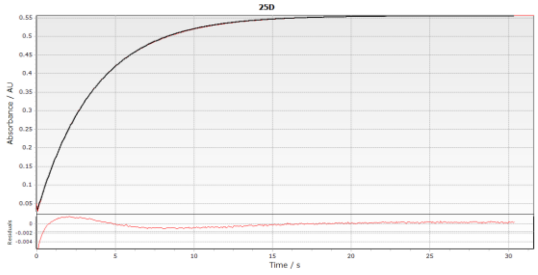

25°C D

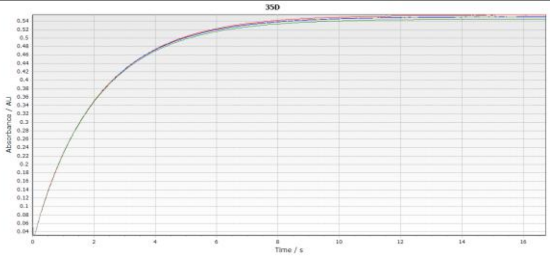

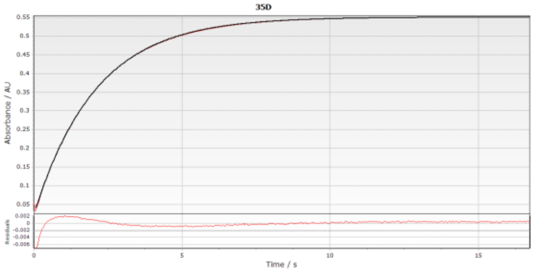

35°C D

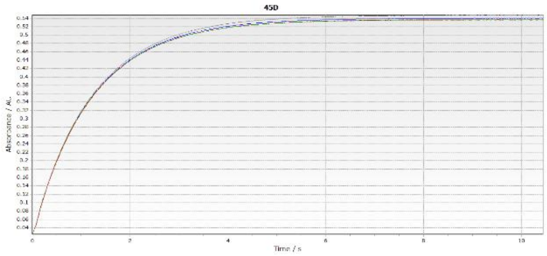

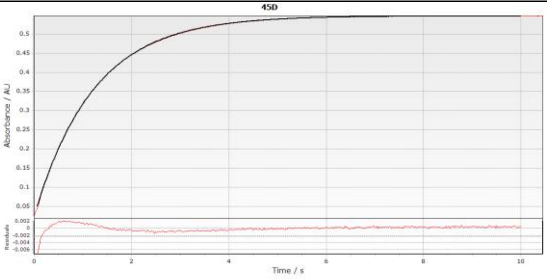

45°C D

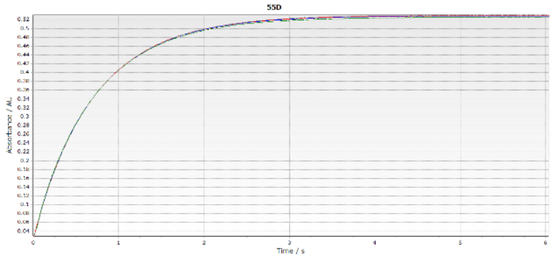

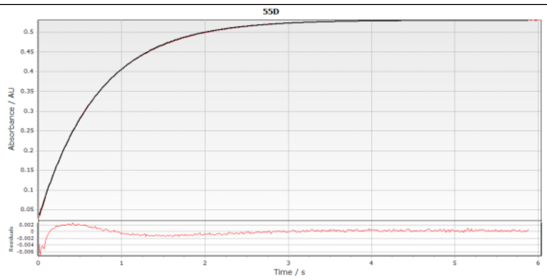

55°C D

Primary kinetic data for the rate constants in Table S2 (MAH with Tr⁺)

Day 1 data (July 17, 2025)

Temp (°C)	Trial H1	Trial H2	Trial H3	Average k_H^{pfo} (s^{-1})	Stdev	k_{2H} ($M^{-1}s^{-1}$)	Stdev [a]
	Pseudo-first-order rate constants k^{pfo} (s^{-1})						
55	0.48393	0.47802	0.47867	0.48021	0.00324	24.01033	0.16204
45	0.28223	0.27656	0.27951	0.27943	0.00284	13.97167	0.14179
35	0.15574	0.15638	0.15191	0.15468	0.00242	7.73383	0.12086
25	0.08078	0.08028	0.08117	0.08074	0.00045	4.03717	0.02231
15	0.04046	0.04103	0.04060	0.04070	0.00030	2.03483	0.01485
**Temp (°C)**	**Trial D1**	**Trial D2**	**Trial D3**	**Average k_D^{pfo} (s^{-1})**	**Stdev**	**k_{2D} ($M^{-1}s^{-1}$)**	**Stdev [a]**
55	0.11578	0.11711	0.11548	0.11612	0.00087	5.80617	0.04338
45	0.06477	0.06509	0.06541	0.06509	0.00032	3.25450	0.01600
35	0.03328	0.03311	0.03344	0.03328	0.00017	1.66383	0.00825
25	0.01664	0.01623	0.01651	0.01646	0.00021	0.82300	0.01048
15	0.00770	0.00769	0.00763	0.00767	0.00038	0.38367	0.00189

[a] = (Stdev(for k^{pfo})/k^{pfo})*k_2

Three kinetic runs together with the averaged data	First-order kinetic fit of the averaged data as an example

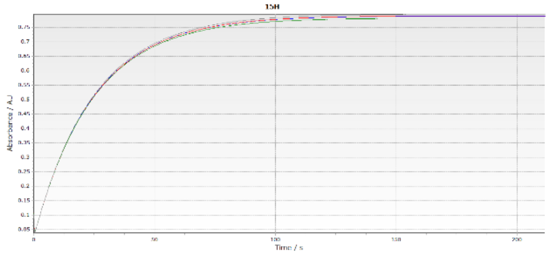

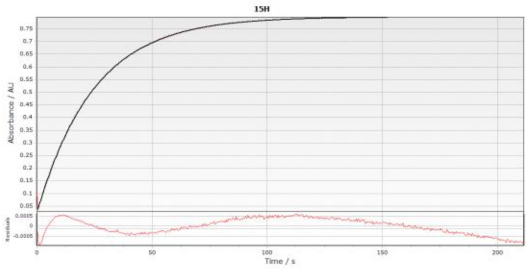

15°C H

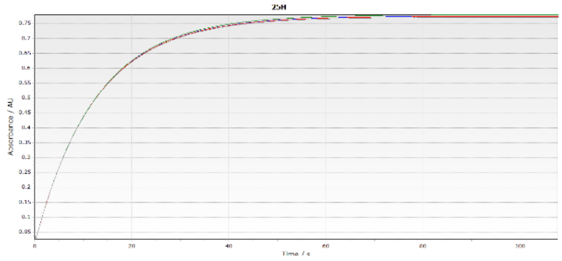

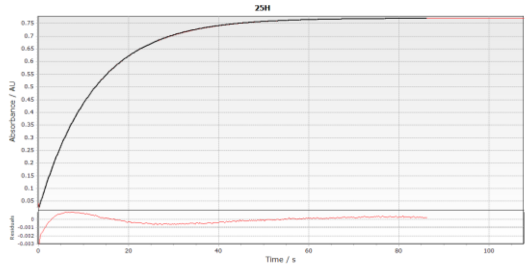

25°C H

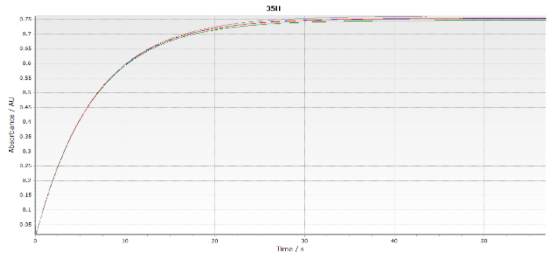

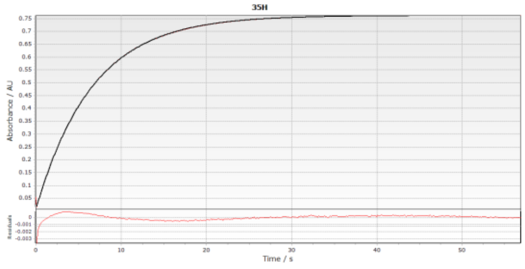

35°C H

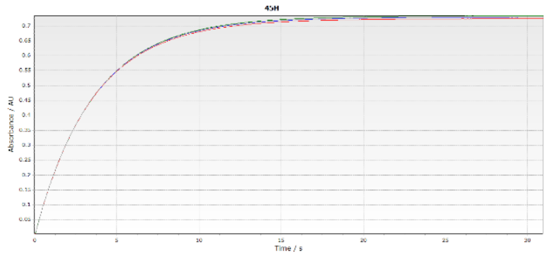

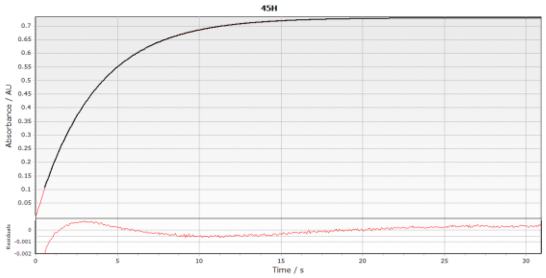

45°C H

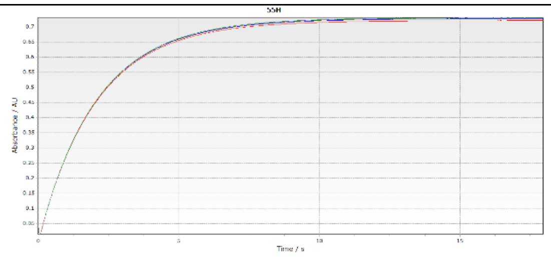

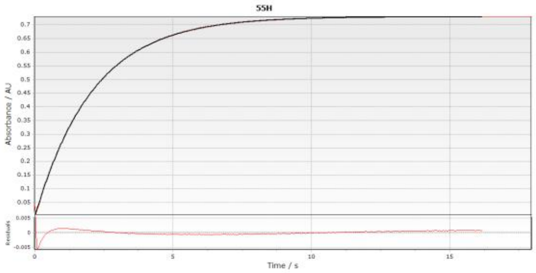

55°C H

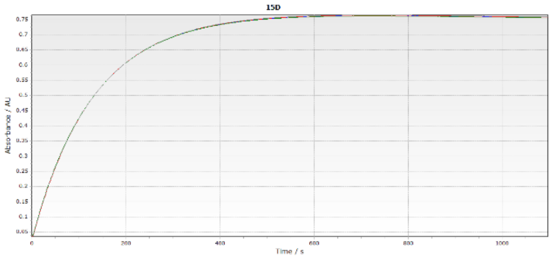

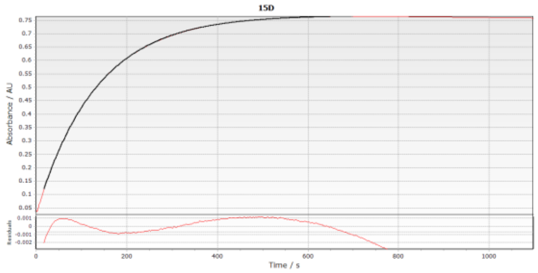

15°C D

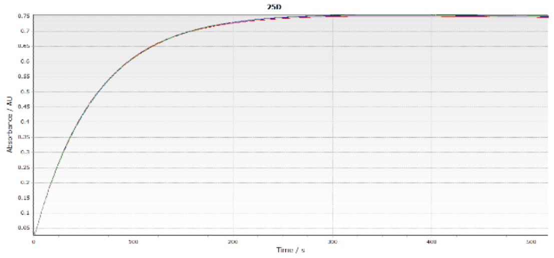

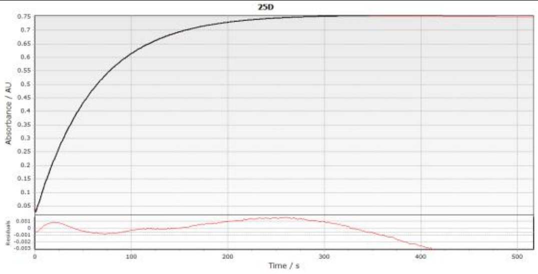

25°C D

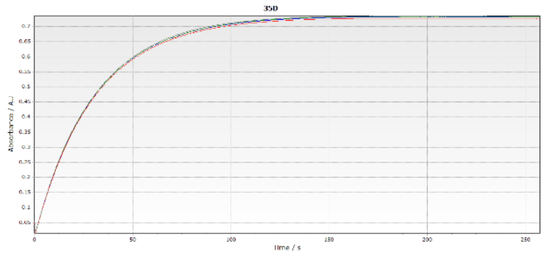

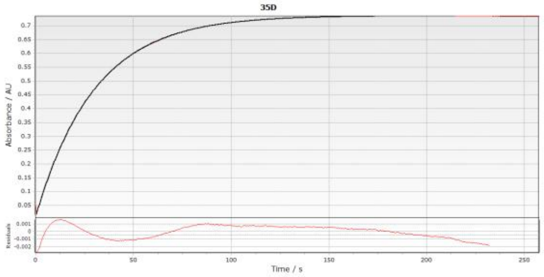

35°C D

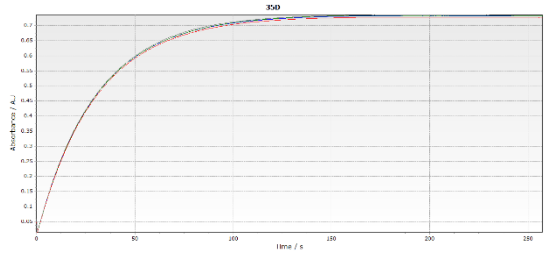

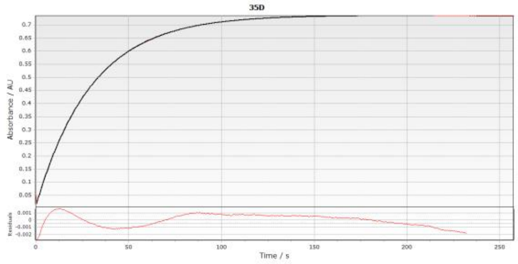

45°C D

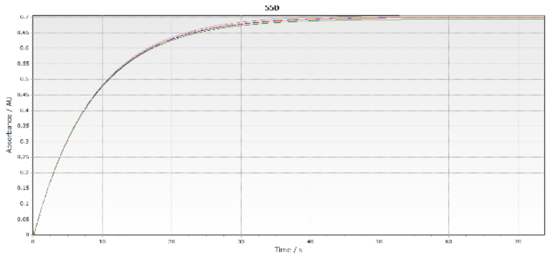

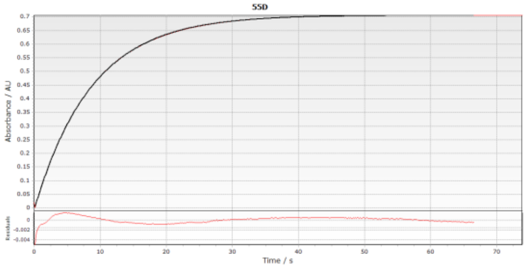

55°C D

Day 2 data (July 22, 2025)

Pseudo-first-order rate constants (k^{pfo} (s^{-1}))

Temp (°C)	Trial H1	Trial H2	Trial H3	Average k_H^{pfo} (s^{-1})	Stdev	k_{2H} (M^{-1}s^{-1})	Stdev [a]
55	0.48010	0.48460	0.48435	0.48302	0.00253	24.15083	0.12645
45	0.27404	0.27473	0.27265	0.27381	0.00106	13.69033	0.05297
35	0.14850	0.14969	0.14956	0.14925	0.00065	7.46250	0.03264
25	0.07973	0.08008	0.07962	0.07981	0.00024	3.99050	0.01201
15	0.04058	0.04063	0.04030	0.04050	0.00018	2.02517	0.00889

Temp (°C)	Trial D1	Trial D2	Trial D3	Average k_D^{pfo} (s^{-1})	Stdev	k_{2D} (M^{-1}s^{-1})	Stdev [a]
55	0.11439	0.11504	0.1151	0.11484	0.00039	5.74217	0.01969
45	0.05978	0.06004	0.06200	0.06061	0.00121	3.03033	0.06068
35	0.03208	0.03163	0.03177	0.03183	0.00023	1.59133	0.01151
25	0.01613	0.01625	0.01639	0.01626	0.00013	0.81283	0.00651
15	0.00759	0.00761	0.00755	0.00758	0.00031	0.37916	0.00153

[a] = (Stdev(for k^{pfo})/k^{pfo})*k_2

Three kinetic runs together with the averaged data	First-order kinetic fit of the averaged data as an example

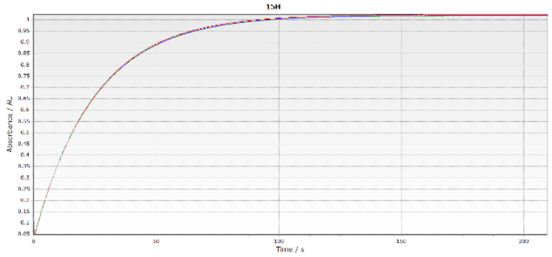

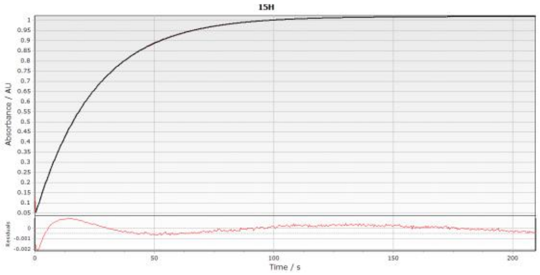

15°C H

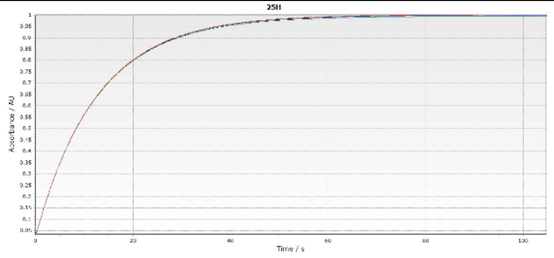

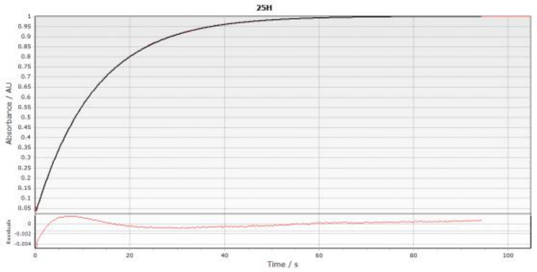

25°C H

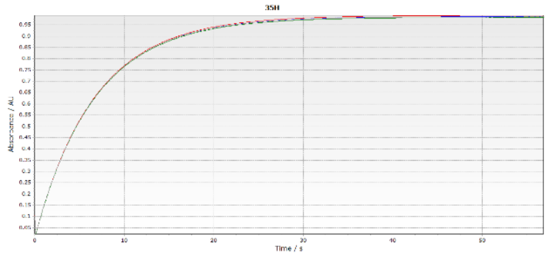

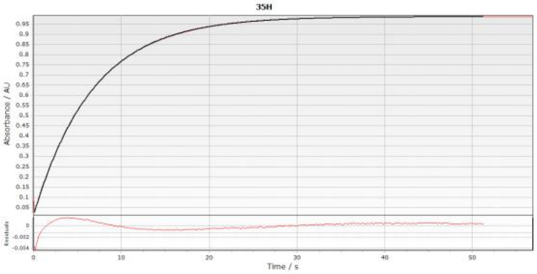

35°C H

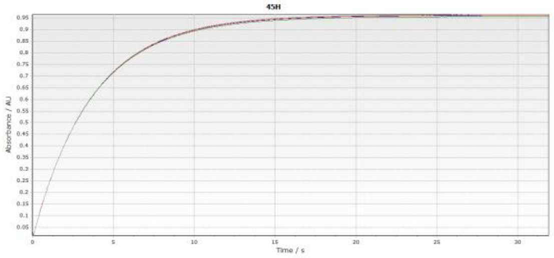

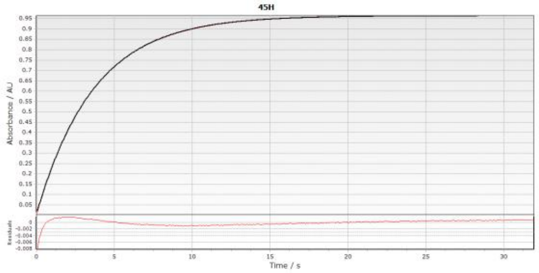

45°C H

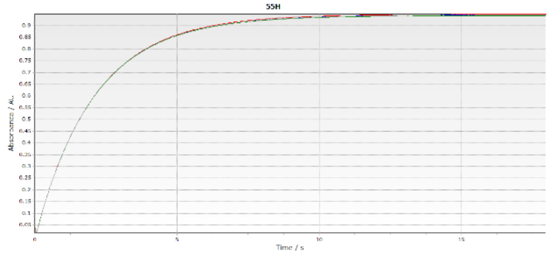

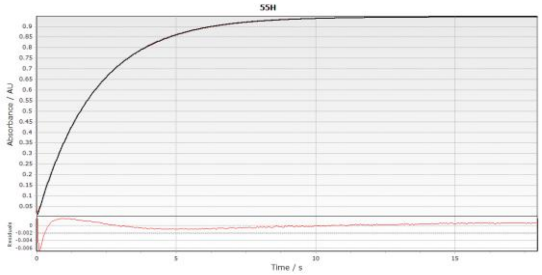

55°C H

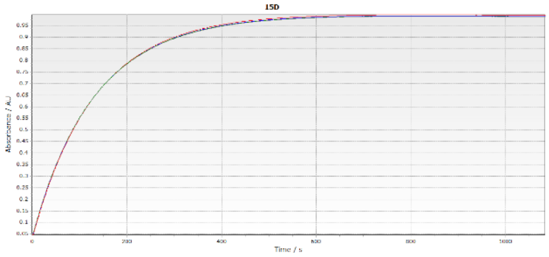

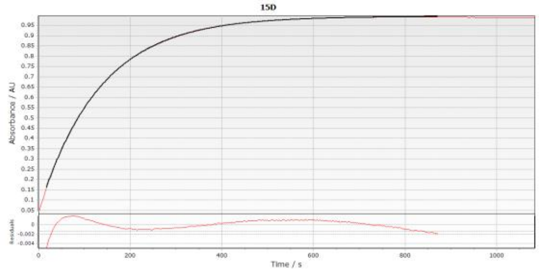

15°C D

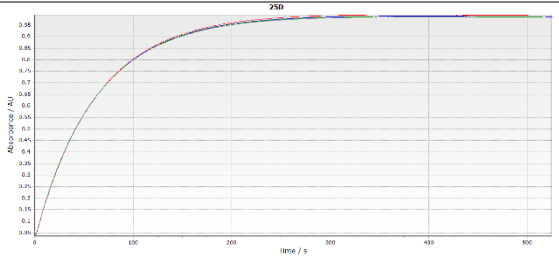

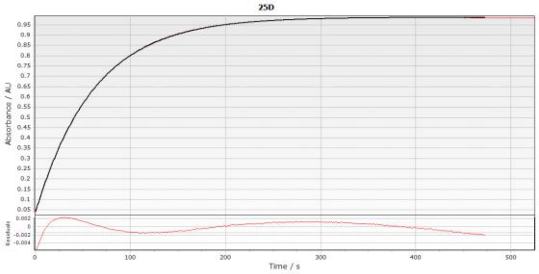

25°C D

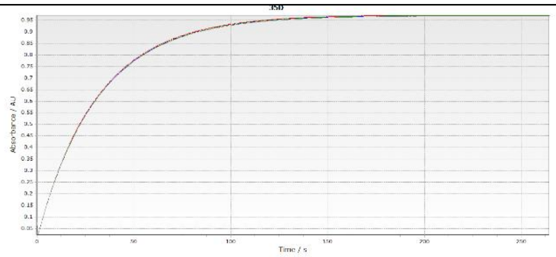

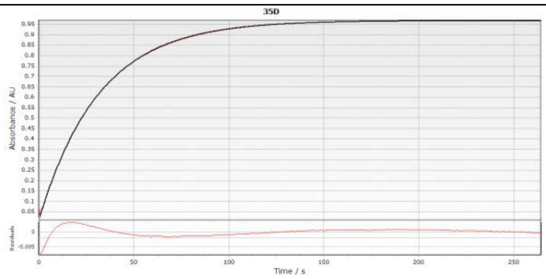

35°C D

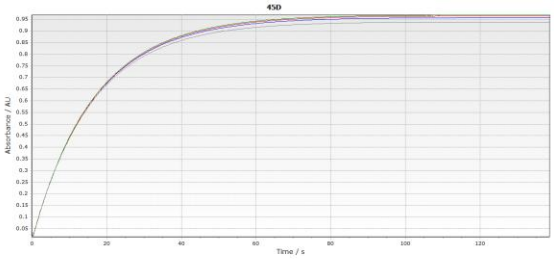

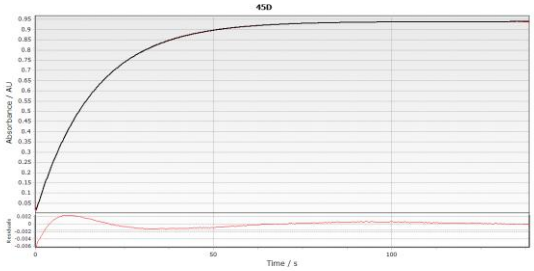

45°C D

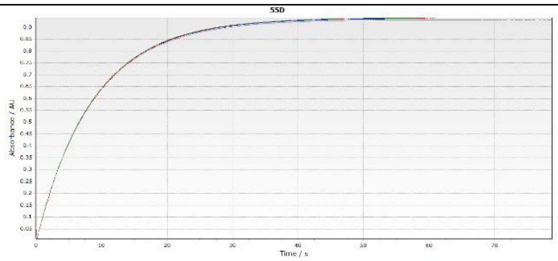

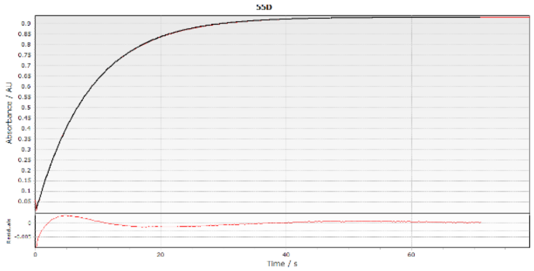

55°C D

Day 3 data (July 24, 2025)

Pseudo-first-order rate constants (k^{pfo} (s^{-1}))

Temp (°C)	Trial H1	Trial H2	Trial H3	Average k_H^{pfo} (s^{-1})	Stdev	k_{2H} (M^{-1}s^{-1})	Stdev [a]
55	4.80E-01	4.80E-01	4.83E-01	4.81E-01	1.48E-03	2.41E+01	0.07418
45	2.74E-01	2.76E-01	2.73E-01	2.74E-01	1.70E-03	1.37E+01	0.08493
35	1.50E-01	1.50E-01	1.50E-01	1.50E-01	3.06E-04	7.50E+00	0.01532
25	7.97E-02	7.98E-02	8.07E-02	8.01E-02	5.50E-04	4.00E+00	0.02750
15	4.10E-02	4.08E-02	4.08E-02	4.09E-02	1.30E-04	2.04E+00	0.00650

Temp (°C)	Trial D1	Trial D2	Trial D3	Average k_D^{pfo} (s^{-1})	Stdev	k_{2D} (M^{-1}s^{-1})	Stdev [a]
55	1.13E-01	1.13E-01	1.14E-01	0.1133	0.000324	5.66E+00	0.01621
45	6.24E-02	6.24E-02	6.17E-02	0.0622	0.000395	3.11E+00	0.01973
35	3.16E-02	3.18E-02	3.17E-02	0.0317	8.02E-05	1.58E+00	0.00401
25	1.65E-02	1.58E-02	1.54E-02	0.0159	0.000579	7.96E-01	0.02897
15	7.60E-03	7.52E-03	7.52E-03	0.0075	4.62E-05	3.77E-01	0.00231

[a] = (Stdev(for k^{pfo})/k^{pfo})*k_2

Three kinetic runs together with the averaged data	First-order kinetic fit of the averaged data as an example

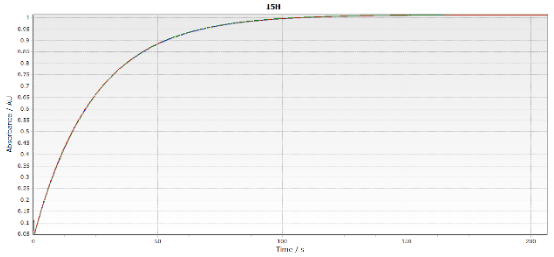

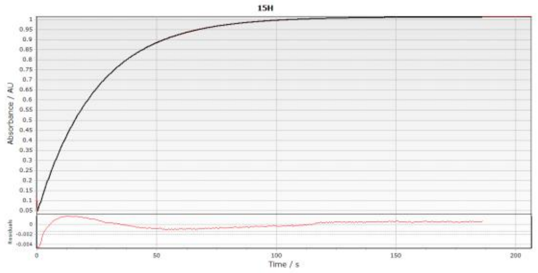

15°C H

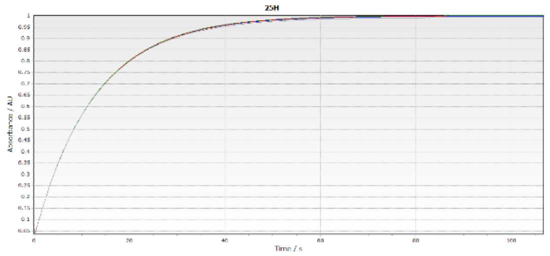

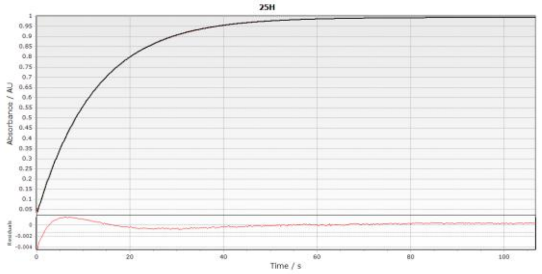

25°C H

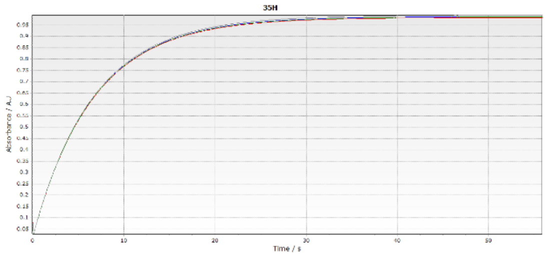

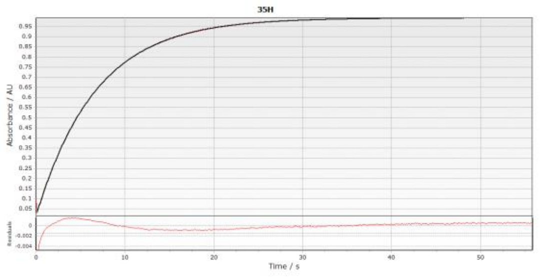

35°C H

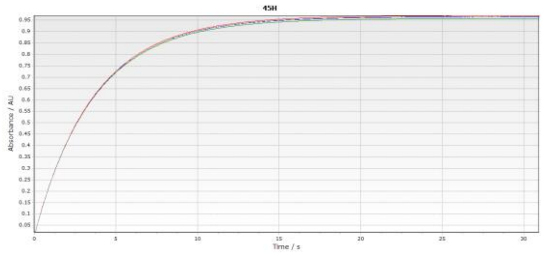

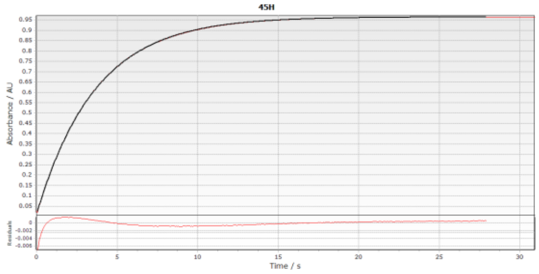

45°C H

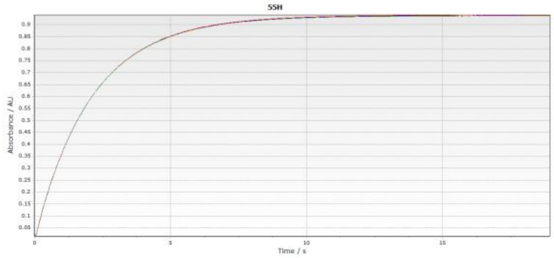

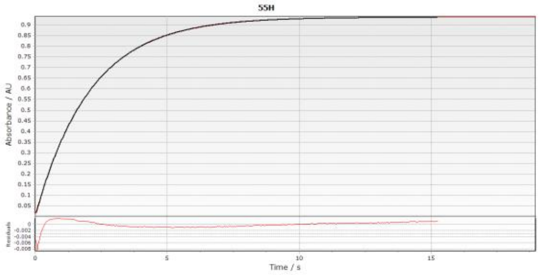

55°C H

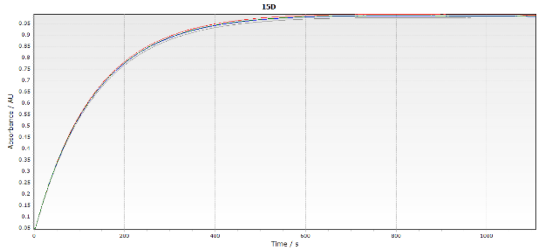

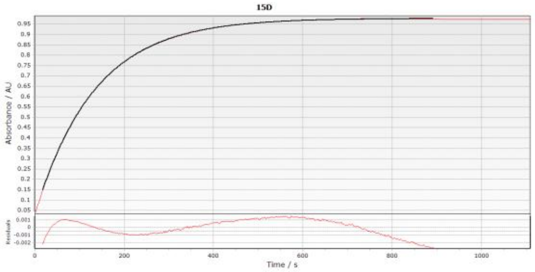

15°C D

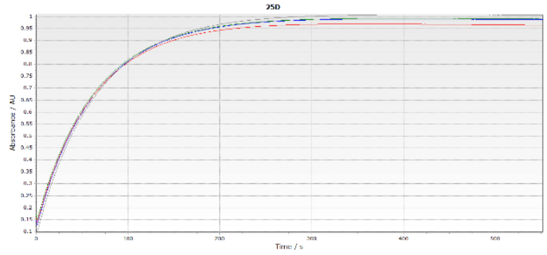

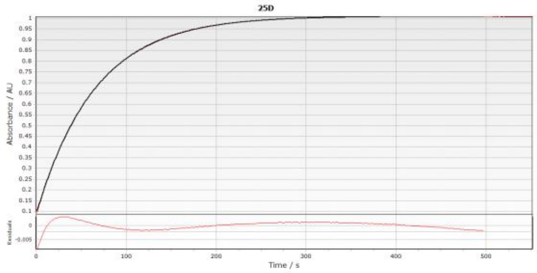

25°C D

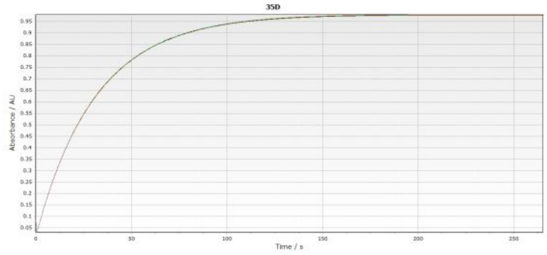

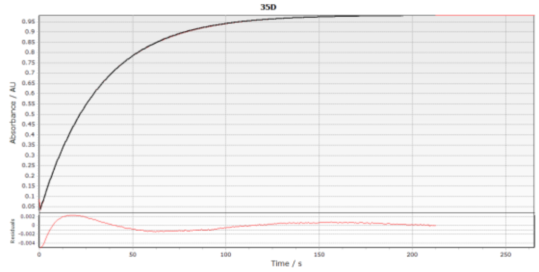

35°C D

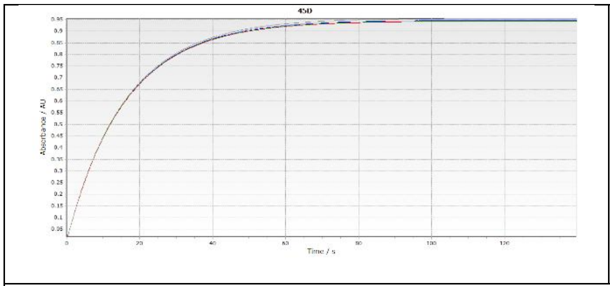

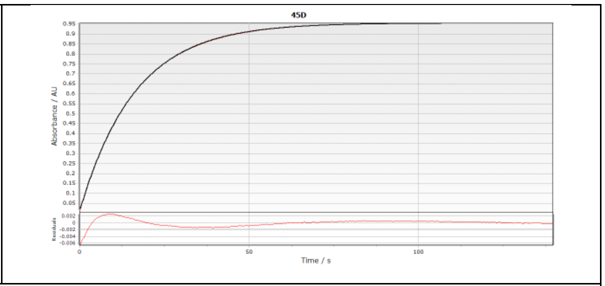

45°C D

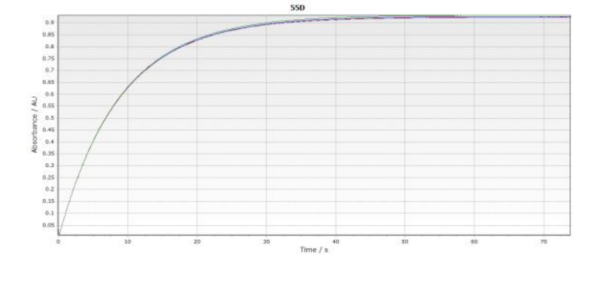

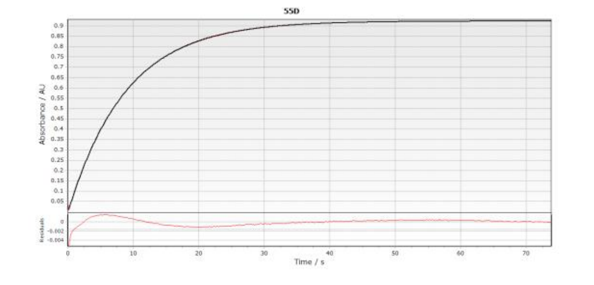

55°C D

Primary kinetic data for the rate constants in Table S3 (HAH with PhXn⁺)

Day 1 data (August 27, 2025)

Pseudo-first-order rate constants

Temp (°C)	k^{pfo} (s^{-1}) Trial H1	Trial H2	Trial H3	Trial H4	Trial H5	Trial H6	Average k_H^{pfo} (s^{-1})	Stdev	k_{2H} (M^{-1}s^{-1})	Stdev [a]
45	4.69904	4.63165	4.71895	4.68395	4.68718	4.67372	4.6824	0.029288	2.34E+03	14.64402
35	3.19837	3.23605	3.25778	3.23817	3.23287	3.24954	3.2355	0.020428	1.62E+03	10.21395
25	2.24451	2.24196	2.23300	2.26753	2.25721	2.24163	2.2476	0.012488	1.12E+03	6.24382
15	1.48145	1.48893	1.49830	1.49844	1.50659	1.49307	1.4945	0.008712	7.47E+02	4.35601
5	0.96800	0.97215	0.97173	0.97655	0.97153	0.97098	0.9718	0.002752	4.86E+02	1.37608
**Temp (°C)**	**Trial D1**	**Trial D2**	**Trial D3**	**Trial D4**	**Trial D5**	**Trial D6**	**Average k_D^{pfo} (s^{-1})**	**Stdev**	**k_{2D} (M^{-1}s^{-1})**	**Stdev [a]**
45	1.21932	1.22052	1.20892	1.20785	1.21386	1.20957	1.2133	0.005504	6.07E+02	2.75202
35	0.81733	0.81543	0.82798	0.81632	0.81273	0.80737	0.8162	0.006792	4.08E+02	3.39604
25	0.53945	0.54439	0.54426	0.54445	0.54110	0.53982	0.5422	0.002389	2.71E+02	1.19430
15	0.34278	0.34187	0.34395	0.34235	0.34370	0.34434	0.3432	0.000977	1.72E+02	0.48850
5	0.21077	0.20613	0.20823	0.20901	0.20693	0.20959	0.2084	0.001717	1.04E+02	0.85839

[a] = (Stdev(for k^{pfo})/k^{pfo})*k_2

Three kinetic runs together with the averaged data	First-order kinetic fit of the averaged data as an example

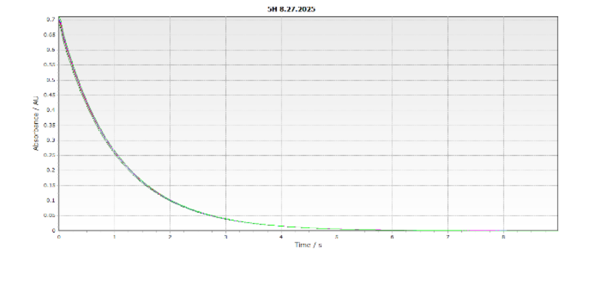

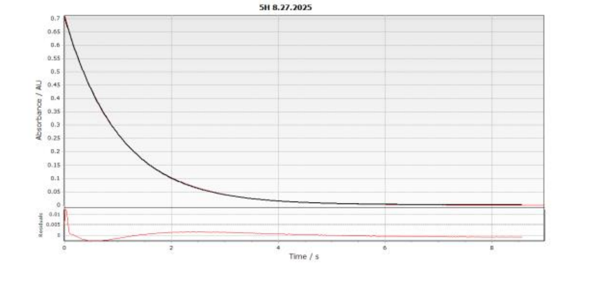

5°C H

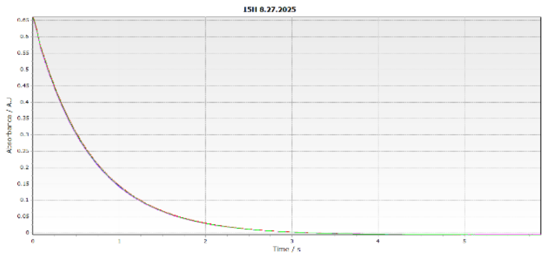

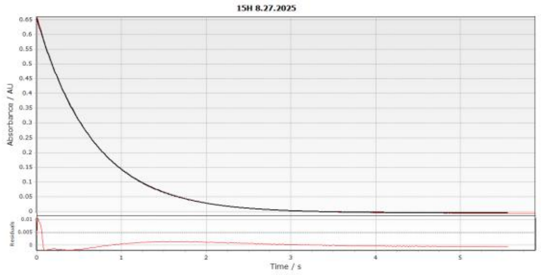

15°C H

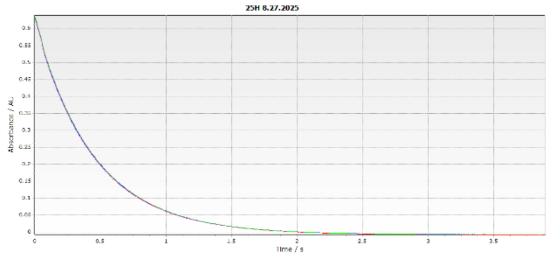

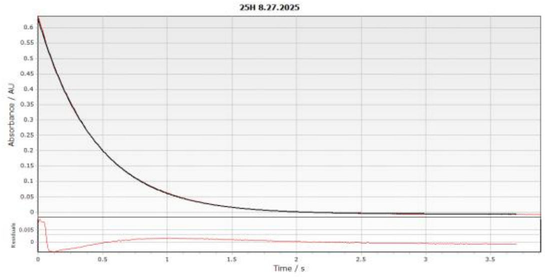

25°C H

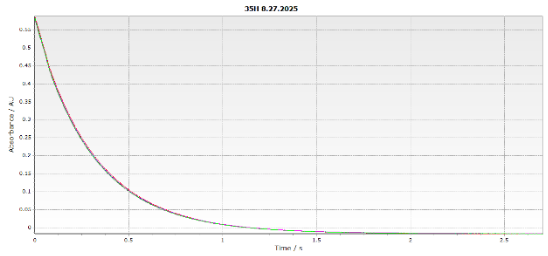

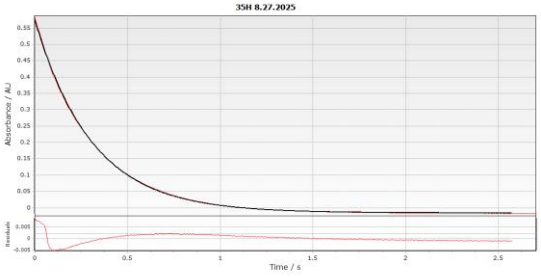

35°C H

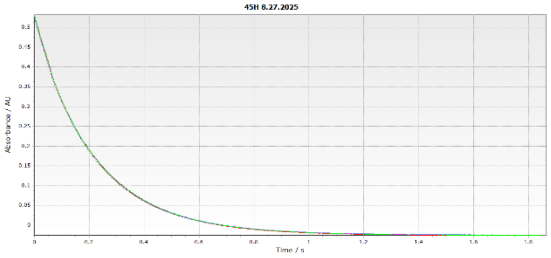

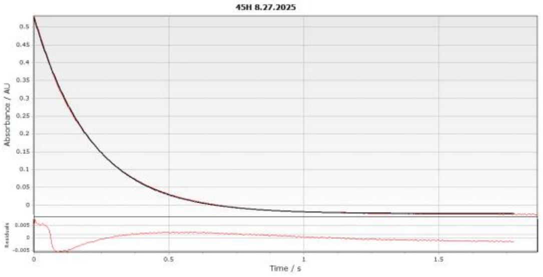

45°C H

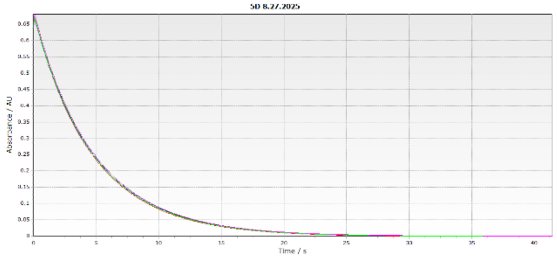

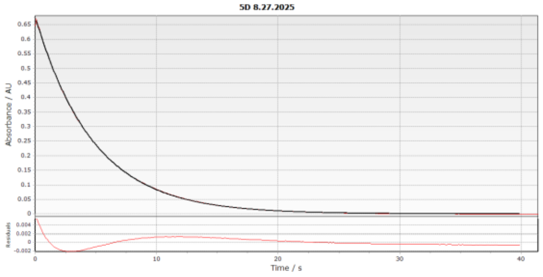

5°C D

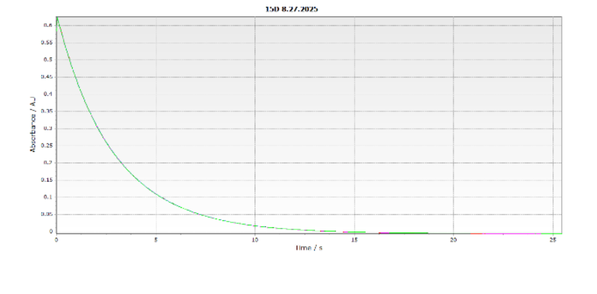

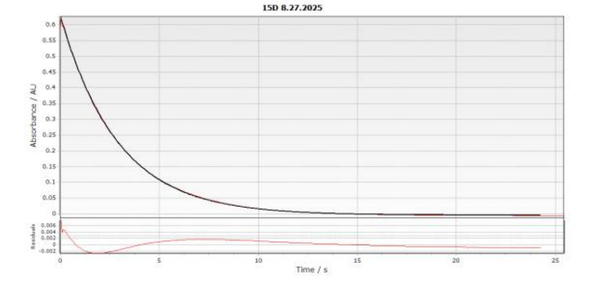

15℃ D

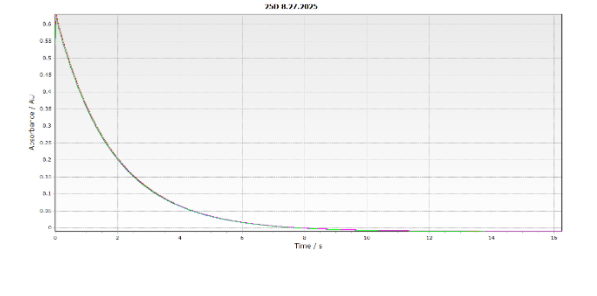

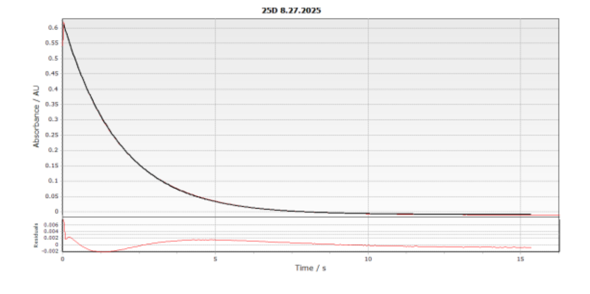

25℃ D

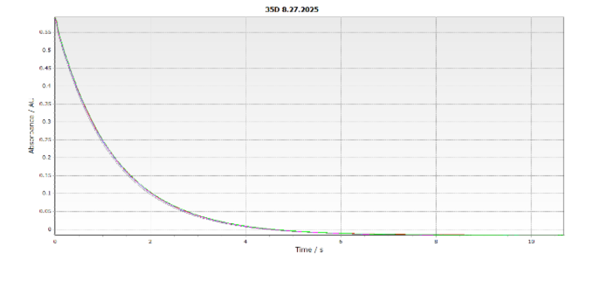

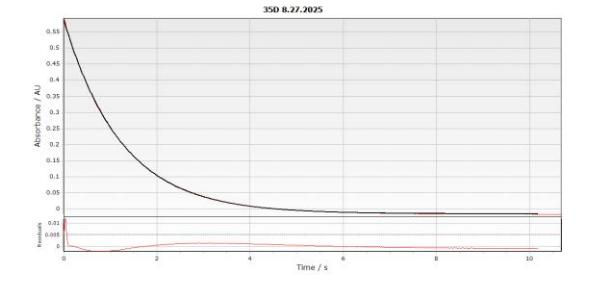

35℃ D

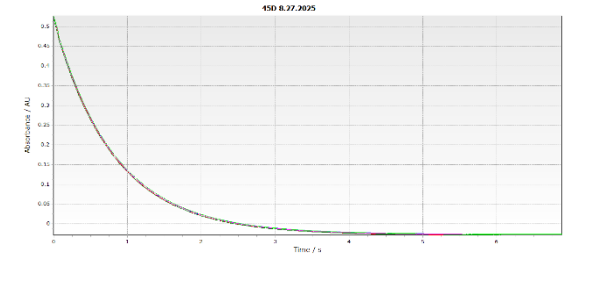

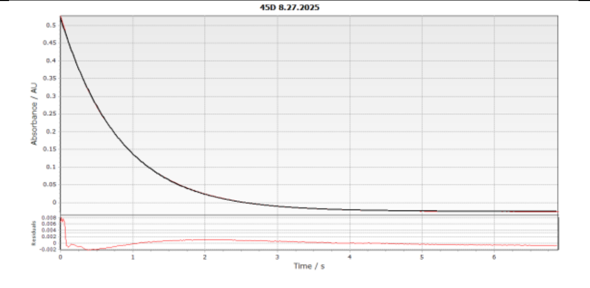

45℃ D

Day 2 data (August 29, 2025)

	Pseudo-first-order rate constants (k^{pfo} (s^{-1}))									
**Temp (°C)**	**Trial H1**	**Trial H2**	**Trial H3**	**Trial H4**	**Trial H5**	**Trial H6**	**Average k_H^{pfo} (s^{-1})**	**Stdev**	**k_{2H} ($M^{-1}s^{-1}$)**	**Stdev [a]**
45	4.65601	4.68586	4.67670	4.79018	4.78067	4.77458	4.7273	0.060659	2.36E+03	30.32947
35	3.29693	3.30257	3.29314	3.27115	3.30516	3.44630	3.3192	0.063422	1.66E+03	31.71117
25	2.27584	2.25802	2.25536	2.26697	2.25239	2.26585	2.2624	0.008745	1.13E+03	4.37245
15	1.50900	1.51551	1.50829	1.50777	1.50819	1.51831	1.5112	0.004544	7.56E+02	2.27221
5	0.97651	0.97454	0.96536	0.98063	0.97607	0.97790	0.9752	0.005225	4.88E+02	2.61250
**Temp (°C)**	**Trial D1**	**Trial D2**	**Trial D3**	**Trial D4**	**Trial D5**	**Trial D6**	**Average k_D^{pfo} (s^{-1})**	**Stdev**	**k_{2D} ($M^{-1}s^{-1}$)**	**Stdev [a]**
45	1.35278	1.36255	1.35521	1.35019	1.35088	1.35167	1.3539	0.004596694	6.77E+02	2.29835
35	0.91092	0.91633	0.91531	0.89836	0.91898	0.9073	0.9112	0.007535136	4.56E+02	3.76757
25	0.59879	0.60510	0.60348	0.59643	0.59856	0.60336	0.6010	0.003471009	3.00E+02	1.73550
15	0.37985	0.37718	0.37611	0.37979	0.37512	0.37966	0.3780	0.002093183	1.89E+02	1.04659
5	0.23241	0.23035	0.22972	0.23034	0.23026	0.23102	0.2307	0.000941375	1.15E+02	0.47069

[a] = (Stdev(for k^{pfo})/k^{pfo})*k_2

Three kinetic runs together with the averaged data	First-order kinetic fit of the averaged data as an example
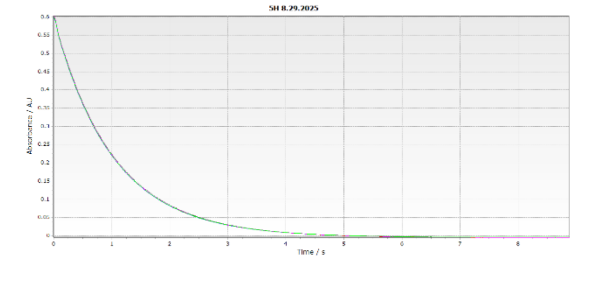	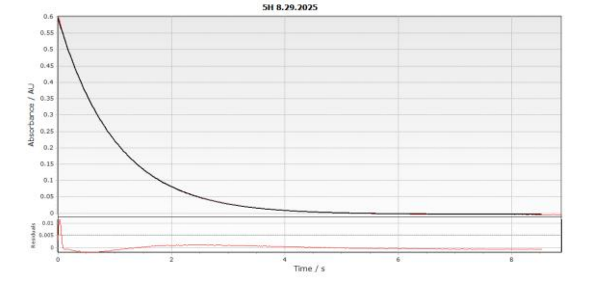
5°C H	
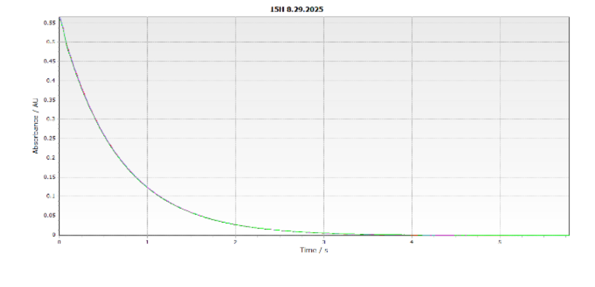	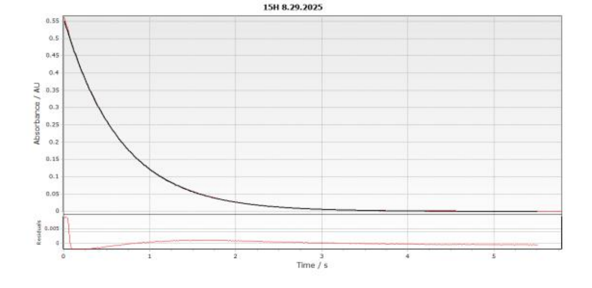
15°C H	
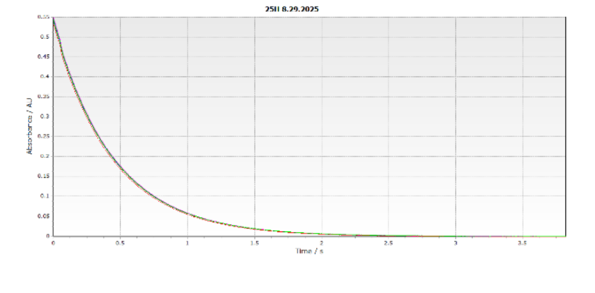	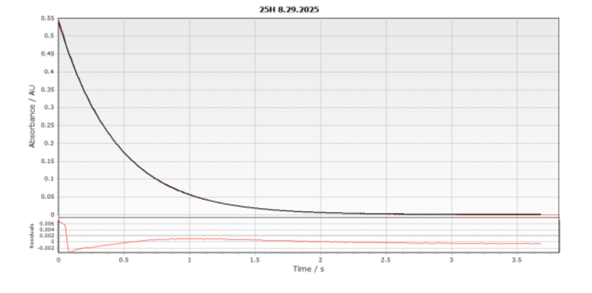
25°C H	

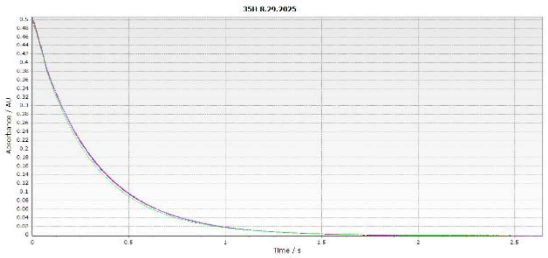	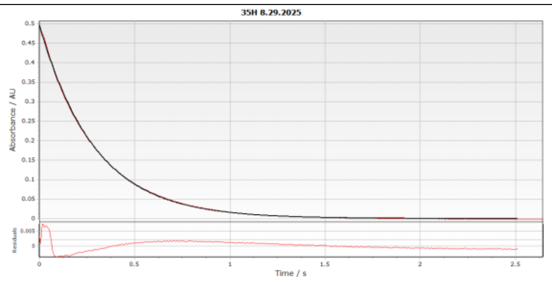
35°C H	
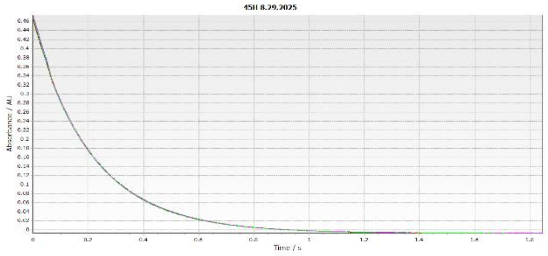	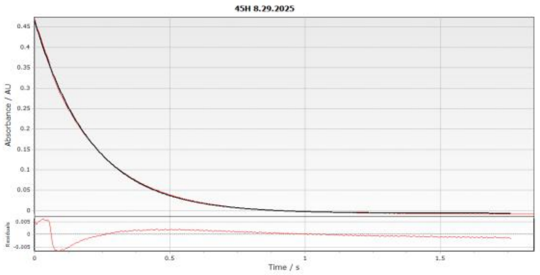
45°C H	
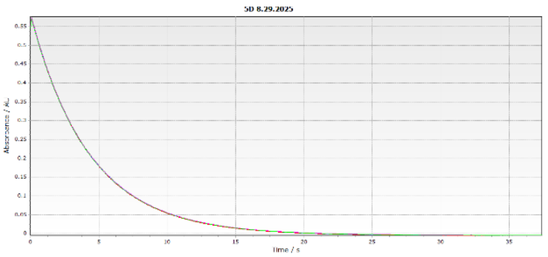	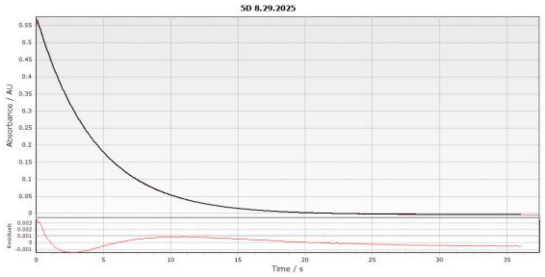
5°C D	
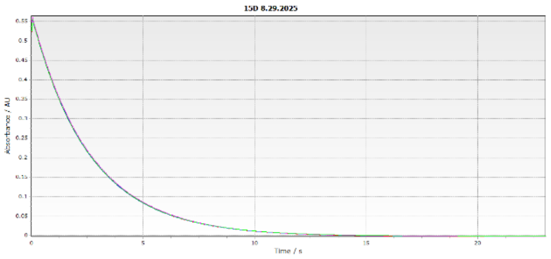	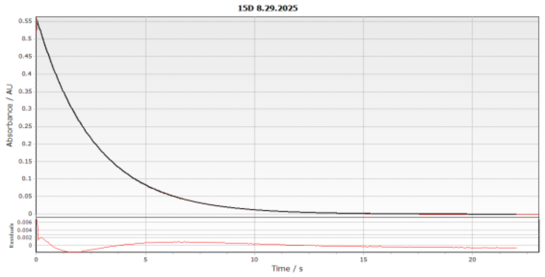
15°C D	
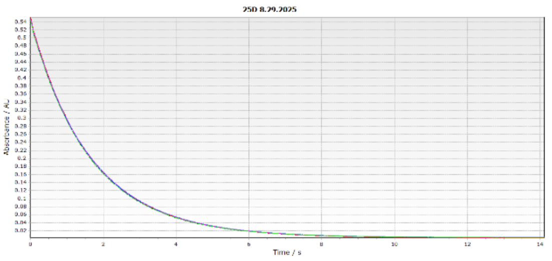	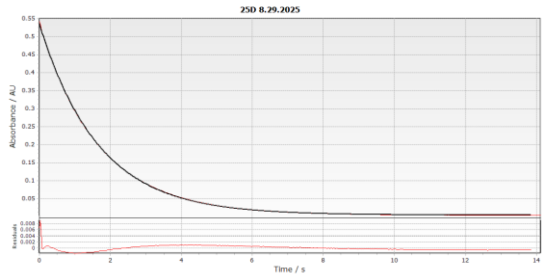
25°C D	

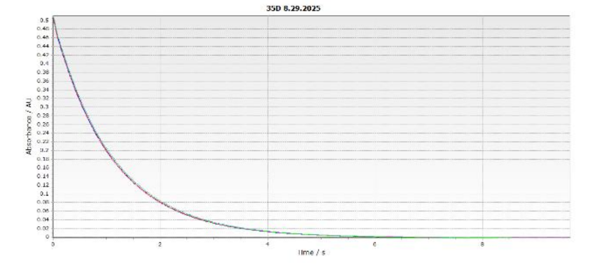

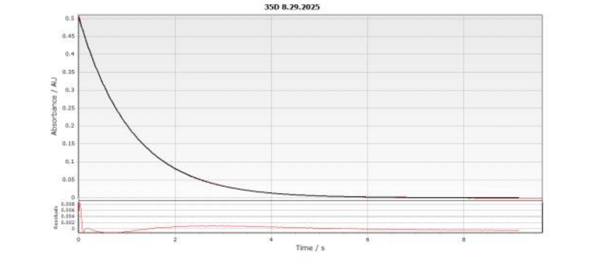

35℃ D

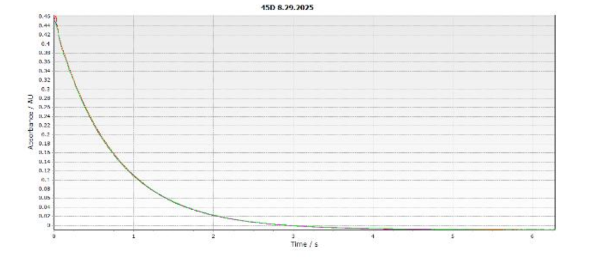

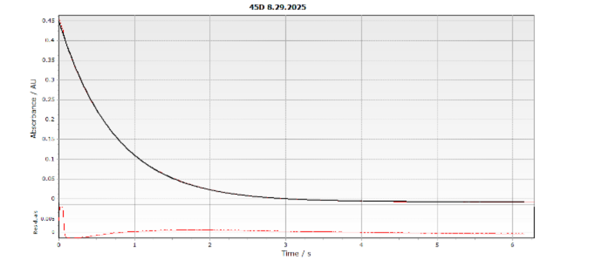

45℃ D

Day 3 data (September 3, 2025)

Temp (°C)	Pseudo-first-order rate constants k^{pfo} (s^{-1}) Trial H1	Trial H2	Trial H3	Trial H4	Trial H5	Trial H6	Average k_H^{pfo} (s^{-1})	Stdev	k_{2H} (M^{-1}s^{-1})	Stdev [a]
45	4.93452	4.97951	4.93160	5.01324	4.98505	5.02878	4.9788	0.03978	2.49E+03	19.88999
35	3.42505	3.46794	3.46264	3.49594	3.50799	3.49748	3.4762	0.03073	1.74E+03	15.36478
25	2.38530	2.38763	2.37355	2.38186	2.38749	2.38960	2.3842	0.005863	1.19E+03	2.93128
15	1.58117	1.58220	1.60416	1.60516	1.60706	1.60874	1.5981	0.012802	7.99E+02	6.40114
5	1.02971	1.02238	1.02638	1.01311	1.02159	1.02309	1.0227	0.005588	5.11E+02	2.79407
**Temp (°C)**	**Trial D1**	**Trial D2**	**Trial D3**	**Trial D4**	**Trial D5**	**Trial D6**	**Average k_D^{pfo} (s^{-1})**	**Stdev**	**k_{2D} (M^{-1}s^{-1})**	**Stdev [a]**
45	1.40166	1.38698	1.39492	1.40328	1.38486	1.40711	1.3965	0.009096994	6.98E+02	4.54850
35	0.95077	0.93717	0.95167	0.94439	0.94726	0.94502	0.9460	0.005253527	4.73E+02	2.62676
25	0.61408	0.62330	0.61784	0.61281	0.61142	0.61913	0.6164	0.004476651	3.08E+02	2.23833
15	0.39100	0.38882	0.38829	0.38952	0.38503	0.38836	0.3885	0.001974707	1.94E+02	0.98735
5	0.23617	0.23581	0.23902	0.23553	0.23521	0.23628	0.2363	0.001373225	1.18E+02	0.68661

[a] = (Stdev(for k^{pfo})/k^{pfo})*k_2

Three kinetic runs together with the averaged data	First-order kinetic fit of the averaged data as an example

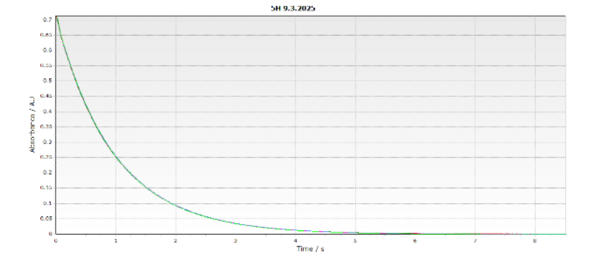

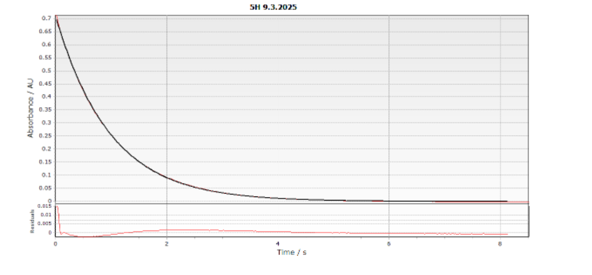

5℃ H

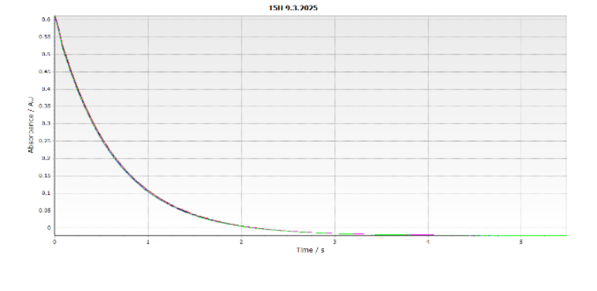

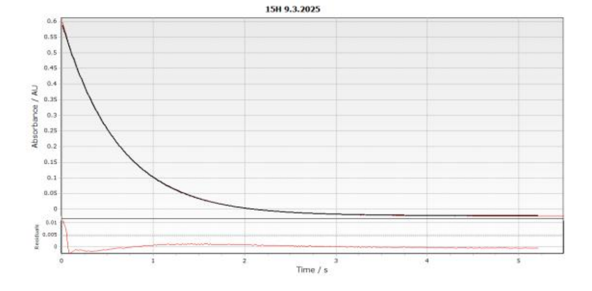

15°C H

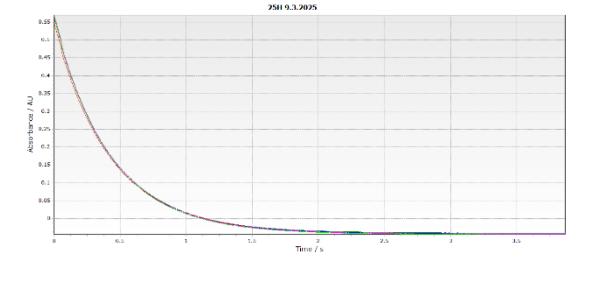

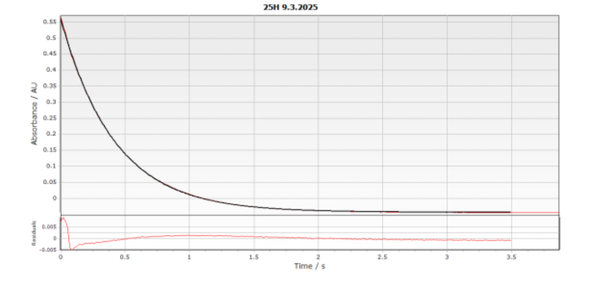

25°C H

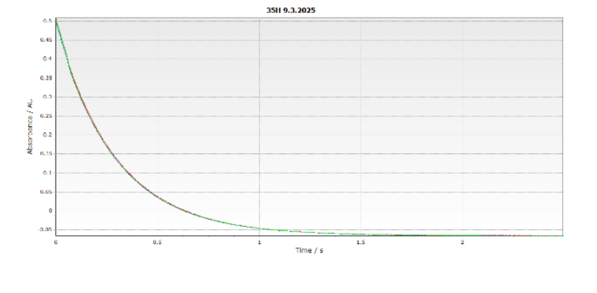

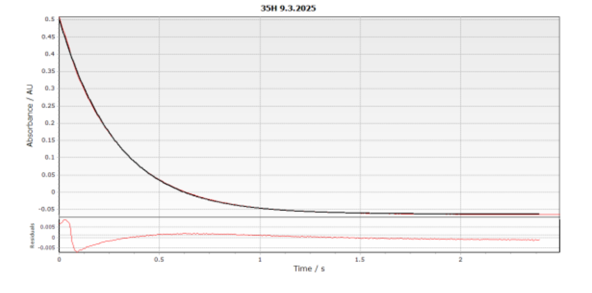

35°C H

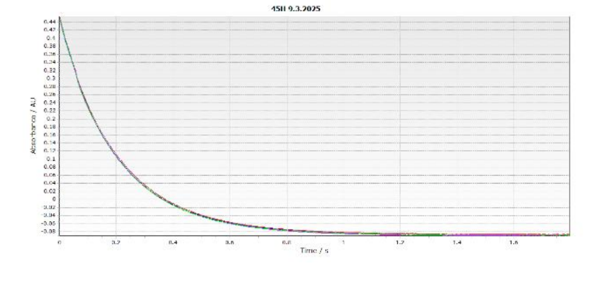

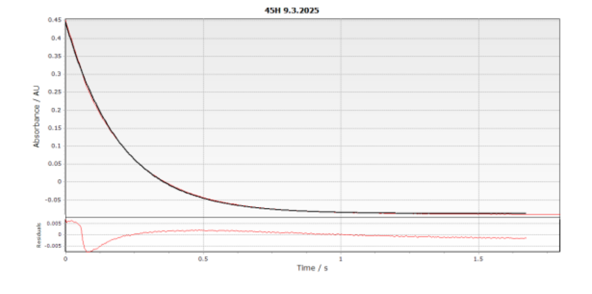

45°C H

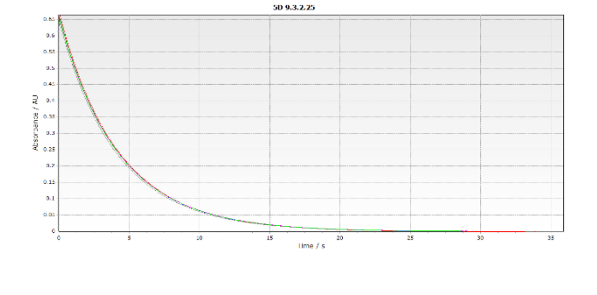

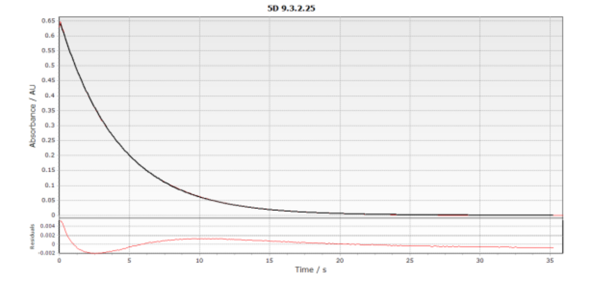

5°C D

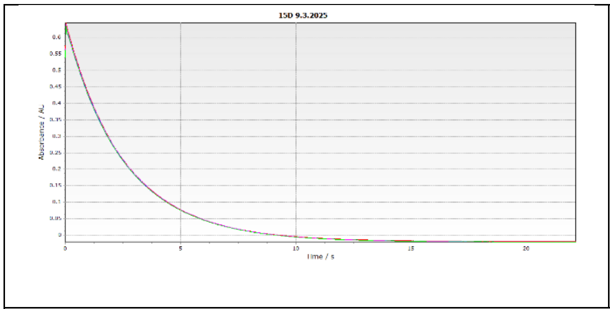

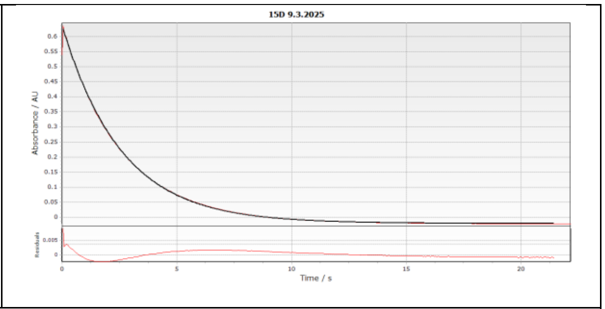

15℃ D

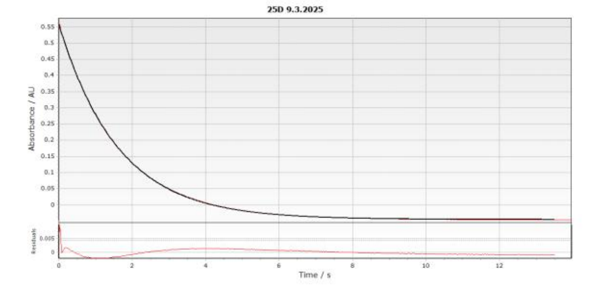

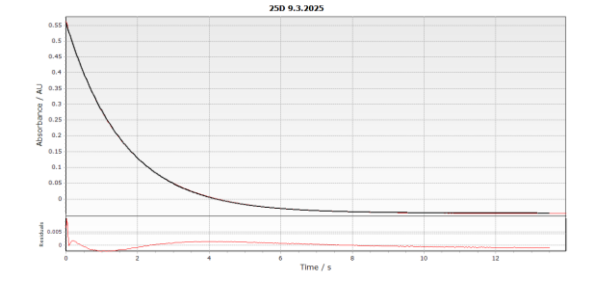

25℃ D

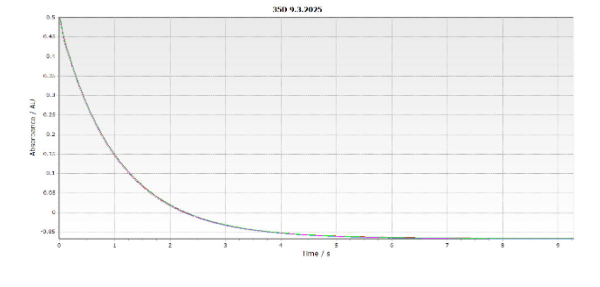

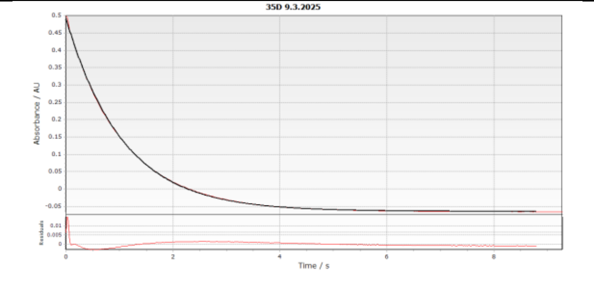

35℃ D

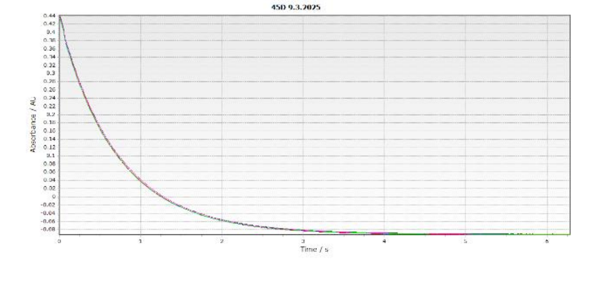

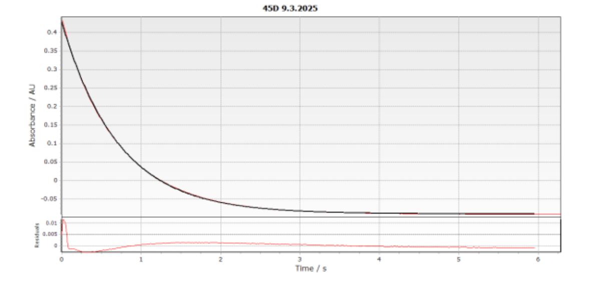

45℃ D

Day 4 data (September 5, 2025)

Temp (°C)	Pseudo-first-order rate constants k^{pfo} (s^{-1}) Trial H1	Trial H2	Trial H3	Trial H4	Trial H5	Trial H6	Average k_H^{pfo} (s^{-1})	Stdev	k_{2H} ($M^{-1}s^{-1}$)	Stdev [a]
45	4.69534	4.78351	4.67474	4.69216	4.71409	4.72277	4.7138	0.038144	2.36E+03	19.07193
35	3.31766	3.33172	3.33172	3.33172	3.33978	3.33942	3.3320	0.008018	1.67E+03	4.00909
25	2.27581	2.30783	2.28581	2.31304	2.29278	2.28585	2.2935	0.014271	1.15E+03	7.13537
15	1.54712	1.52935	1.54347	1.54347	1.53254	1.53475	1.5385	0.007169	7.69E+02	3.58467
5	1.00387	0.98874	0.99482	0.98851	0.99492	0.98936	0.9934	0.005931	4.97E+02	2.96557

Temp (°C)	Trial D1	Trial D2	Trial D3	Trial D4	Trial D5	Trial D6	Average k_D^{pfo} (s^{-1})	Stdev	k_{2D} ($M^{-1}s^{-1}$)	Stdev [a]
45	1.29409	1.2946	1.27815	1.28338	1.26379	1.27545	1.2816	0.011790558	6.41E+02	5.89528
35	0.8637	0.87701	0.86688	0.86773	0.86833	0.87621	0.8700	0.005386411	4.35E+02	2.69321
25	0.56436	0.56963	0.57061	0.56780	0.56923	0.57104	0.5688	0.002444516	2.84E+02	1.22226
15	0.35926	0.36103	0.35842	0.35556	0.35614	0.35671	0.3579	0.002092517	1.79E+02	1.04626
5	0.21664	0.22108	0.22097	0.21673	0.21525	0.21688	0.2179	0.002471961	1.09E+02	1.23598

[a] = (Stdev(for k^{pfo})/k^{pfo})*k_2

Three kinetic runs together with the averaged data	First-order kinetic fit of the averaged data as an example
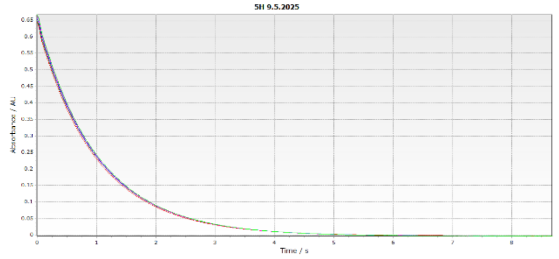	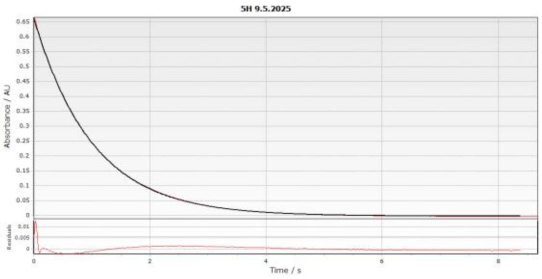
5°C H	
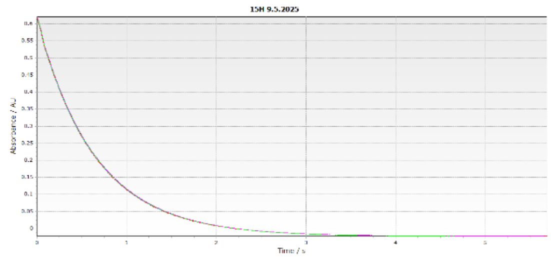	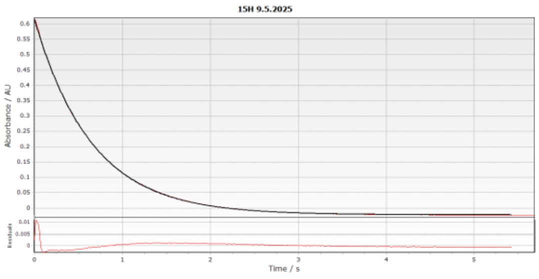
15°C H	
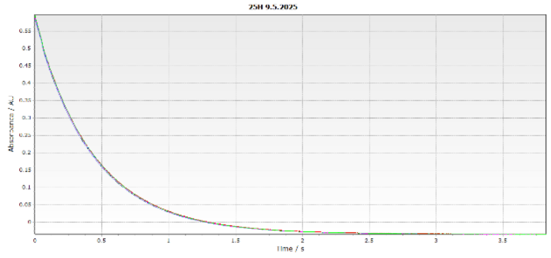	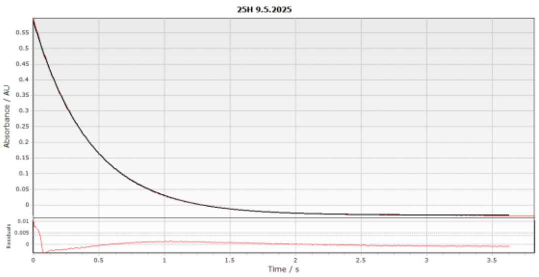
25°C H	

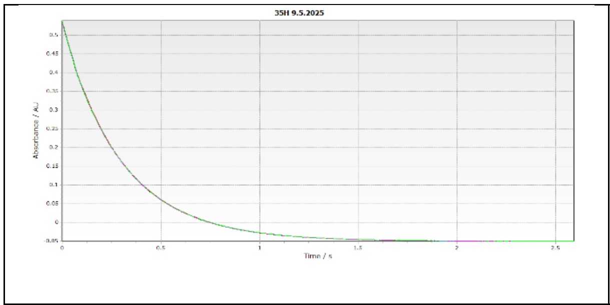

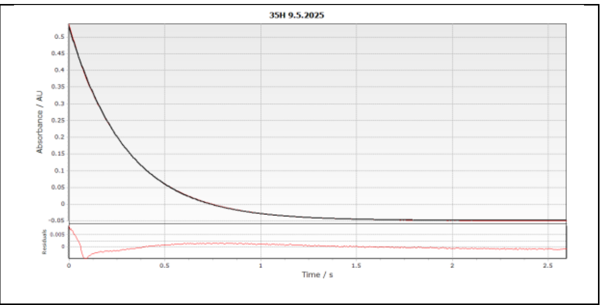

35°C H

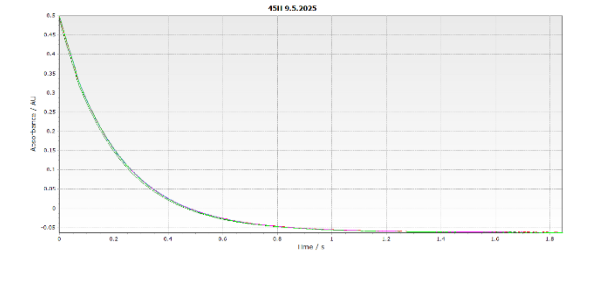

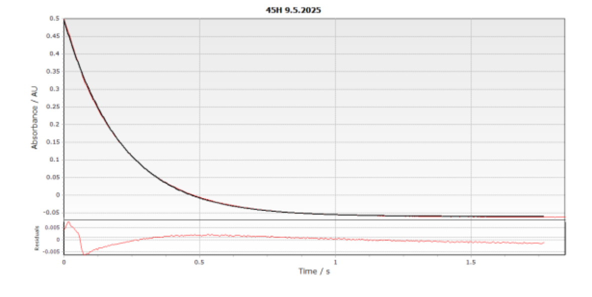

45°C H

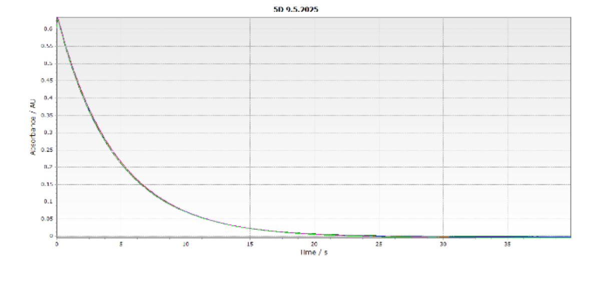

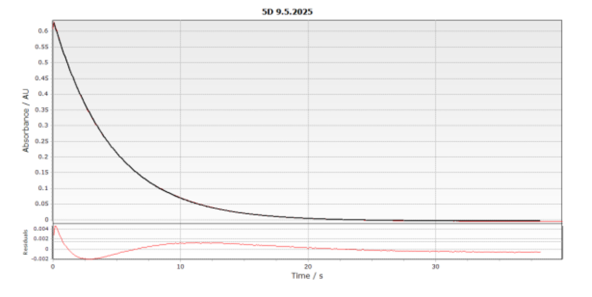

5°C D

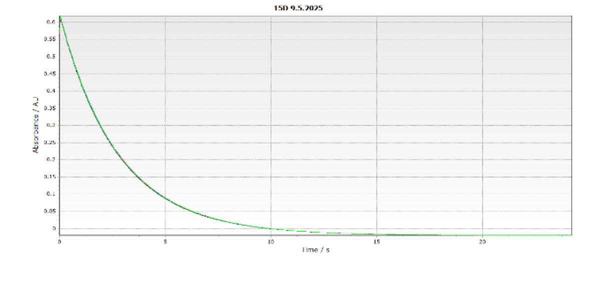

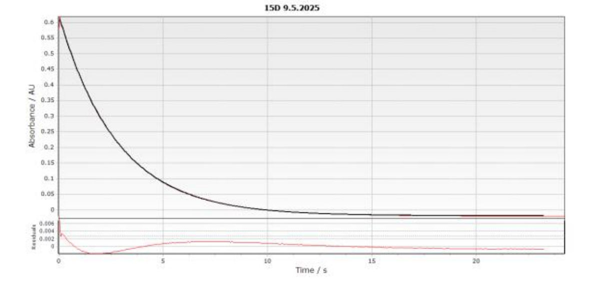

15°C D

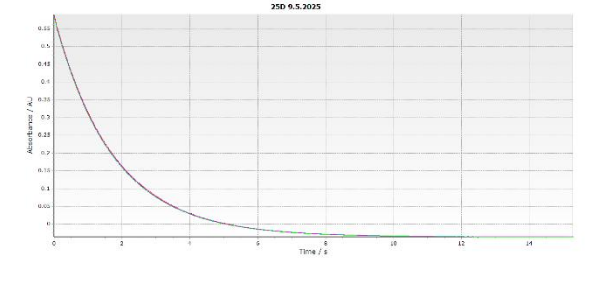

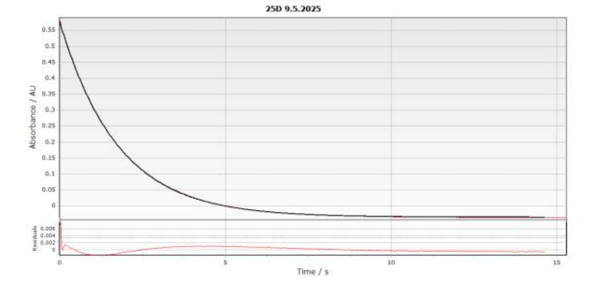

25°C D

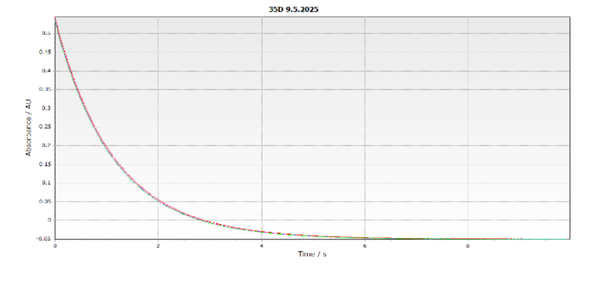

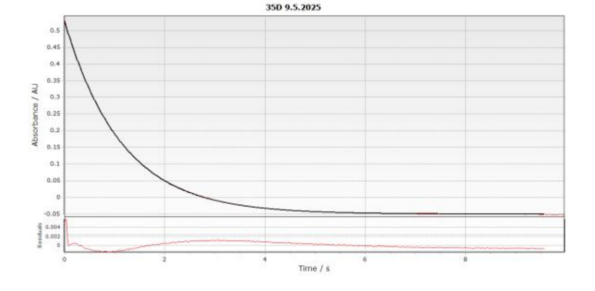

35°C D

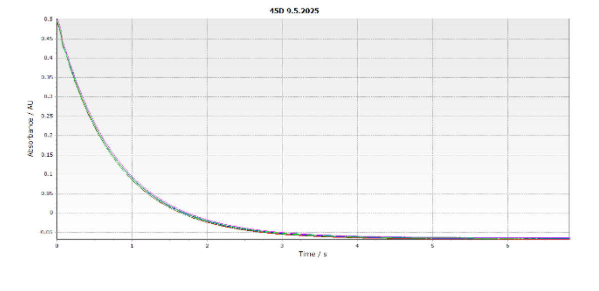

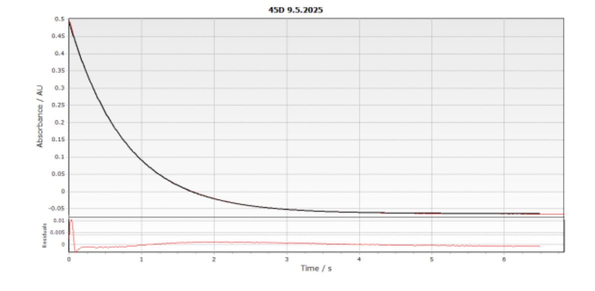

45°C D

Primary kinetic data for the rate constants in Table S4 (MPH with MeOPhXn⁺)

Day 1 data (July 20, 2020)

Pseudo-first-order rate constants

k^{pfo} (s^{-1})

Temp (°C)	Trial H1	Trial H2	Trial H3	Trial H4	Trial H5	Trial H6	Average k_H^{pfo} (s^{-1})	Stdev	k_{2H} ($M^{-1}s^{-1}$)	Stdev [a]
45	2.17445	2.14421	2.14648	2.15851	2.18591	2.13870	2.1580	1.87E-02	3.46E+03	3.00E+01
35	1.47745	1.47526	1.46805	1.46351	1.47010	1.45906	1.4689	6.95E-03	2.36E+03	1.12E+01
25	0.96304	0.97016	0.96957	0.97103	0.97524	0.98159	0.9718	6.21E-03	1.56E+03	9.96E+00
15	0.62864	0.62317	0.62595	0.62515	0.62610	0.62772	0.6261	1.93E-03	1.01E+03	3.09E+00
5	0.39064	0.38972	0.38736	0.38577	0.39009	0.38639	0.3883	2.08E-03	6.23E+02	3.34E+00

Temp (°C)	Trial D1	Trial D2	Trial D3	Trial D4	Trial D5	Trial D6	Average k_D^{pfo} (s-1)	Stdev	k_{2D} (M-1s-1)	Stdev [a]
45	0.71114	0.71539	0.71401	0.70687	0.71872	0.71216	0.7130	4.03E-03	1.15E+03	6.47E+00
35	0.46578	0.46575	0.46327	0.47037	0.47081	0.46963	0.4676	3.08E-03	7.51E+02	4.95E+00
25	0.29750	0.29778	0.29554	0.29829	0.29717	0.29801	0.2974	9.83E-04	4.78E+02	1.58E+00
15	0.18531	0.18541	0.18577	0.18520	0.18711	0.18516	0.1857	7.43E-04	2.98E+02	1.19E+00
5	0.11115	0.11187	0.11043	0.11149	0.11104	0.11103	0.1112	4.85E-04	1.79E+02	7.79E-01

[a] = (Stdev(for k^{pfo})/k^{pfo})*k_2

Six kinetic trials and the mean fit	First-order kinetic fitted average of six trials

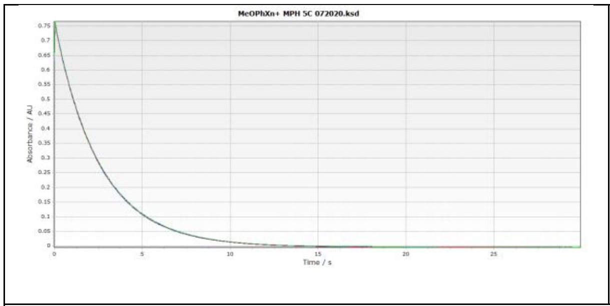

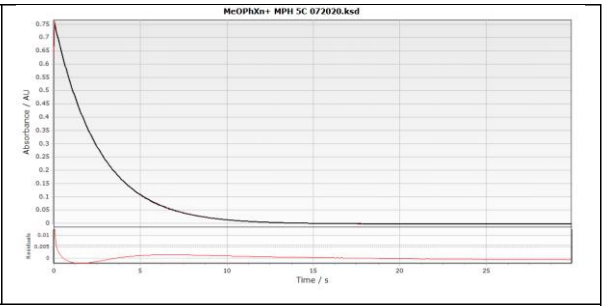

5°C H

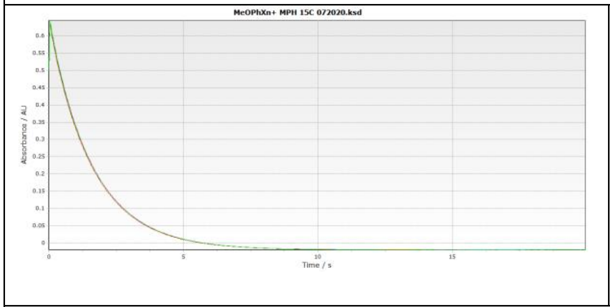

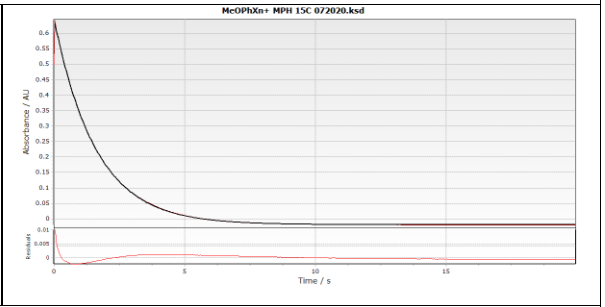

15°C H

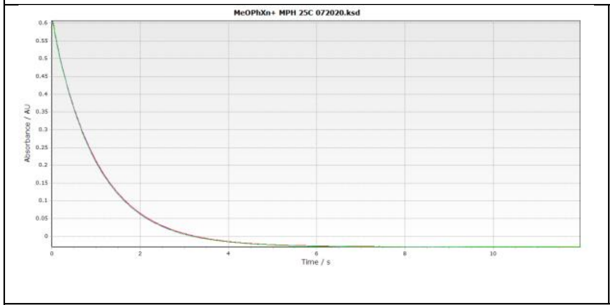

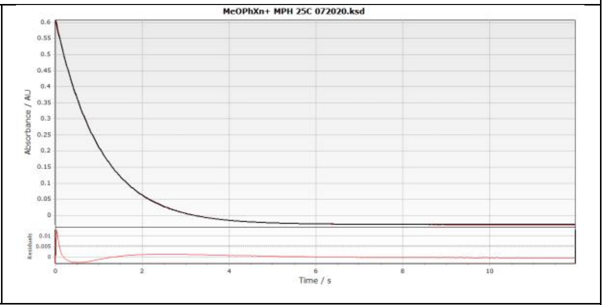

25°C H

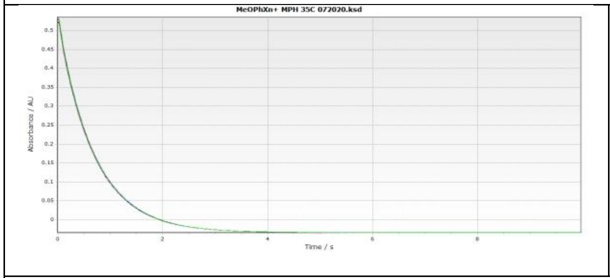

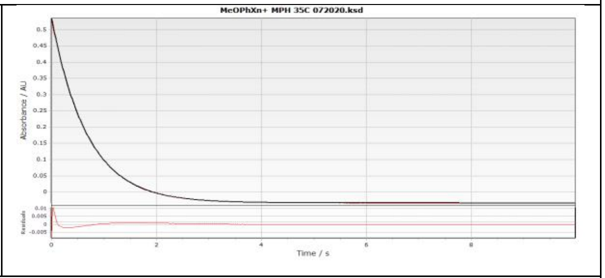

35°C H

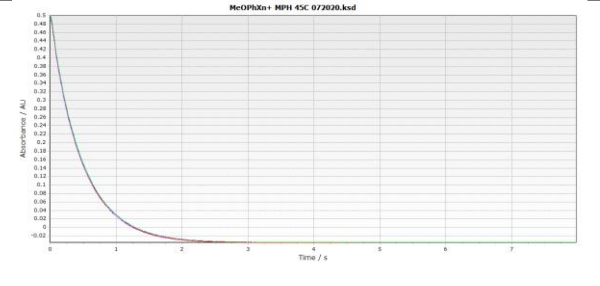

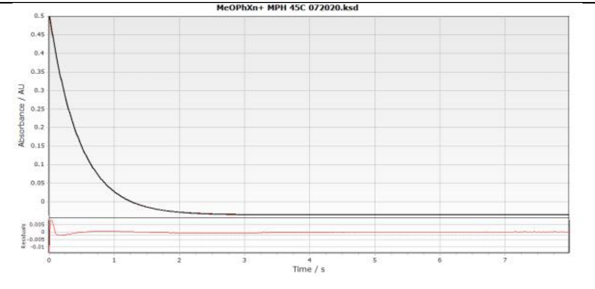

45°C H

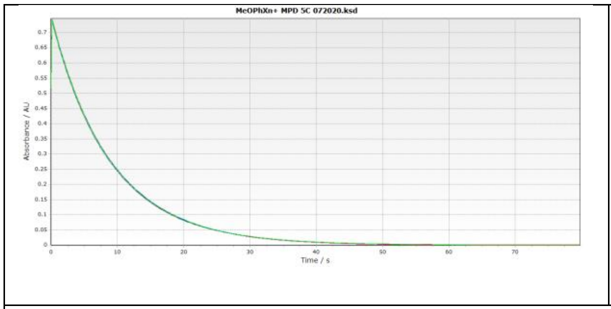

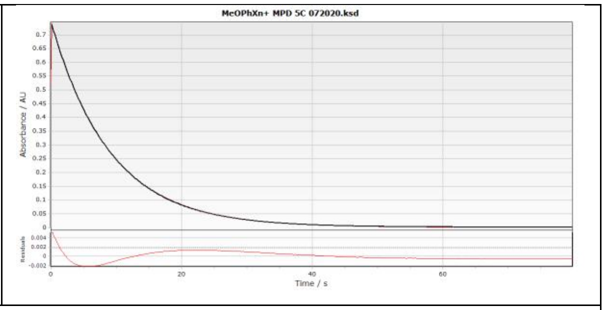

5°C D

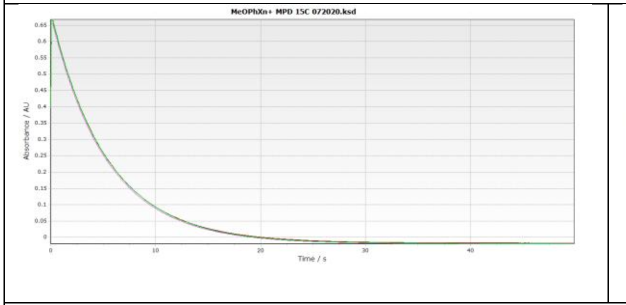

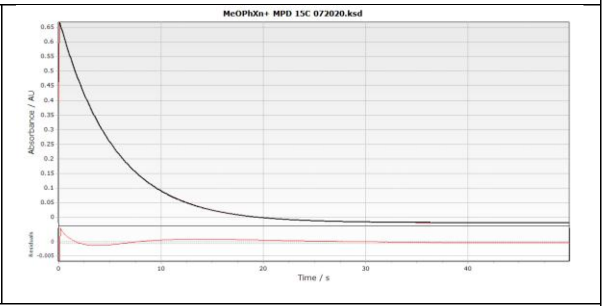

15°C D

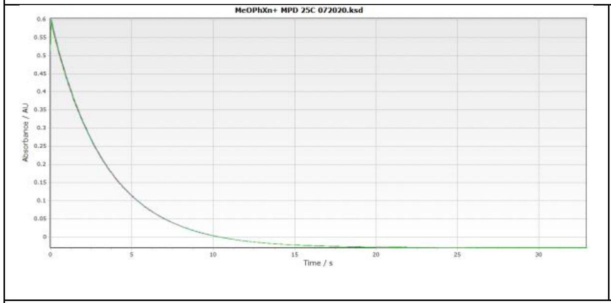

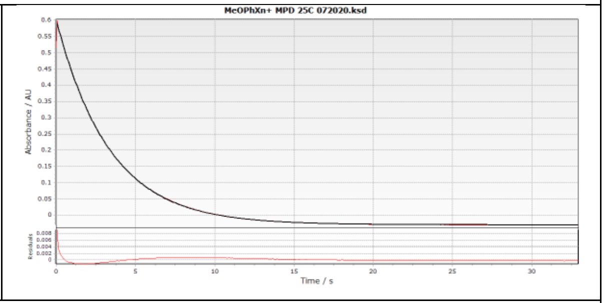

25°C D

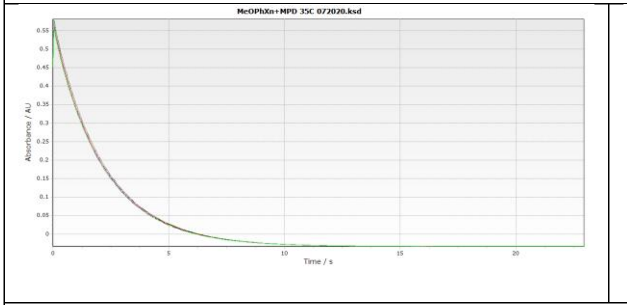

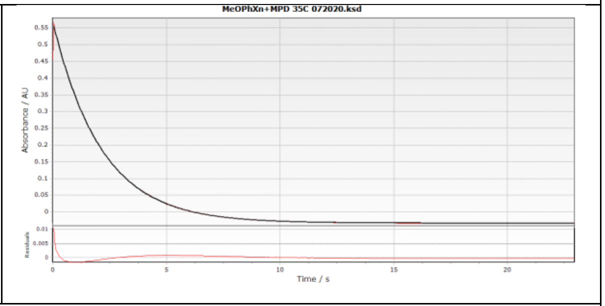

35°C D

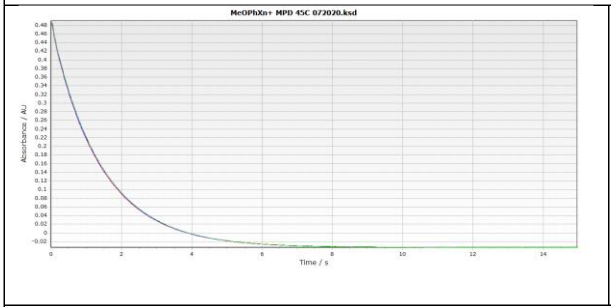

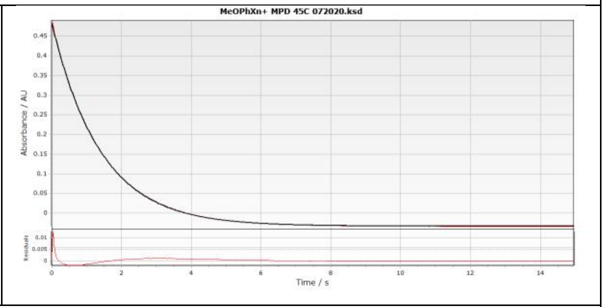

45°C D

Day 2 data (July 21, 2020)

	Pseudo-first-order rate constants k^{pfo} (s^{-1})									
**Temp (°C)**	**Trial H1**	**Trial H2**	**Trial H3**	**Trial H4**	**Trial H5**	**Trial H6**	**Average k_H^{pfo} (s^{-1})**	**Stdev**	**k_{2H} ($M^{-1}s^{-1}$)**	**Stdev [a]**
45	2.03018	2.03954	2.03937	2.03964	2.04643	2.05189	2.0412	7.37E-03	3.28E+03	1.18E+01
35	1.40968	1.39023	1.39352	1.38886	1.39570	1.38737	1.3942	8.16E-03	2.24E+03	1.31E+01
25	0.92304	0.91386	0.92117	0.91425	0.91958	0.91725	0.9182	3.73E-03	1.47E+03	5.98E+00
15	0.59009	0.59732	0.59991	0.58969	0.59685	0.60119	0.5958	4.88E-03	9.56E+02	7.84E+00
5	0.37278	0.37332	0.36810	0.36770	0.36676	0.37134	0.3700	2.83E-03	5.94E+02	4.54E+00
**Temp (°C)**	**Trial D1**	**Trial D2**	**Trial D3**	**Trial D4**	**Trial D5**	**Trial D6**	**Average k_D^{pfo} (s-1)**	**Stdev**	**k_{2D} (M-1s-1)**	**Stdev [a]**
45	0.70486	0.70459	0.70381	0.69998	0.70387	0.70677	0.7040	2.23E-03	1.03E+03	3.27E+00
35	0.45887	0.46342	0.46634	0.46903	0.46584	0.46580	0.4649	3.45E-03	6.80E+02	5.04E+00
25	0.29418	0.29377	0.29482	0.29598	0.29377	0.29660	0.2949	1.19E-03	4.31E+02	1.75E+00
15	0.18299	0.18332	0.18225	0.18335	0.18444	0.18563	0.1837	1.19E-03	2.69E+02	1.75E+00
5	0.11031	0.11031	0.10986	0.11041	0.10915	0.11087	0.1102	5.87E-04	1.61E+02	8.58E-01

[a] = (Stdev(for k^{pfo})/k^{pfo})*k_2

Six kinetic trials and the mean fit	First-order kinetic fitted average of six trials
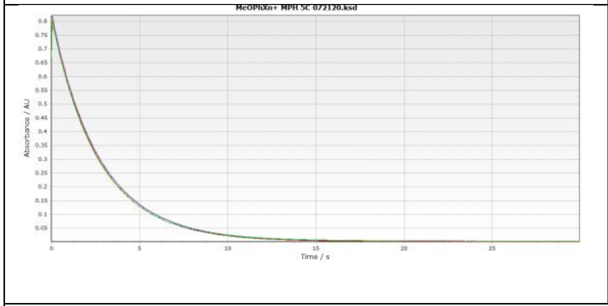	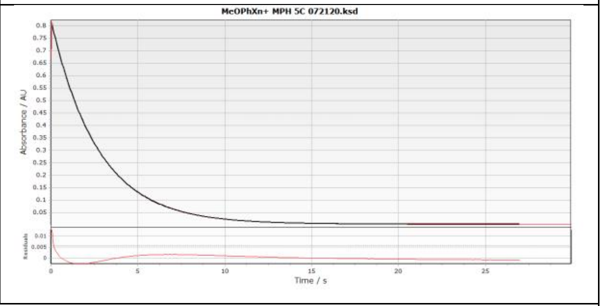

5°C H

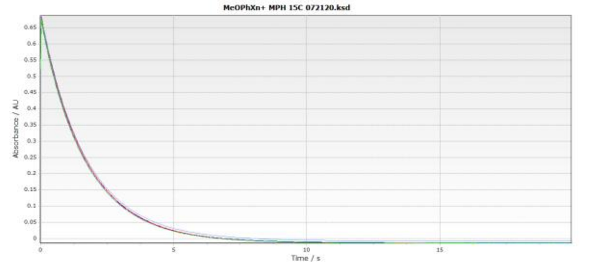	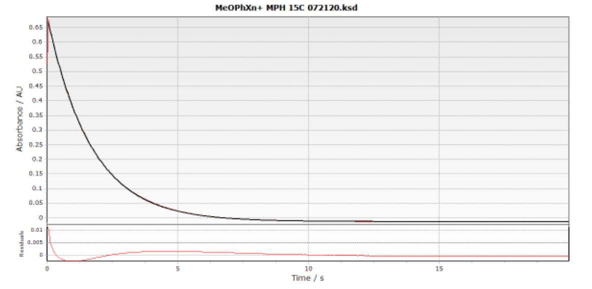

15°C H

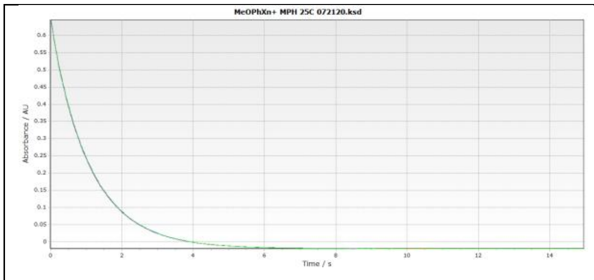

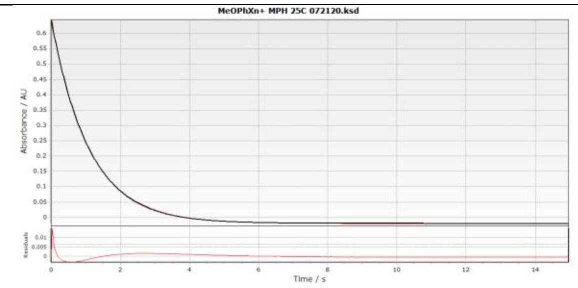

25℃ H

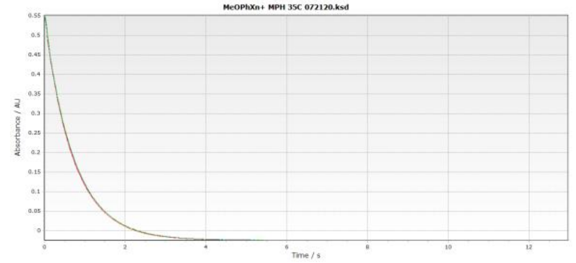

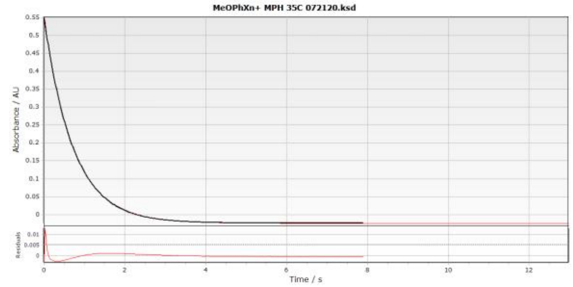

35℃ H

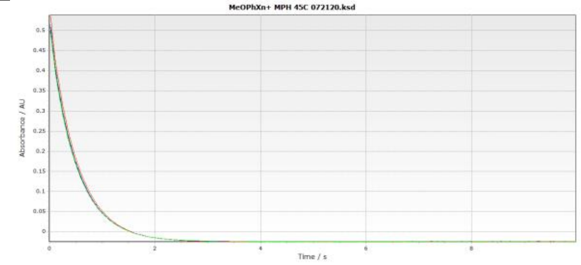

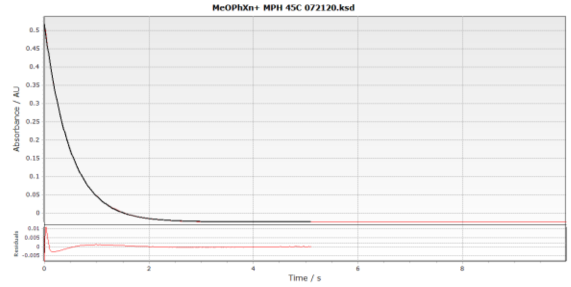

45℃ H

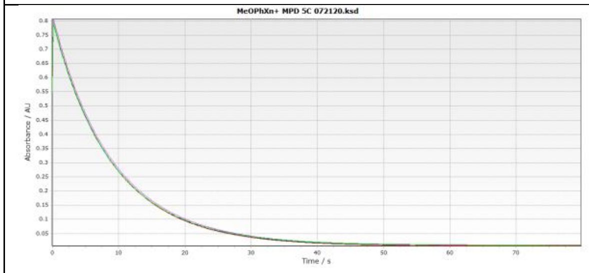

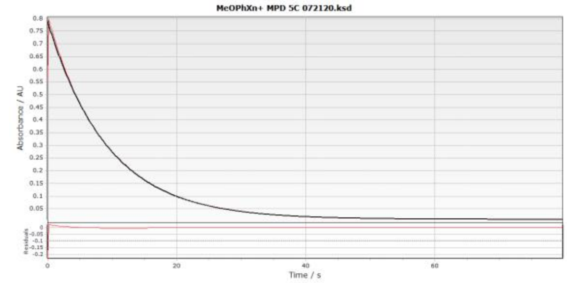

5℃ D

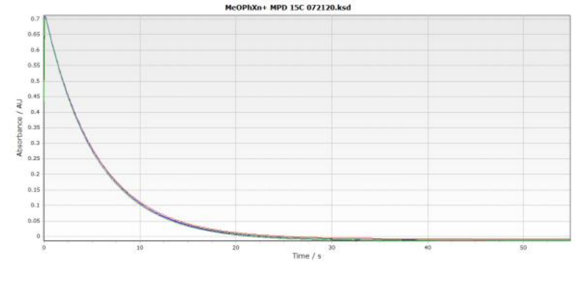

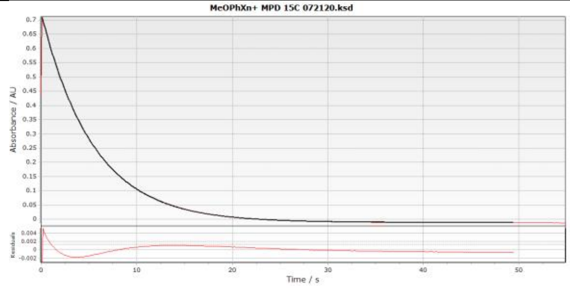

15℃ D

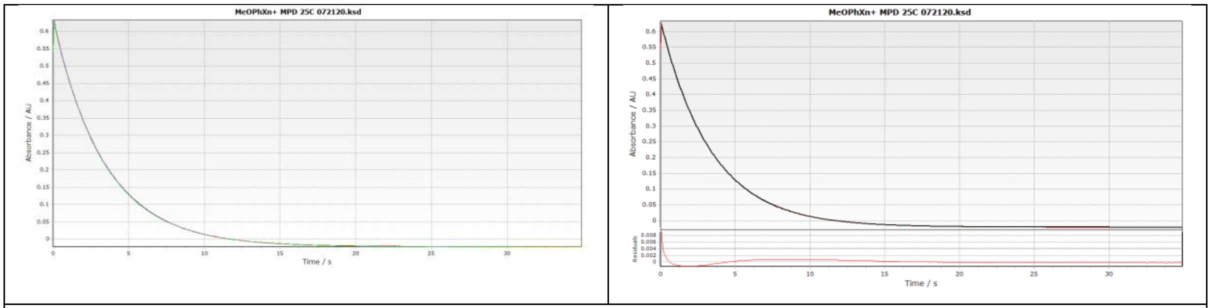

25°C D

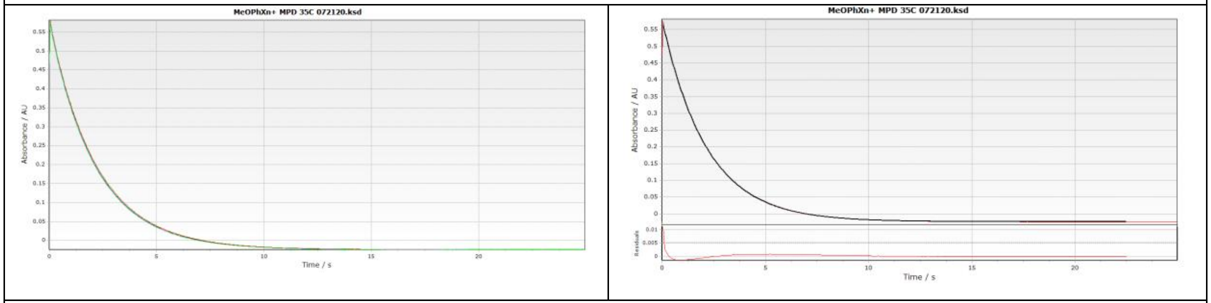

35°C D

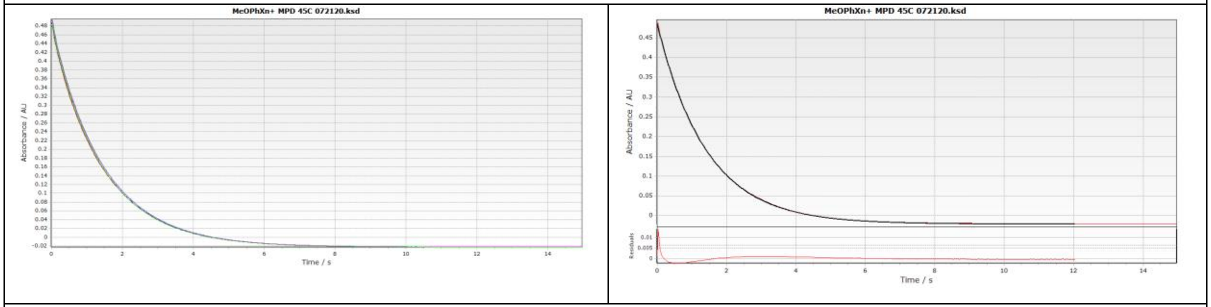

45°C D

Day 3 data (January 22, 2025)

Pseudo-first-order rate constants

k^{pfo} (s^{-1})

Temp (°C)	Trial H1	Trial H2	Trial H3	Trial H4	Trial H5	Trial H6	Average k_H^{pfo} (s^{-1})	Stdev	k_{2H} ($M^{-1}s^{-1}$)	Stdev [a]
45	7.96999	7.92687	7.99990	8.00746	7.92577	7.95799	7.96466	0.0348916	3.98E+03	17.44579
35	5.37474	5.39941	5.36986	5.35730	5.41445	5.29595	5.36862	0.0412225	2.68E+03	20.61125
25	3.54559	3.55609	3.54155	3.57150	3.58253	3.55363	3.55848	0.015684	1.78E+03	7.84198
15	2.29914	2.32300	2.31689	2.30586	2.27704	2.30285	2.30413	0.0160143	1.15E+03	8.00717
5	1.38597	1.40232	1.42583	1.40672	1.41253	1.40689	1.40671	0.01303	7.03E+02	6.51498

Temp (°C)	Trial D1	Trial D2	Trial D3	Trial D4	Trial D5	Trial D6	Average k_D^{pfo} (s-1)	Stdev	k_{2D} (M-1s-1)	Stdev [a]
45	2.70688	2.65809	2.68091	2.69456	2.71427	2.71017	2.6941	0.021444332	1.35E+03	10.72217
35	1.78085	1.76016	1.73442	1.76055	1.77106	1.78103	1.7647	0.017450792	8.82E+02	8.72540
25	1.10537	1.12417	1.11052	1.11446	1.13129	1.11012	1.1160	0.009803117	5.58E+02	4.90156
15	0.69482	0.70518	0.69997	0.70602	0.69176	0.69125	0.6982	0.00654522	3.49E+02	3.27261
5	0.40901	0.41469	0.41844	0.41698	0.41346	0.41527	0.4146	0.003268158	2.07E+02	1.63408

[a] = (Stdev(for k^{pfo})/k^{pfo})*k_2

Six kinetic trials and the mean fit	First-order kinetic fitted average of six trials
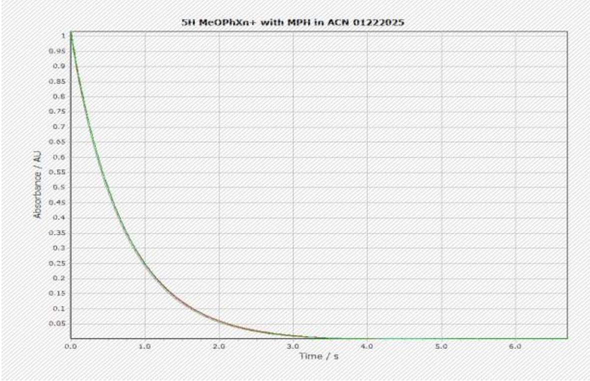	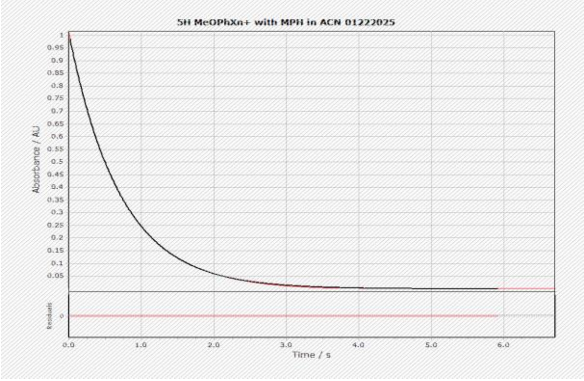
5℃ H	
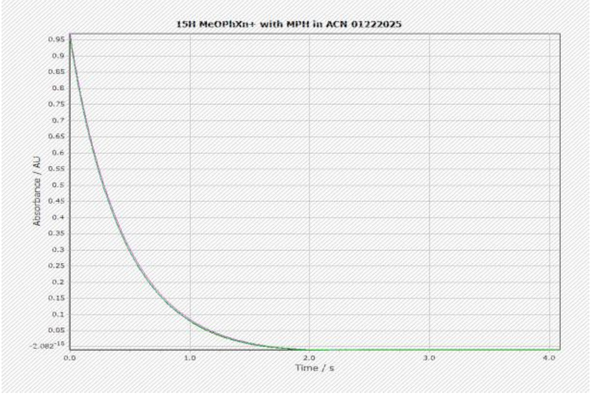	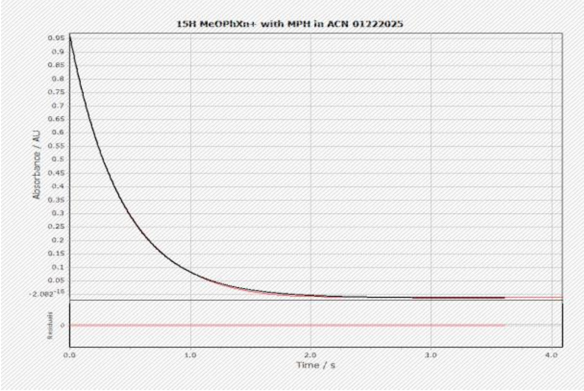
15℃ H	
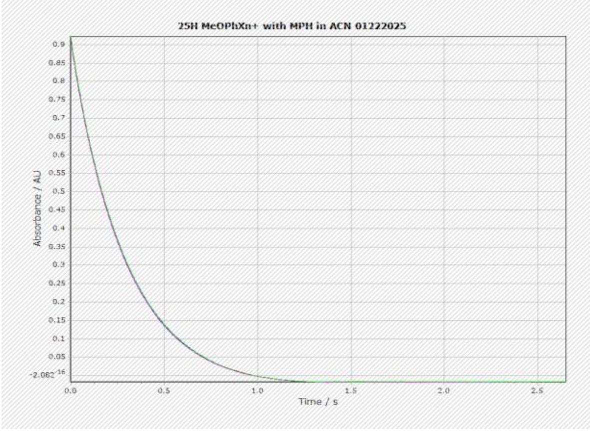	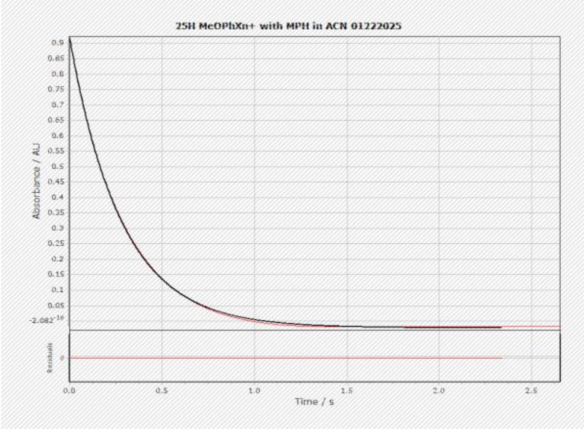
25℃ H	

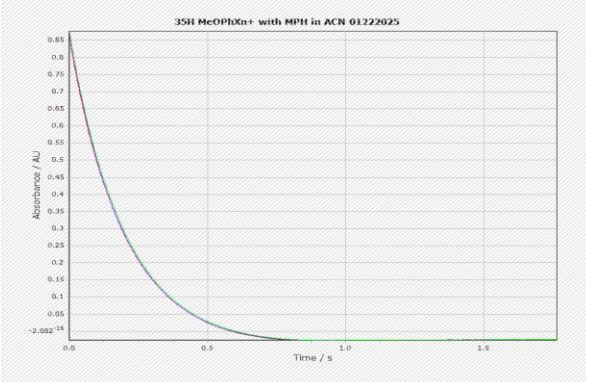
35H MeOPhXn+ with MPH in ACN 01222025
Absorbance / AU
Time / s

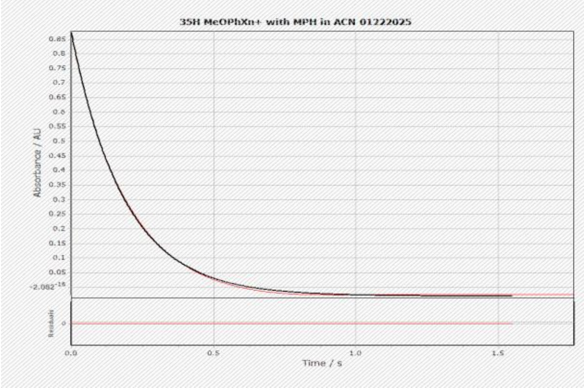
35H MeOPhXn+ with MPH in ACN 01222025
Absorbance / AU
Residuals
Time / s

35℃ H

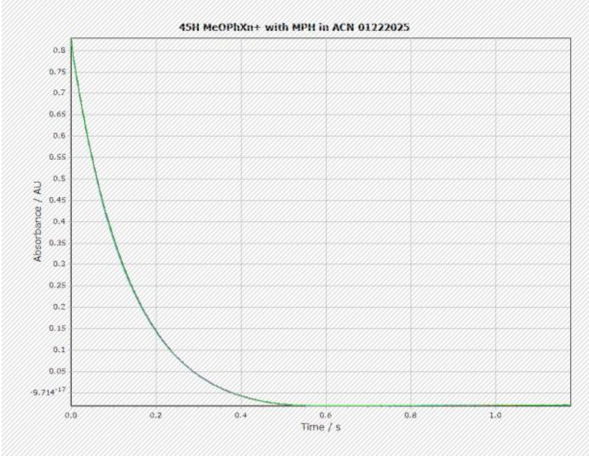
45H MeOPhXn+ with MPH in ACN 01222025
Absorbance / AU
Time / s

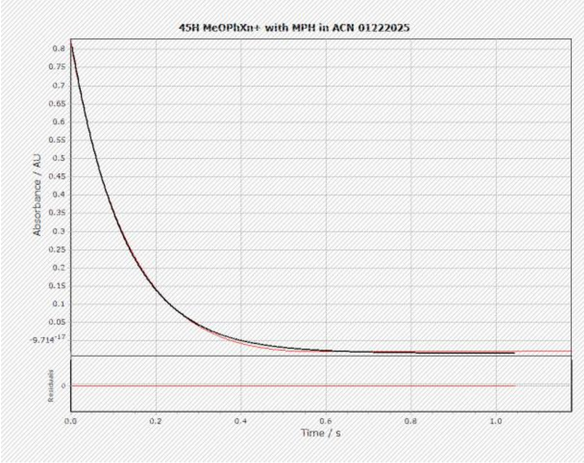
45H MeOPhXn+ with MPH in ACN 01222025
Absorbance / AU
Residuals
Time / s

45℃ H

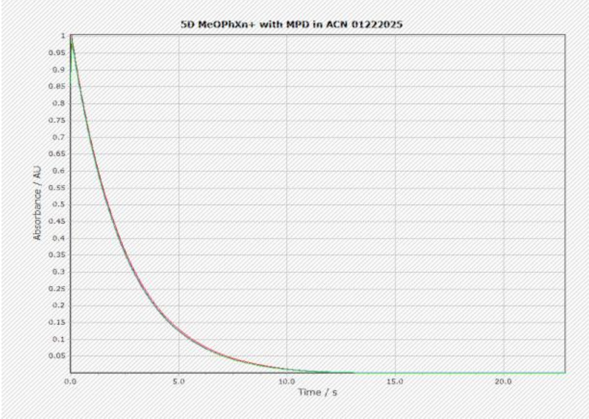
5D MeOPhXn+ with MPD in ACN 01222025
Absorbance / AU
Time / s

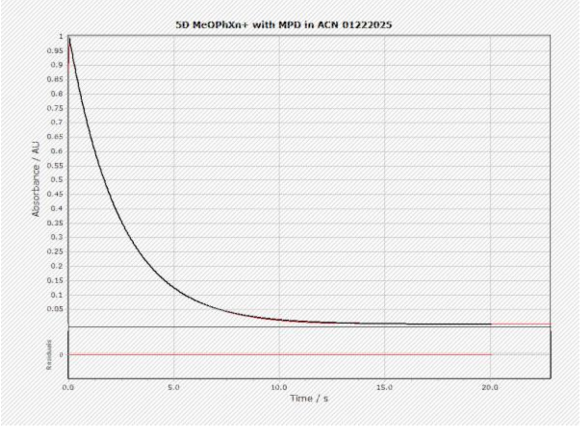
5D MeOPhXn+ with MPD in ACN 01222025
Absorbance / AU
Residuals
Time / s

5℃ D

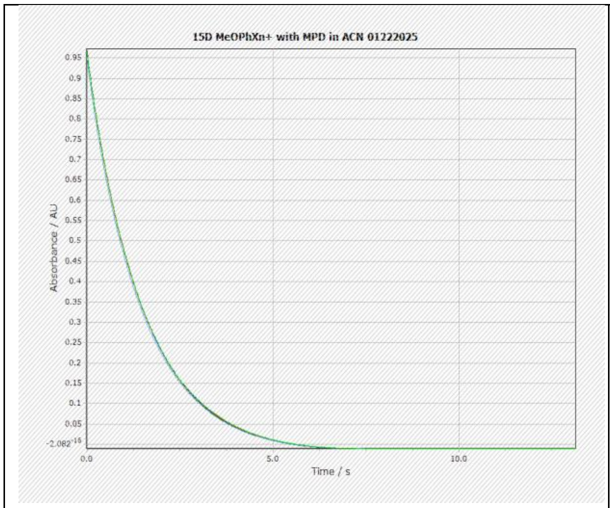

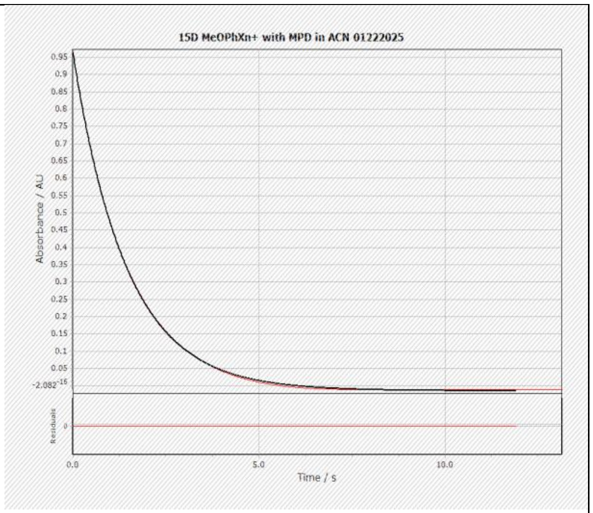

15°C D

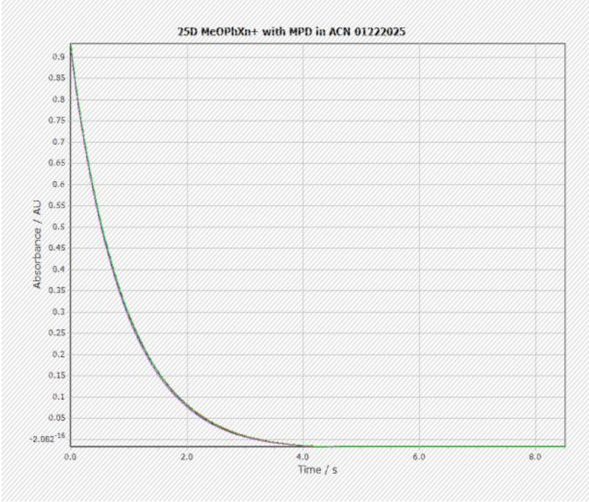

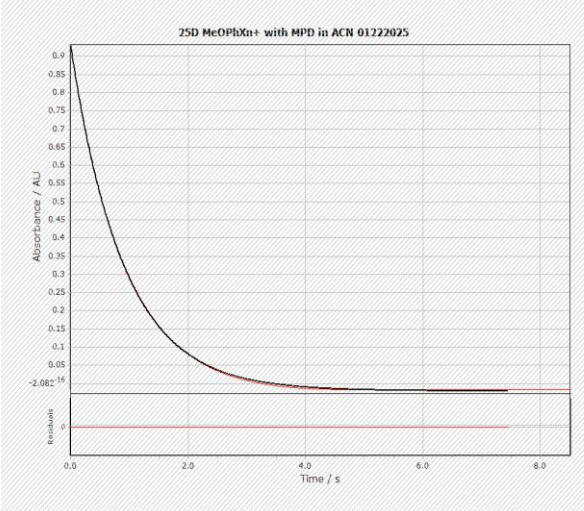

25°C D

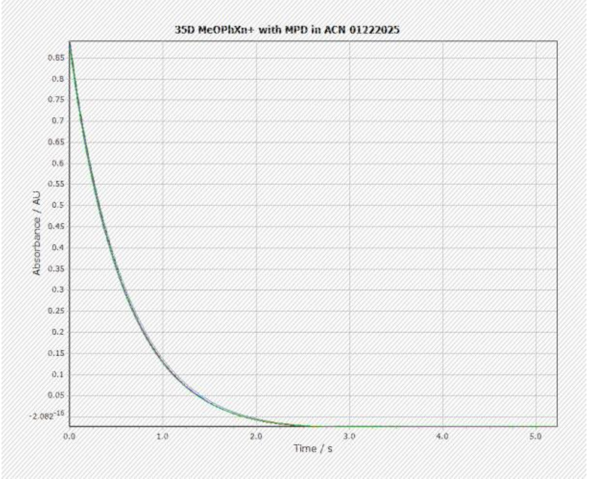

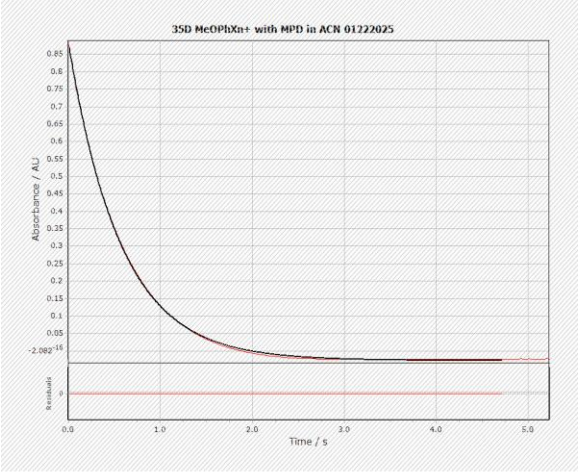

35°C D

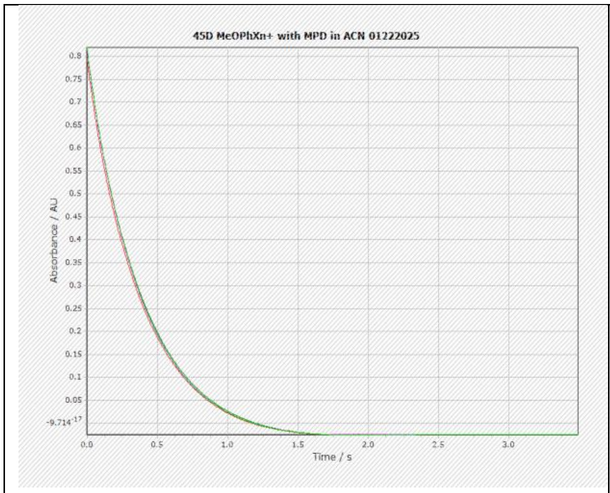

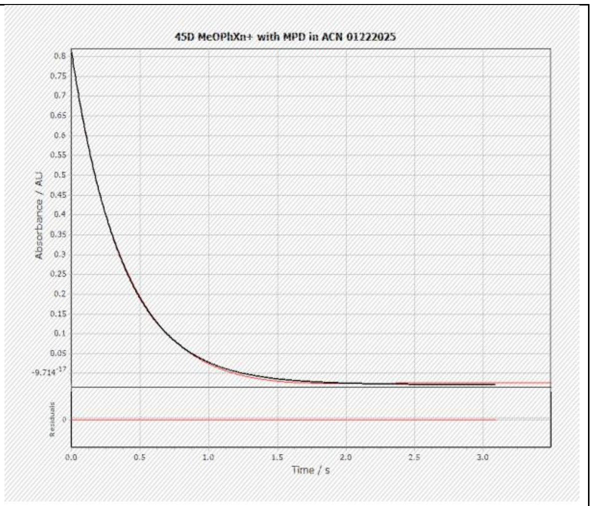

45℃ D

Day 4 data
(January 23, 2025)

Temp (°C)	Trial H1	Trial H2	Trial H3	Trial H4	Trial H5	Trial H6	Average k_H^{pfo} (s^{-1})	Stdev	k_{2H} ($M^{-1}s^{-1}$)	Stdev [a]
	Pseudo-first-order rate constants k^{pfo} (s^{-1})									
45	7.92687	7.75982	7.96936	7.99483	7.98046	7.88474	7.91935	8.79E-02	3.96E+03	4.40E+01
35	5.24557	5.27191	5.31957	5.33621	5.35846	5.32165	5.30890	4.21E-02	2.65E+03	2.10E+01
25	3.52840	3.53806	3.58429	3.60230	3.58995	3.54652	3.56492	3.10E-02	1.78E+03	1.55E+01
15	2.27558	2.26821	2.26127	2.28279	2.28784	2.28312	2.27647	1.01E-02	1.14E+03	5.07E+00
5	1.40160	1.40016	1.40070	1.41495	1.41035	1.41594	1.40728	7.34E-03	7.04E+02	3.67E+00
**Temp (°C)**	**Trial D1**	**Trial D2**	**Trial D3**	**Trial D4**	**Trial D5**	**Trial D6**	**Average k_D^{pfo} (s-1)**	**Stdev**	**k_{2D} (M-1s-1)**	**Stdev [a]**
45	2.68373	2.66639	2.65473	2.67308	2.66578	2.65907	2.6671	1.03E-02	1.33E+03	5.16E+00
35	1.69646	1.69628	1.70859	1.71452	1.73634	1.70959	1.7103	1.47E-02	8.55E+02	7.37E+00
25	1.08770	1.10885	1.09574	1.11006	1.08900	1.10814	1.0999	1.04E-02	5.50E+02	5.18E+00
15	0.68509	0.68762	0.68400	0.68400	0.69008	0.68168	0.6854	2.99E-03	3.43E+02	1.49E+00
5	0.40809	0.41201	0.41445	0.41101	0.41024	0.41440	0.4117	2.47E-03	2.06E+02	1.24E+00

[a] = (Stdev(for k^{pfo})/k^{pfo})*k_2

Six kinetic trials and the mean fit	First-order kinetic fitted average of six trials

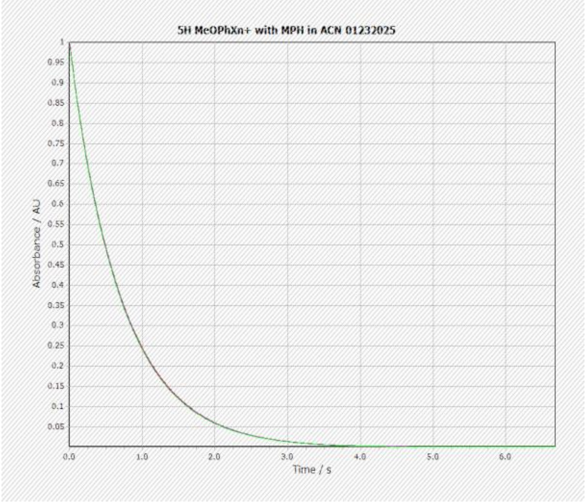

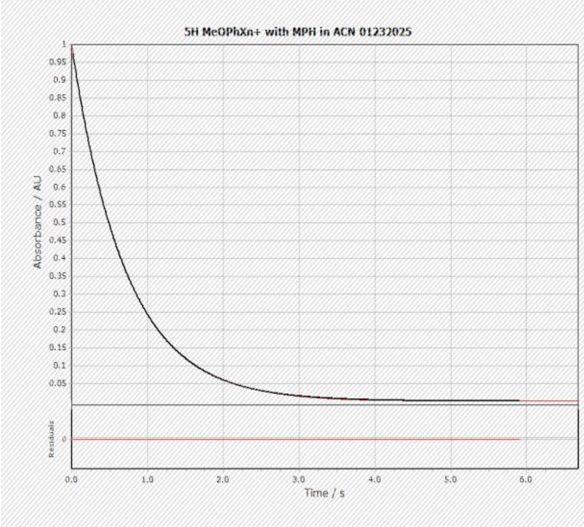

5°C H

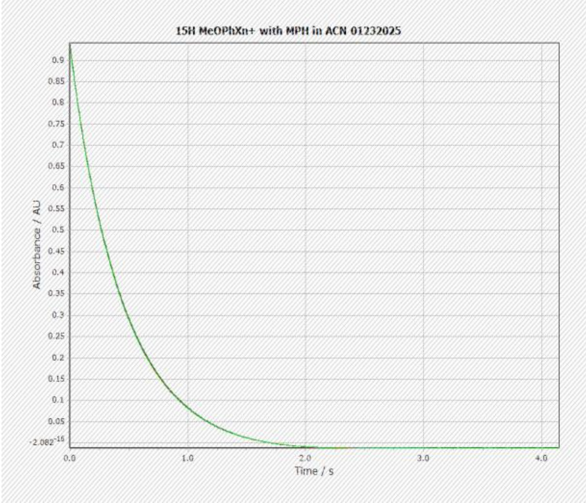

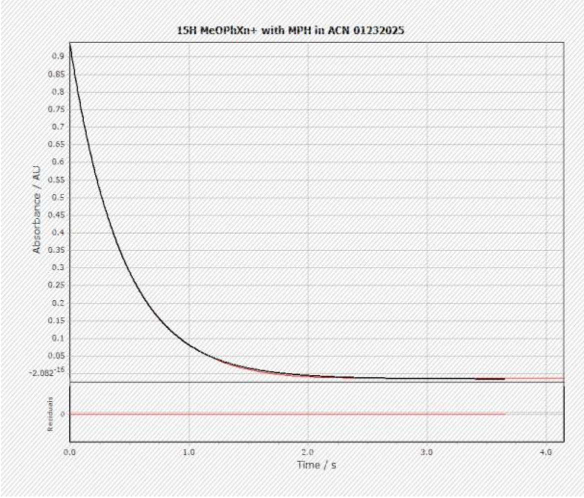

15°C H

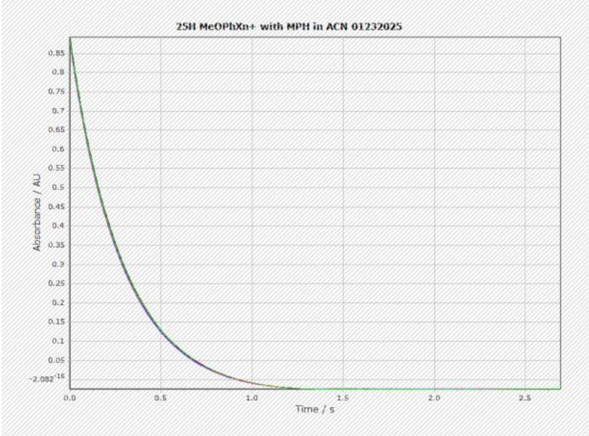

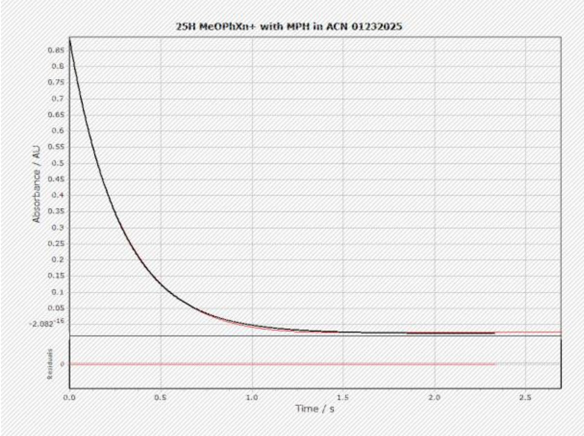

25°C H

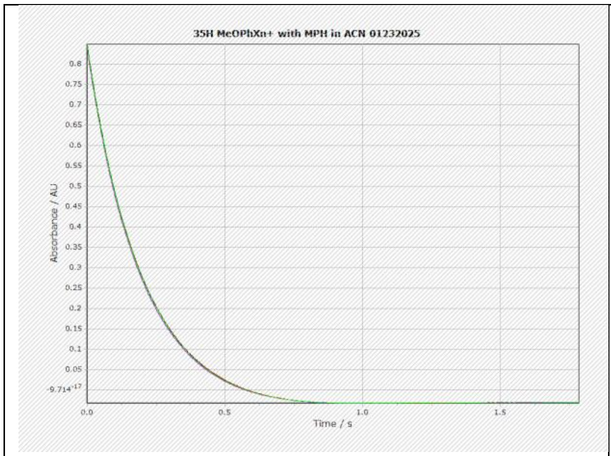

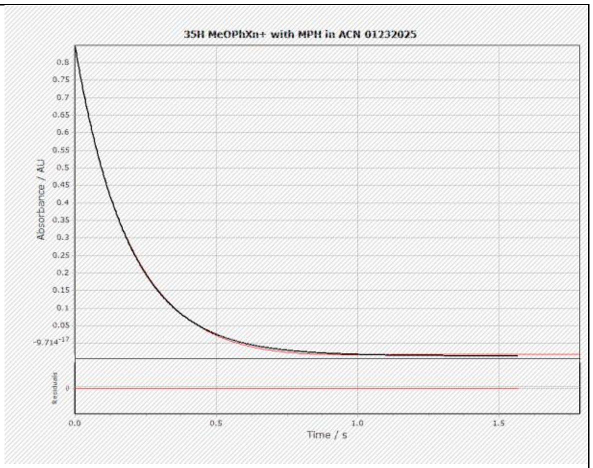

35°C H

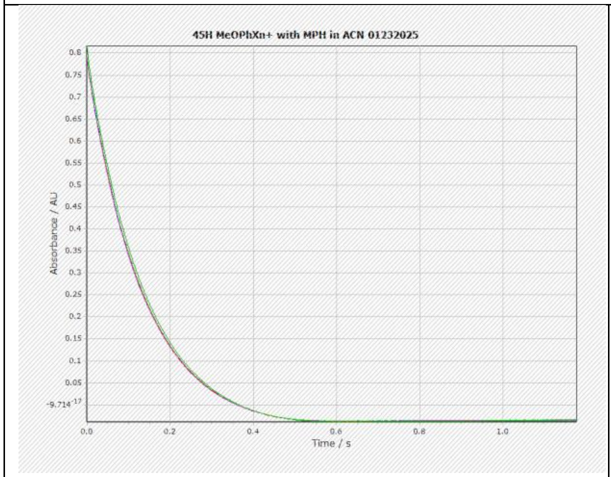

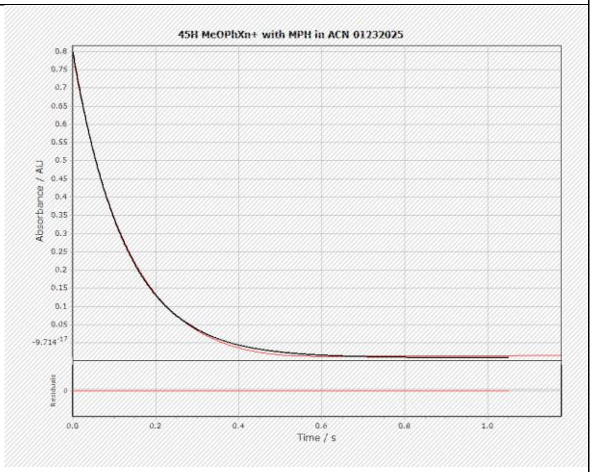

45°C H

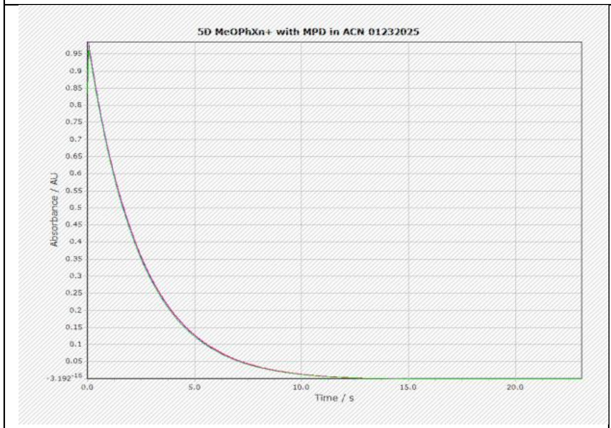

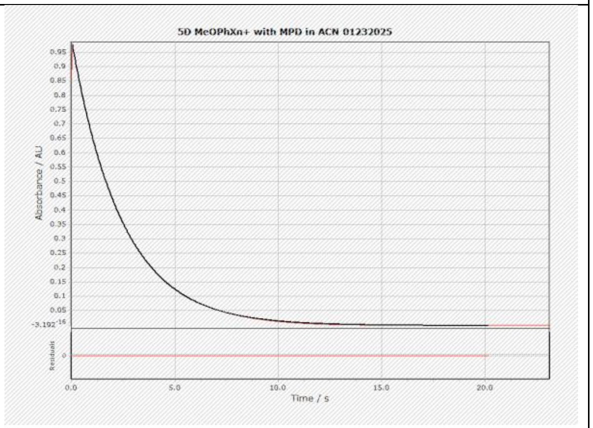

5°C D

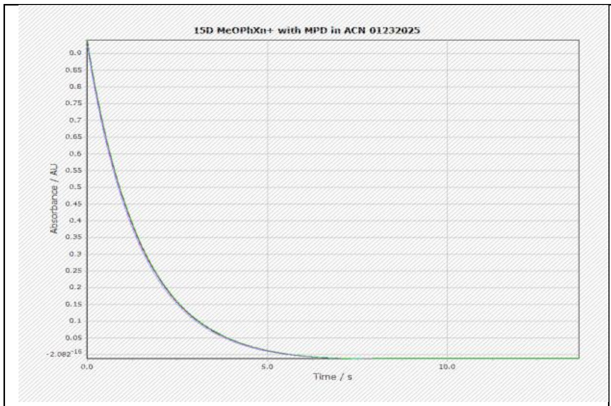

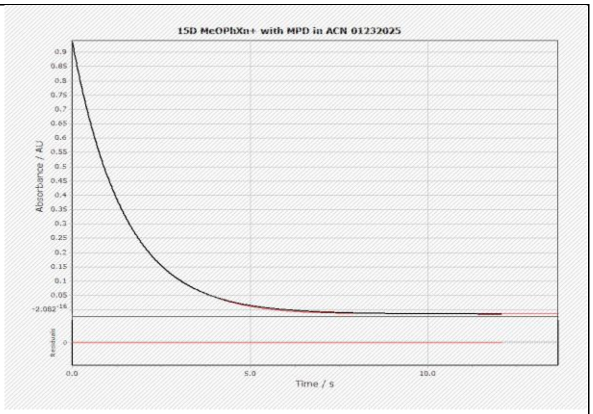

15°C D

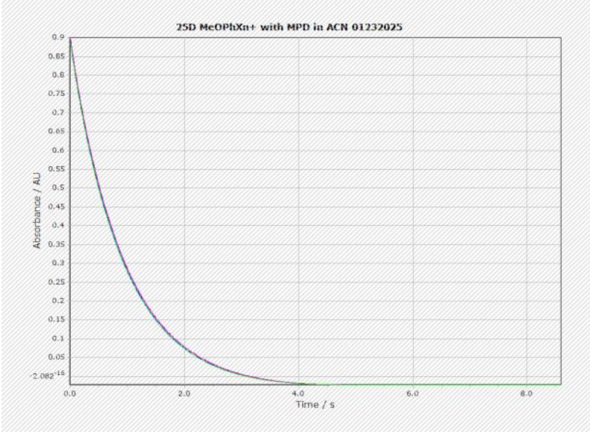

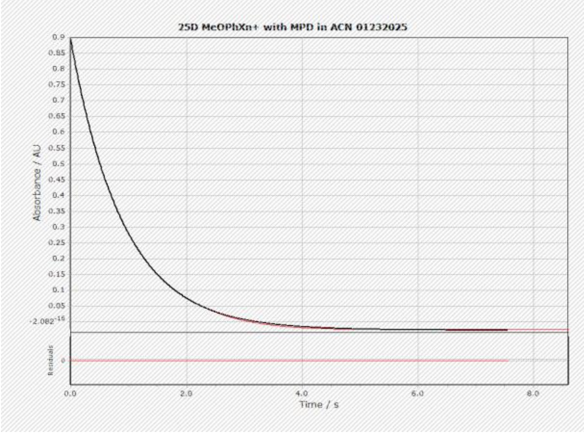

25°C D

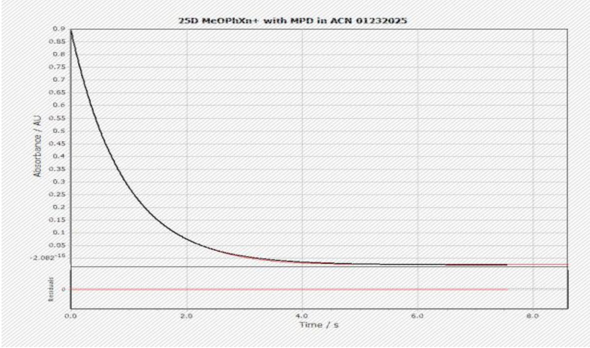

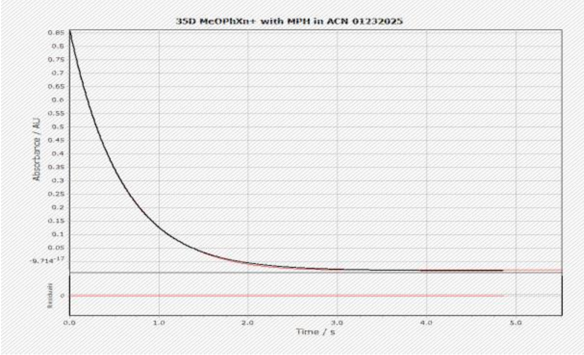

35°C D

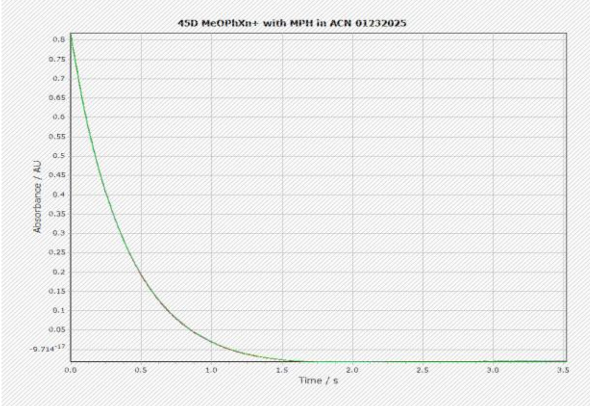

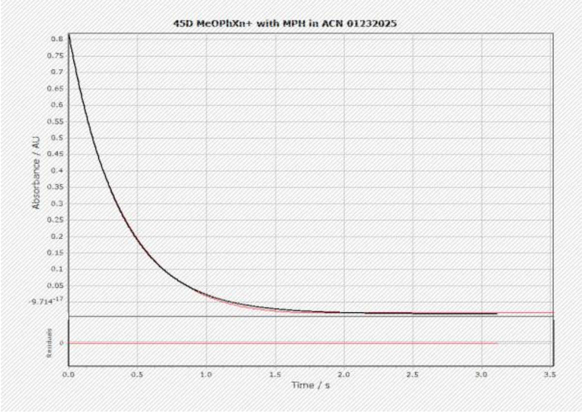

45°C D

Primary kinetic data for the rate constants in Table S5 (BNAH with (p-MeOPh)$_3$C$^+$)

Day 1 data (July 31, 2024)

	Pseudo-first-order rate constants k^{pfo} (s^{-1})									
**Temp (°C)**	**Trial H1**	**Trial H2**	**Trial H3**	**Trial H4**	**Trial H5**	**Trial H6**	**Average k_H^{pfo} (s^{-1})**	**Stdev**	**k_{2H} ($M^{-1}s^{-1}$)**	**Stdev [a]**
45	19.1665	19.0479	18.8111	18.7981	18.6738	19.0454	18.9238	0.1898	4.86E+04	487.37
35	15.8562	15.7639	15.6811	15.5460	15.7197	15.5785	15.6909	0.1159	4.03E+04	297.75
25	13.0514	13.2600	13.2595	13.2454	13.1066	13.0149	13.1563	0.1121	3.38E+04	287.83
15	10.4685	10.5742	10.3994	10.5110	10.5338	10.4315	10.4864	0.0655	2.69E+04	168.28
5	8.2608	8.2842	8.4112	8.3032	8.3334	8.3366	8.3216	0.0526	2.14E+04	135.06

**Temp (°C)**	**Trial D1**	**Trial D2**	**Trial D3**	**Trial D4**	**Trial D5**	**Trial D6**	**Average k_D^{pfo} (s^{-1})[b]**	**Stdev**	**k_{2D} ($M^{-1}s^{-1}$)**	**Stdev [a]**
45	7.5366	7.5240	7.4733	7.4743	7.5208	7.4757	7.0248	0.0294	1.80E+04	75.38
35	6.1102	6.1150	5.9790	6.0562	6.0346	6.0706	5.6597	0.0507	1.45E+04	130.32
25	4.8363	4.8222	4.8239	4.8520	4.8896	4.7747	4.4863	0.0379	1.15E+04	97.25
15	3.7357	3.7094	3.7490	3.7748	3.7542	3.7449	3.4638	0.0216	8.09E+03	55.55
5	2.8712	2.8585	2.9048	2.8596	2.8495	2.8498	2.6382	0.0208	6.78E+03	53.37

[a] = (Stdev(for k^{pfo})/k^{pfo})*k_2

[b] = Average k_D^{pfo} – (0.04*Average k_H^{pfo})/0.96 due to 4% H content

Six kinetic runs together with the averaged data	First-order kinetic fit of the averaged data as an example
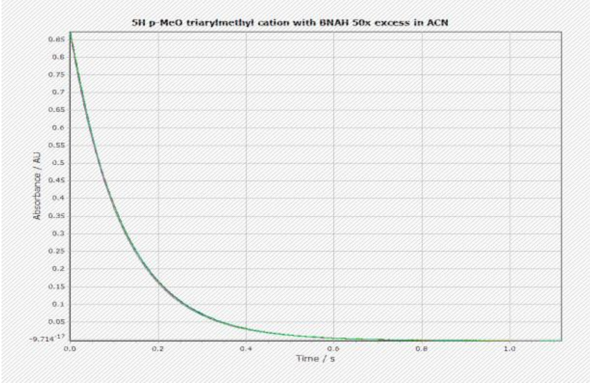	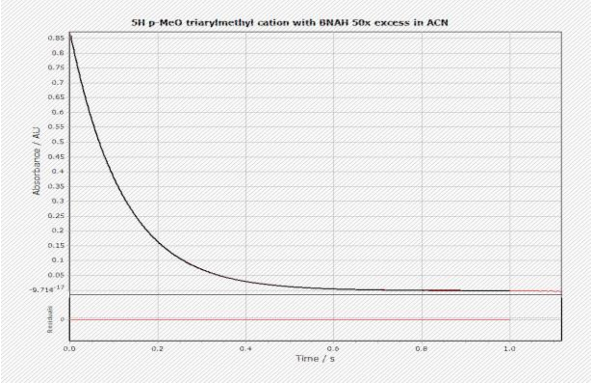

5°C H

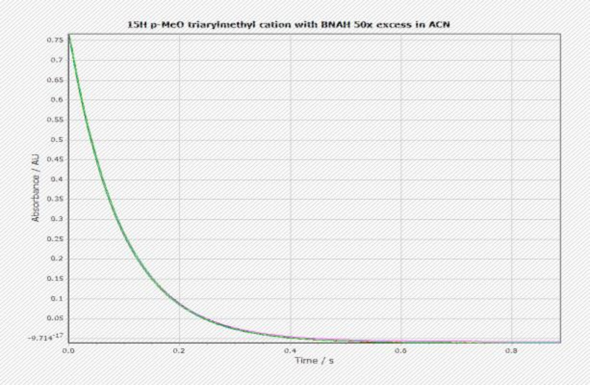

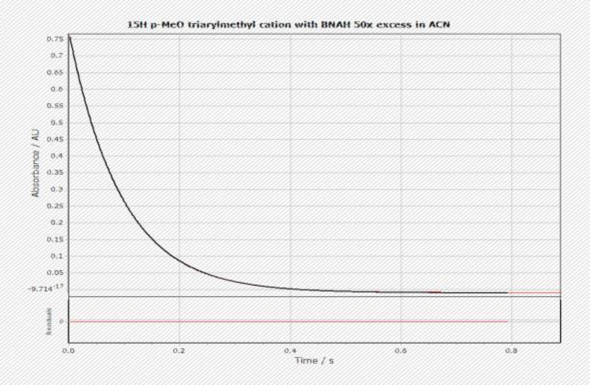

15°C H

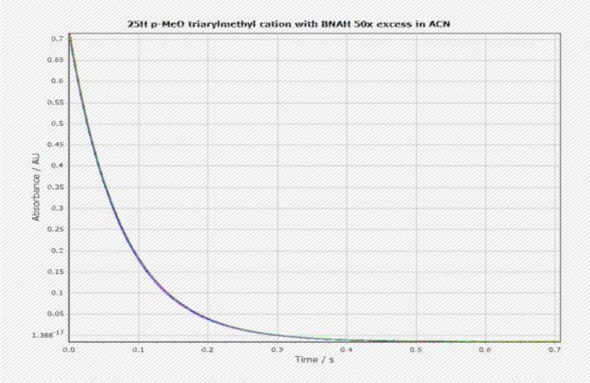

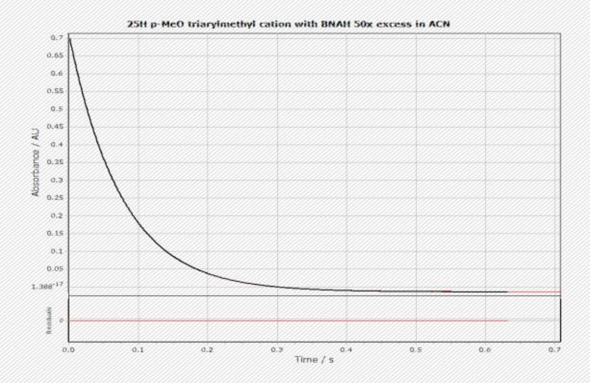

25°C H

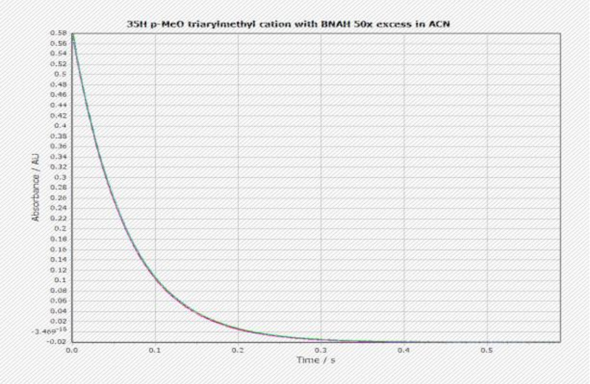

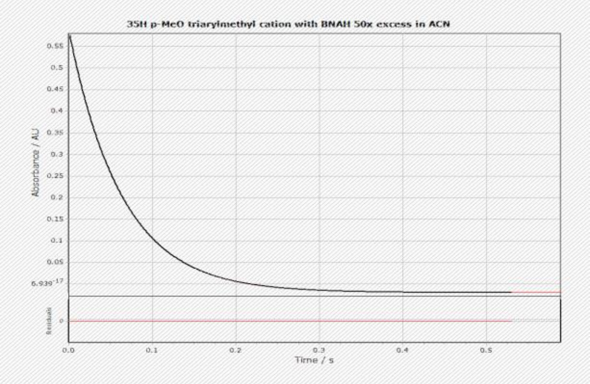

35°C H

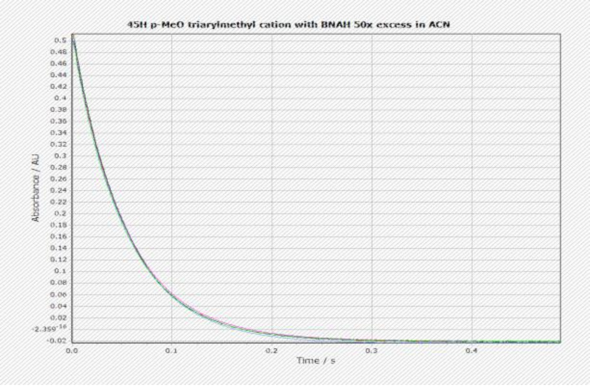

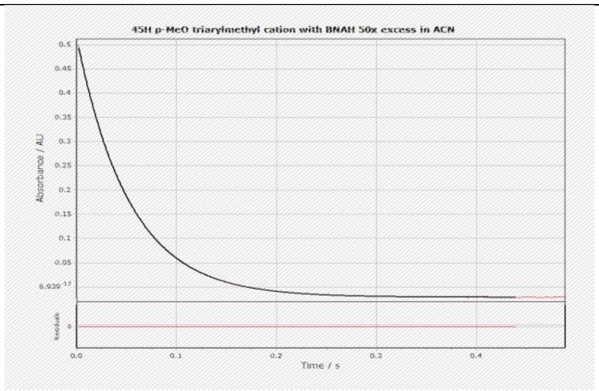

45°C H

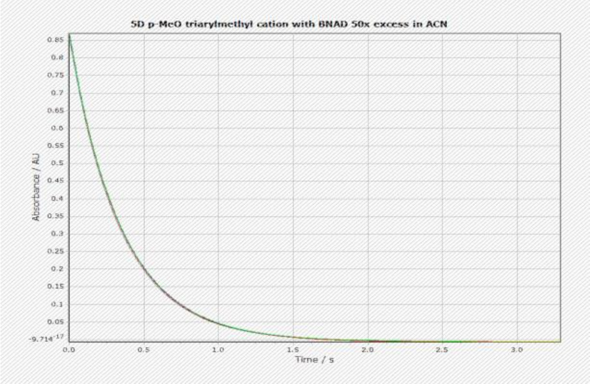

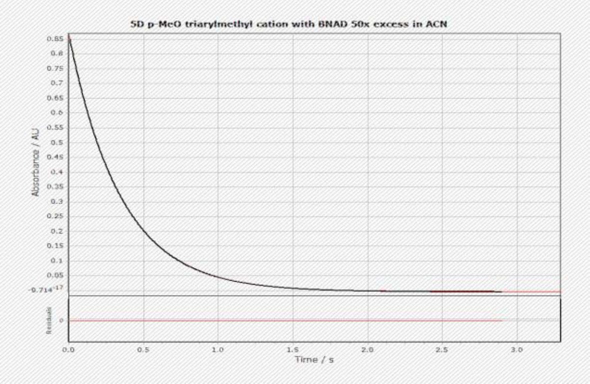

5°C D

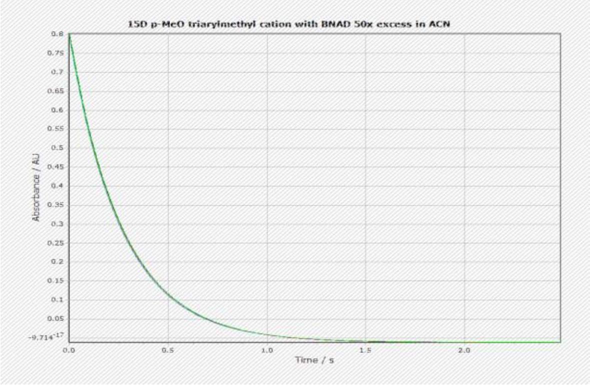

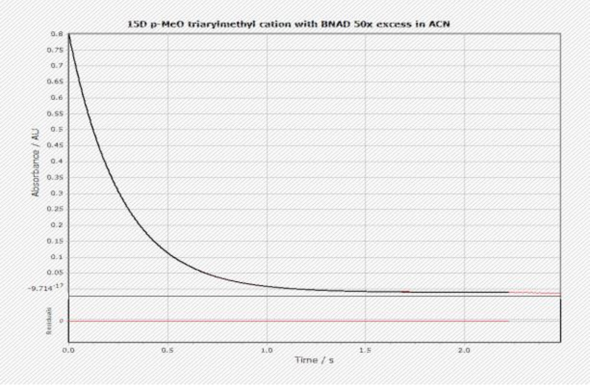

15°C D

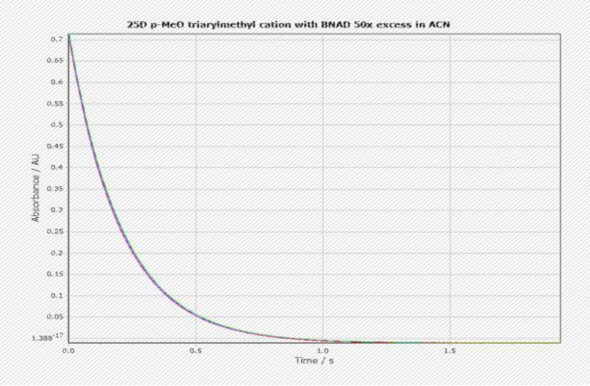	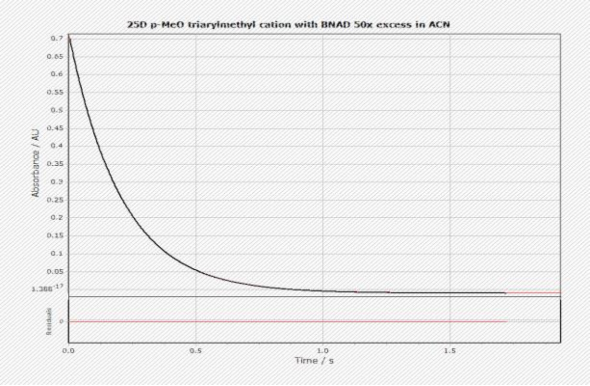
25°C D	
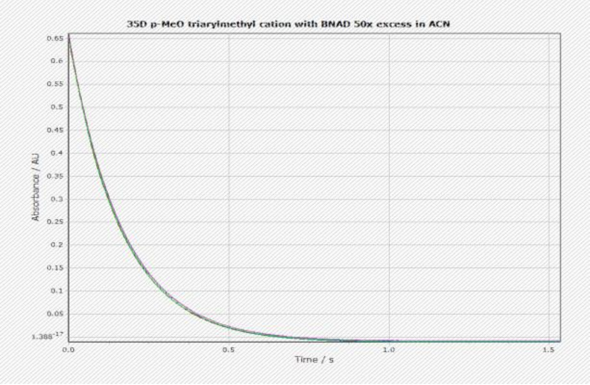	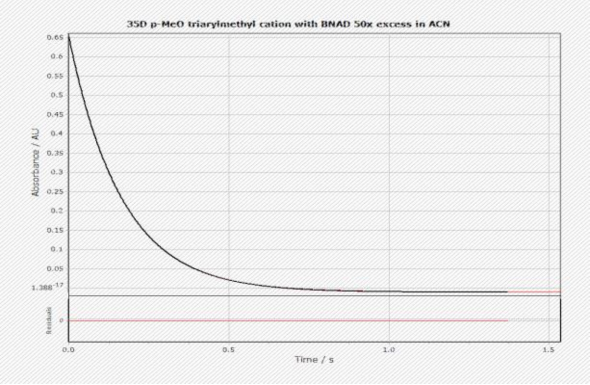
35°C D	
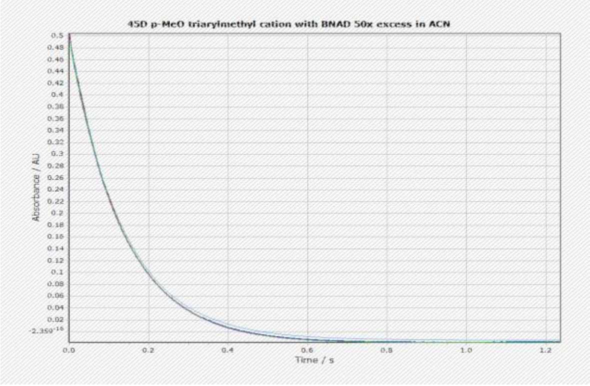	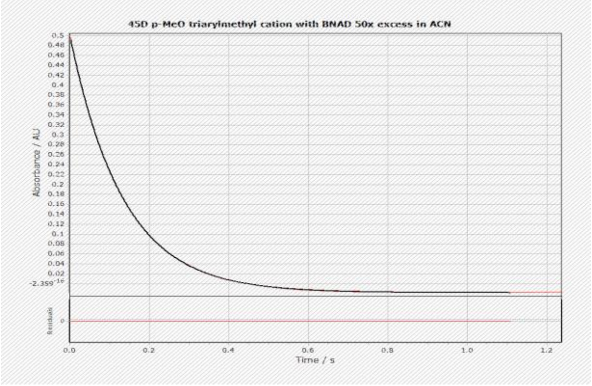
45°C D	

Day 2 (1) data (August 5, 2024)

	Pseudo-first-order rate constants k^{pfo} (s^{-1})									
**Temp (°C)**	**Trial H1**	**Trial H2**	**Trial H3**	**Trial H4**	**Trial H5**	**Trial H6**	**Average k_H^{pfo} (s^{-1})**	**Stdev**	**k_{2H} ($M^{-1}s^{-1}$)**	**Stdev [a]**
45	18.6097	18.5759	18.9519	18.7876	19.0496	19.0453	18.8367	0.2117	4.84E+04	543.69
35	15.8051	15.8620	15.6015	15.8186	15.8459	15.5618	15.7492	0.1319	4.04E+04	338.65
25	12.9943	12.7962	12.8359	12.8354	12.6725	12.8410	12.8292	0.1031	3.29E+04	264.72
15	10.4292	10.3138	10.2757	10.3215	10.4869	10.3931	10.3700	0.0801	2.66E+04	205.59
5	8.2375	8.2290	8.3091	8.4168	8.3743	8.2475	8.3024	0.0786	2.13E+04	201.97

Temp (°C)	Trial D1	Trial D2	Trial D3	Trial D4	Trial D5	Trial D6	Average k_D^{pfo} (s^{-1})[b]	Stdev	k_{2D} ($M^{-1}s^{-1}$)	Stdev [a]
45	7.8296	7.8860	7.8717	7.7471	7.9144	7.8025	7.3838	0.0613	1.90E+04	157.42
35	6.3670	6.2964	6.2744	6.3121	6.3180	6.3066	5.9192	0.0308	1.52E+04	79.15
25	5.0584	5.0538	5.0047	5.0172	5.0190	4.9925	4.6991	0.0265	1.21E+04	68.08
15	3.8715	3.8978	3.8783	3.8858	3.8841	3.9050	3.6170	0.0124	9.29E+03	31.83
5	2.9695	2.9437	2.9638	2.9629	2.9578	2.9303	2.7319	0.0148	7.02E+03	38.02

[a] = (Stdev(for k^{pfo})/k^{pfo})*k_2

[b] = (Average k_D^{pfo} – (0.04*Average k_H^{pfo})/0.96 due to 4% H content

Six kinetic runs together with the averaged data	First-order kinetic fit of the averaged data as an example
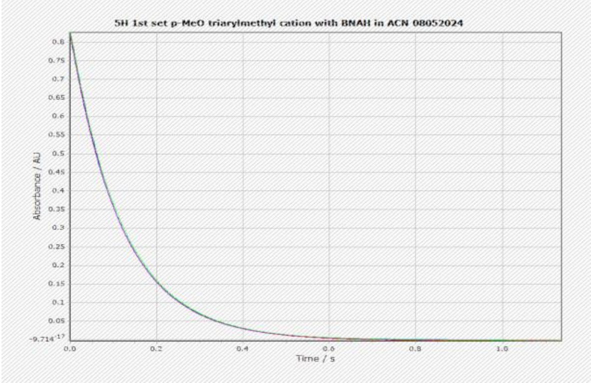	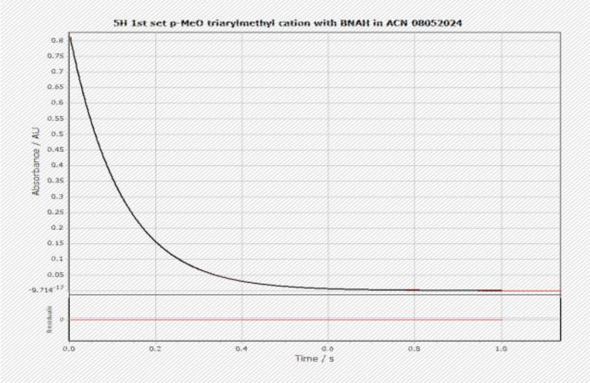
5°C H	
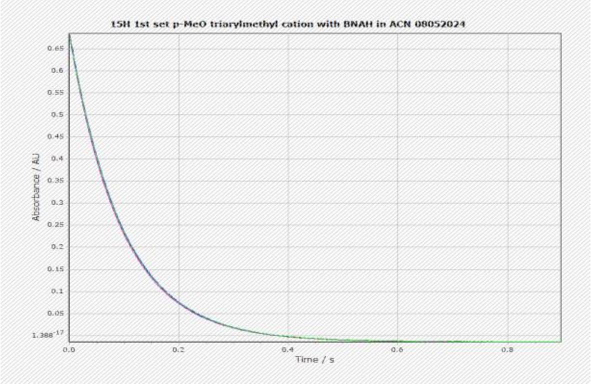	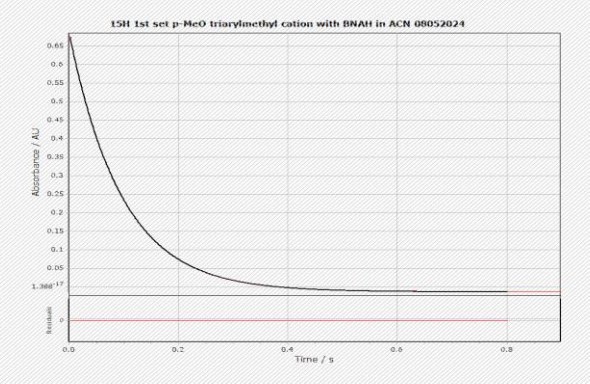
15°C H	

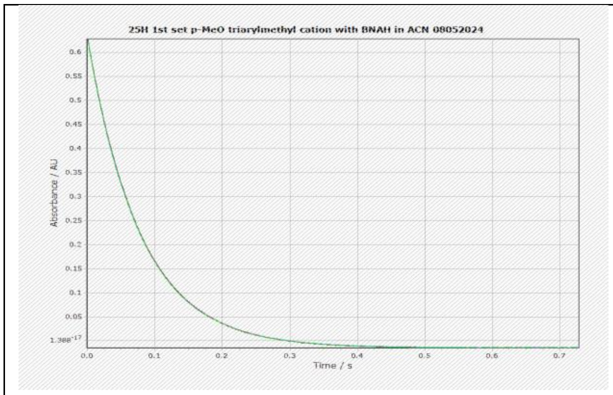

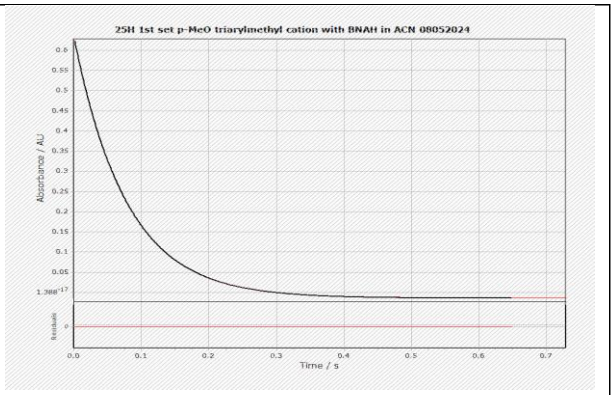

25°C H

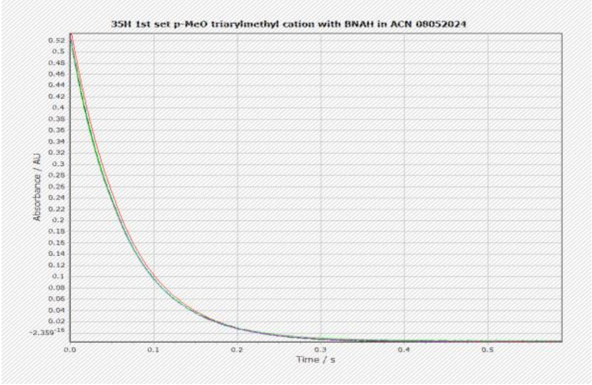

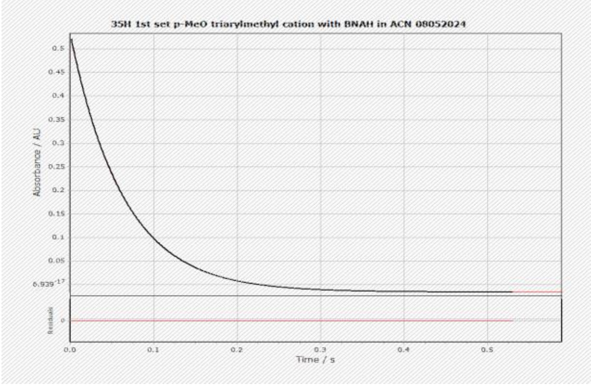

35°C H

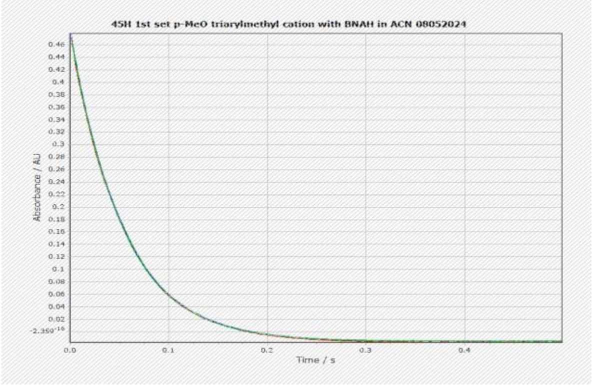

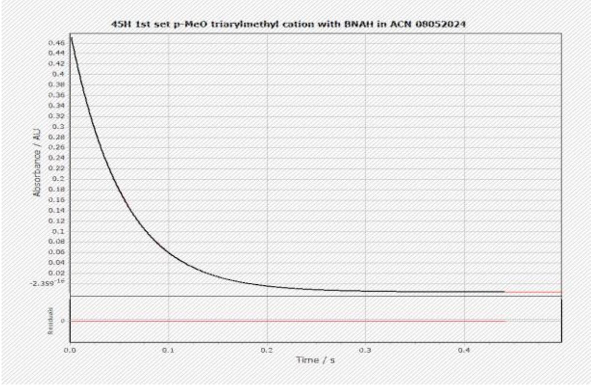

45°C H

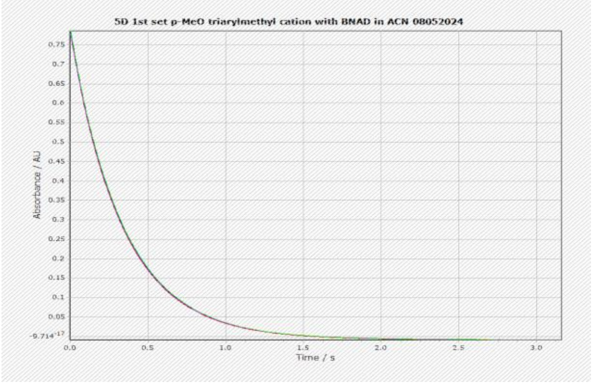

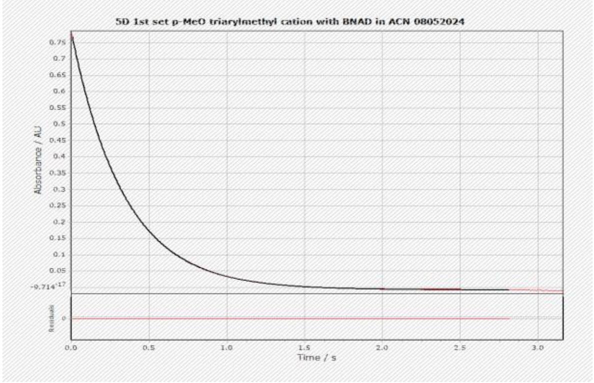

5°C D

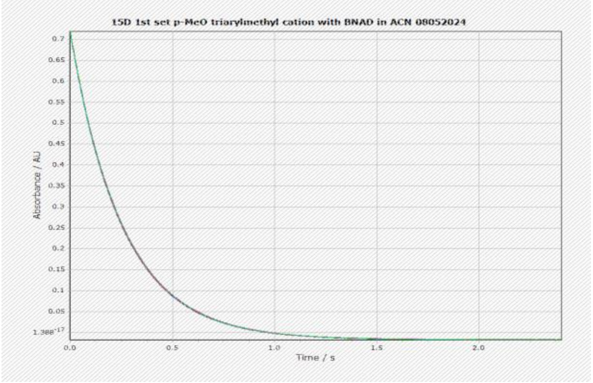

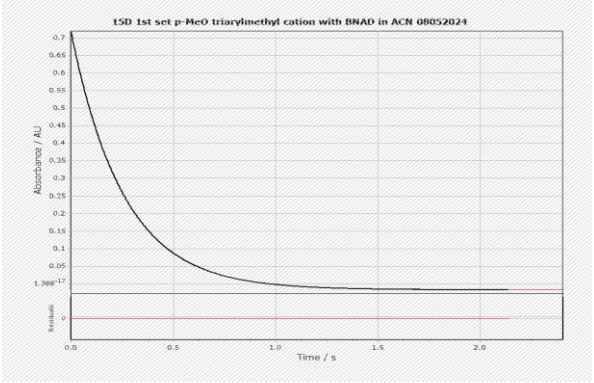

15°C D

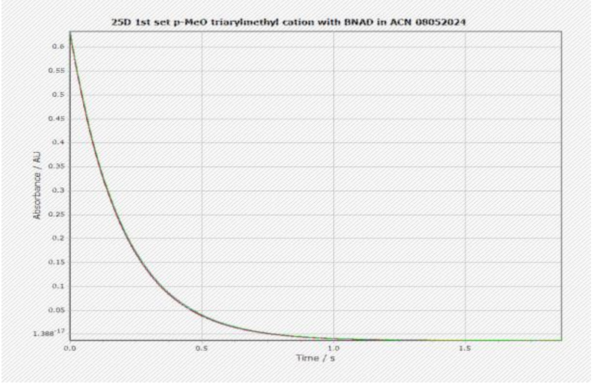

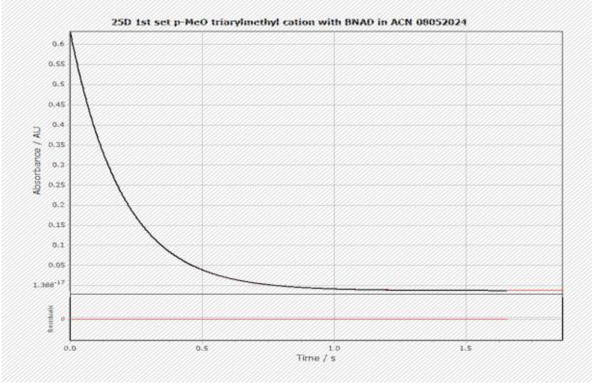

25°C D

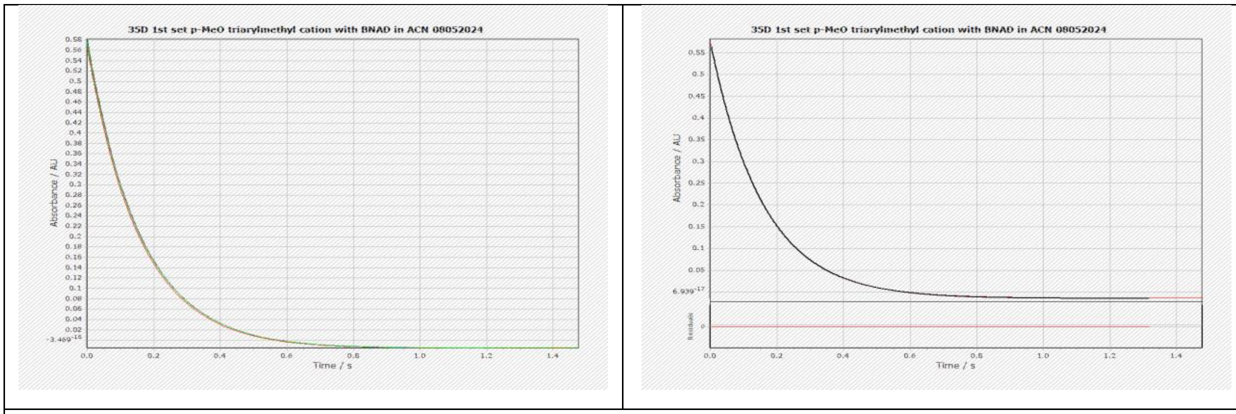

35°C D

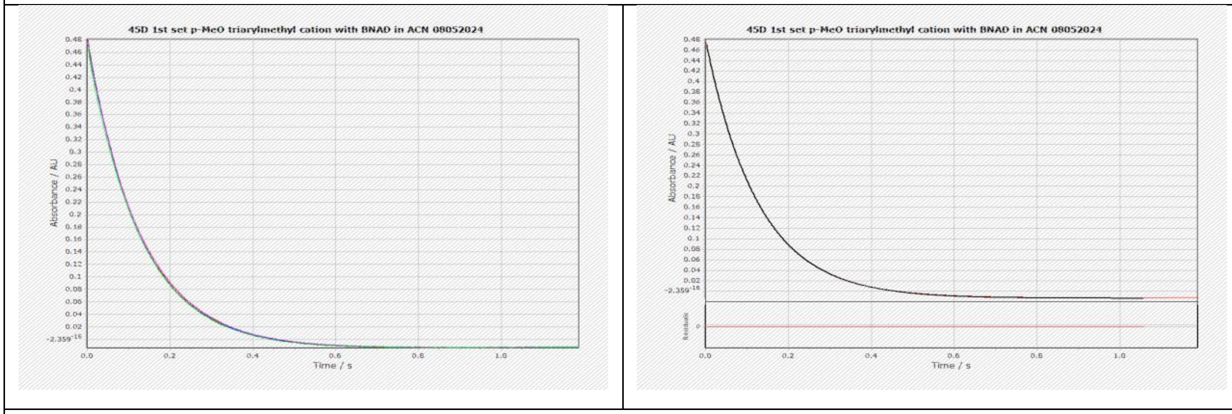

45°C D

Day 2 (2) data (August 5, 2024)

	Pseudo-first-order rate constants									
	k^{pfo} (s^{-1})									
**Temp (°C)**	**Trial H1**	**Trial H2**	**Trial H3**	**Trial H4**	**Trial H5**	**Trial H6**	**Average k_H^{pfo} (s^{-1})**	**Stdev**	**k_{2H} (M^{-1}s^{-1})**	**Stdev [a]**
45	18.7581	18.4957	18.5180	18.7367	18.5419	18.7924	18.6404	0.1355	4.79E+04	348.05
35	15.4145	15.6751	15.7372	15.8302	15.6980	15.7285	15.6806	0.1408	4.03E+04	361.37
25	12.8717	12.7833	12.7766	12.8816	12.7862	12.8304	12.8216	0.0467	3.29E+04	120.06
15	10.3712	10.3587	10.4444	10.3086	10.3798	10.3147	10.3629	0.0495	2.66E+04	127.24
5	8.2624	8.1767	8.2880	8.2499	8.2744	8.1909	8.2403	0.0458	2.12E+04	117.68
**Temp (°C)**	**Trial D1**	**Trial D2**	**Trial D3**	**Trial D4**	**Trial D5**	**Trial D6**	**Average k_D^{pfo} (s^{-1})[b]**	**Stdev**	**k_{2D} (M^{-1}s^{-1})**	**Stdev [a]**
45	7.8703	7.9944	7.8272	7.8636	7.7346	7.8861	7.4136	0.0844	1.90E+04	216.78
35	6.3906	6.3414	6.3408	6.4440	6.3761	6.4119	5.9968	0.0404	1.54E+04	103.78
25	4.9331	5.0379	5.0478	5.0094	4.9677	5.0279	4.6782	0.0447	1.20E+04	114.92
15	3.8978	3.8708	3.8892	3.9216	3.8704	3.8798	3.6185	0.0195	9.29E+03	50.05
5	2.9685	2.9762	2.9431	2.9568	2.9305	2.9404	2.7322	0.0176	7.02E+03	45.27

[a] = (Stdev(for k^{pfo})/k^{pfo})*k_2
[b] = (Average k_D^{pfo} – (0.04*Average k_H^{pfo})/0.96 due to 4% H content

<table>
<tr><td>Six kinetic runs together with the averaged data</td><td>First-order kinetic fit of the averaged data as an example</td></tr>
<tr><td>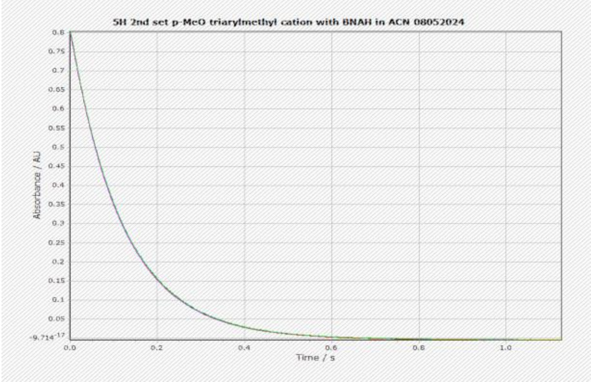
</td><td>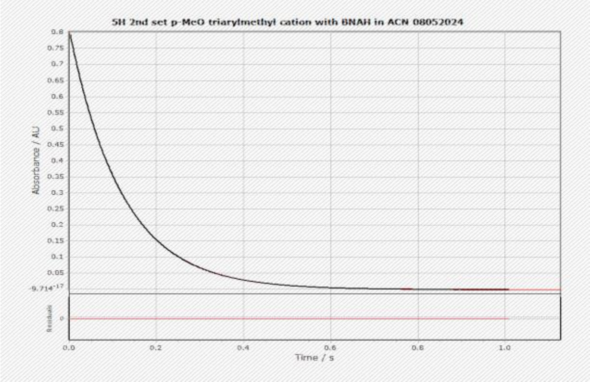
</td></tr>
<tr><td colspan="2">5℃ H</td></tr>
<tr><td>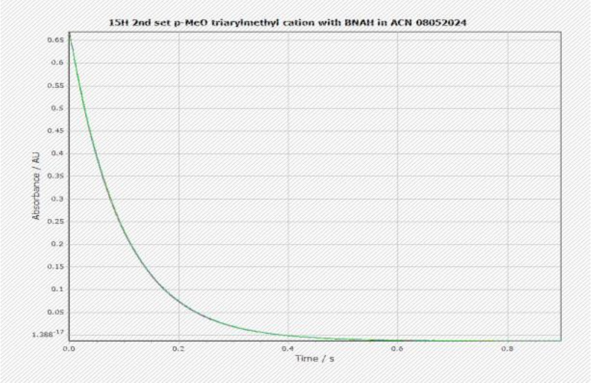
</td><td>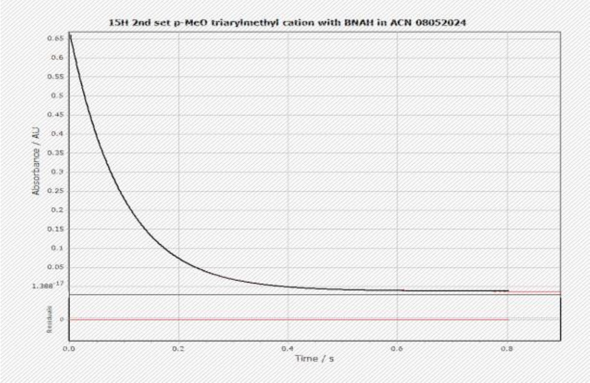
</td></tr>
<tr><td colspan="2">15℃ H</td></tr>
<tr><td>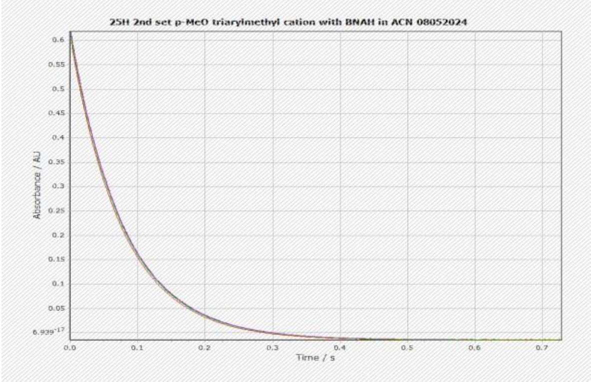
</td><td>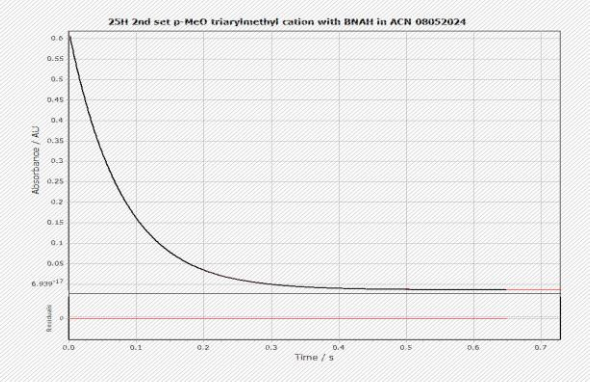
</td></tr>
<tr><td colspan="2">25℃ H</td></tr>
</table>

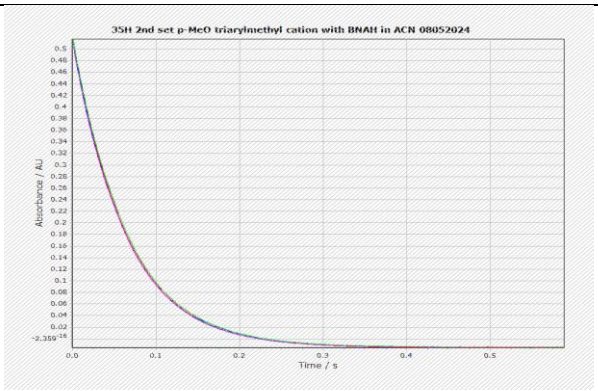
35H 2nd set p-MeO triarylmethyl cation with BNAH in ACN 08052024
Absorbance / AU
Time / s

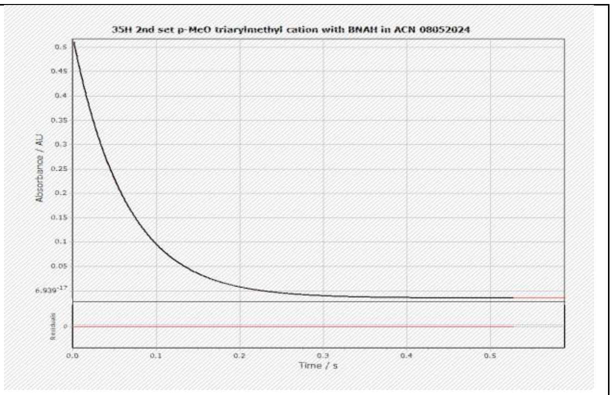
35H 2nd set p-MeO triarylmethyl cation with BNAH in ACN 08052024
Absorbance / AU
Residuals
Time / s

35℃ H

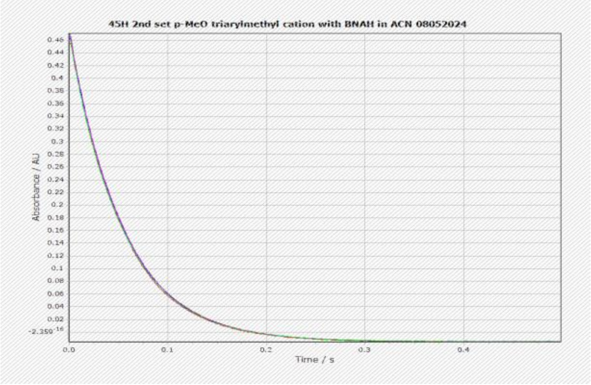
45H 2nd set p-MeO triarylmethyl cation with BNAH in ACN 08052024
Absorbance / AU
Time / s

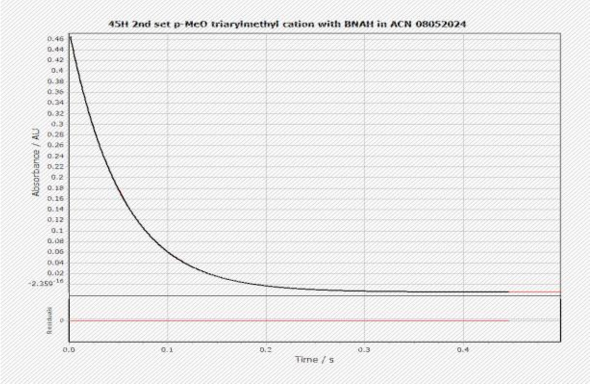
45H 2nd set p-MeO triarylmethyl cation with BNAH in ACN 08052024
Absorbance / AU
Residuals
Time / s

45℃ H

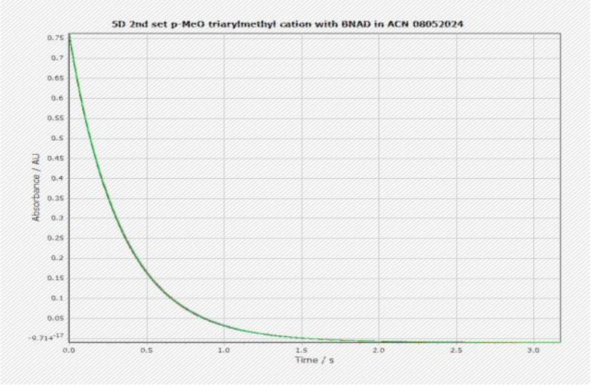
5D 2nd set p-MeO triarylmethyl cation with BNAD in ACN 08052024
Absorbance / AU
Time / s

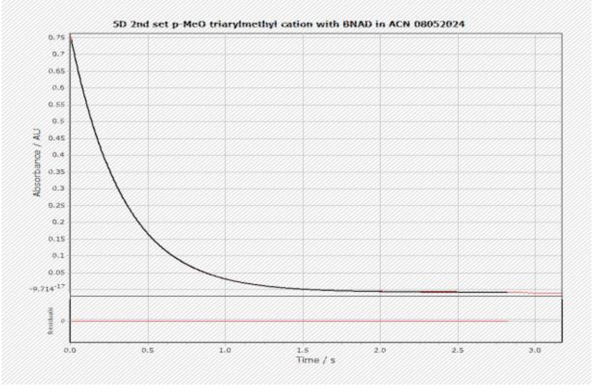
5D 2nd set p-MeO triarylmethyl cation with BNAD in ACN 08052024
Absorbance / AU
Residuals
Time / s

5℃ D

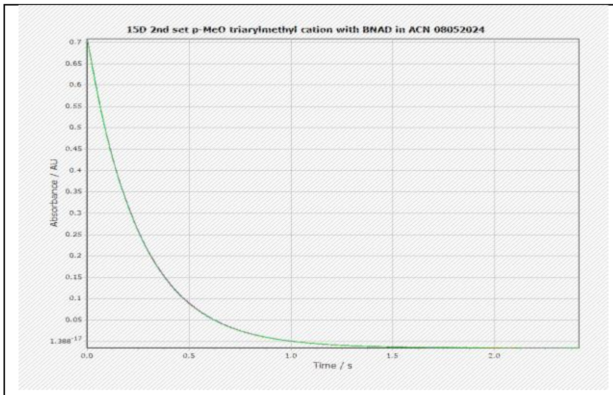

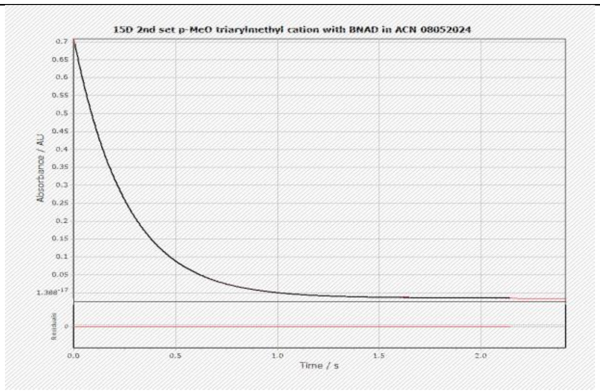

15°C D

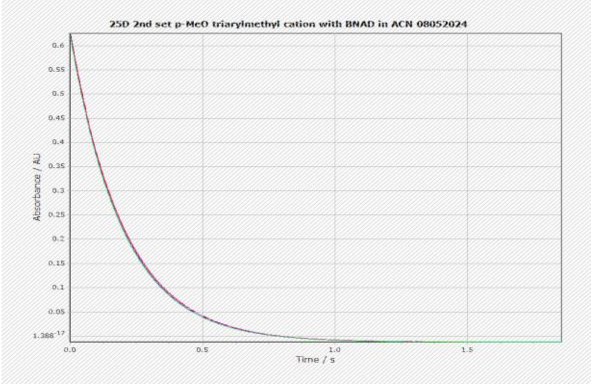

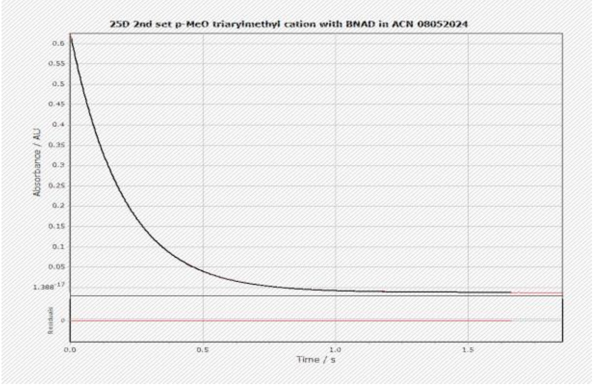

25°C D

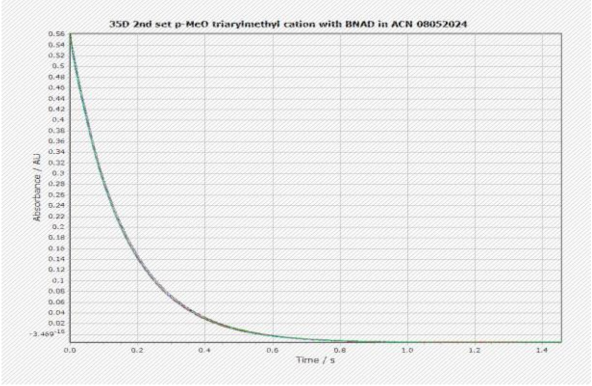

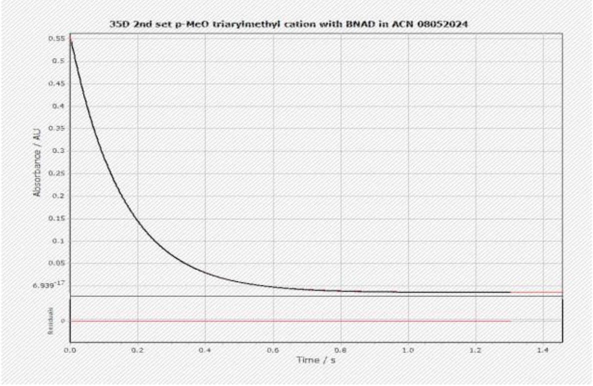

35°C D

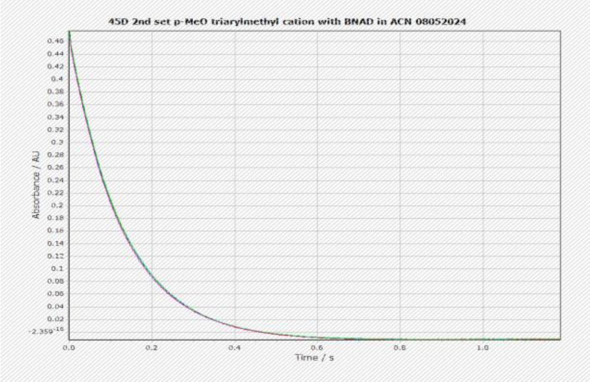	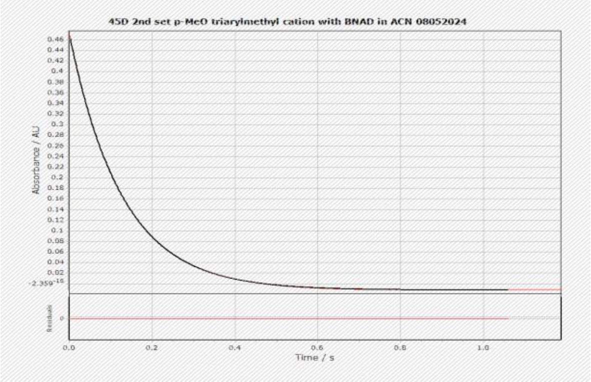
45°C D	

Primary kinetic data for the rate constants in Table S6 (HEH to $(CH_3OPh)_3C^+BF_4^-$)

Day 1 data (July 19, 2024)

	Pseudo-first-order rate constants k_H^{pfo} (s^{-1})									
**Temp (°C)**	**Trial H1**	**Trial H2**	**Trial H3**	**Trial H4**	**Trial H5**	**Trial H6**	**Average k_H^{pfo} (s^{-1})**	**Stdev**	**k_{2H} ($M^{-1}s^{-1}$)**	**Stdev [a]**
45	12.0682	11.9567	11.9373	11.9297	12.0208	12.0197	11.9887	0.0556	1.03E+04	47.61
35	8.9639	9.0033	9.1014	8.9756	9.1286	8.9972	9.0283	0.0692	7.73E+03	59.22
25	6.7549	6.7674	6.7895	6.7909	6.7855	6.7261	6.7691	0.0253	5.80E+03	21.70
15	4.9022	4.9414	4.9473	4.9317	4.8989	4.9049	4.9211	0.0216	4.21E+03	18.46
5	3.4541	3.4517	3.4584	3.4613	3.4486	3.4679	3.4570	0.0070	2.96E+03	6.01
**Temp (°C)**	**Trial D1**	**Trial D2**	**Trial D3**	**Trial D4**	**Trial D5**	**Trial D6**	**Average k_D^{pfo} (s^{-1})**	**Stdev**	**k_{2D} ($M^{-1}s^{-1}$)**	**Stdev [a]**
45	3.5803	3.5817	3.5749	3.5946	3.5885	3.5942	3.5857	0.0080	3.07E+03	6.88
35	2.5565	2.5283	2.5557	2.5758	2.5633	2.5698	2.5582	0.0166	2.19E+03	14.18
25	1.8471	1.8163	1.8184	1.8282	1.8266	1.8150	1.8253	0.0120	1.56E+03	10.26
15	1.2523	1.2566	1.2548	1.2622	1.2591	1.2506	1.2559	0.0043	1.08E+03	3.68
5	0.8435	0.8493	0.8467	0.8485	0.8470	0.8415	0.8461	0.0030	7.24E+02	2.57

[a] = (Stdev(for k^{pfo})/k^{pfo})*k_2

Six kinetic runs together with the averaged data	First-order kinetic fit of the averaged data as an example
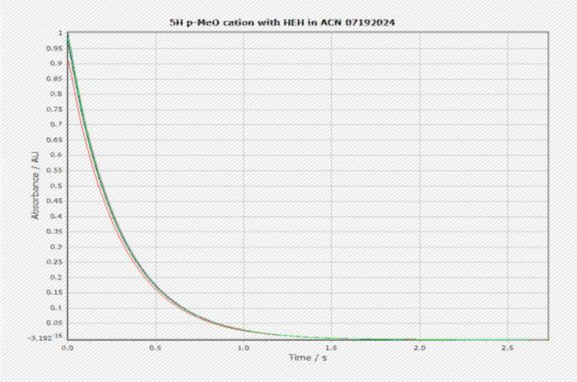	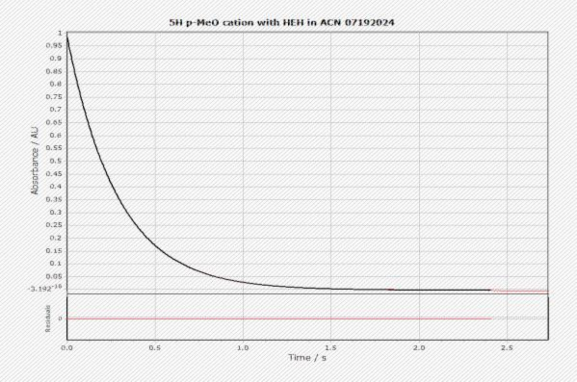
5°C H	

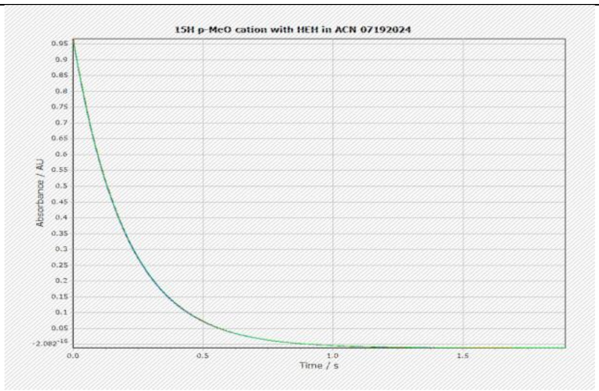

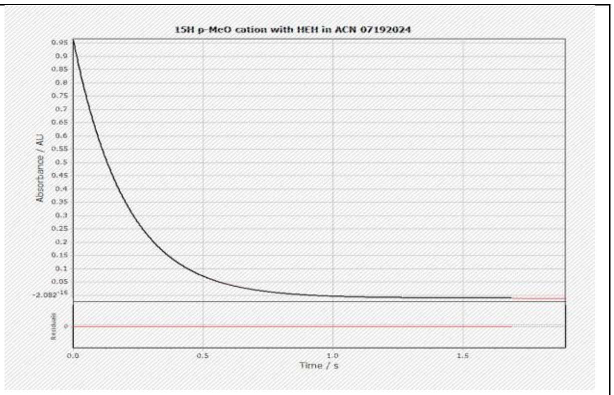

15°C H

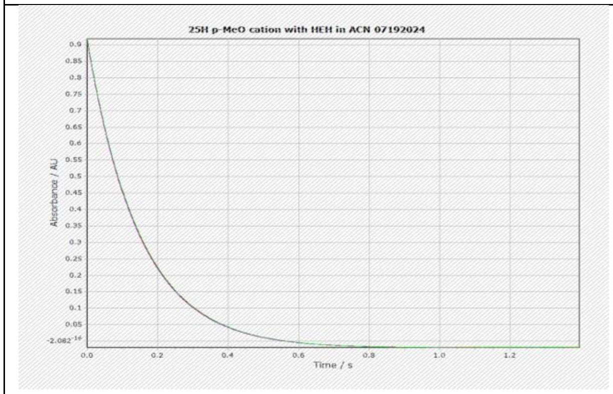

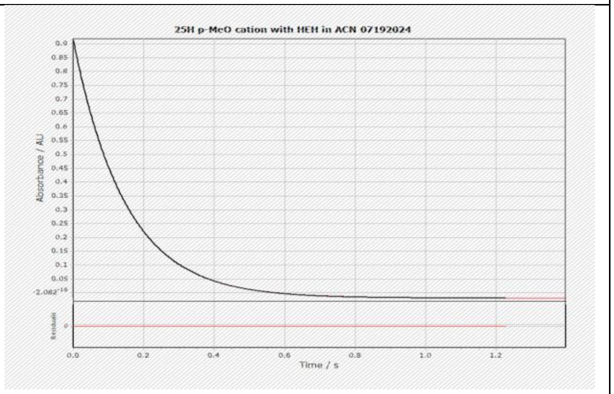

25°C H

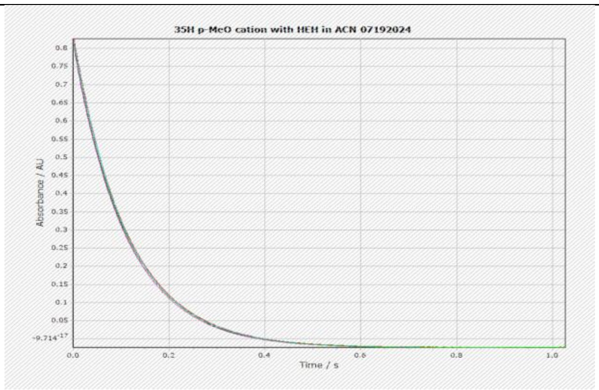

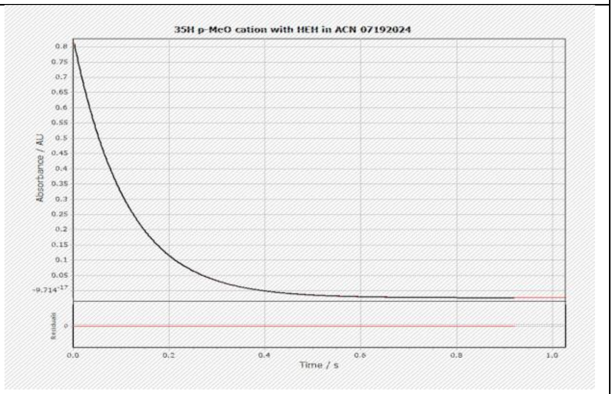

35°C H

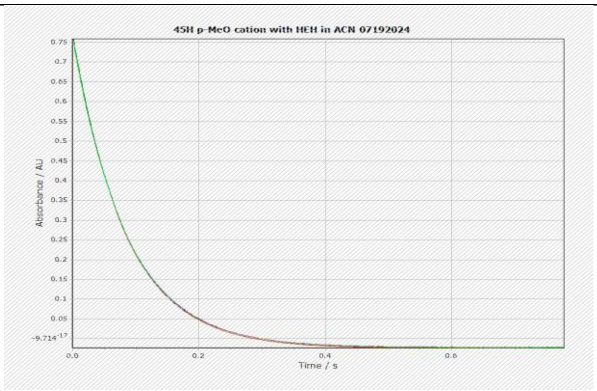
45H p-MeO cation with HEH in ACN 07192024
Absorbance / AU
Time / s

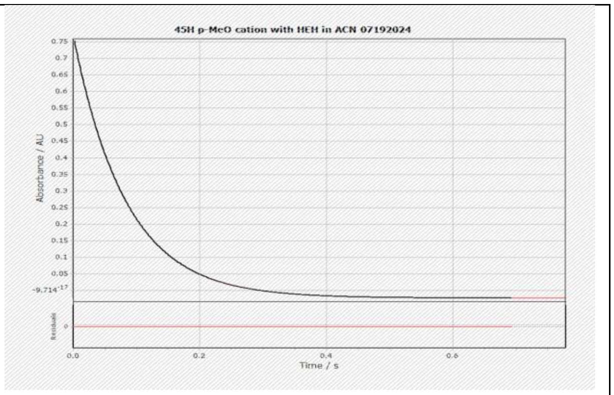
45H p-MeO cation with HEH in ACN 07192024
Absorbance / AU
Residuals
Time / s

45℃ H

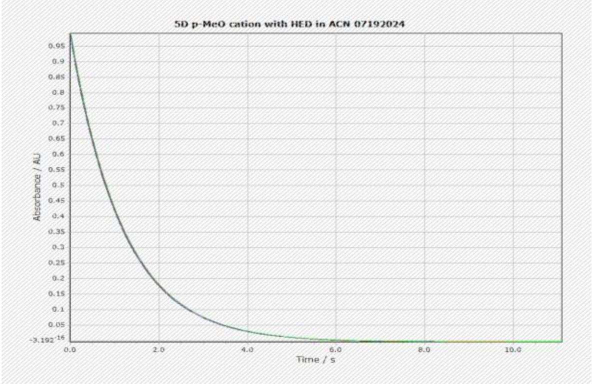
5D p-MeO cation with HED in ACN 07192024
Absorbance / AU
Time / s

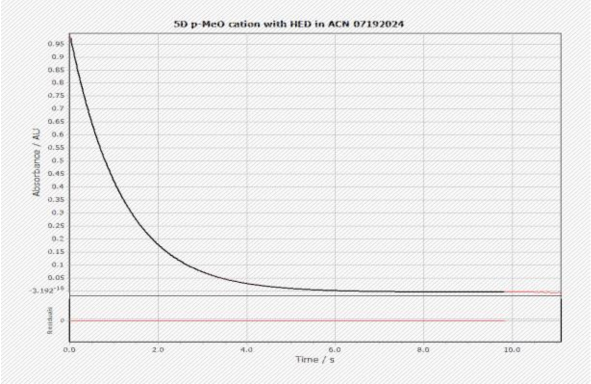
5D p-MeO cation with HED in ACN 07192024
Absorbance / AU
Residuals
Time / s

5℃ D

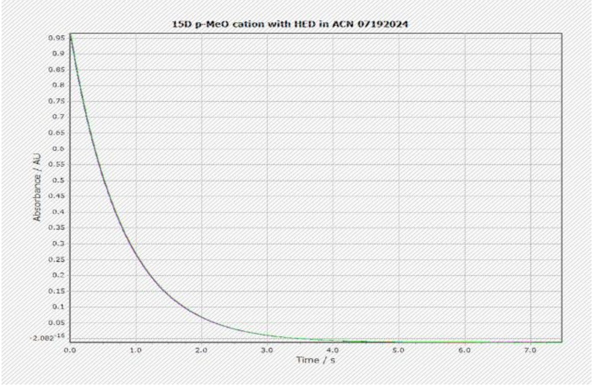
15D p-MeO cation with HED in ACN 07192024
Absorbance / AU
Time / s

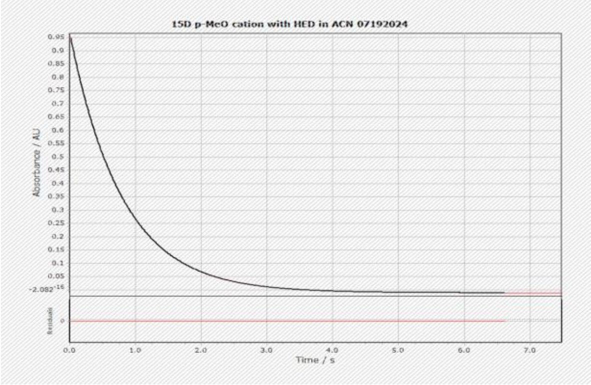
15D p-MeO cation with HED in ACN 07192024
Absorbance / AU
Residuals
Time / s

15℃ D

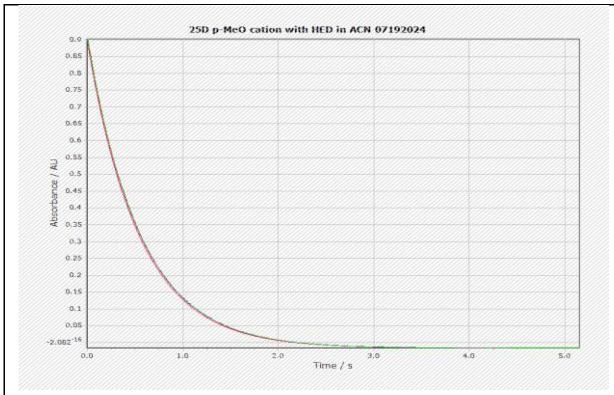

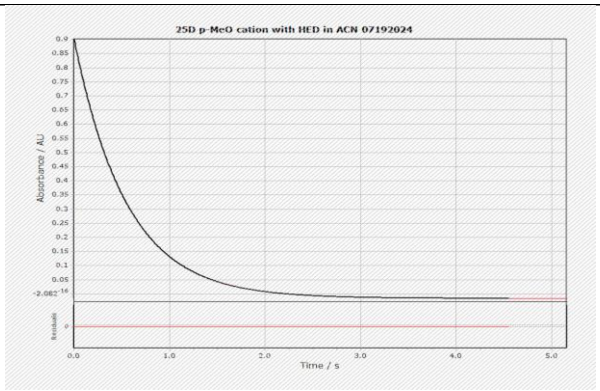

25°C D

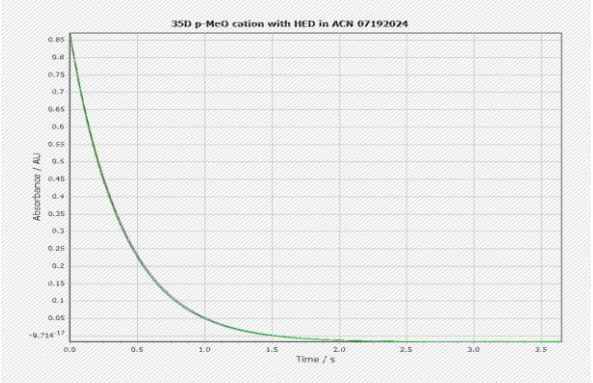

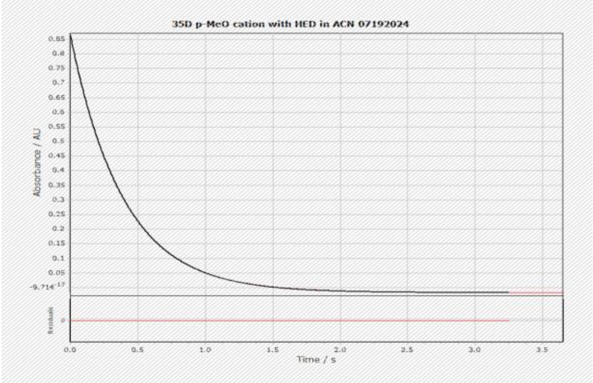

35°C D

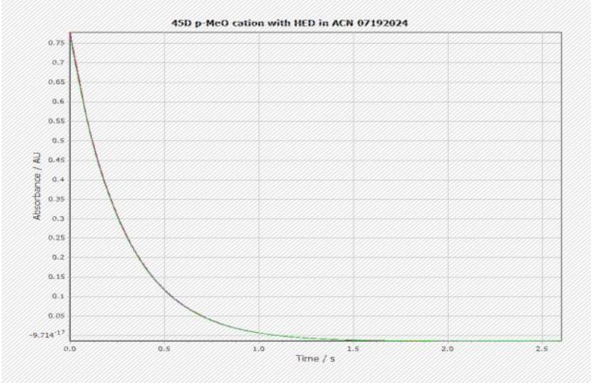

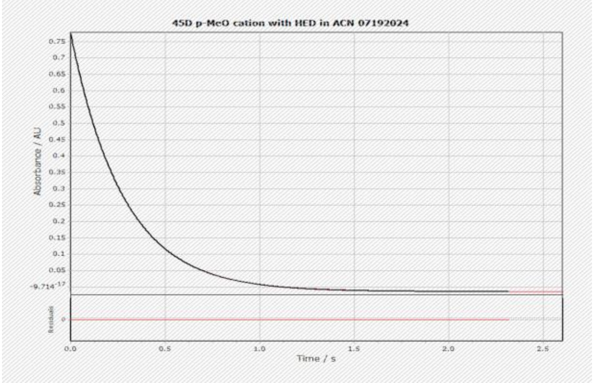

45°C D

Day 2 data (July 25, 2024)

	Pseudo-first-order rate constants k_H^{pfo} (s^{-1})									
**Temp (°C)**	**Trial H1**	**Trial H2**	**Trial H3**	**Trial H4**	**Trial H5**	**Trial H6**	**Average k_H^{pfo} (s^{-1})**	**Stdev**	**k_{2H} ($M^{-1}s^{-1}$)**	**Stdev [a]**
45	11.2161	11.4366	11.4140	11.2679	11.2576	11.3160	11.3180	0.0892	9.69E+03	76.39
35	8.6258	8.5739	8.6519	8.6510	8.6374	8.6090	8.6248	0.0297	7.38E+03	25.46
25	6.4658	6.4390	6.5146	6.4570	6.4231	6.4976	6.4662	0.0347	5.54E+03	29.68
15	4.6036	4.6381	4.6826	4.7132	4.6629	4.6513	4.6586	0.0376	3.99E+03	32.19
5	3.2335	3.2471	3.2359	3.2728	3.2705	3.3209	3.2634	0.0328	2.79E+03	28.05

Temp (°C)	Trial D1	Trial D2	Trial D3	Trial D4	Trial D5	Trial D6	Average k_D^{pfo} (s^{-1})	Stdev	k_{2D} ($M^{-1}s^{-1}$)	Stdev [a]
45	3.5148	3.4708	3.5295	3.4756	3.4956	3.5071	3.4989	0.0228	3.00E+03	19.54
35	2.5156	2.5379	2.5355	2.5059	2.5107	2.5275	2.5222	0.0134	2.16E+03	11.45
25	1.7959	1.7751	1.7872	1.7857	1.7855	1.8063	1.7893	0.0106	1.53E+03	9.09
15	1.2341	1.2250	1.2355	1.2230	1.2357	1.2493	1.2338	0.0094	1.06E+03	8.05
5	0.8309	0.8229	0.8277	0.8269	0.8268	0.8295	0.8274	0.0028	7.08E+02	2.35

[a] = (Stdev(for k^{pfo})/k^{pfo})*k_2

Six kinetic runs together with the averaged data

First-order kinetic fit of the averaged data as an example

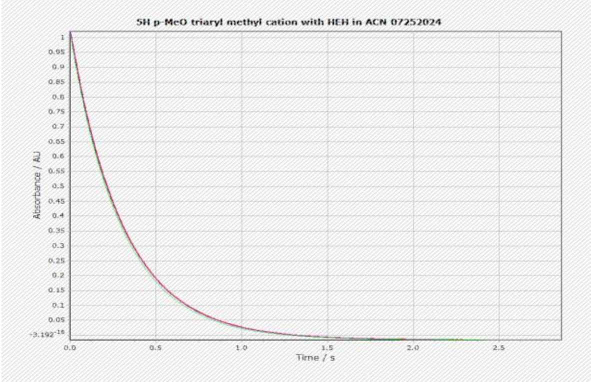

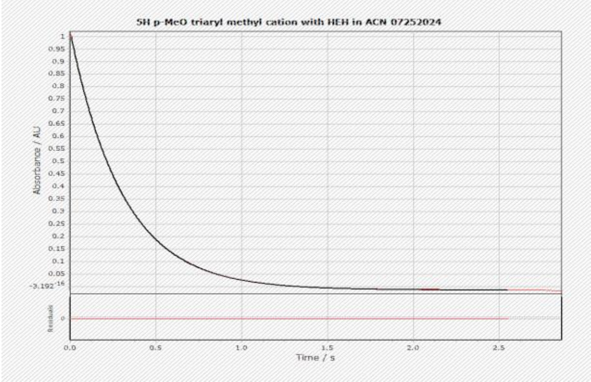

5°C H

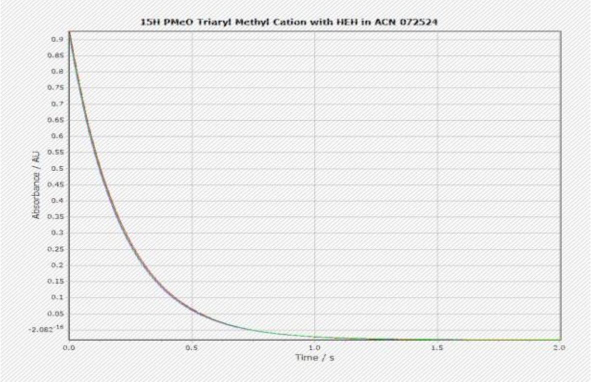

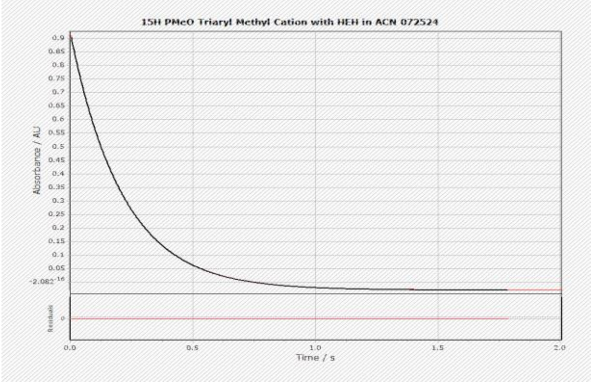

15°C H

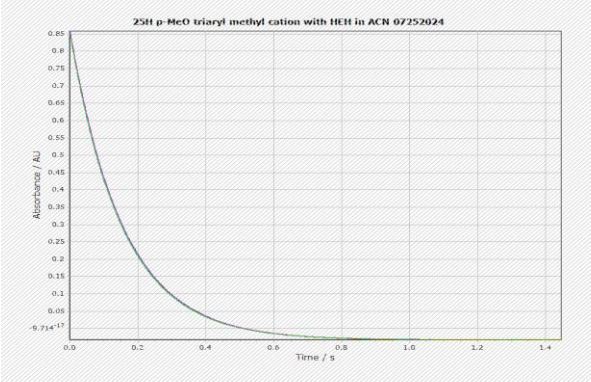

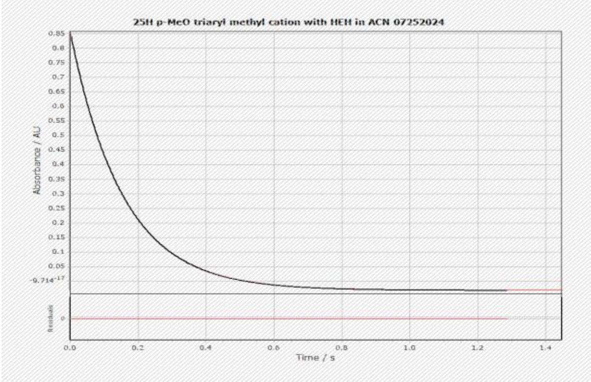

25°C H

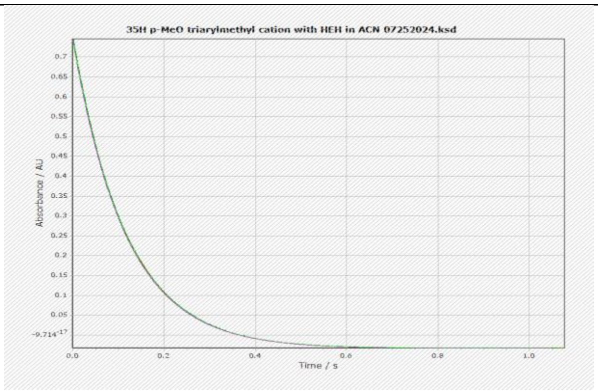
35H p-MeO triarylmethyl cation with HEH in ACN 07252024.ksd
Absorbance / AU
Time / s
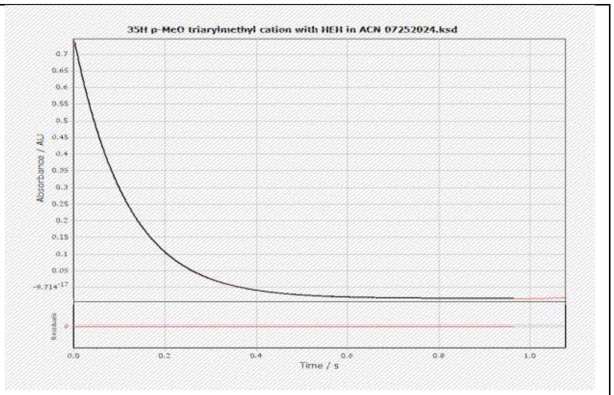
35H p-MeO triarylmethyl cation with HEH in ACN 07252024.ksd
Absorbance / AU
Residuals
Time / s

35℃ H

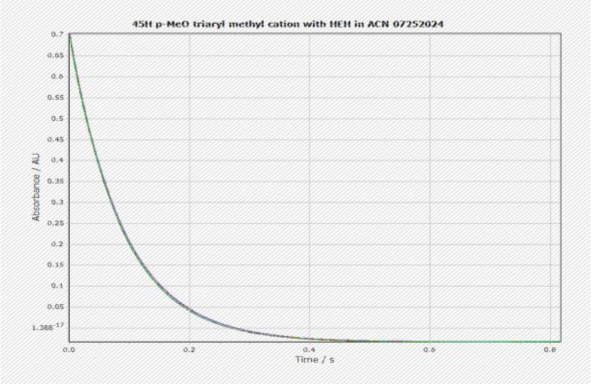
45H p-MeO triaryl methyl cation with HEH in ACN 07252024
Absorbance / AU
Time / s
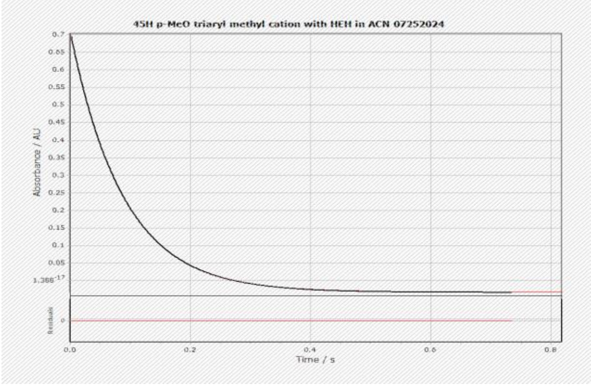
45H p-MeO triaryl methyl cation with HEH in ACN 07252024
Absorbance / AU
Residuals
Time / s

45℃ H

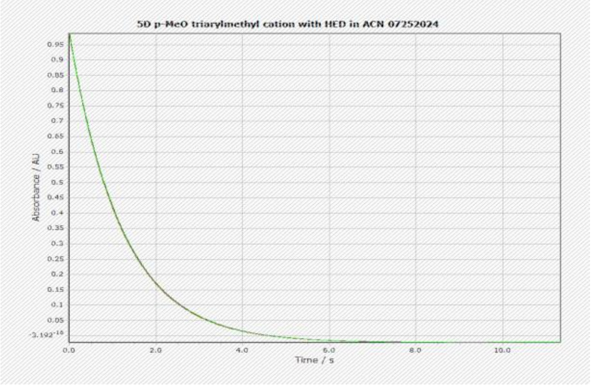
5D p-MeO triarylmethyl cation with HED in ACN 07252024
Absorbance / AU
Time / s
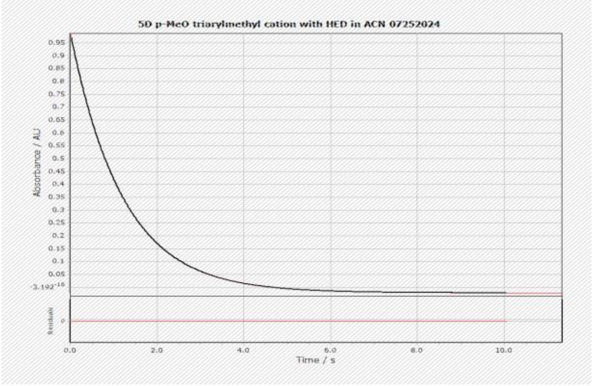
5D p-MeO triarylmethyl cation with HED in ACN 07252024
Absorbance / AU
Residuals
Time / s

5℃ D

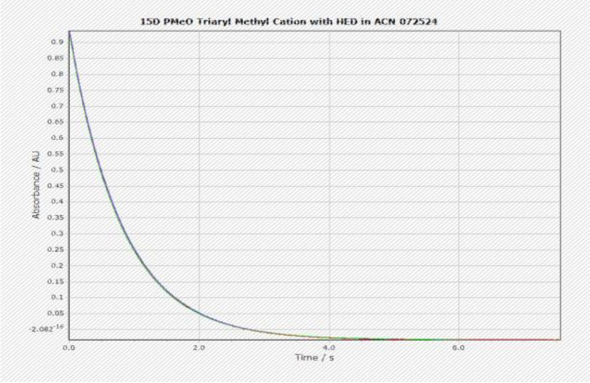	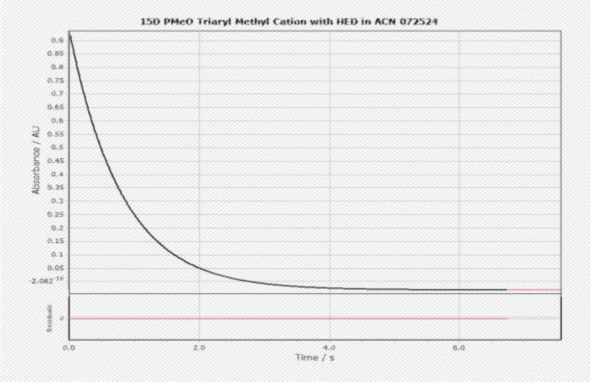
15°C D	
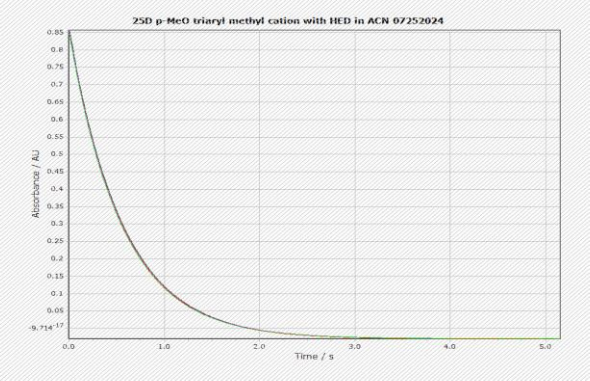	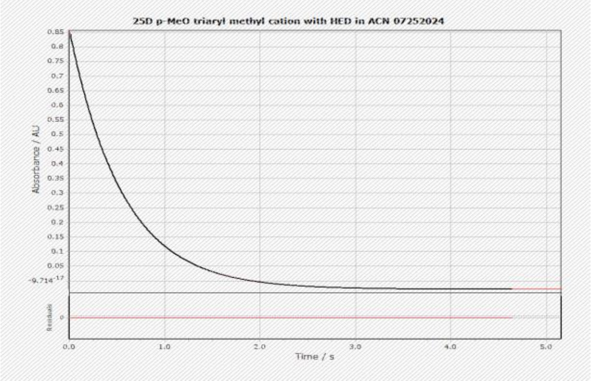
25°C D	
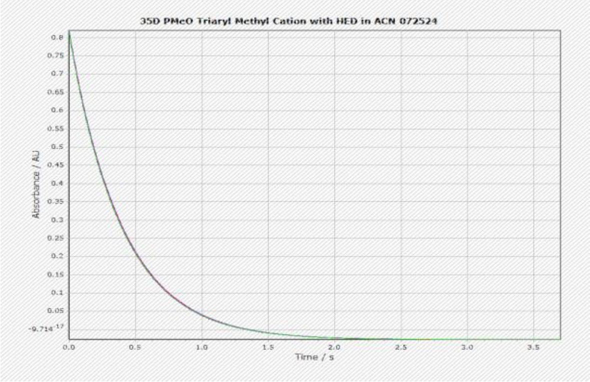	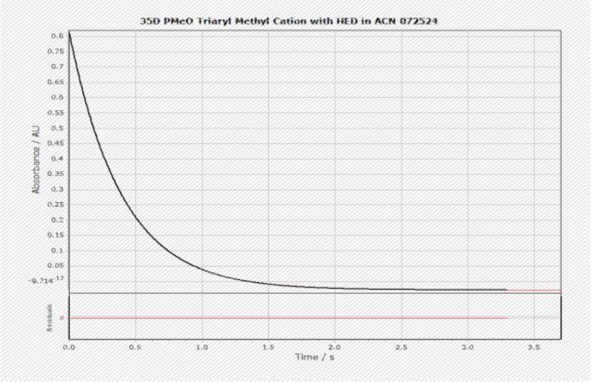
35°C D	

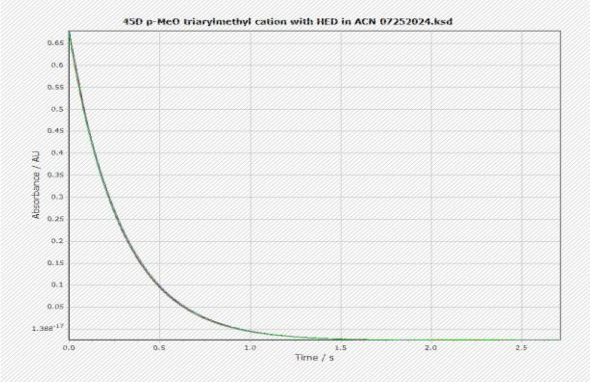

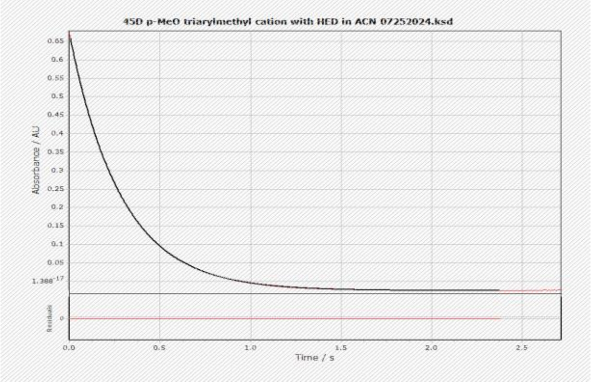

45°C D

Day 3 data (July 30, 2024)

	Pseudo-first-order rate constants k_H^{pfo} (s^{-1})									
**Temp (°C)**	**Trial H1**	**Trial H2**	**Trial H3**	**Trial H4**	**Trial H5**	**Trial H6**	**Average k_H^{pfo} (s^{-1})**	**Stdev**	**k_{2H} (M^{-1}s^{-1})**	**Stdev [a]**
45	11.5605	11.5920	11.5489	11.5569	11.7754	11.7362	11.6283	0.1006	9.96E+03	86.13
35	9.1768	9.0506	8.9406	9.0894	9.1476	9.1197	9.0874	0.0843	7.78E+03	72.19
25	6.4831	6.5703	6.6330	6.6231	6.5921	6.5991	6.5834	0.0540	5.64E+03	46.23
15	4.7923	4.6957	4.7002	4.7967	4.7572	4.7165	4.7431	0.0454	4.06E+03	38.84
5	3.3301	3.3318	3.3343	3.3105	3.3372	3.3142	3.3264	0.0112	2.85E+03	9.58
**Temp (°C)**	**Trial D1**	**Trial D2**	**Trial D3**	**Trial D4**	**Trial D5**	**Trial D6**	**Average k_D^{pfo} (s^{-1})**	**Stdev**	**k_{2D} (M^{-1}s^{-1})**	**Stdev [a]**
45	3.6293	3.6184	3.5973	3.6163	3.6307	3.6371	3.6215	0.0142	3.10E+03	12.17
35	2.6306	2.5956	2.6559	2.6258	2.6586	2.6197	2.6310	0.0236	2.25E+03	20.23
25	1.8365	1.8468	1.8254	1.8165	1.8449	1.8267	1.8328	0.0120	1.57E+03	10.23
15	1.2610	1.2443	1.2614	1.2513	1.2510	1.2474	1.2527	0.0070	1.07E+03	6.03
5	0.8378	0.8387	0.8411	0.8398	0.8444	0.8368	0.8398	0.0027	7.19E+02	2.35

[a] = (Stdev(for k^{pfo})/k^{pfo})*k_2

Six kinetic runs together with the averaged data	First-order kinetic fit of the averaged data as an example
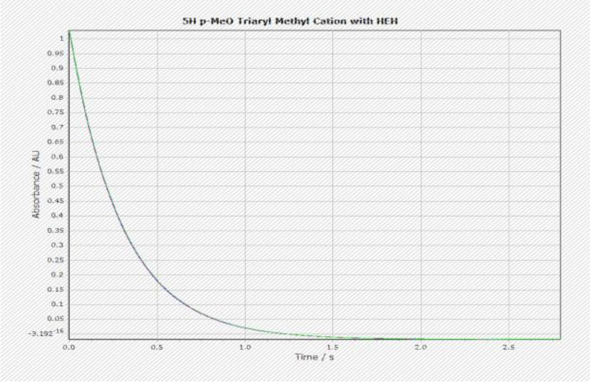	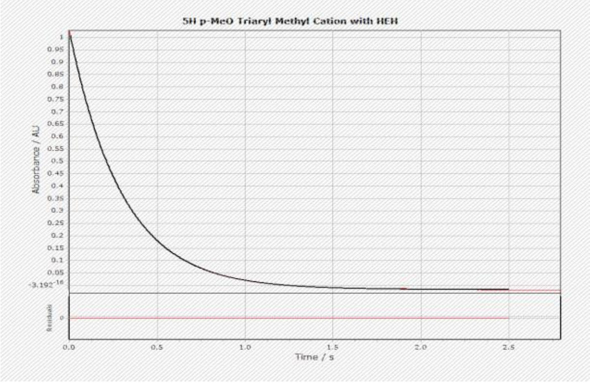

5°C H

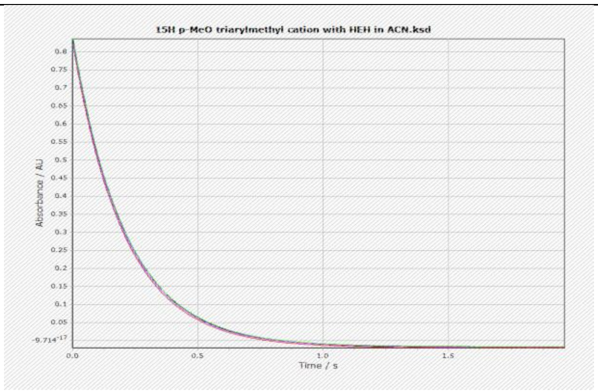

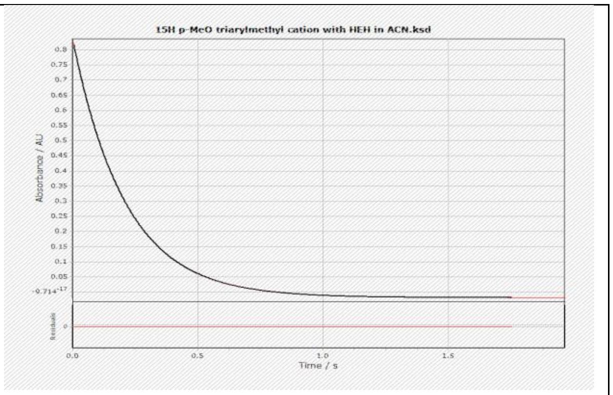

15°C H

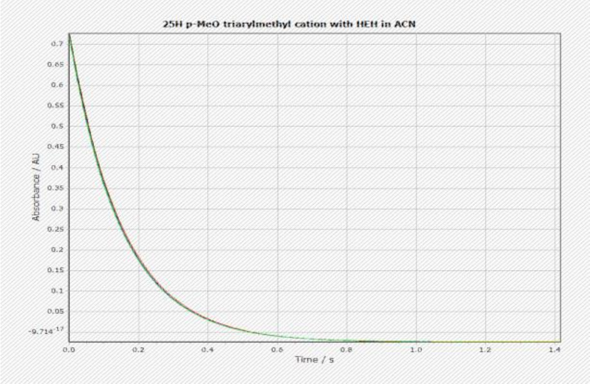

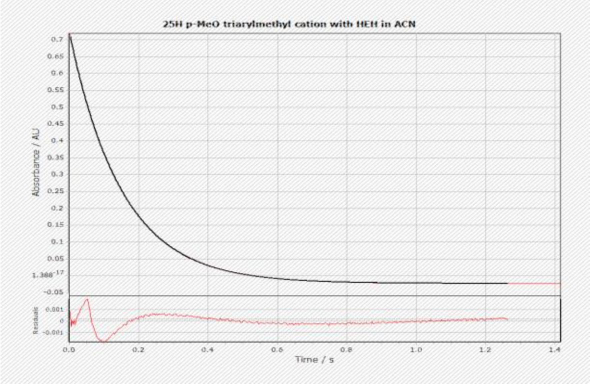

25°C H

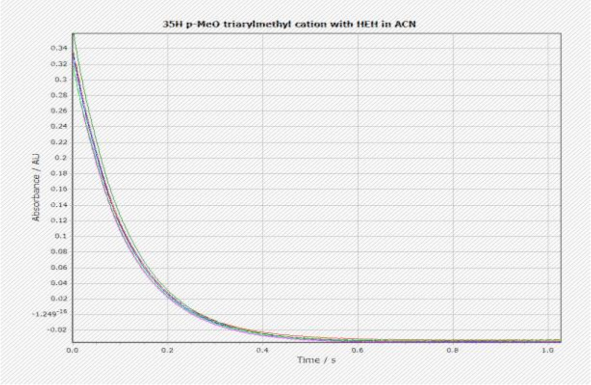

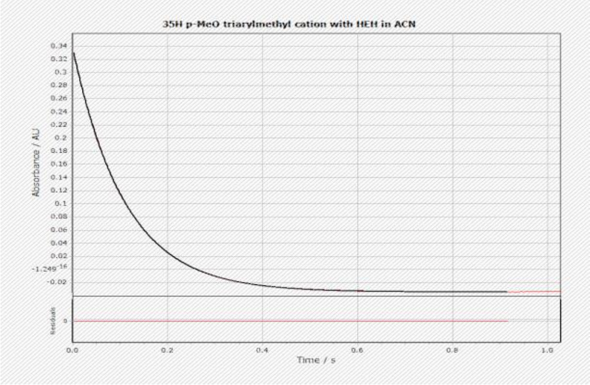

35°C H

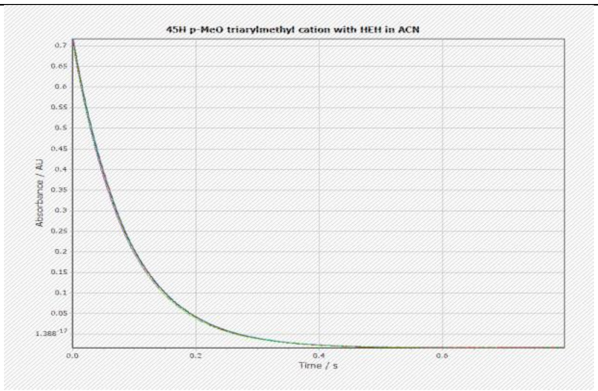

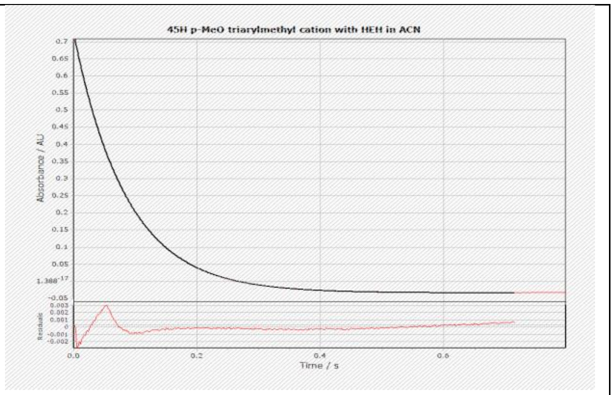

45°C H

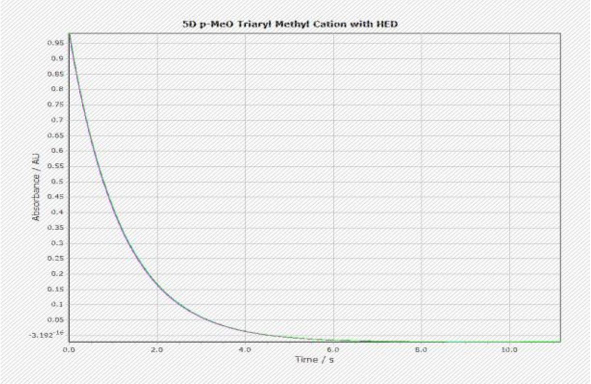

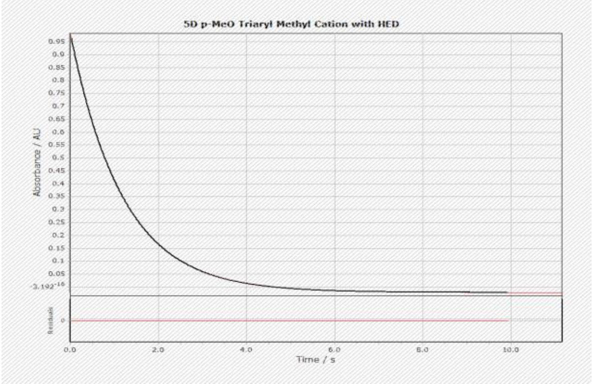

5°C D

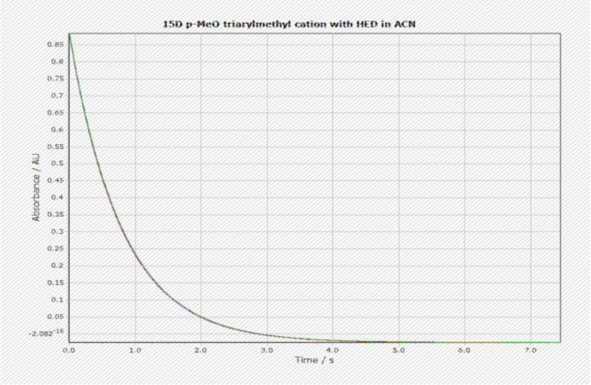

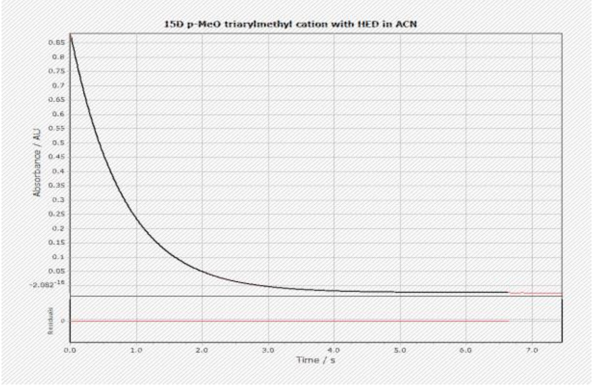

15°C D

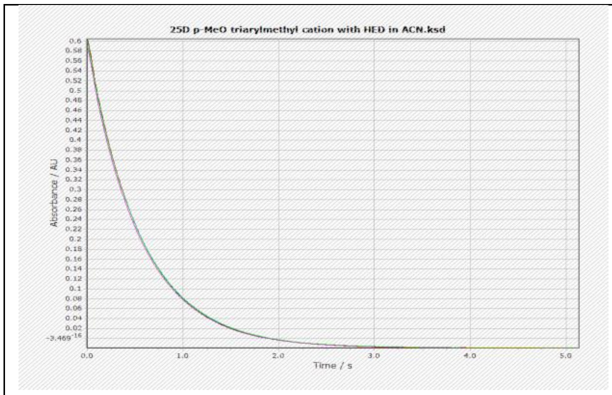	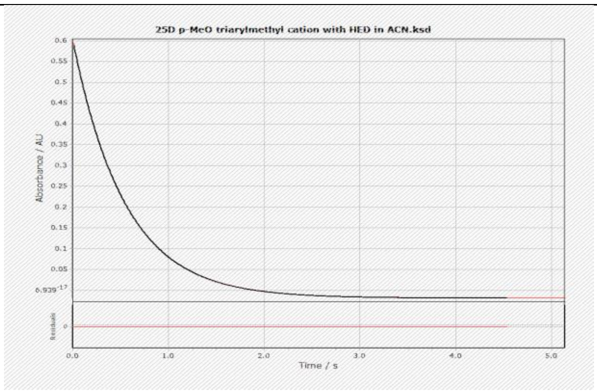
25°C D	
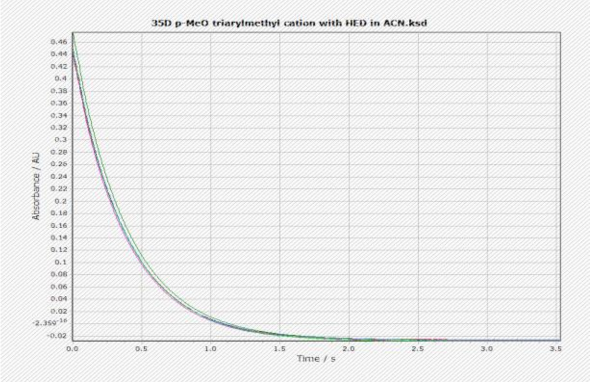	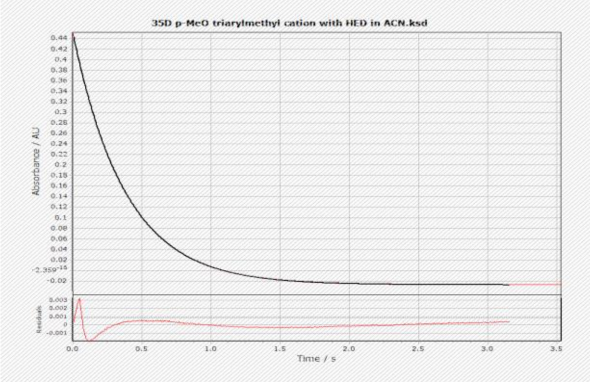
35°C D	
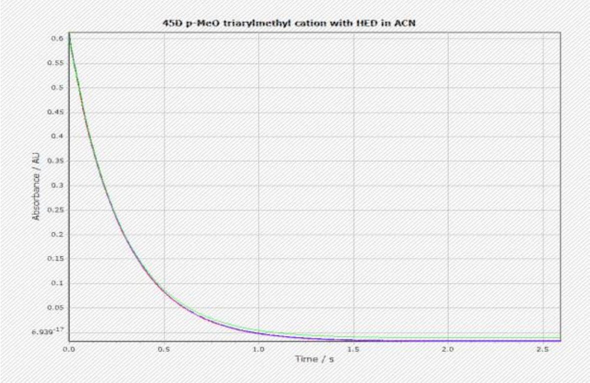	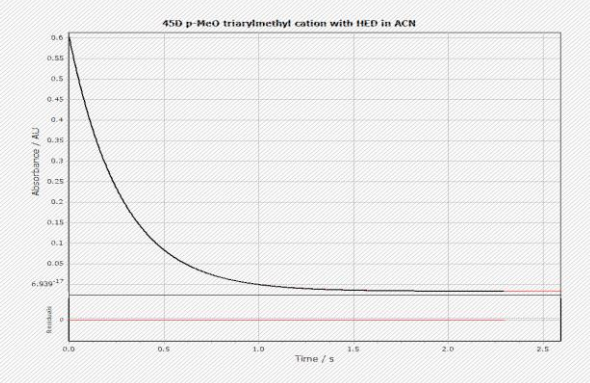
45°C D	

Primary kinetic data for the rate constants in Table S7 (BNAH with $MA^+BF_4^-$)

Day 1 data (February 05, 2024)

	Pseudo-first-order rate constants k^{pfo} (s^{-1})									
**Temp (°C)**	**Trial H1**	**Trial H2**	**Trial H3**	**Trial H4**	**Trial H5**	**Trial H6**	**Average k_H^{pfo} (s^{-1})**	**Stdev**	**k_{2H} ($M^{-1}s^{-1}$)**	**Stdev [a]**
45	0.44398	0.4374	0.43681	0.44213	0.4423	0.45027	0.4421	4.91E-03	1.47E+02	1.64E+00
35	0.31424	0.3099	0.31111	0.30979	0.31495	0.31327	0.3122	2.24E-03	1.04E+02	7.48E-01
25	0.22045	0.22063	0.21478	0.2156	0.21543	0.2183	0.2175	2.62E-03	7.25E+01	8.75E-01
15	0.15045	0.15172	0.15463	0.15085	0.15251	0.15008	0.1517	1.68E-03	5.06E+01	5.61E-01
5	0.10861	0.10775	0.10832	0.10962	0.10759	0.10763	0.1083	7.85E-04	3.61E+01	2.62E-01

Temp (°C)	Trial D1	Trial D2	Trial D3	Trial D4	Trial D5	Trial D6	Average k_D^{pfo} (s-1) [b]	Stdev	k_{2D} (M-1s-1)	Stdev [a]
45	0.12865	0.12781	0.12761	0.1273	0.12617	0.12847	0.1146	8.96E-04	3.82E+01	2.68E-01
35	0.08582	0.08396	0.08428	0.08623	0.08528	0.08496	0.0756	8.74E-04	2.52E+01	2.59E-01
25	0.05769	0.05687	0.05692	0.05663	0.05712	0.05659	0.0503	4.03E-04	1.68E+01	1.19E-01
15	0.03718	0.03748	0.03702	0.03683	0.037	0.03749	0.0324	2.70E-04	1.08E+01	7.86E-02
5	0.02554	0.02486	0.02506	0.02542	0.02465	0.02513	0.0216	3.34E-04	7.22E+00	9.60E-02

[a] = (Stdev(for k^{pfo})/k^{pfo})*k_2
[b] = (Average k_D^{pfo} – (0.04*Average k_H^{pfo})/0.96 due to 4% H content

Six kinetic trials and the mean fit	First-order kinetic fitted average of six trials
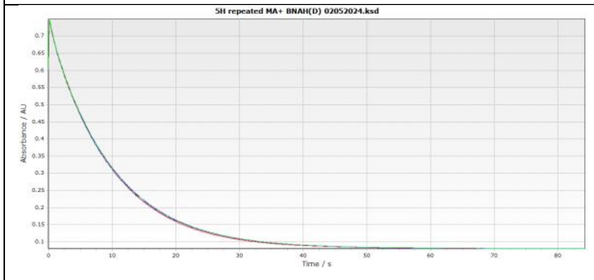	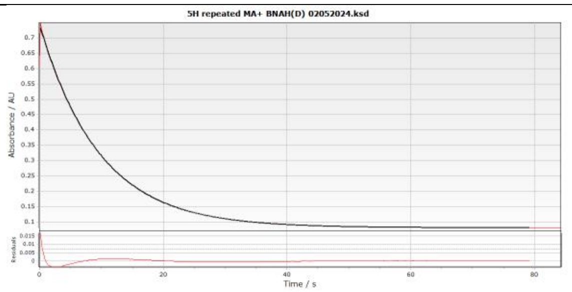
5°C H	
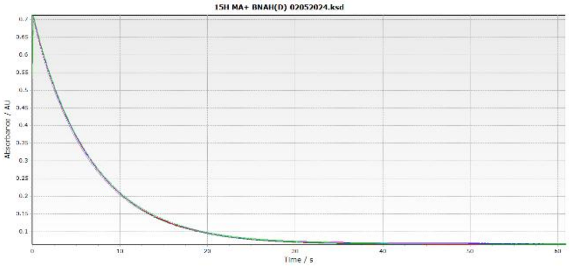	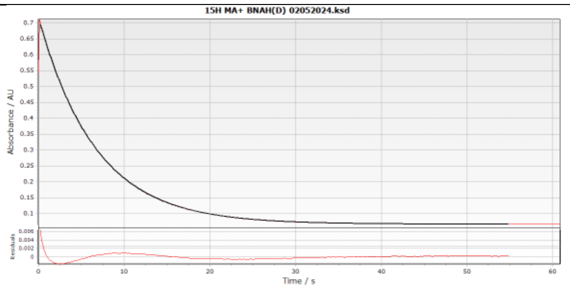
15°C H	
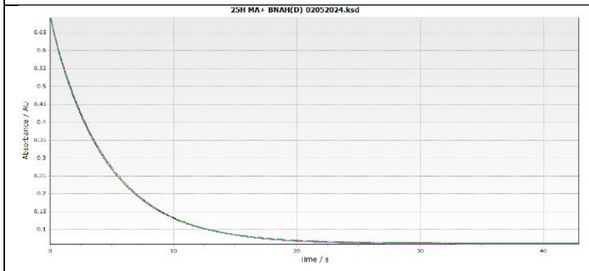	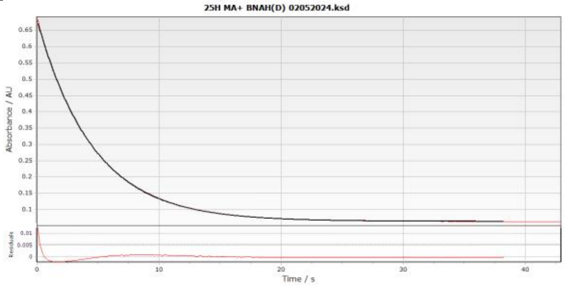
25°C H	
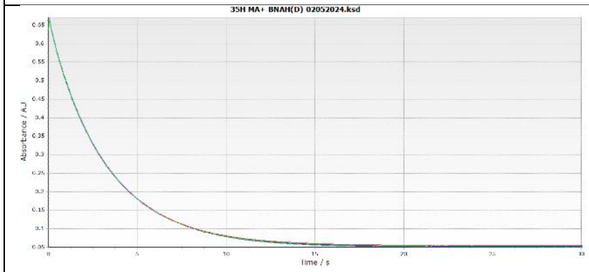	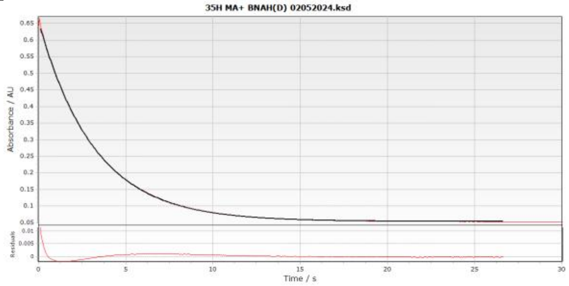
35°C H	

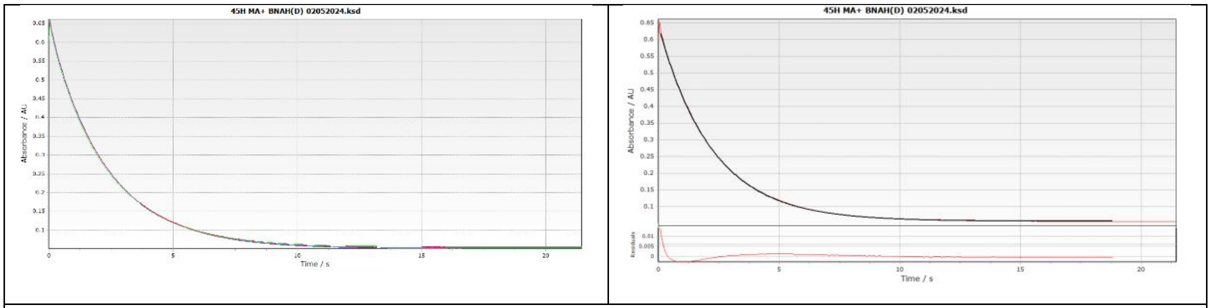

45℃ H

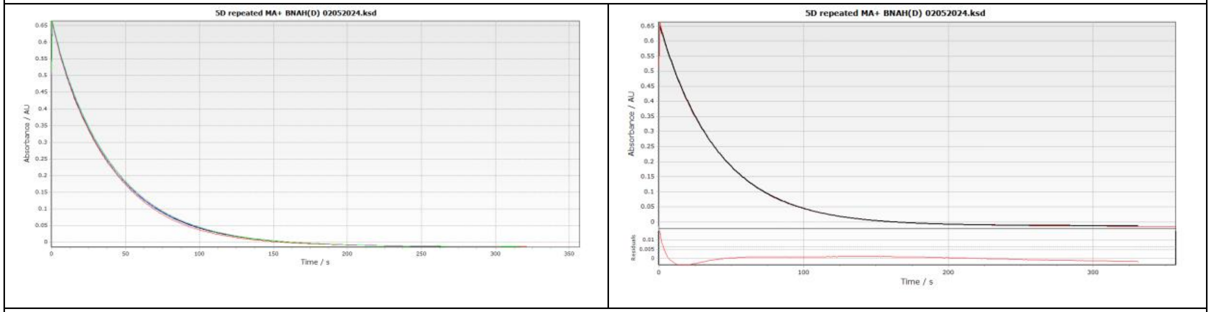

5℃ D

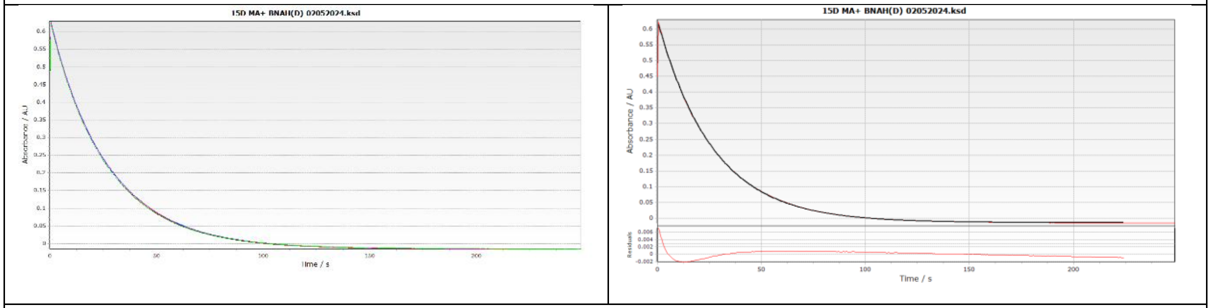

15℃ D

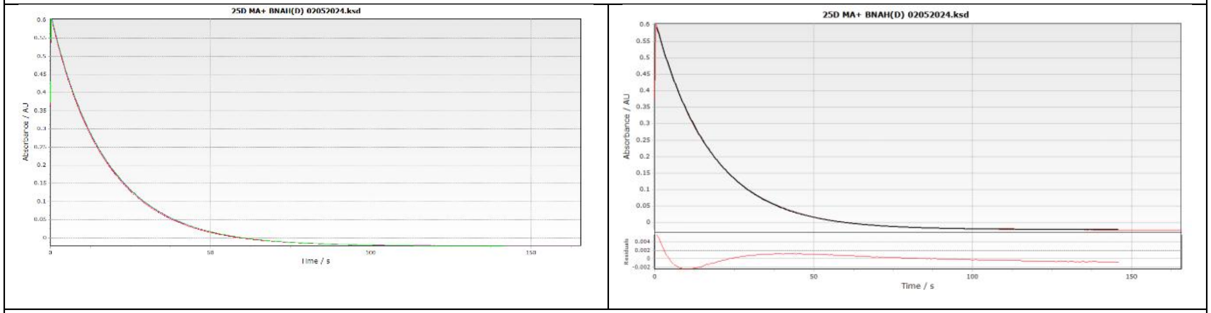

25℃ D

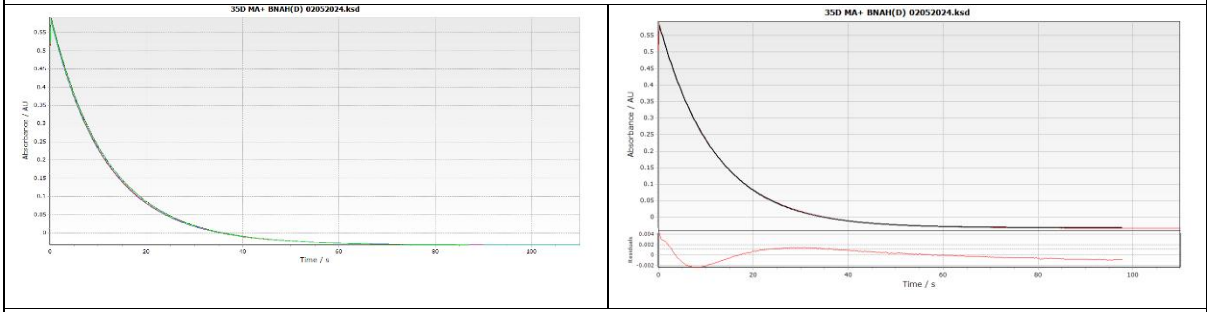

35℃ D

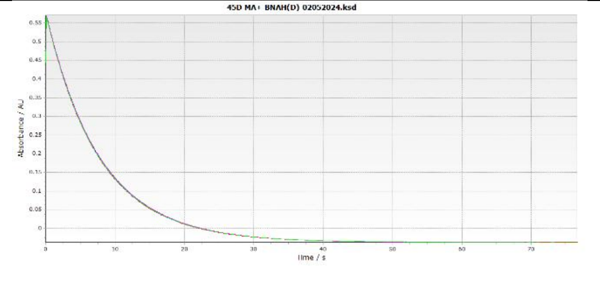

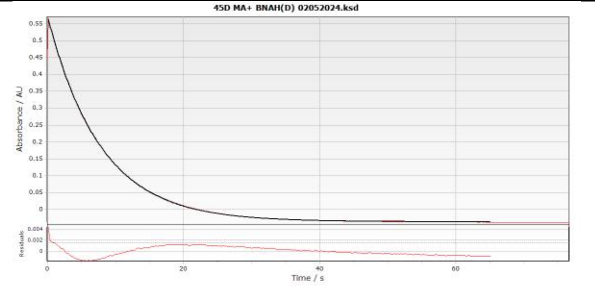

45°C D

Day 2 data (April 11, 2025)

Pseudo-first-order rate constants

k^{pfo} (s^{-1})

Temp (°C)	Trial H1	Trial H2	Trial H3	Trial H4	Trial H5	Trial H6	Average k_H^{pfo} (s^{-1})	Stdev	k_{2H} (M^{-1}s^{-1})	Stdev [a]
45	0.47935	0.4786	0.47768	0.47598	0.47286	0.48164	0.4777	3.01E-03	1.59E+02	1.00E+00
35	0.32079	0.32488	0.31588	0.32316	0.32026	0.31881	0.3206	3.18E-03	1.07E+02	1.06E+00
25	0.21581	0.21558	0.21473	0.21769	0.21832	0.21864	0.2168	1.63E-03	7.23E+01	5.42E-01
15	0.13993	0.1412	0.13948	0.1405	0.1403	0.14186	0.1405	8.64E-04	4.68E+01	2.88E-01
5	0.09012	0.09093	0.08994	0.09064	0.09048	0.09241	0.0908	8.86E-04	3.03E+01	2.95E-01

Temp (°C)	Trial D1	Trial D2	Trial D3	Trial D4	Trial D5	Trial D6	Average k_D^{pfo} (s-1) [b]	Stdev	k_{2D} (M-1s-1)	Stdev [a]
45	0.13194	0.13168	0.13154	0.13119	0.12978	0.13232	0.1170	8.84E-04	3.90E+01	2.62E-01
35	0.08627	0.08574	0.08544	0.08557	0.08683	0.08439	0.0759	8.25E-04	2.53E+01	2.44E-01
25	0.05458	0.0549	0.05464	0.05347	0.05387	0.05428	0.0475	5.34E-04	1.58E+01	1.56E-01
15	0.03368	0.03345	0.03398	0.03423	0.0339	0.03333	0.0293	3.39E-04	9.77E+00	9.83E-02
5	0.02554	0.02486	0.02506	0.02542	0.02465	0.02513	0.0177	1.91E-04	5.90E+00	5.47E-02

[a] = (Stdev(for k^{pfo})/k^{pfo})*k_2

[b] = (Average observed k_D^{pfo} – (0.04*Average k_H^{pfo})/0.96 due to 4% H content

Six kinetic trials and the mean fit	First-order kinetic fitted average of six trials
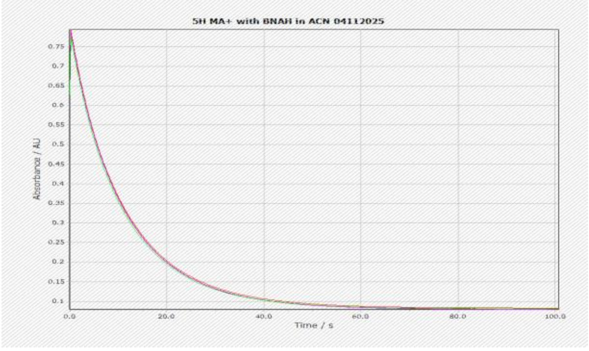	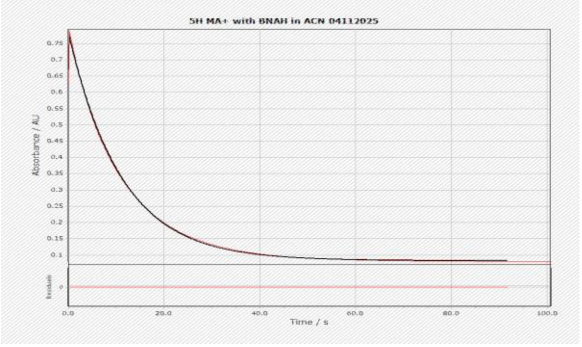

5°C H

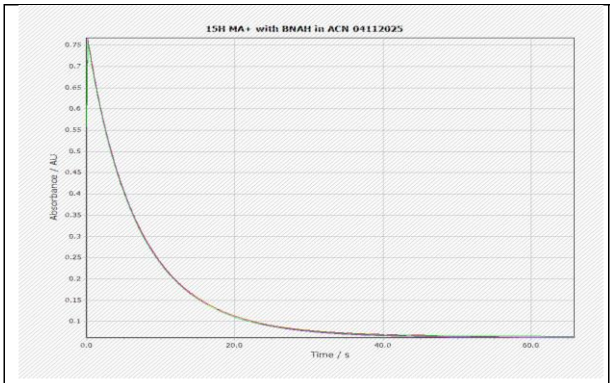

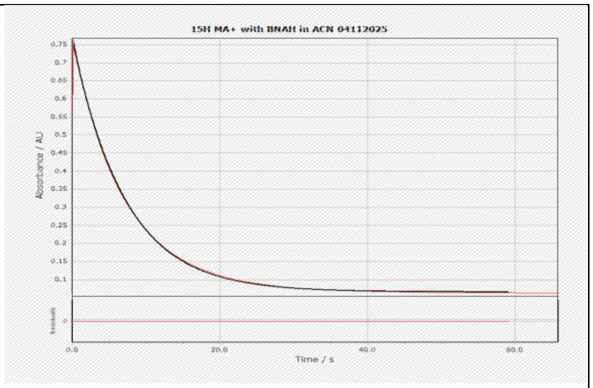

15℃ H

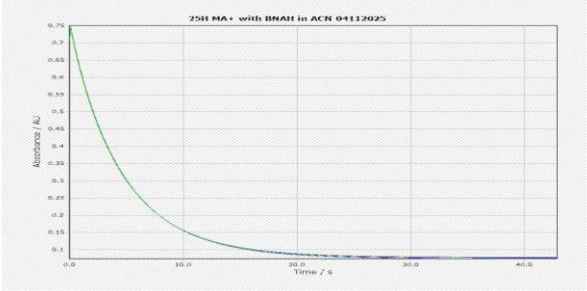

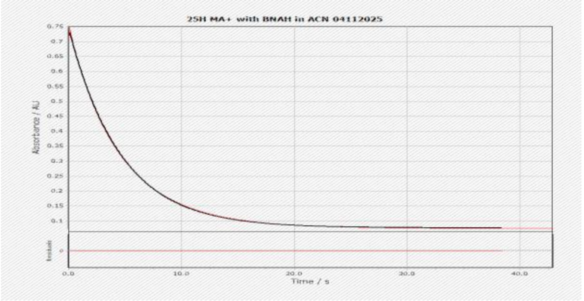

25℃ H

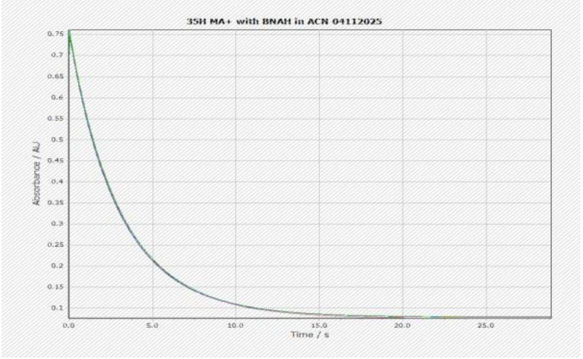

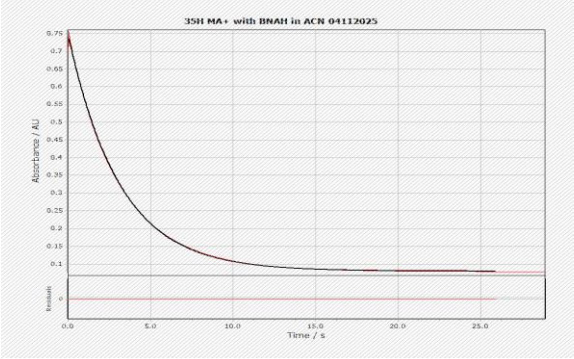

35℃ H

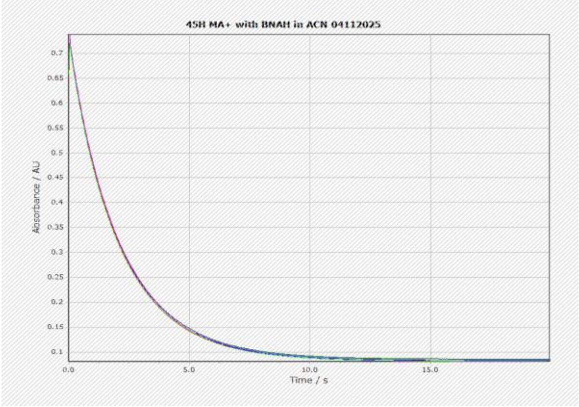

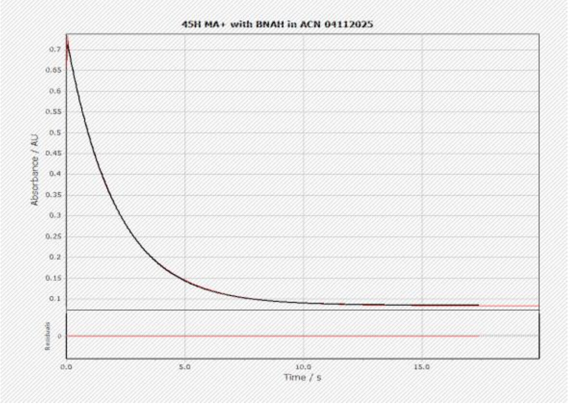

45℃ H

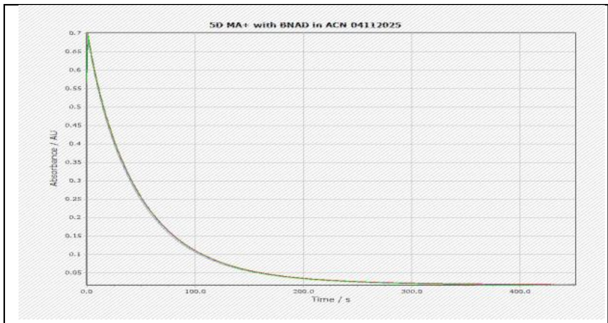

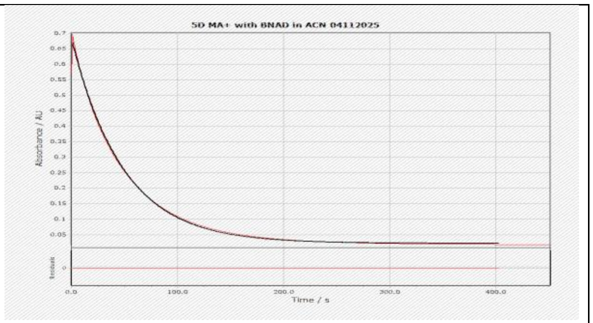

5°C D

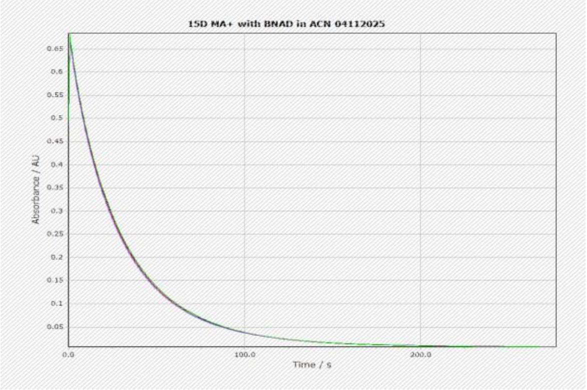

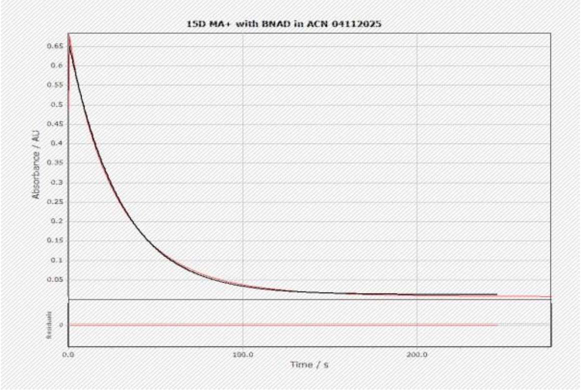

15°C D

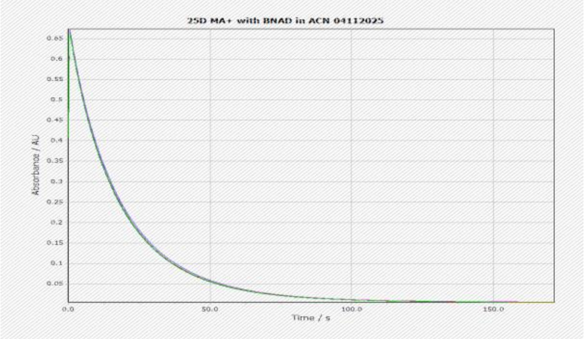

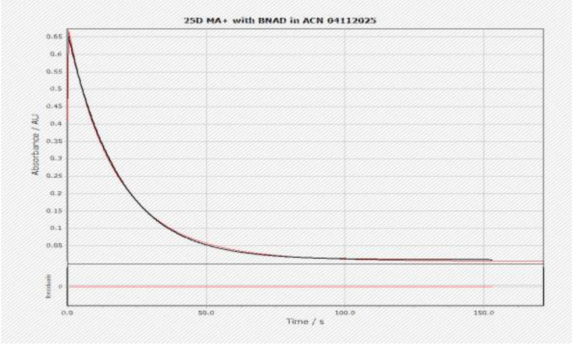

25°C D

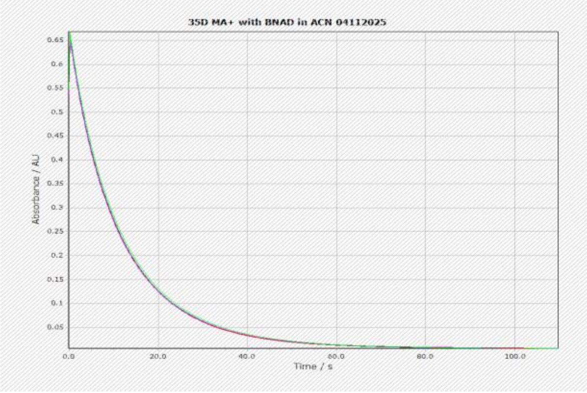

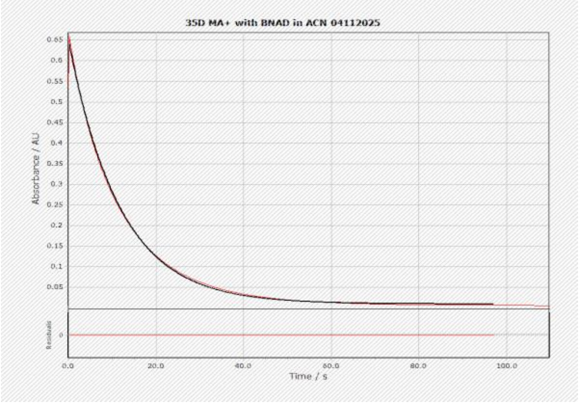

35°C D

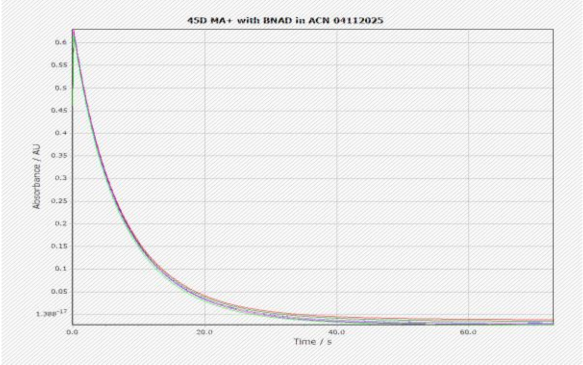 	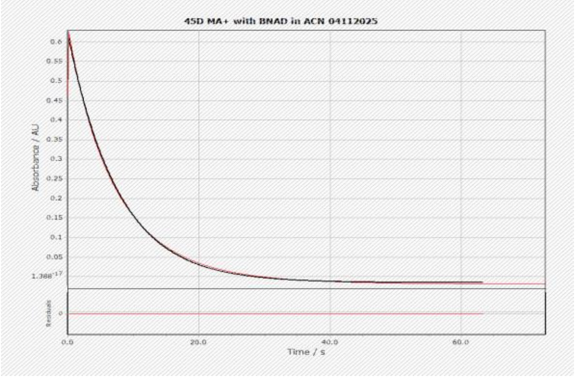
45°C D	

Day 3 data (May 08, 2025)

				Pseudo-first-order rate constants						
				k^{pfo} (s^{-1})						
**Temp (°C)**	**Trial H1**	**Trial H2**	**Trial H3**	**Trial H4**	**Trial H5**	**Trial H6**	**Average k_H^{pfo} (s^{-1})**	**Stdev**	**k_{2H} ($M^{-1}s^{-1}$)**	**Stdev [a]**
45	0.45569	0.45714	0.45514	0.45459	0.45269	0.45404	0.4549	1.51E-03	1.52E+02	5.04E-01
35	0.31257	0.30988	0.30684	0.30966	0.3113	0.30999	0.3100	1.92E-03	1.03E+02	6.39E-01
25	0.21038	0.21191	0.21197	0.20925	0.20831	0.21043	0.2104	1.44E-03	7.01E+01	4.82E-01
15	0.13719	0.137	0.13563	0.13692	0.13764	0.13954	0.1373	1.28E-03	4.58E+01	4.26E-01
5	0.08956	0.08931	0.08825	0.08854	0.08859	0.08797	0.0887	6.14E-04	2.96E+01	2.05E-01
**Temp (°C)**	**Trial D1**	**Trial D2**	**Trial D3**	**Trial D4**	**Trial D5**	**Trial D6**	**Average k_D^{pfo} (s-1) [b]**	**Stdev**	**k_{2D} (M-1s-1)**	**Stdev [a]**
45	0.1364	0.13564	0.13662	0.13584	0.13363	0.13703	0.1091	1.20E-03	3.64E+01	3.22E-01
35	0.08893	0.08806	0.08706	0.08888	0.08828	0.08899	0.0697	7.45E-04	2.32E+01	1.96E-01
25	0.05612	0.05529	0.05675	0.05632	0.05582	0.05667	0.0432	5.49E-04	1.44E+01	1.41E-01
15	0.03477	0.0345	0.03463	0.03494	0.03526	0.03488	0.0262	2.65E-04	8.74E+00	6.66E-02
5	0.02162	0.02174	0.02185	0.02187	0.02177	0.02157	0.0161	1.21E-04	5.37E+00	2.99E-02

[a] = (Stdev(for k^{pfo})/k^{pfo})*k_2
[b] = (Average k_D^{pfo} – (0.04*Average k_H^{pfo})/0.96 due to 4% H content

Six kinetic trials and the mean fit	First-order kinetic fitted average of six trials
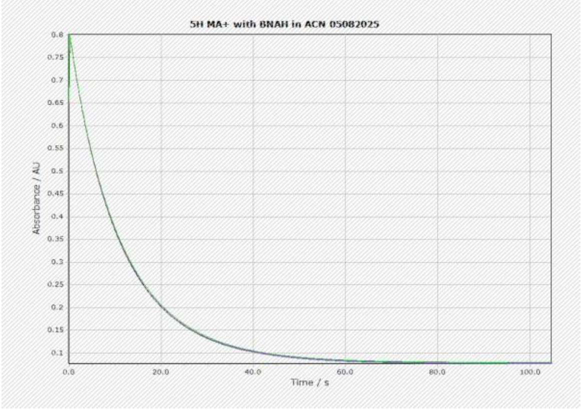 	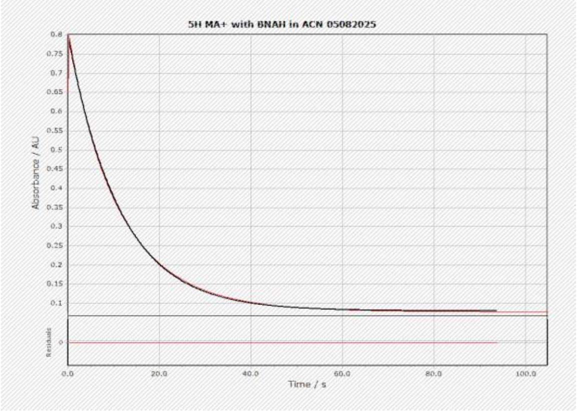
5°C H	

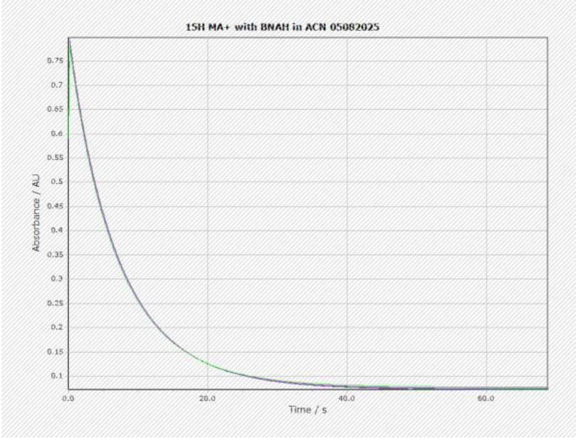

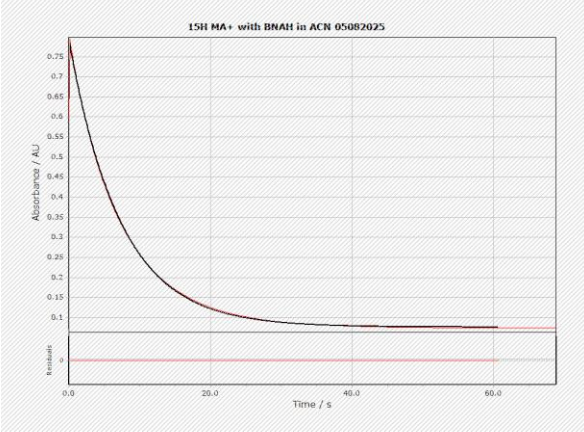

15°C H

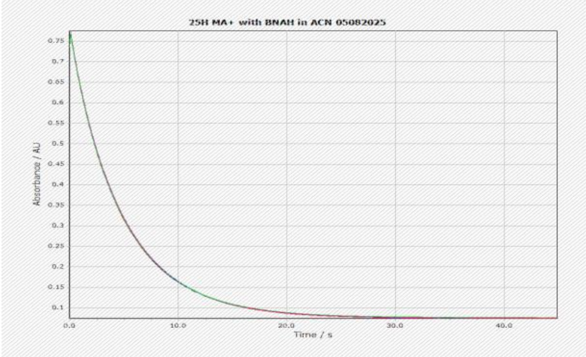

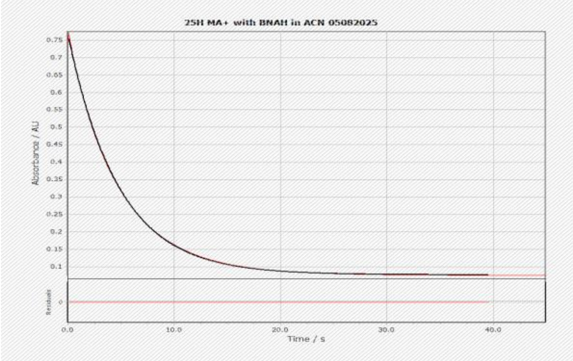

25°C H

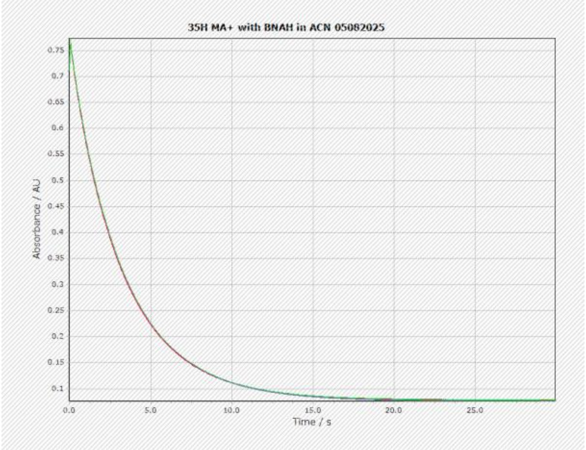

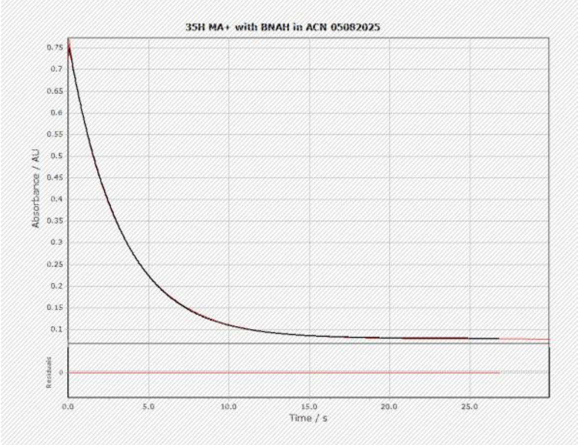

35°C H

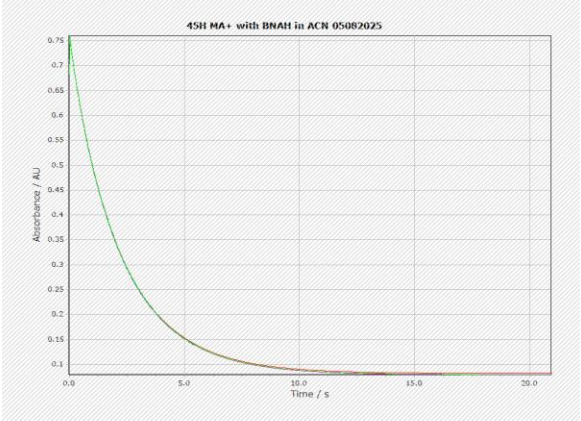

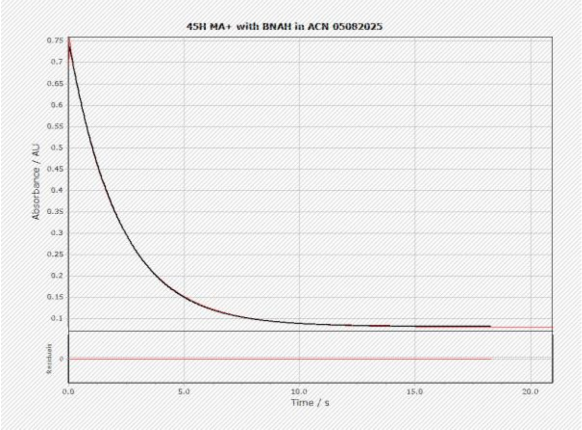

45°C H

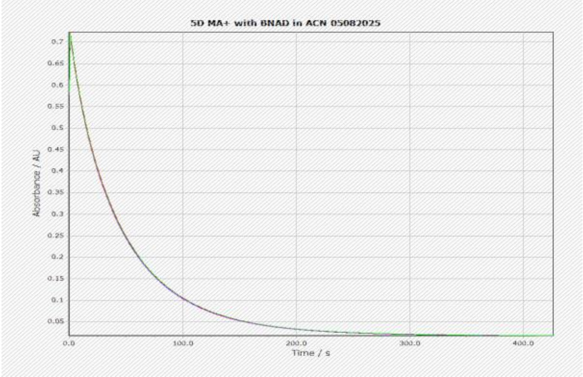

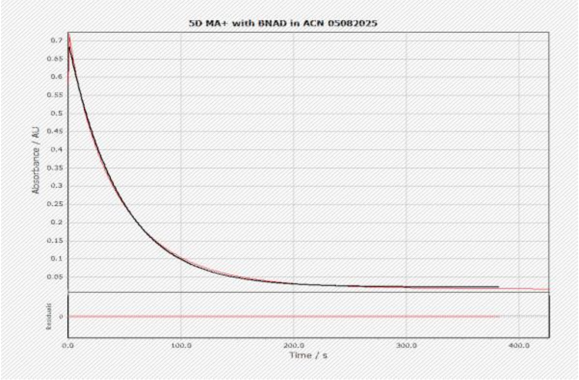

5°C D

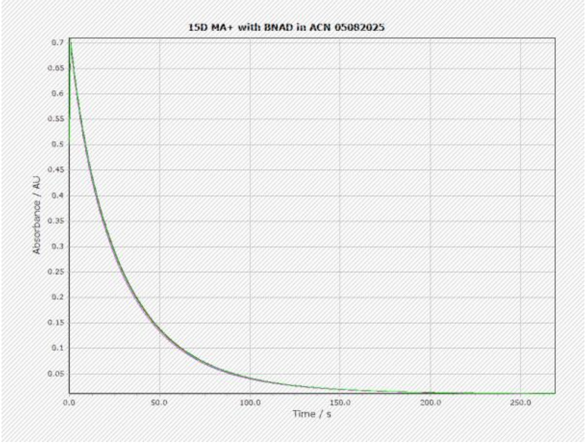

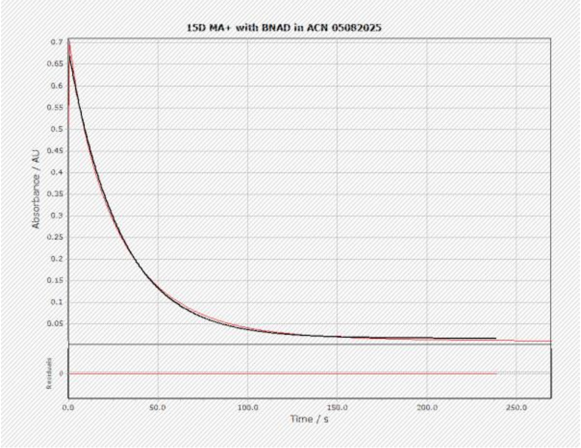

15°C D

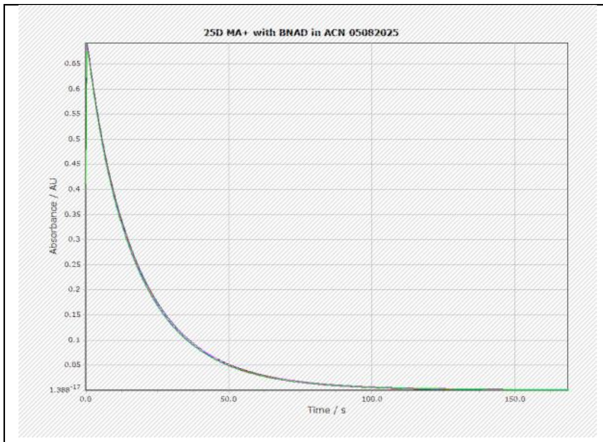

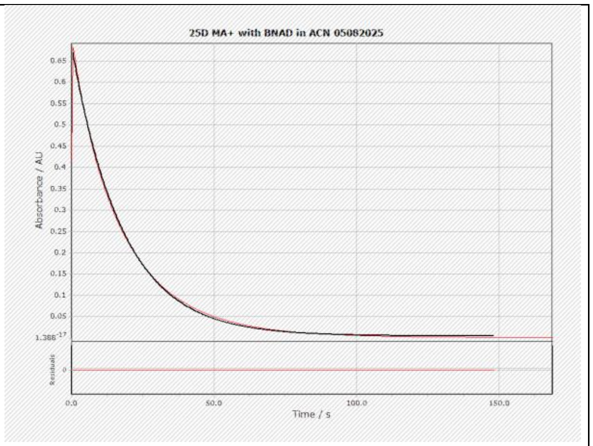

25°C D

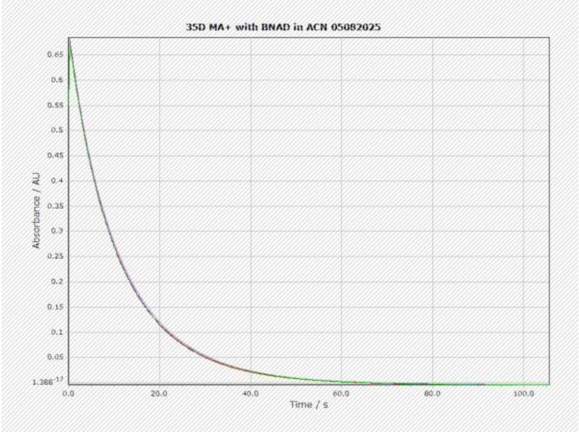

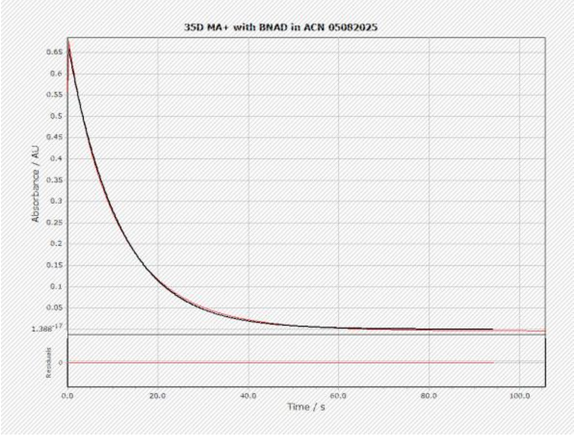

35°C D

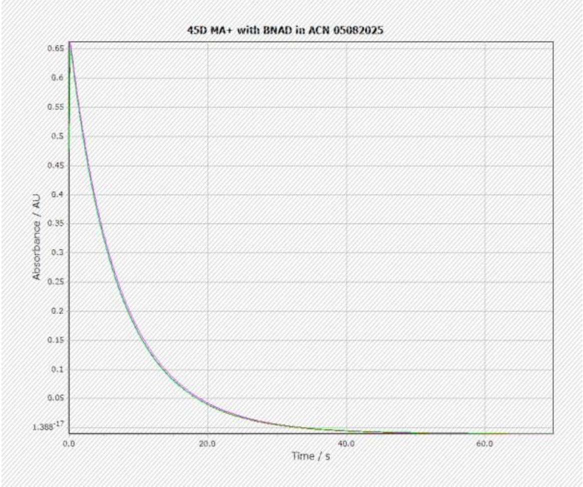

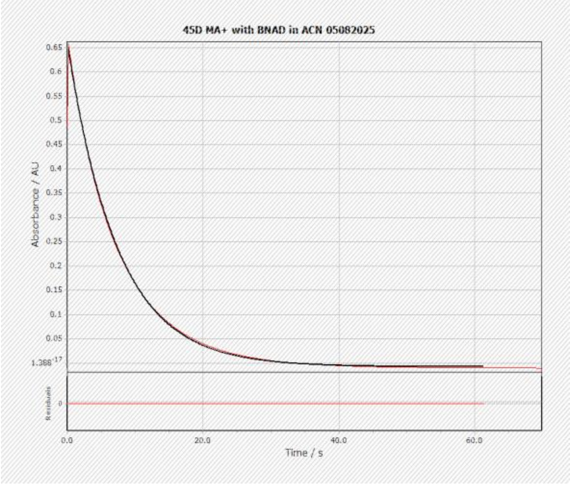

45°C D

Primary kinetic data for the rate constants in Table S8 (BNAH with $BA^+BF_4^-$)

Day 1 (March 26, 2024)

Temp (°C)	Trial H1	Trial H2	Trial H3	Trial H4	Trial H5	Trial H6	Average k_H^{pfo} (s^{-1})	Stdev	k_{2H} ($M^{-1}s^{-1}$)	Stdev [a]
	Pseudo-first-order rate constants k^{pfo} (s^{-1})									
45	1.47147	1.48684	1.4701	1.47804	1.47246	1.46647	1.4742	0.0072326	4.91E+02	2.41087
35	1.04756	1.02767	1.04904	1.03795	1.04394	1.0401	1.0410	0.0077988	3.47E+02	2.59961
25	0.74796	0.74537	0.75156	0.74129	0.75502	0.74997	0.7485	0.0048188	2.50E+02	1.60626
15	0.52258	0.52172	0.52269	0.52517	0.52029	0.52178	0.5224	0.0016176	1.74E+02	0.53921
5	0.36574	0.368	0.36449	0.37119	0.36674	0.36607	0.3670	0.0023396	1.22E+02	0.77985

Temp (°C)	Trial D1	Trial D2	Trial D3	Trial D4	Trial D5	Trial D6	Average k_D^{pfo} (s^{-1}) [b]	Stdev	k_{2D} ($M^{-1}s^{-1}$)	Stdev [a]
45	0.41058	0.41038	0.41807	0.41492	0.41012	0.4133	0.3687	0.003175857	1.23E+02	0.94524
35	0.28921	0.28567	0.28431	0.28355	0.28424	0.28988	0.2547	0.002731312	8.49E+01	0.81036
25	0.19823	0.19937	0.19993	0.19837	0.19887	0.19998	0.1762	0.000758307	5.87E+01	0.22371
15	0.13365	0.13445	0.13229	0.13273	0.13288	0.13414	0.1171	0.00085472	3.90E+01	0.25028
5	0.08813	0.08858	0.08771	0.08769	0.08758	0.08715	0.0762	0.000491555	2.54E+01	0.14214

[a] = (Stdev(for k^{pfo})/k^{pfo})*k_2

[b] = (Average k_D^{pfo} – (0.04*Average k_H^{pfo})/0.96 due to 4% H content

Six kinetic runs together with the averaged data	First-order kinetic fit of the averaged data as an example
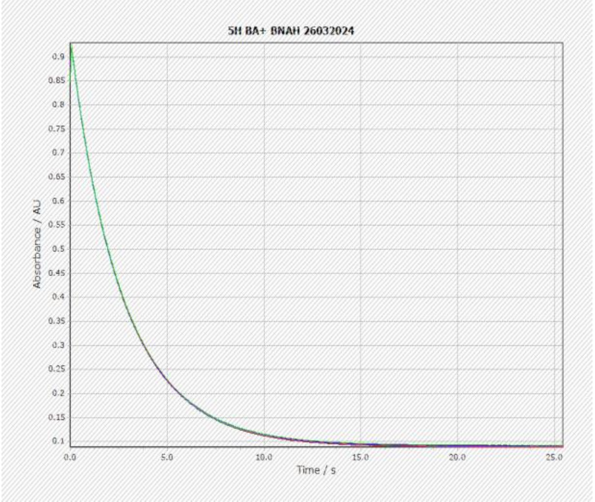	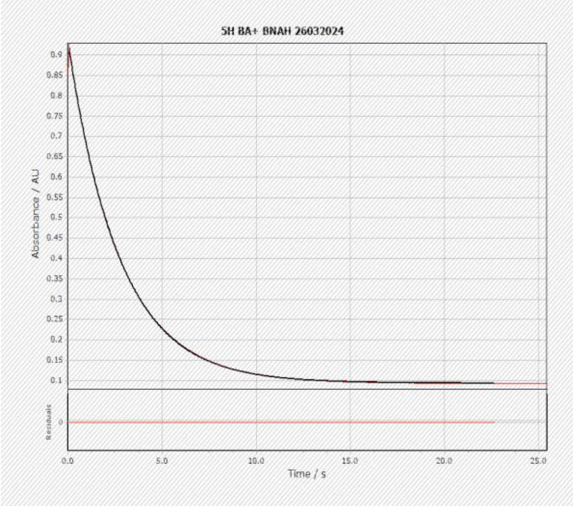
5°C H	

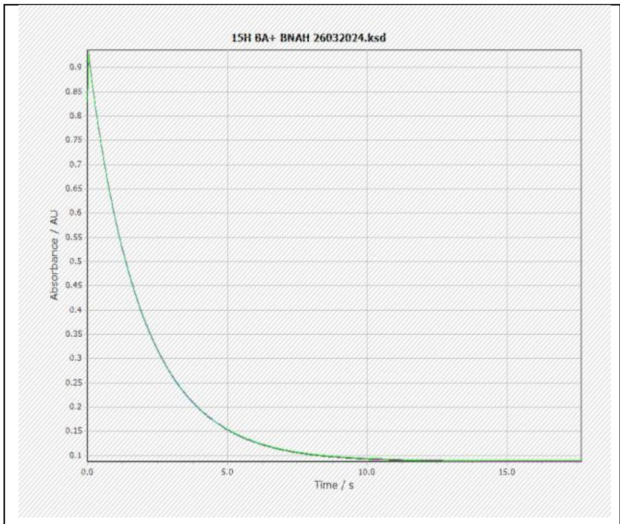

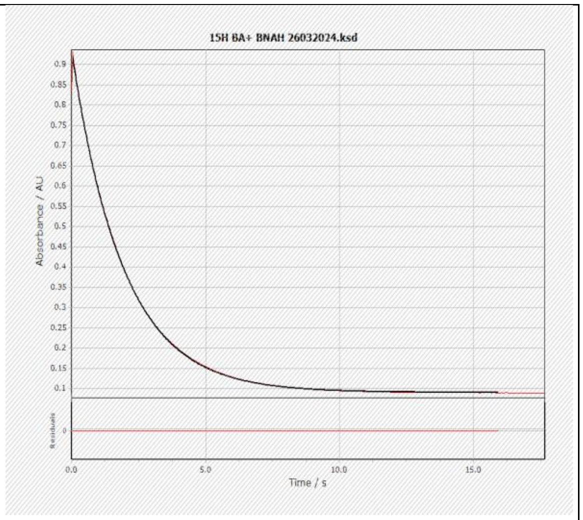

15°C H

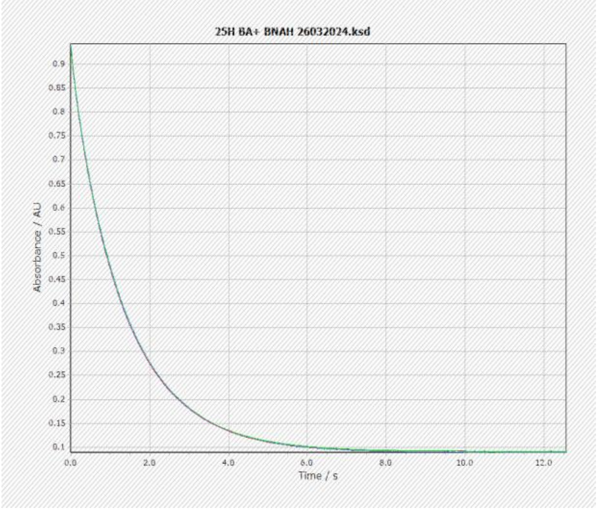

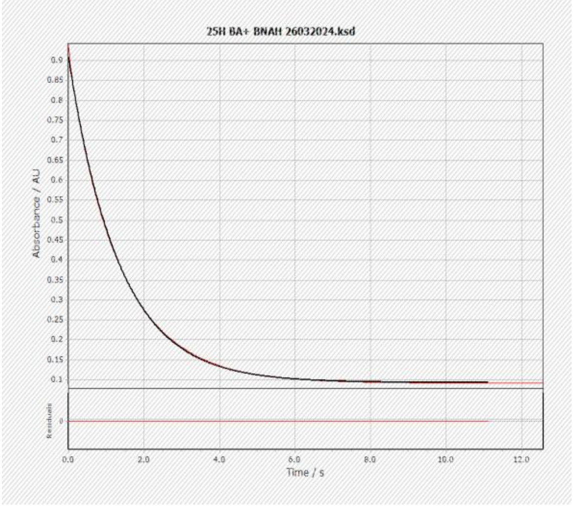

25°C H

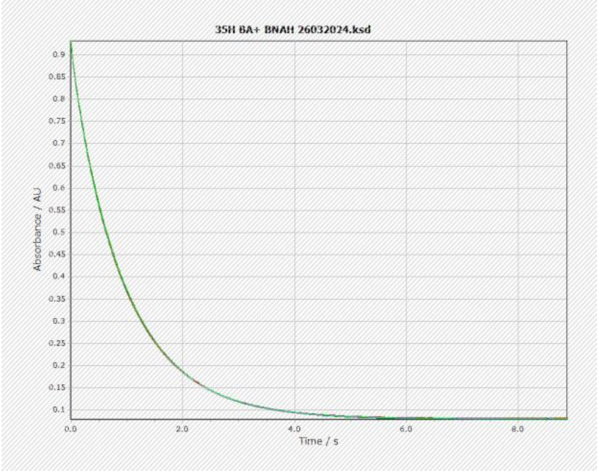

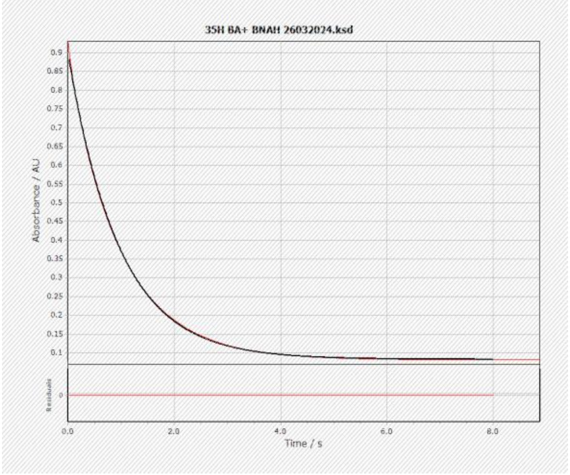

35°C H

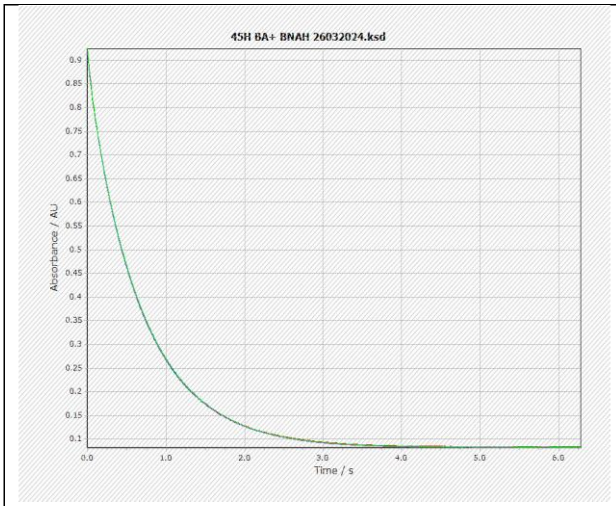

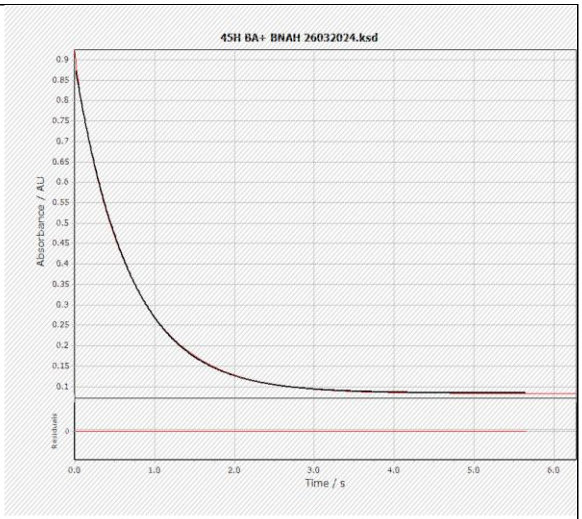

45°C H

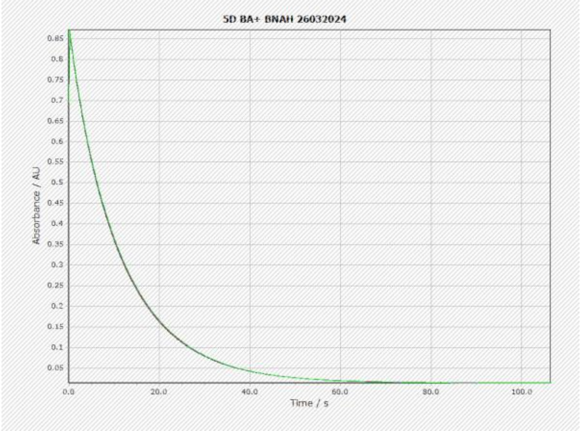

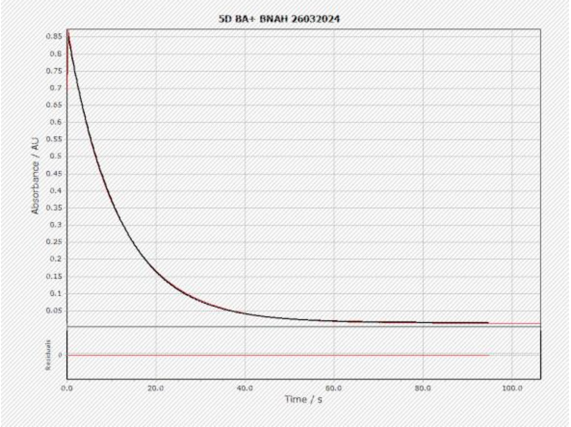

5°C D

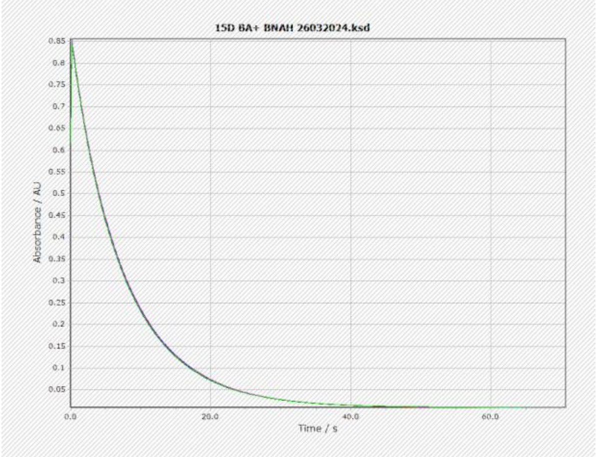

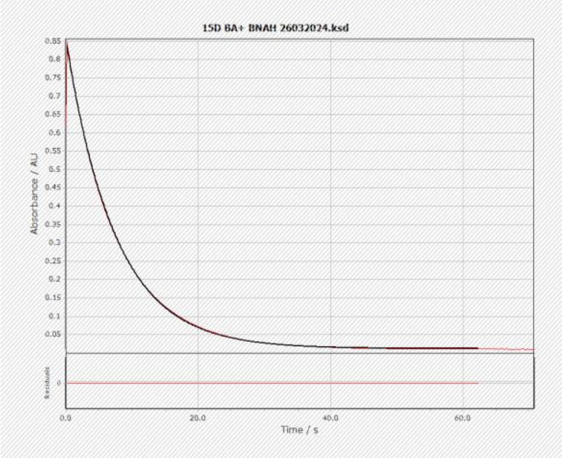

15°C D

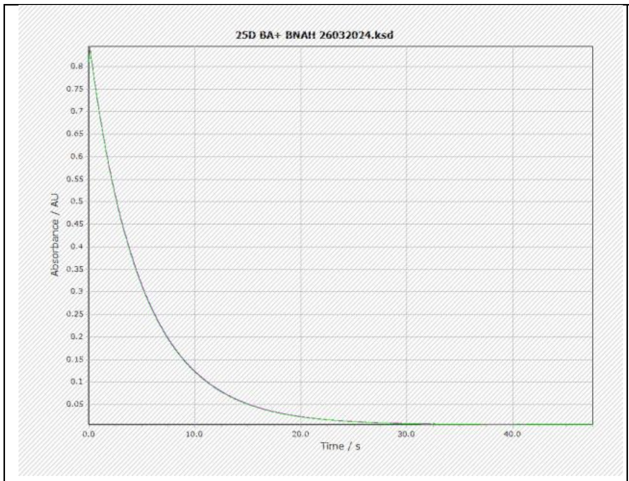
25D BA+ BNAH 26032024.ksd
Absorbance / AU
Time / s

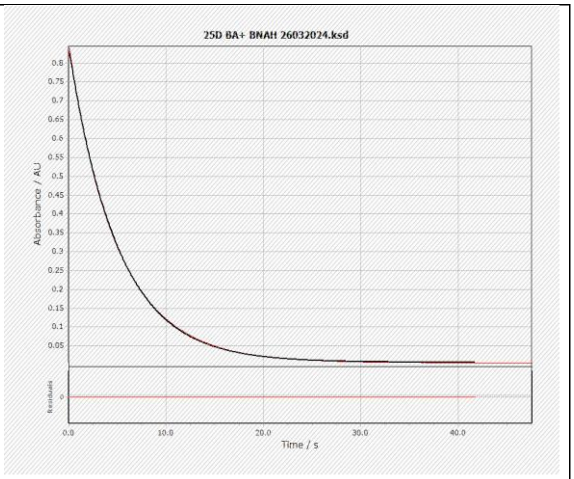
25D BA+ BNAH 26032024.ksd
Absorbance / AU
Residuals
Time / s

25℃ D

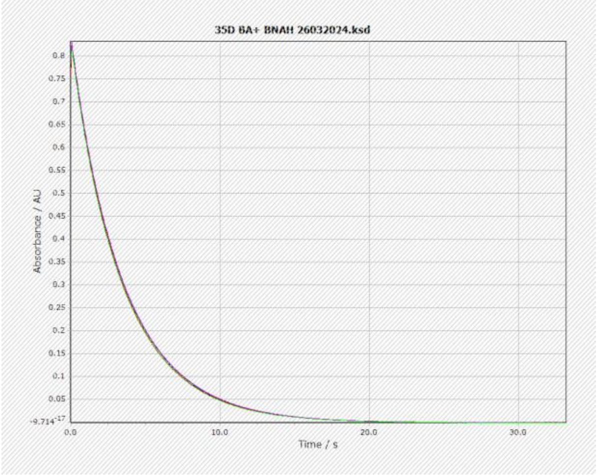
35D BA+ BNAH 26032024.ksd
Absorbance / AU
Time / s

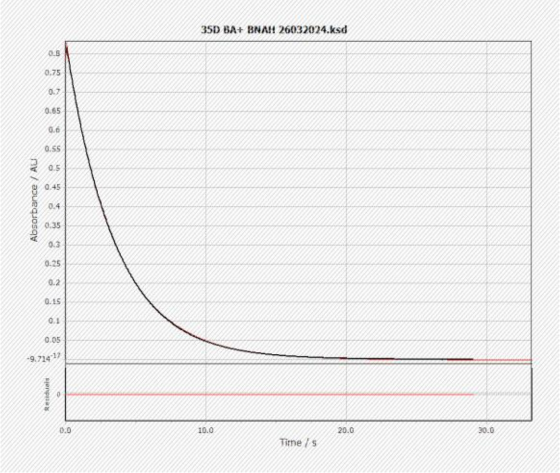
35D BA+ BNAH 26032024.ksd
Absorbance / AU
Residuals
Time / s

35℃ D

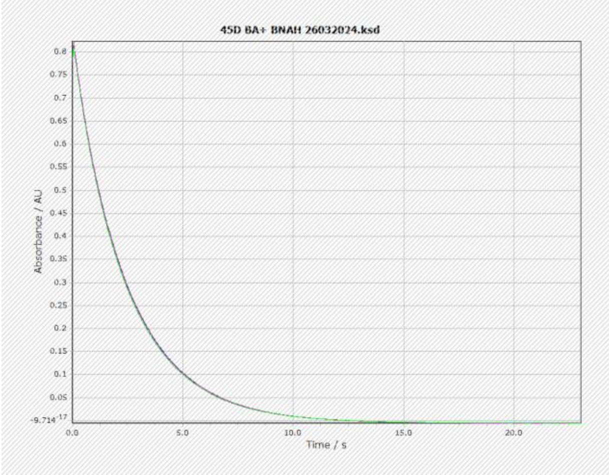
45D BA+ BNAH 26032024.ksd
Absorbance / AU
Time / s

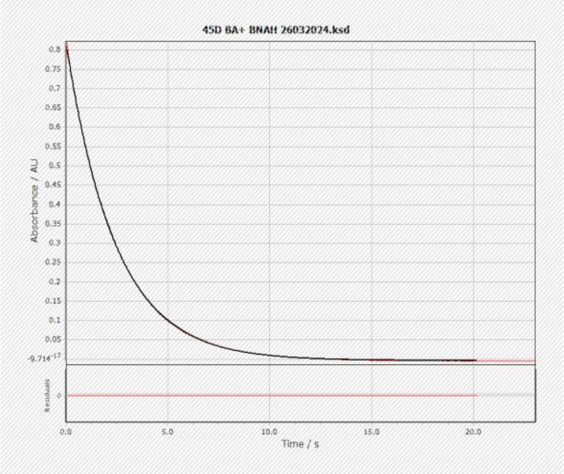
45D BA+ BNAH 26032024.ksd
Absorbance / AU
Residuals
Time / s

45℃ D

Day 2 (April 02, 2024)

	Pseudo-first-order rate constants k^{pfo} (s^{-1})									
**Temp (°C)**	**Trial H1**	**Trial H2**	**Trial H3**	**Trial H4**	**Trial H5**	**Trial H6**	**Average k_H^{pfo} (s^{-1})**	**Stdev**	**k_{2H} ($M^{-1}s^{-1}$)**	**Stdev [a]**
45	1.42722	1.42982	1.45158	1.4314	1.43837	1.44382	1.4370	0.0093757	4.79E+02	3.12523
35	1.03311	1.02938	1.04468	1.04347	1.03761	1.03507	1.0372	0.005964	3.46E+02	1.98800
25	0.74412	0.73184	0.74164	0.73391	0.73501	0.73622	0.7371	0.0047513	2.46E+02	1.58377
15	0.5147	0.51341	0.50913	0.5164	0.52063	0.52356	0.5163	0.0051728	1.72E+02	1.72425
5	0.35215	0.35242	0.35073	0.3552	0.35352	0.35449	0.3531	0.0016431	1.18E+02	0.54770

**Temp (°C)**	**Trial D1**	**Trial D2**	**Trial D3**	**Trial D4**	**Trial D5**	**Trial D6**	**Average k_D^{pfo} (s^{-1}) [b]**	**Stdev**	**k_{2D} ($M^{-1}s^{-1}$)**	**Stdev [a]**
45	0.4113	0.40544	0.40937	0.40921	0.4105	0.40339	0.3653	0.003099764	1.22E+02	0.92475
35	0.28705	0.2834	0.28595	0.28611	0.28619	0.2855	0.2544	0.001234763	8.48E+01	0.36648
25	0.19419	0.19712	0.19704	0.19734	0.1971	0.19899	0.1745	0.00154853	5.82E+01	0.45719
15	0.13075	0.13243	0.13253	0.13312	0.12978	0.13231	0.1158	0.001273797	3.86E+01	0.37300
5	0.08408	0.08646	0.08596	0.08622	0.08562	0.08573	0.0745	0.00084196	2.48E+01	0.24416

[a] = (Stdev(for k^{pfo})/k^{pfo})*k_2

[b] = (Average k_D^{pfo} – (0.04*Average k_H^{pfo})/0.96 due to 4% H content

Six kinetic runs together with the averaged data	First-order kinetic fit of the averaged data as an example
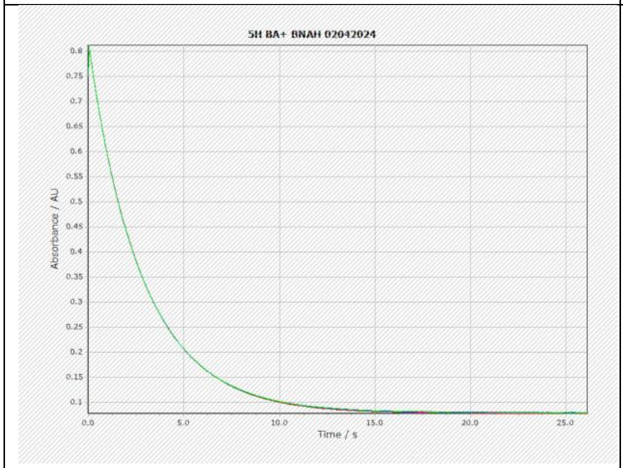	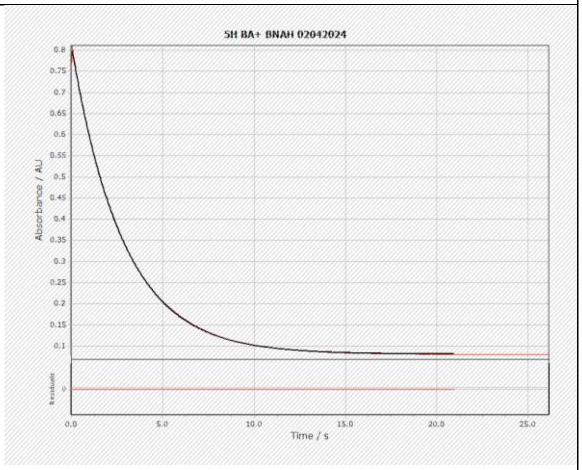
5°C H	

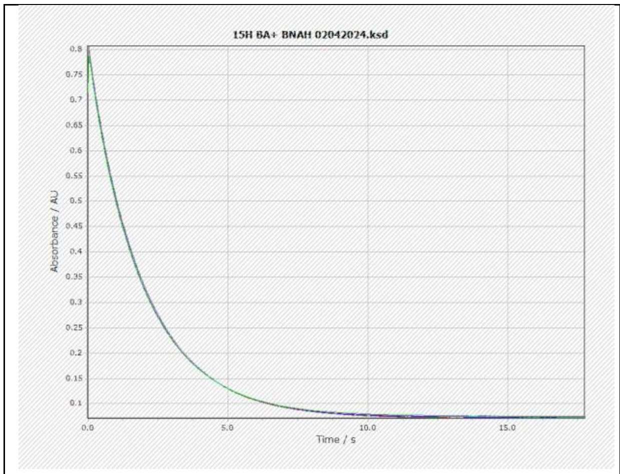

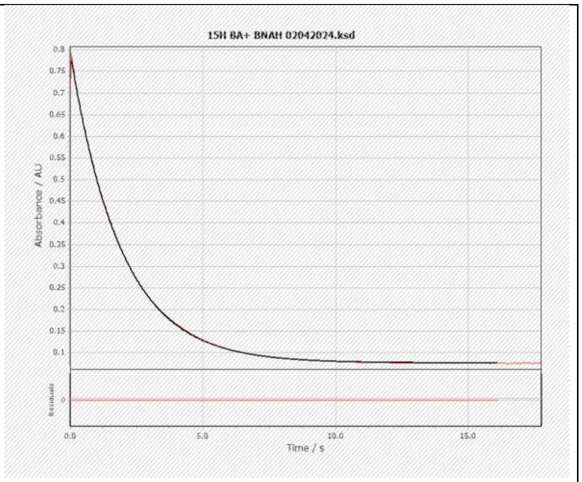

15°C H

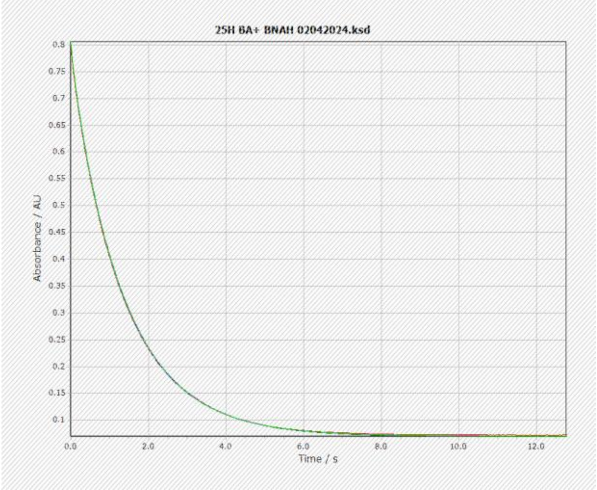

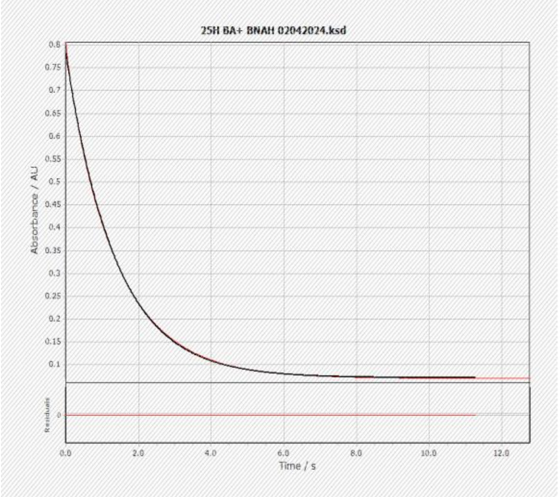

25°C H

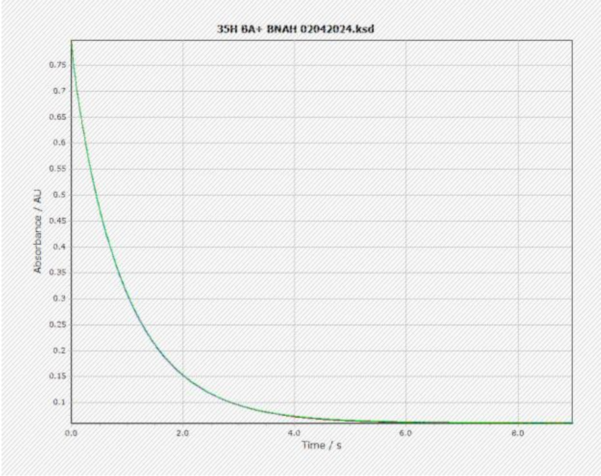

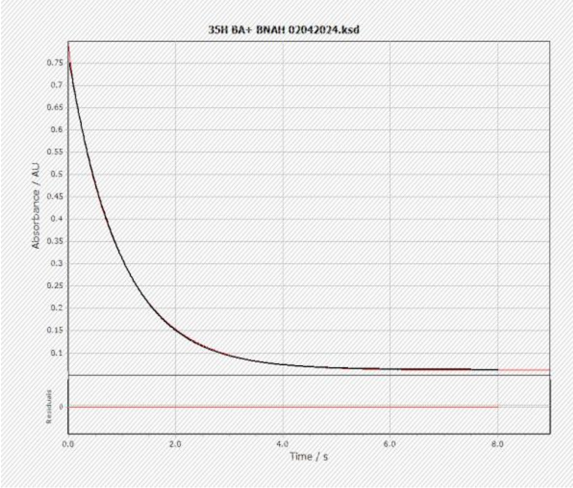

35°C H

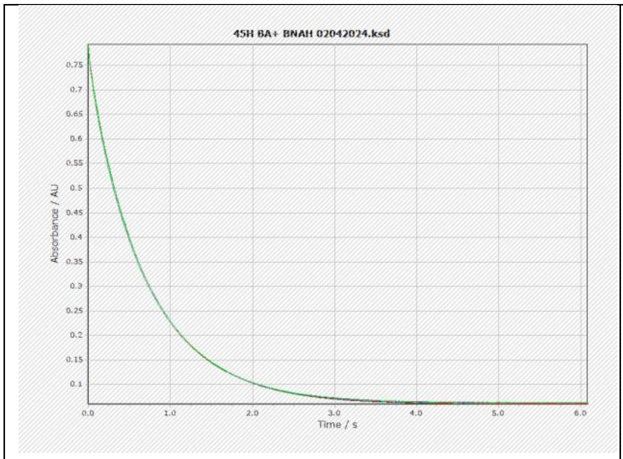

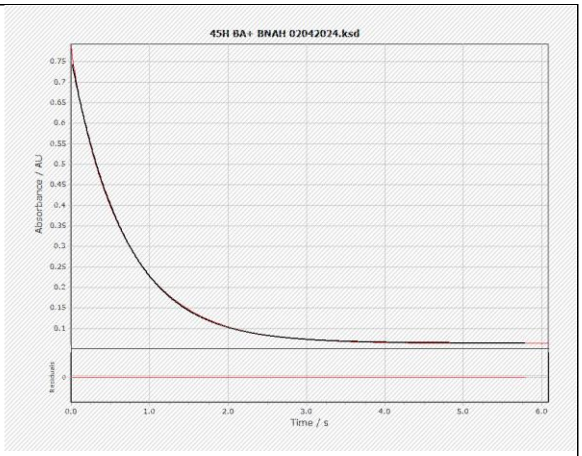

45°C H

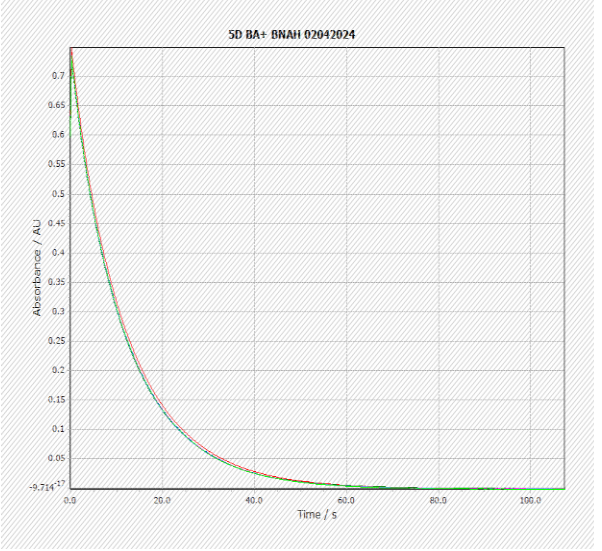

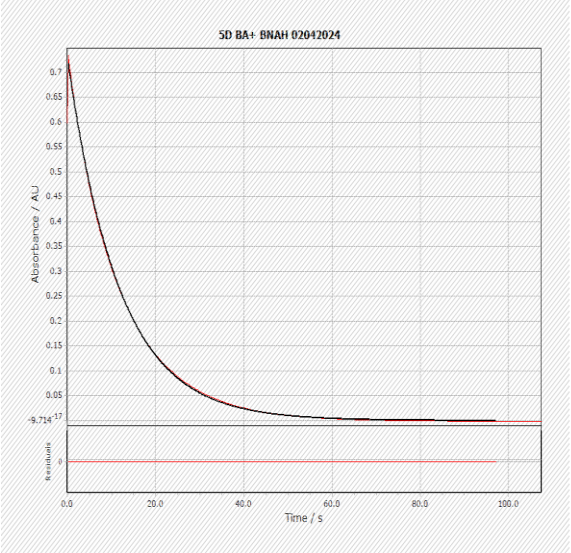

5°C D

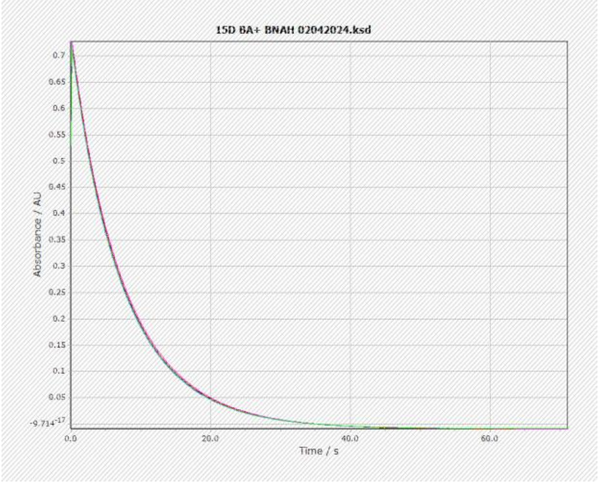

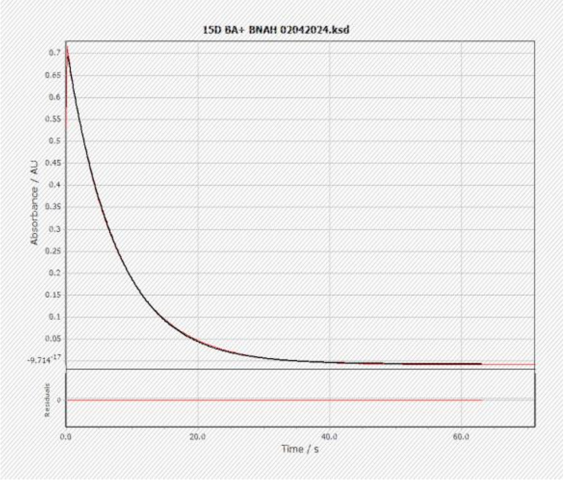

15°C D

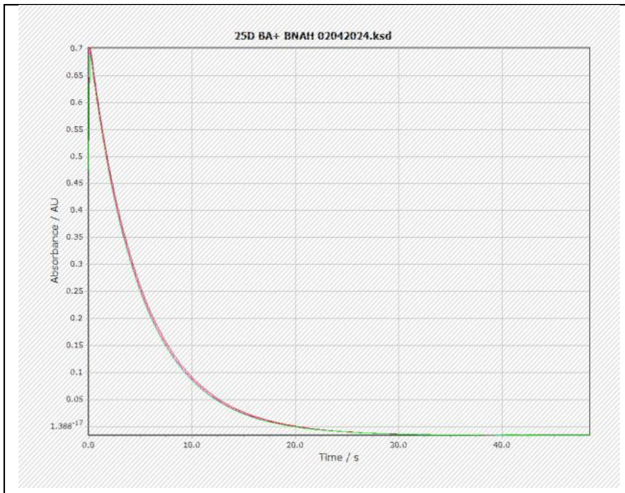

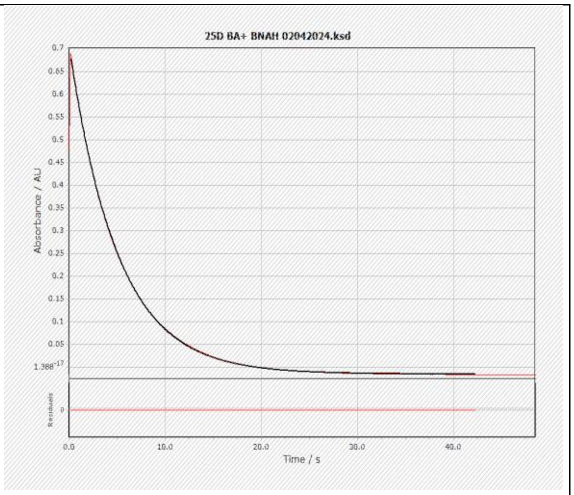

25°C D

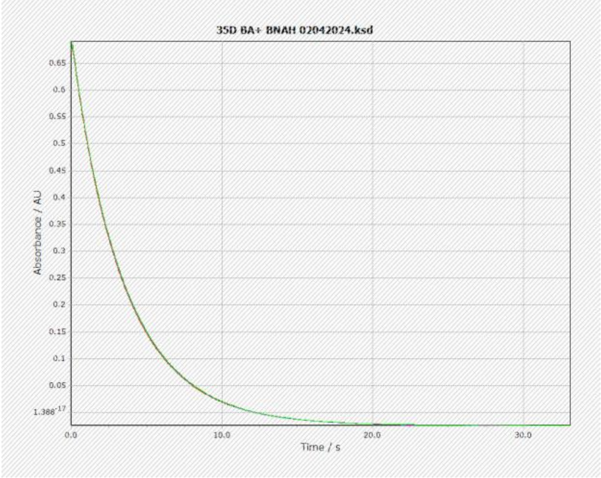

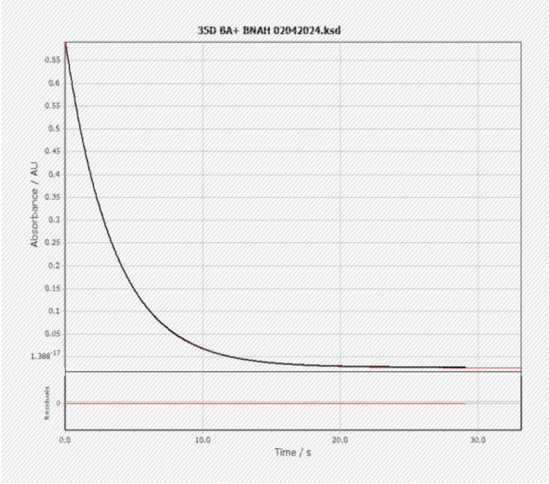

35°C D

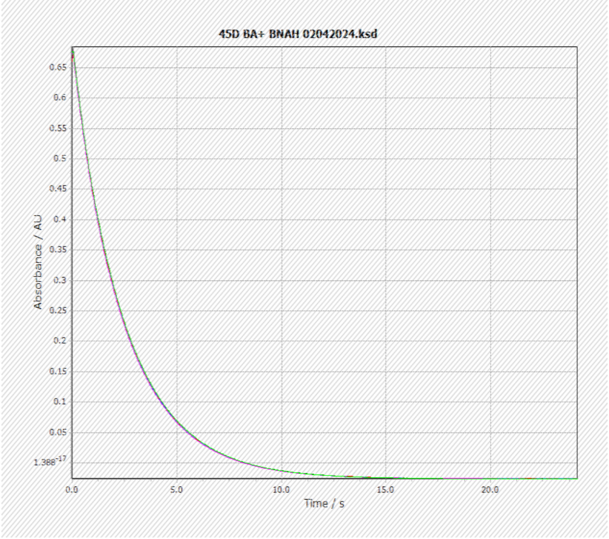

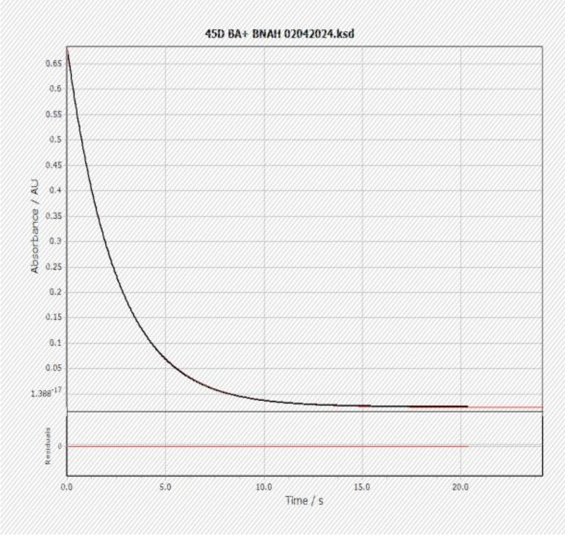

45°C D

Primary kinetic data for the rate constants in Table S9 (HEH with NBMN)

Day 1 data (May 21, 2025)

Pseudo-first-order rate constants k^{pfo} (s^{-1})

**Temp (°C)**	**Trial H1**	**Trial H2**	**Trial H3**	**Average k_H^{pfo} (s^{-1})**	**Stdev**	**k_{2H} ($M^{-1}s^{-1}$)**	**Stdev [a]**
55	0.03409	0.03333	0.03374	0.03372	3.80E-04	4.22E+00	4.75E-02
45	0.02256	0.02259	0.02255	0.02257	2.08E-05	2.82E+00	2.60E-03
35	0.01453	0.01466	0.01459	0.01459	6.51E-05	1.82E+00	8.13E-03
25	0.00929	0.00920	0.00924	0.00924	4.51E-05	1.16E+00	5.64E-03
15	0.00530	0.00533	0.00534	0.00532	2.08E-05	6.65E-01	2.60E-03

**Temp (°C)**	**Trial D1**	**Trial D2**	**Trial D3**	**Average k_D^{pfo} (s^{-1})**	**Stdev**	**k_{2D} ($M^{-1}s^{-1}$)**	**Stdev [a]**
55	0.00792	0.00791	0.00789	0.00791	1.53E-05	9.88E-01	1.91E-03
45	0.00500	0.00495	0.00501	0.00499	3.21E-05	6.23E-01	4.02E-03
35	0.00304	0.00306	0.00302	0.00304	2.00E-05	3.80E-01	2.50E-03
25	0.00180	0.00177	0.00177	0.00178	1.73E-05	2.23E-01	2.17E-03
15	0.00099	0.00100	0.00098	0.00099	1.00E-05	1.24E-01	1.25E-03

[a] = (Stdev(for k^{pfo})/k^{pfo})*k_2

Three kinetic runs together with the averaged data	First-order kinetic fit of the averaged data as an example
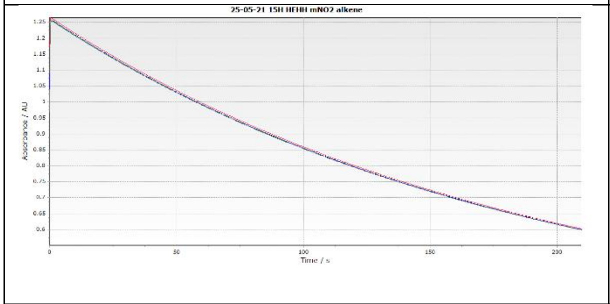	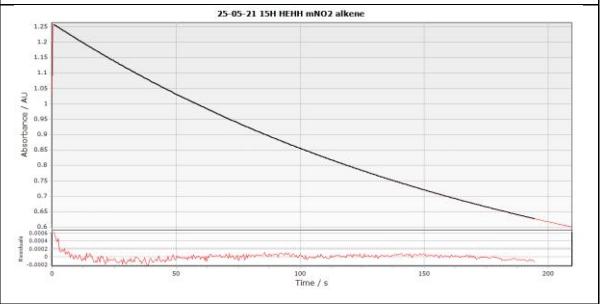
15°C H	
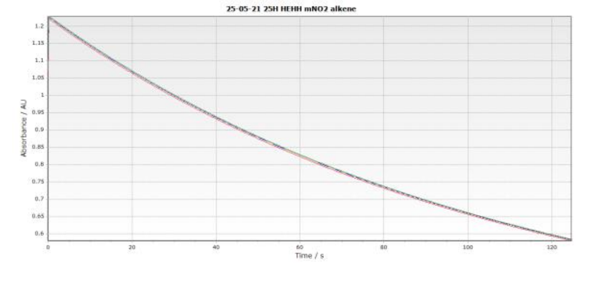	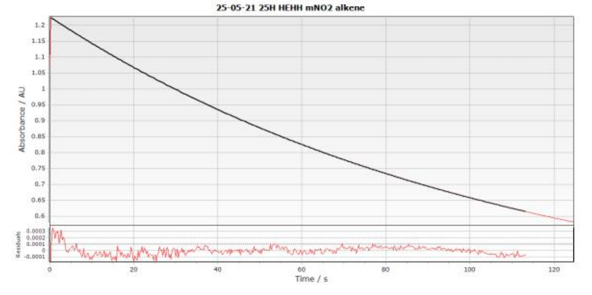
25°C H	

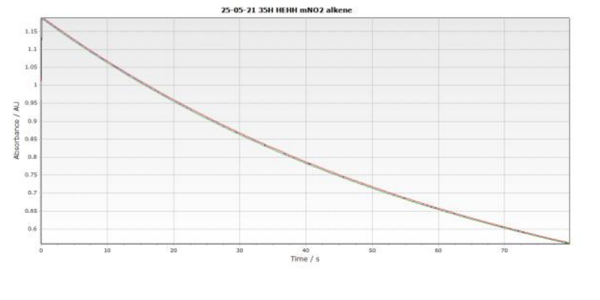

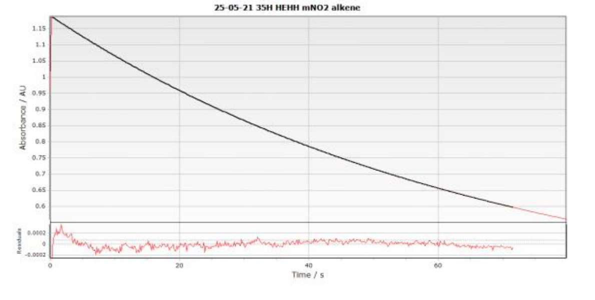

35°C H

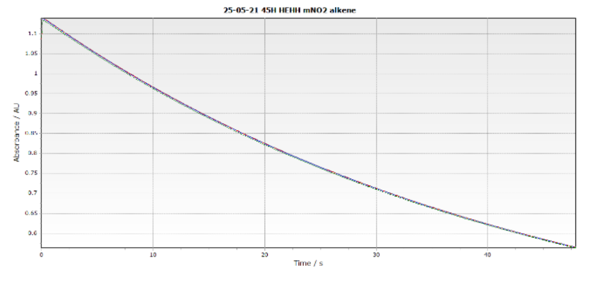

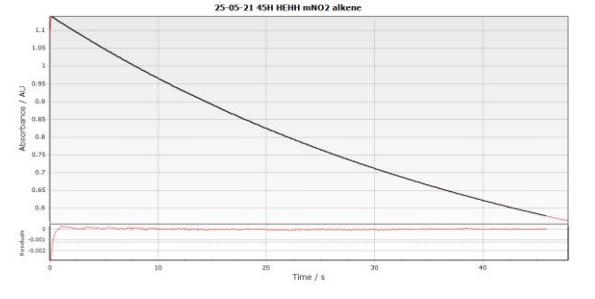

45°C H

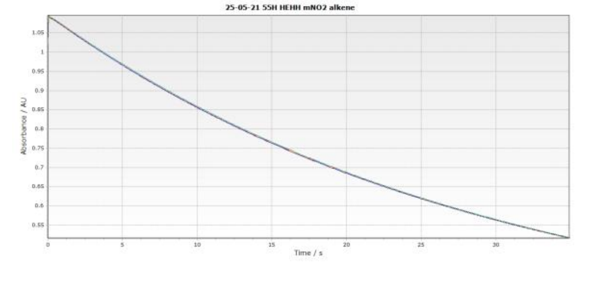

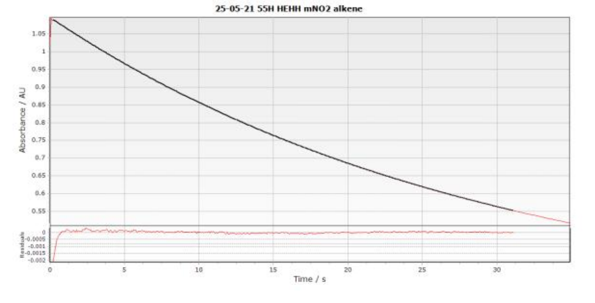

55°C H

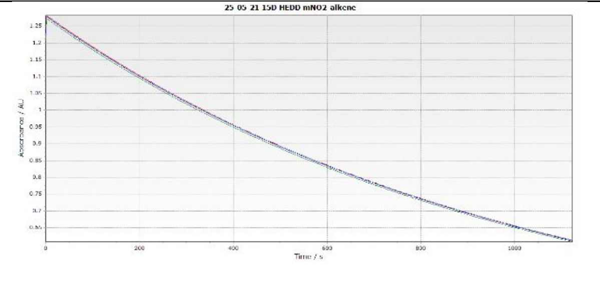

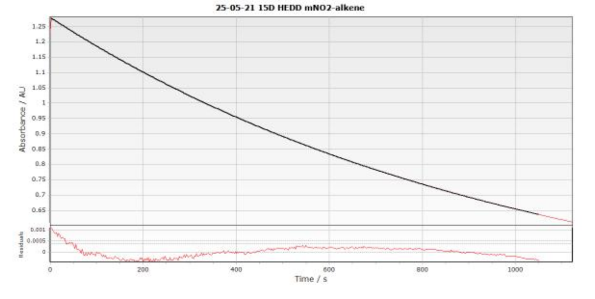

15°C D

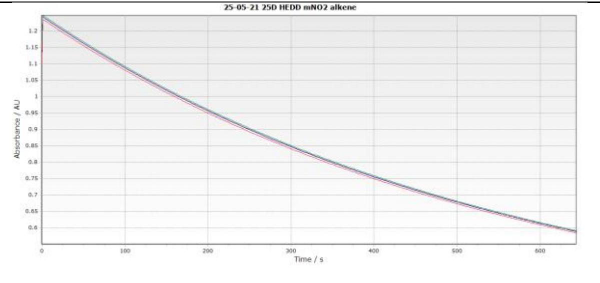

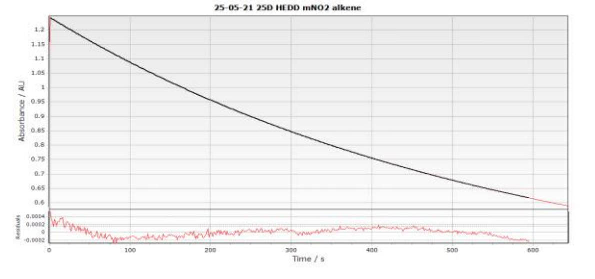

25°C D

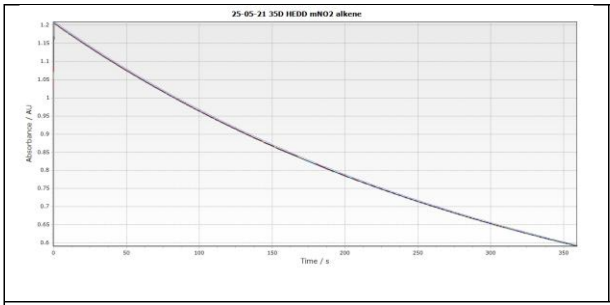

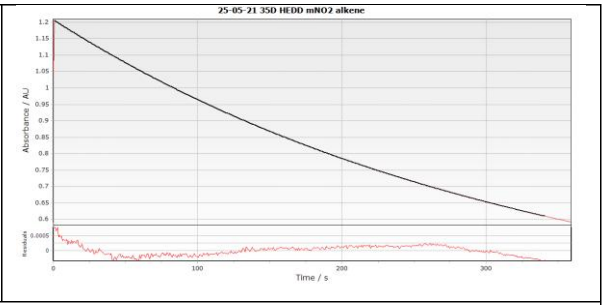

35°C D

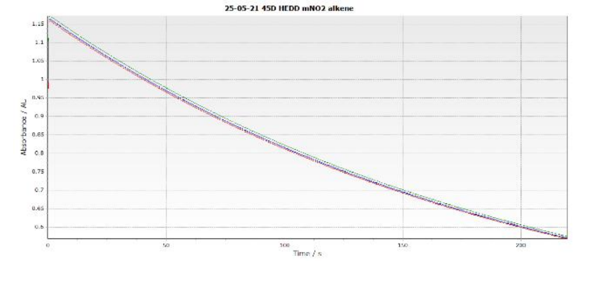

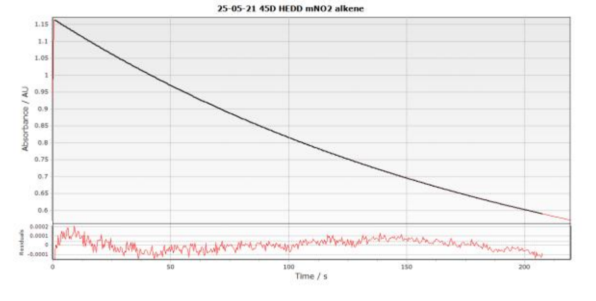

45°C D

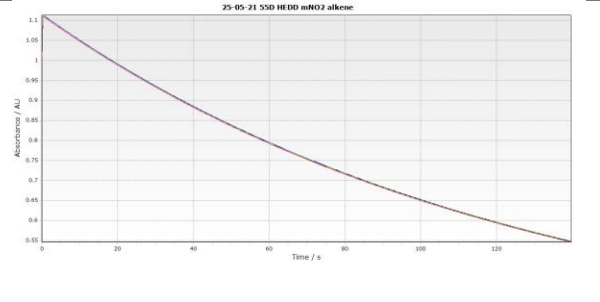

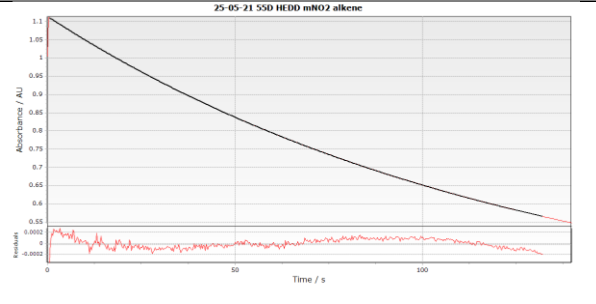

55°C D

Day 2 data (May 27, 2025)

Pseudo-first-order rate constants

k^{pfo} (s^{-1})

**Temp (°C)**	**Trial H1**	**Trial H2**	**Trial H3**	**Average k_H^{pfo} (s^{-1})**	**Stdev**	**k_{2H} ($M^{-1}s^{-1}$)**	**Stdev [a]**
55	0.03335	0.03220	0.03205	0.03253	7.11E-04	4.07E+00	8.89E-02
45	0.02188	0.02198	0.02195	0.02194	5.13E-05	2.74E+00	6.41E-03
35	0.01416	0.01410	0.01422	0.01416	6.00E-05	1.77E+00	7.50E-03
25	0.00896	0.00890	0.00901	0.00896	5.51E-05	1.12E+00	6.88E-03
15	0.00527	0.00527	0.00527	0.00527	0.00E+00	6.59E-01	0.00E+00

**Temp (°C)**	**Trial D1**	**Trial D2**	**Trial D3**	**Average k_D^{pfo} (s^{-1})**	**Stdev**	**k_{2D} ($M^{-1}s^{-1}$)**	**Stdev [a]**
55	0.00751	0.00757	0.00754	0.00754	3.00E-05	9.43E-01	3.75E-03
45	0.00480	0.00482	0.00482	0.00481	1.15E-05	6.02E-01	1.44E-03
35	0.00291	0.00297	0.00297	0.00295	3.46E-05	3.69E-01	4.33E-03
25	0.00169	0.00170	0.00172	0.00170	1.52E-05	2.13E-01	1.91E-03
15	0.00097	0.00098	0.00098	0.00098	5.77E-06	1.22E-01	7.22E-04

[a] = (Stdev(for k^{pfo})/k^{pfo})*k_2

Three kinetic runs together with the averaged data	First-order kinetic fit of the averaged data as an example
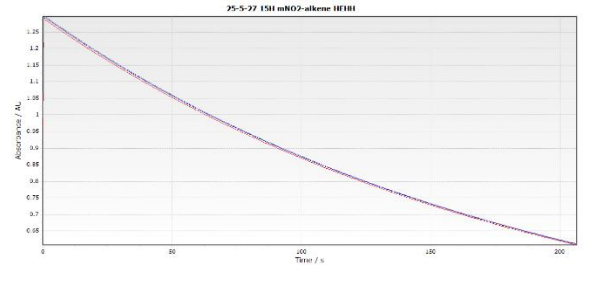	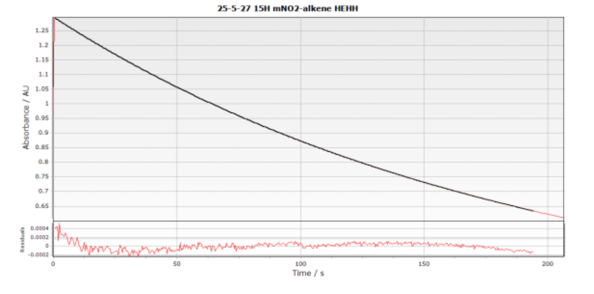
15℃ H	
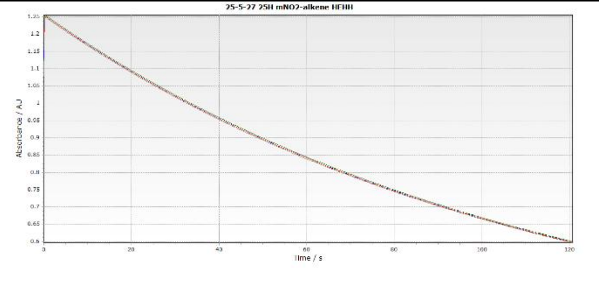	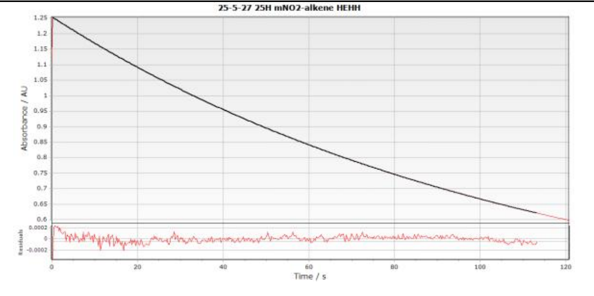
25℃ H	
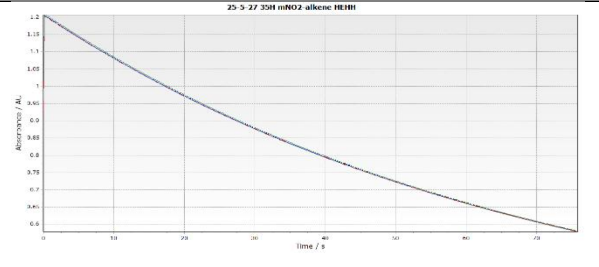	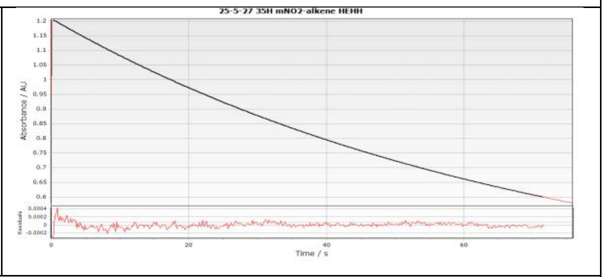
35℃ H	
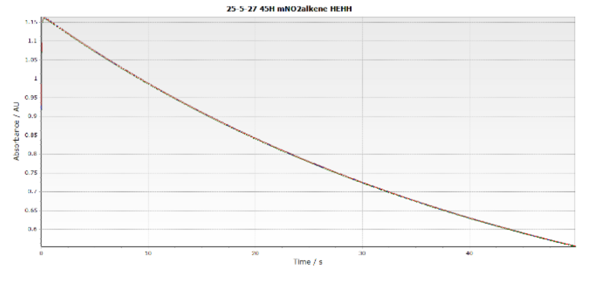	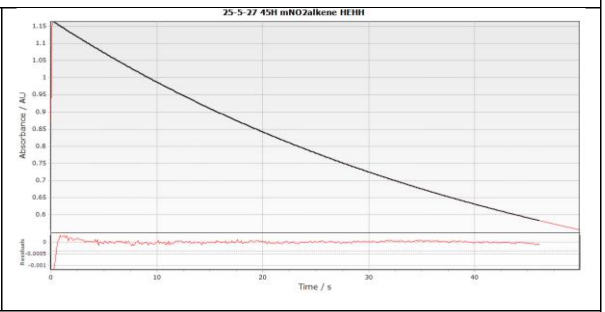
45℃ H	
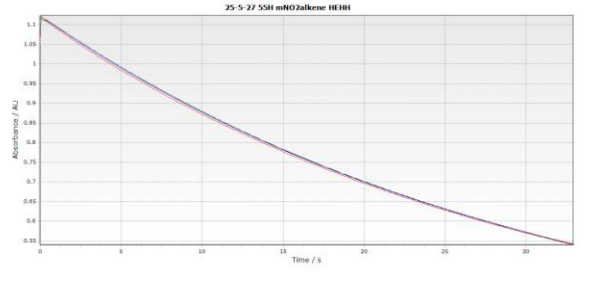	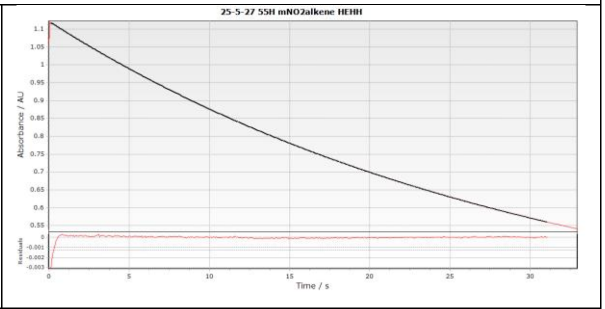
55℃ H	

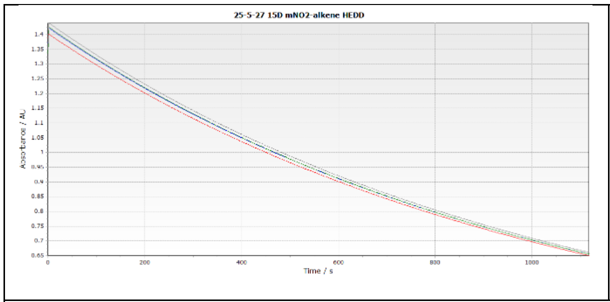

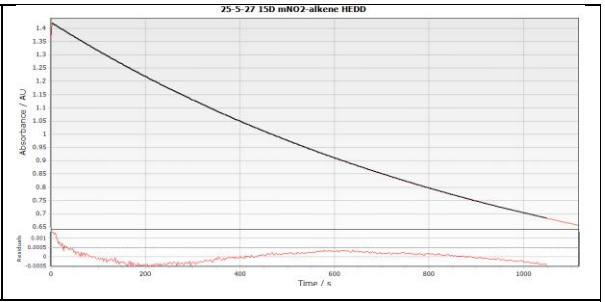

15°C D

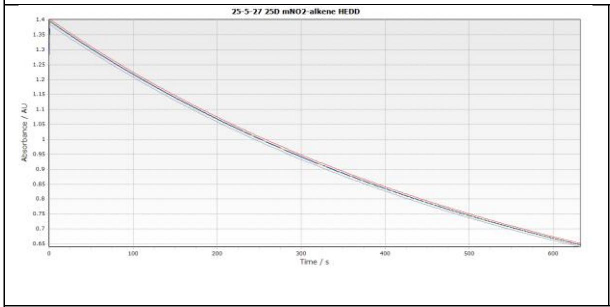

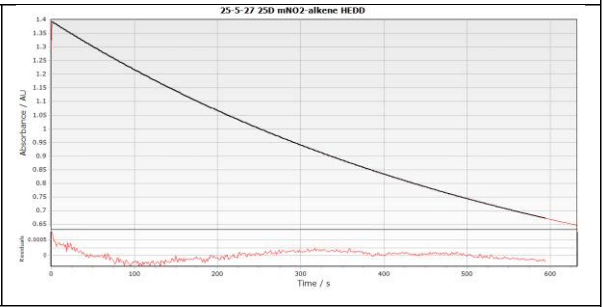

25°C D

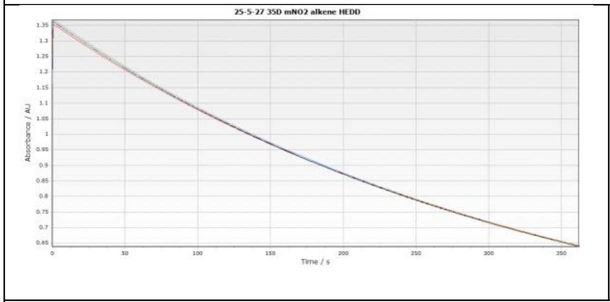

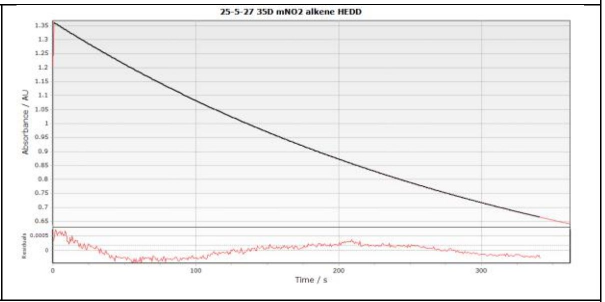

35°C D

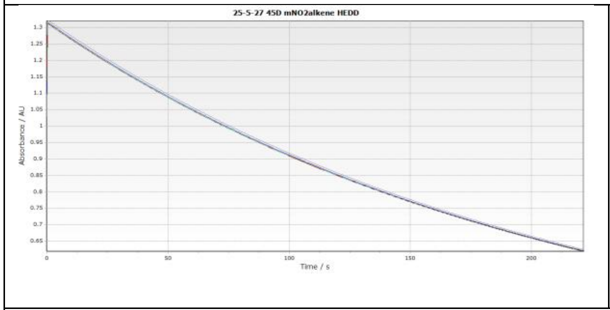

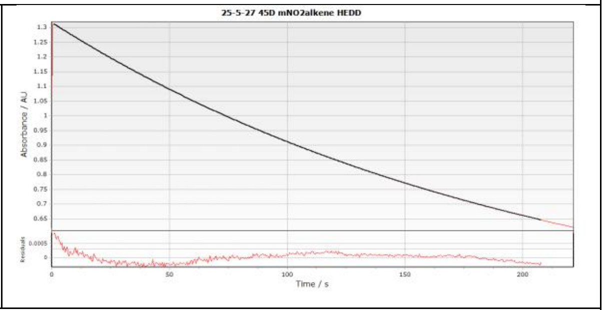

45°C D

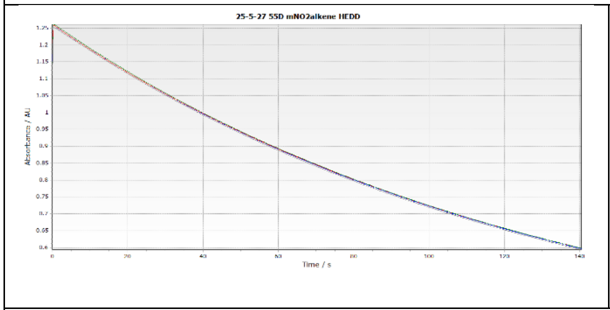

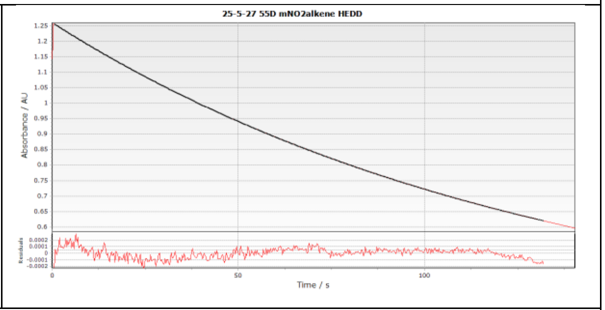

55°C D

Day 3 data (June 29, 2025)

Pseudo-first-order rate constants

k^{pfo} (s^{-1})

Temp (°C)	Trial H1	Trial H2	Trial H3	Average k_H^{pfo} (s^{-1})	Stdev	k_{2H} (M^{-1}s^{-1})	Stdev [a]
55	0.03379	0.03376	0.03389	0.03381	6.81E-05	4.23E+00	8.51E-03
45	0.02252	0.02272	0.02262	0.02262	1.00E-04	2.83E+00	1.25E-02
35	0.01514	0.01515	0.01501	0.01510	7.81E-05	1.89E+00	9.76E-03
25	0.00912	0.00915	0.00907	0.00911	4.04E-05	1.14E+00	5.05E-03
15	0.00555	0.00557	0.00559	0.00557	2.00E-05	6.96E-01	2.50E-03

Temp (°C)	Trial D1	Trial D2	Trial D3	Average k_D^{pfo} (s^{-1})	Stdev	k_{2D} (M^{-1}s^{-1})	Stdev [a]
55	0.00798	0.00798	0.00791	0.00796	4.04E-05	9.95E-01	5.05E-03
45	0.00515	0.00509	0.00502	0.00509	6.51E-05	6.36E-01	8.13E-03
35	0.00309	0.00307	0.00314	0.00310	3.61E-05	3.88E-01	4.51E-03
25	0.00183	0.00184	0.00187	0.00185	2.08E-05	2.31E-01	2.60E-03
15	0.00098	0.00099	0.00099	0.00099	5.77E-06	1.23E-01	7.22E-04

[a] = (Stdev(for k^{pfo})/k^{pfo})*k_2

Three kinetic runs together with the averaged data	First-order kinetic fit of the averaged data as an example
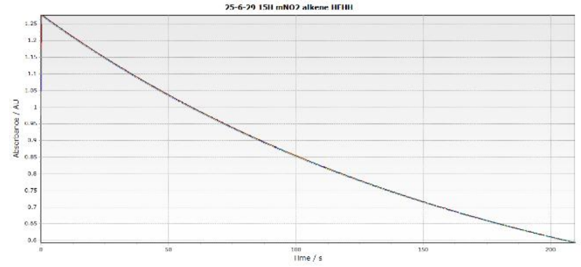	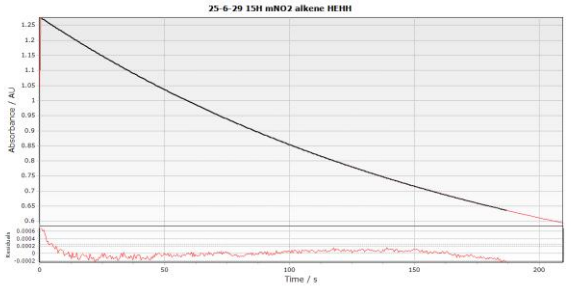
15°C H	
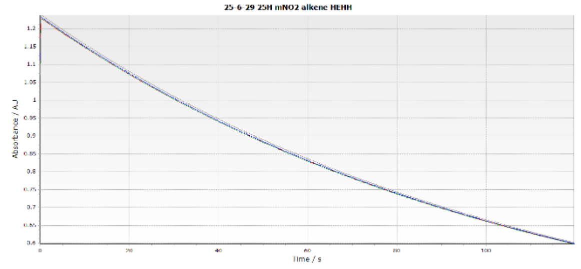	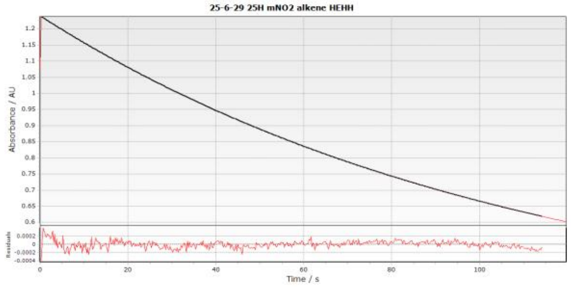
25°C H	

<table>
<tr><td>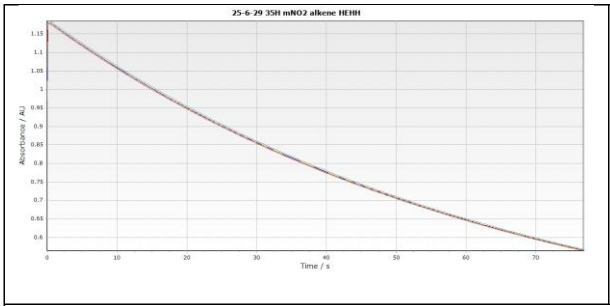
</td><td>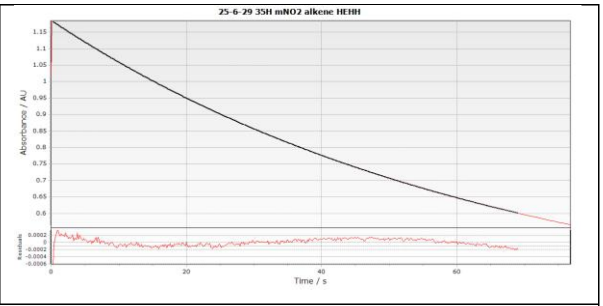
</td></tr>
<tr><td colspan="2">35℃ H</td></tr>
<tr><td>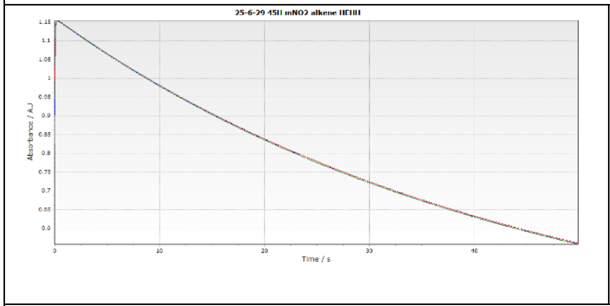
</td><td>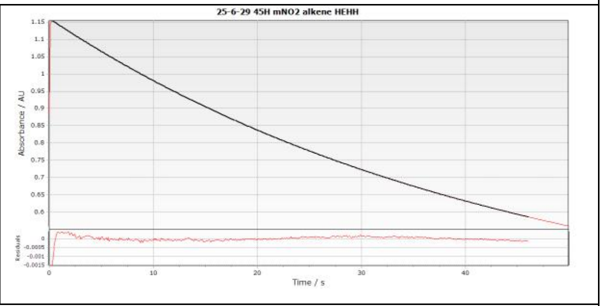
</td></tr>
<tr><td colspan="2">45℃ H</td></tr>
<tr><td>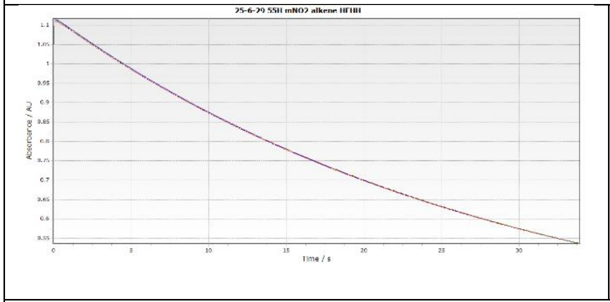
</td><td>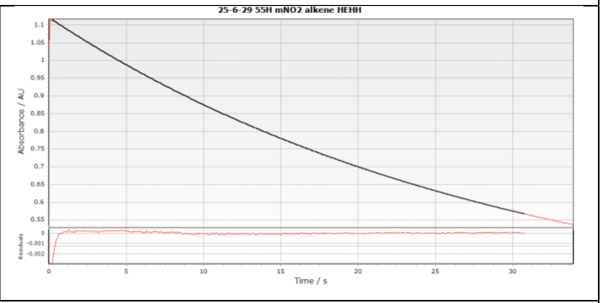
</td></tr>
<tr><td colspan="2">55℃ H</td></tr>
<tr><td>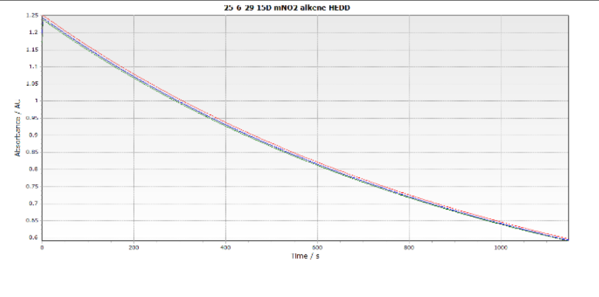
</td><td>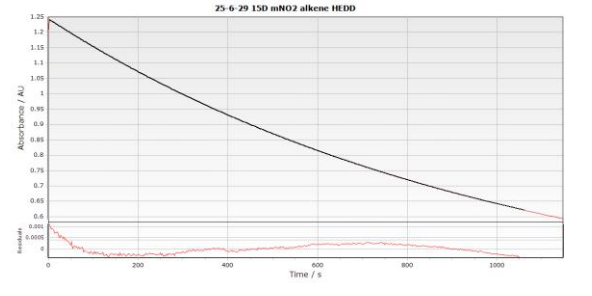
</td></tr>
<tr><td colspan="2">15℃ D</td></tr>
<tr><td>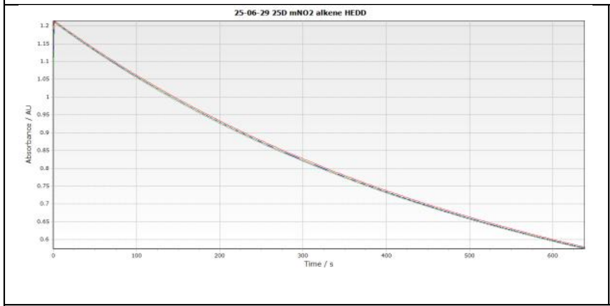
</td><td>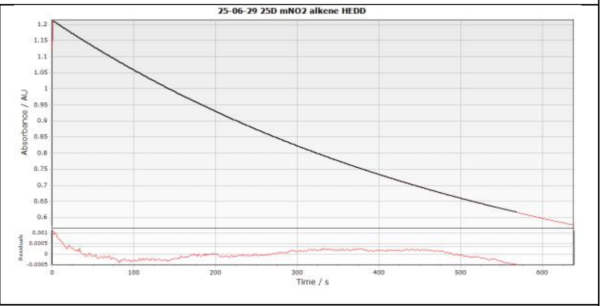
</td></tr>
<tr><td colspan="2">25℃ D</td></tr>
</table>

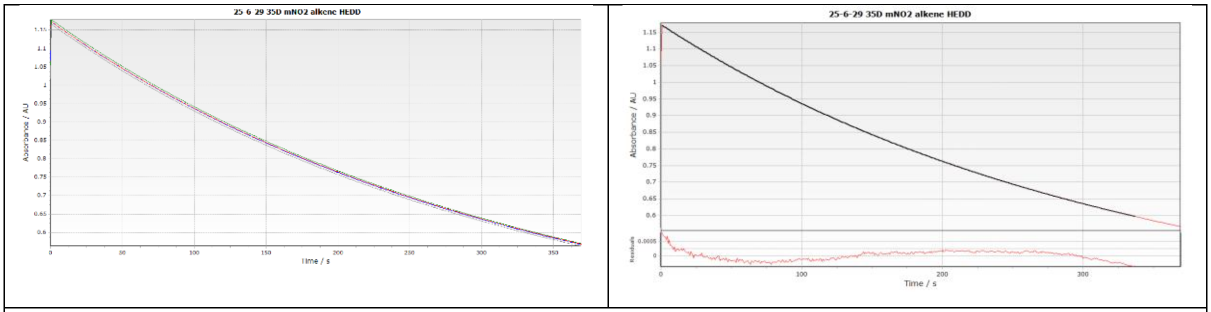

35°C D

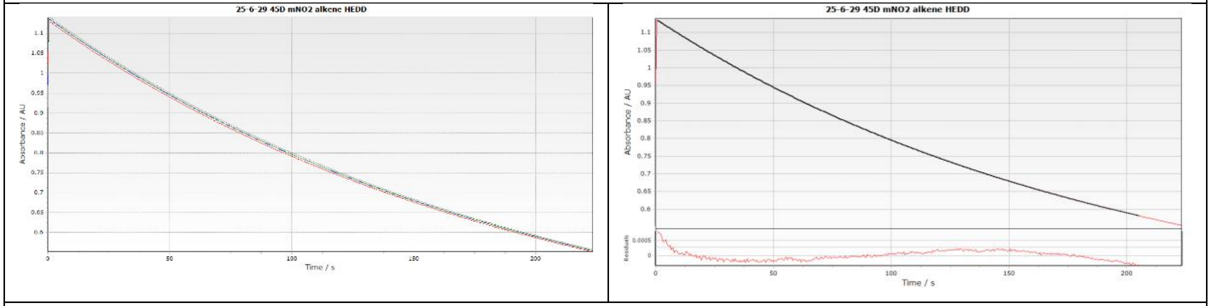

45°C D

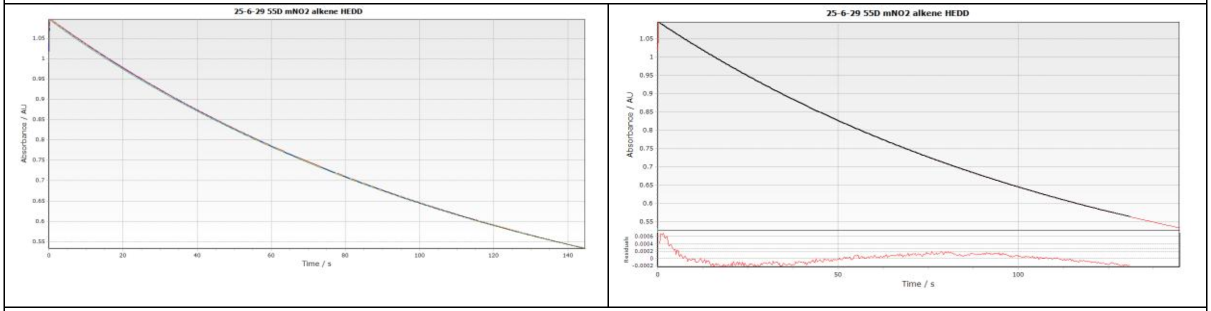

55°C D

Primary kinetic data for the rate constants in Table S10 (HEH with TBMN)

Day 1 data (June 03, 2025)

Pseudo-first-order rate constants

k^{pfo} (s^{-1})

Temp (°C)	Trial H1	Trial H2	Trial H3	Average k_H^{pfo} (s^{-1})	Stdev	k_{2H} (M^{-1}s^{-1})	Stdev [a]
55	0.02752	0.02696	0.02741	0.02730	2.97E-04	2.27E+00	2.47E-02
45	0.01825	0.01840	0.01823	0.01829	9.29E-05	1.52E+00	7.74E-03
35	0.01197	0.01178	0.01180	0.01185	1.04E-04	9.88E-01	8.70E-03
25	0.00750	0.00755	0.00752	0.00752	2.52E-05	6.27E-01	2.10E-03
15	0.00444	0.00441	0.00441	0.00442	1.73E-05	3.68E-01	1.44E-03

Temp (°C)	Trial D1	Trial D2	Trial D3	Average k_D^{pfo} (s^{-1})	Stdev	k_{2D} (M^{-1}s^{-1})	Stdev [a]
55	0.00630	0.00636	0.00643	0.0064	6.51E-05	5.30E-01	5.42E-03
45	0.00405	0.00404	0.00397	0.0040	4.36E-05	3.35E-01	3.63E-03
35	0.00247	0.00245	0.00249	0.0025	2.00E-05	2.06E-01	1.67E-03
25	0.00141	0.00139	0.00141	0.0014	1.15E-05	1.17E-01	9.62E-04
15	0.00081	0.00082	0.00080	0.0008	1.00E-05	6.75E-02	8.33E-04

[a] = (Stdev(for k^{pfo})/k^{pfo})*k_2

Three kinetic runs together with the averaged data	First-order kinetic fit of the averaged data as an example
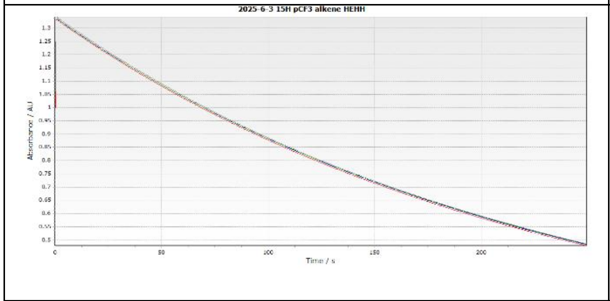	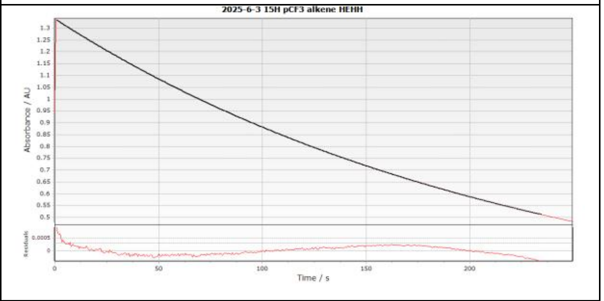
15°C H	
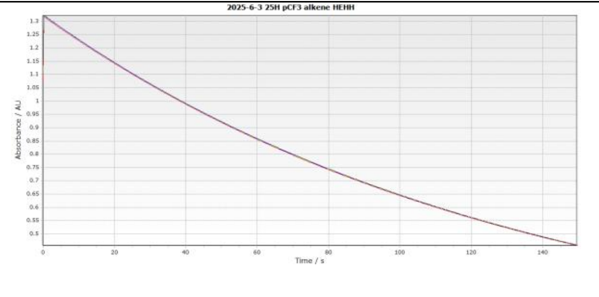	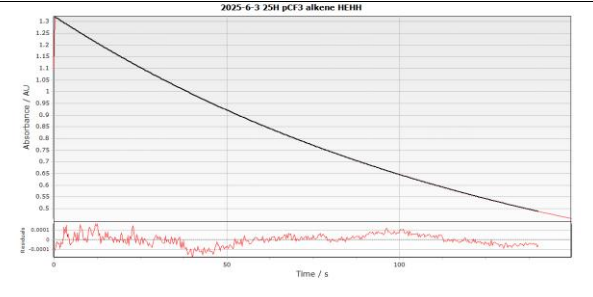
25°C H	
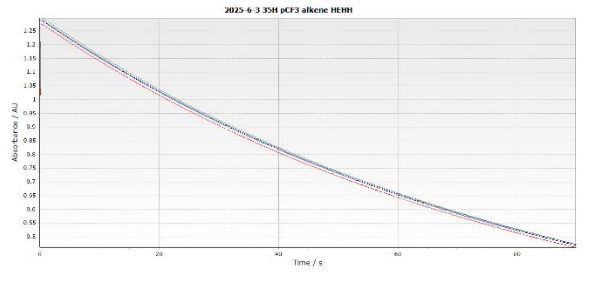	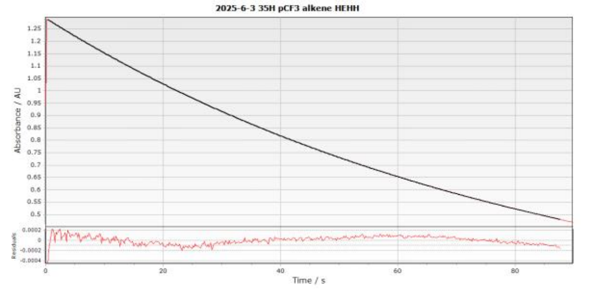
35°C H	
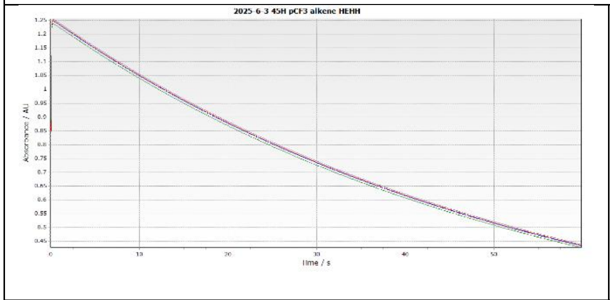	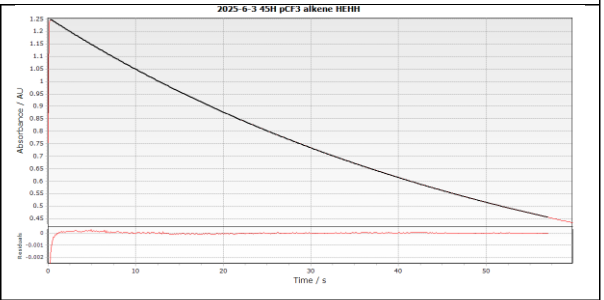
45°C H	

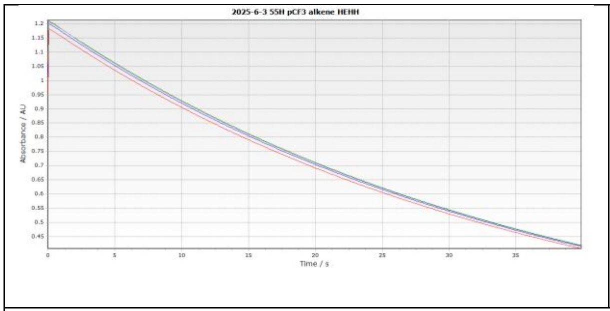

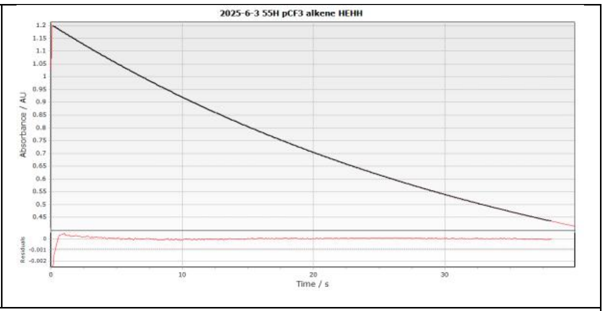

55℃ H

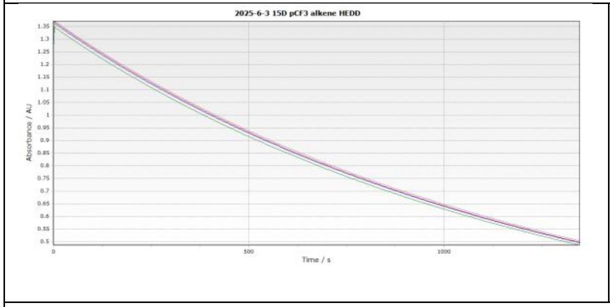

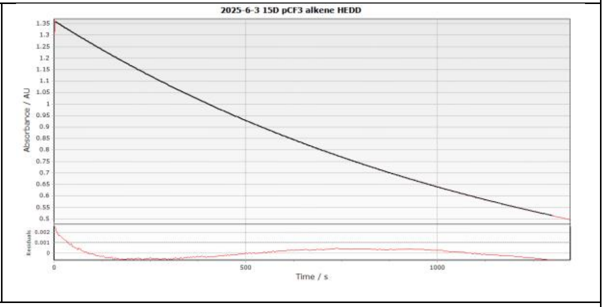

15℃ D

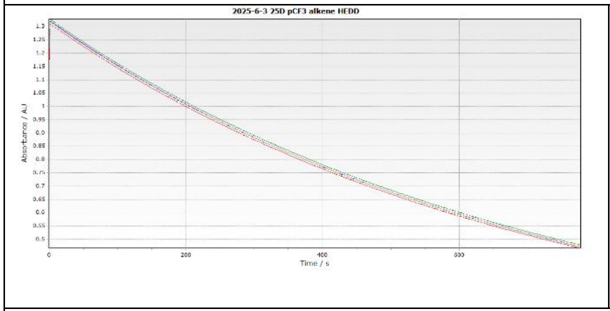

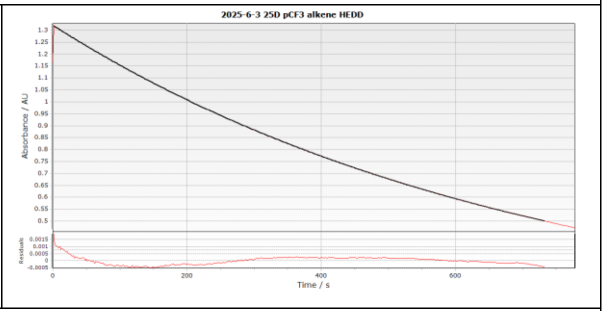

25℃ D

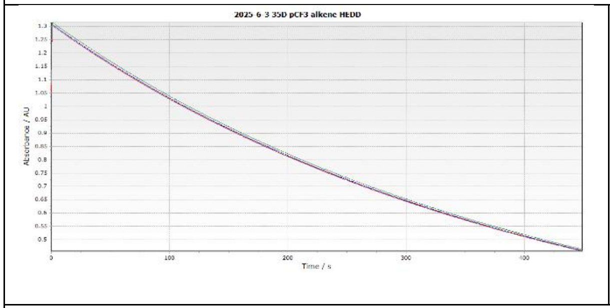

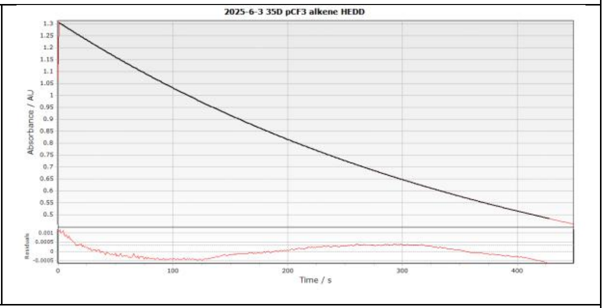

35℃ D

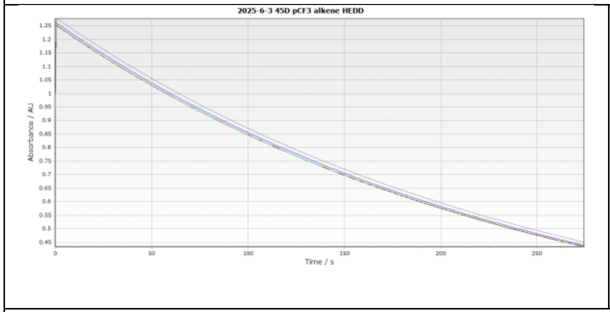

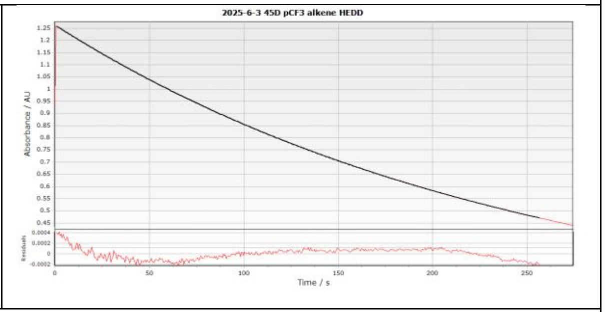

45℃ D

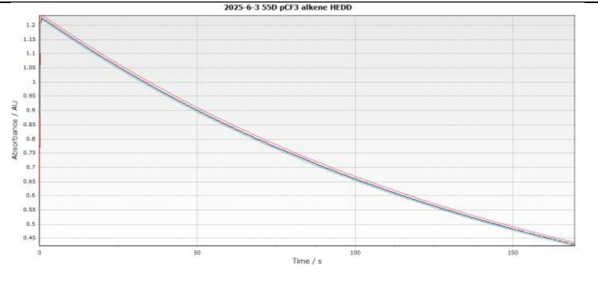

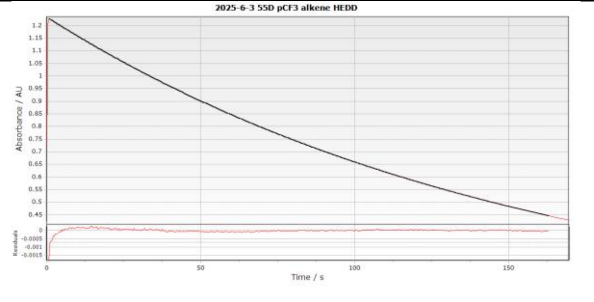

55°C D

Day 2 data (June 10, 2025)

Pseudo-first-order rate constants

k^{pfo} (s^{-1})

**Temp (°C)**	**Trial H1**	**Trial H2**	**Trial H3**	**Average k_H^{pfo} (s^{-1})**	**Stdev**	**k_{2H} (M^{-1}s^{-1})**	**Stdev [a]**
55	0.02588	0.02618	0.02672	0.02626	4.26E-04	2.19E+00	3.55E-02
45	0.01718	0.01742	0.01742	0.01734	1.39E-04	1.45E+00	1.15E-02
35	0.01137	0.01133	0.01115	0.01128	1.17E-04	9.40E-01	9.77E-03
25	0.00700	0.00694	0.00700	0.00698	3.46E-05	5.82E-01	2.89E-03
15	0.00422	0.00423	0.00417	0.00421	3.21E-05	3.51E-01	2.68E-03

**Temp (°C)**	**Trial D1**	**Trial D2**	**Trial D3**	**Average k_D^{pfo} (s^{-1})**	**Stdev**	**k_{2D} (M^{-1}s^{-1})**	**Stdev [a]**
55	0.00606	0.00604	0.00598	0.0060	4.16E-05	5.02E-01	3.47E-03
45	0.00382	0.00382	0.00382	0.0038	0.00E+00	3.18E-01	0.00E+00
35	0.00233	0.00235	0.00233	0.0023	1.15E-05	1.95E-01	9.62E-04
25	0.00139	0.00141	0.00139	0.0014	1.15E-05	1.16E-01	9.62E-04
15	0.00074	0.00074	0.00075	0.0007	5.77E-06	6.19E-02	4.81E-04

[a] = (Stdev(for k^{pfo})/k^{pfo})*k_2

Three kinetic runs together with the averaged data

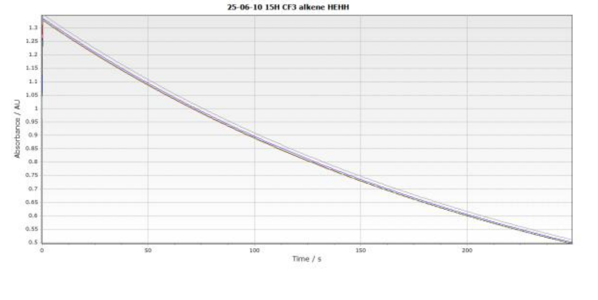

First-order kinetic fit of the averaged data as an example

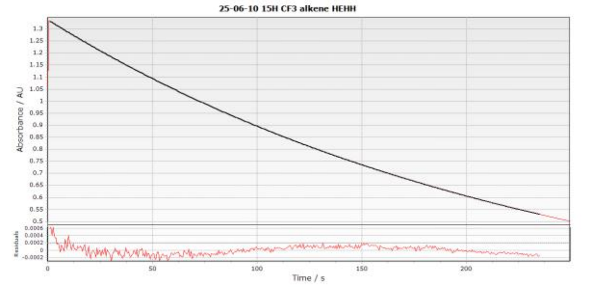

15°C H

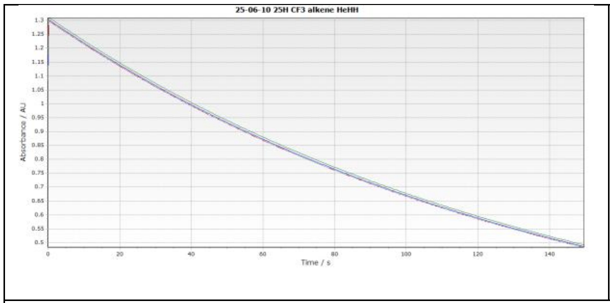

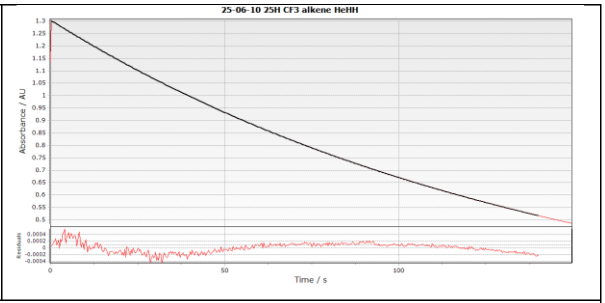

25°C H

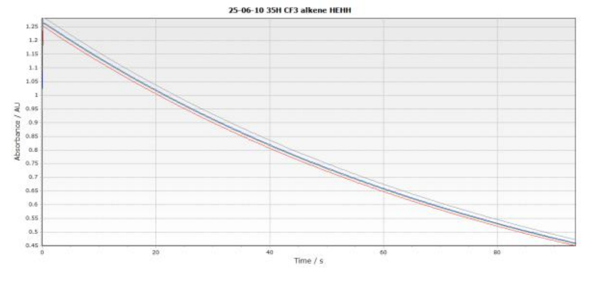

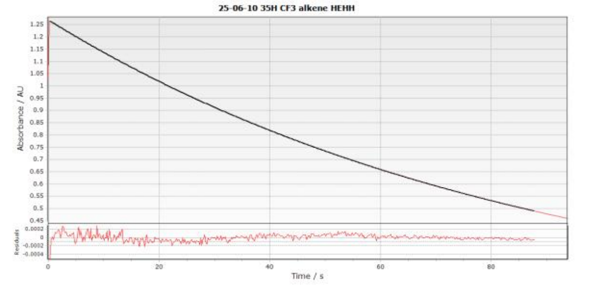

35°C H

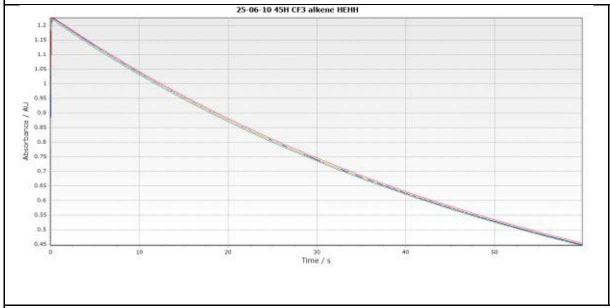

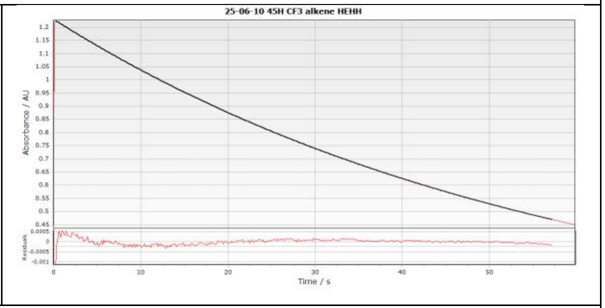

45°C H

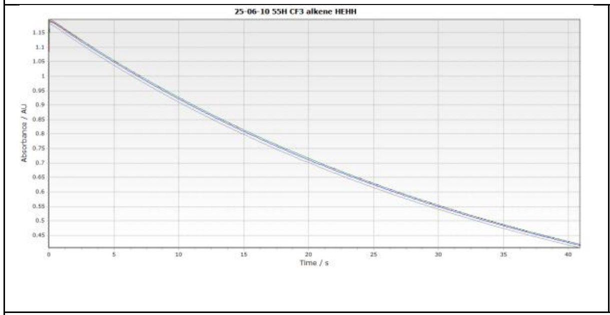

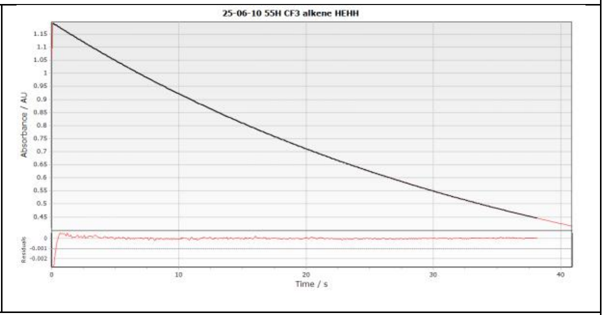

55°C H

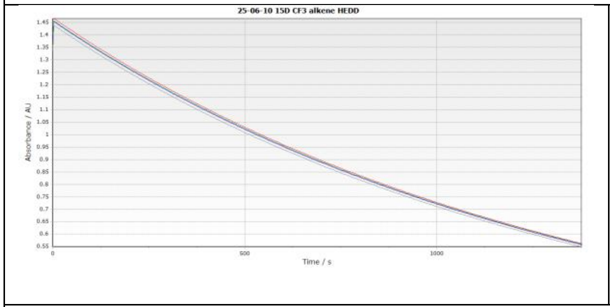

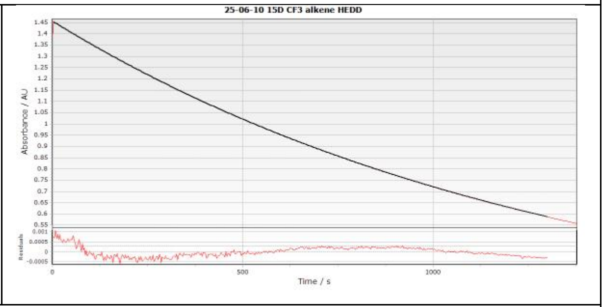

15°C D

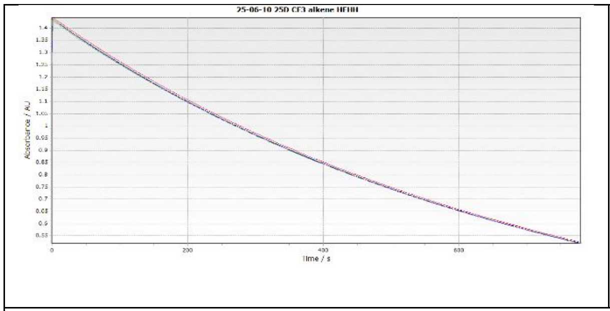

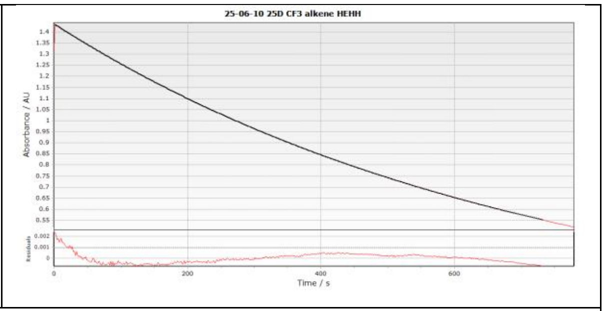

25°C D

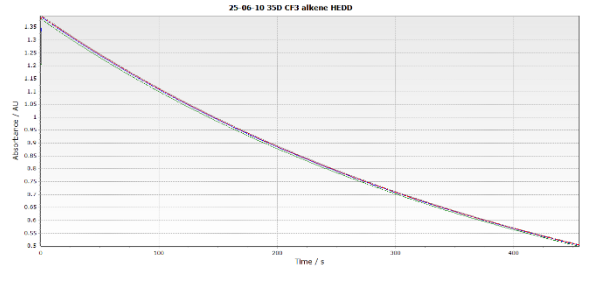

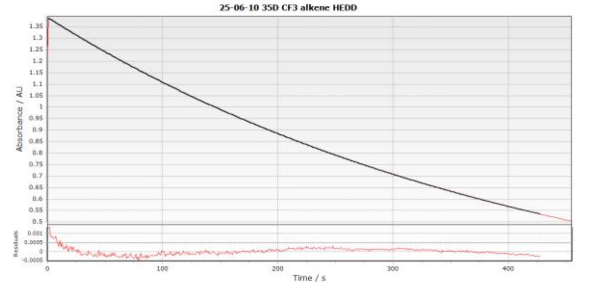

35°C D

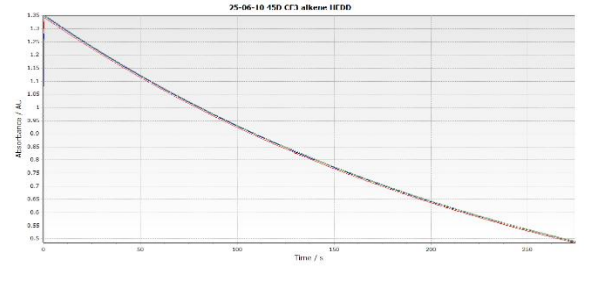

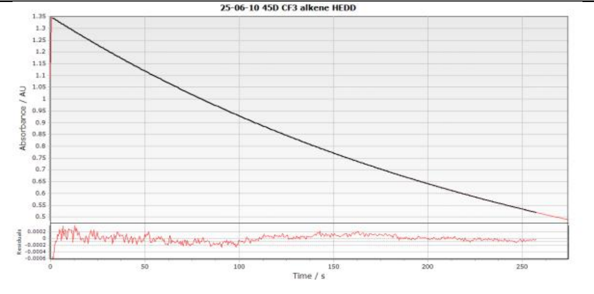

45°C D

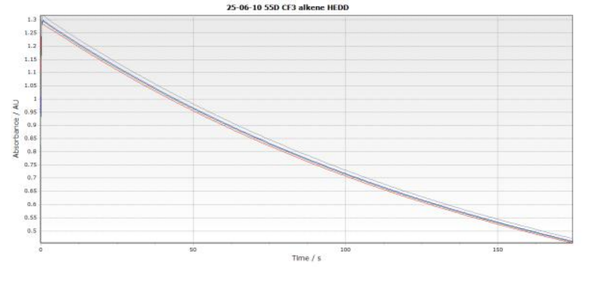

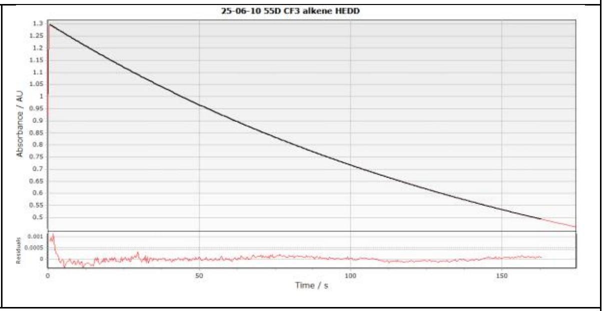

55°C D

Day 3 data (June 22, 2025)

Pseudo-first-order rate constants

k^{pfo} (s^{-1})

**Temp (°C)**	**Trial H1**	**Trial H2**	**Trial H3**	**Average k_H^{pfo} (s^{-1})**	**Stdev**	**k_{2H} ($M^{-1}s^{-1}$)**	**Stdev [a]**
55	0.02687	0.02683	0.02673	0.02681	7.21E-05	2.23E+00	6.01E-03
45	0.01794	0.01768	0.01777	0.01780	1.32E-04	1.48E+00	1.10E-02
35	0.01153	0.01177	0.01154	0.01161	1.36E-04	9.68E-01	1.13E-02
25	0.00732	0.00737	0.00732	0.00734	2.89E-05	6.11E-01	2.41E-03
15	0.00415	0.00413	0.00415	0.00414	1.15E-05	3.45E-01	9.62E-04

**Temp (°C)**	**Trial D1**	**Trial D2**	**Trial D3**	**Average k_D^{pfo} (s^{-1})**	**Stdev**	**k_{2D} ($M^{-1}s^{-1}$)**	**Stdev [a]**
55	0.00622	0.00627	0.00624	0.0062	2.52E-05	5.20E-01	2.10E-03
45	0.00394	0.00390	0.00395	0.0039	2.65E-05	3.28E-01	2.20E-03
35	0.00240	0.00242	0.00239	0.0024	1.53E-05	2.00E-01	1.27E-03
25	0.00134	0.00133	0.00137	0.0013	2.08E-05	1.12E-01	1.73E-03
15	0.00077	0.00076	0.00078	0.0008	1.00E-05	6.42E-02	8.33E-04

[a] = (Stdev(for k^{pfo})/k^{pfo})*k_2

Three kinetic runs together with the averaged data	First-order kinetic fit of the averaged data as an example
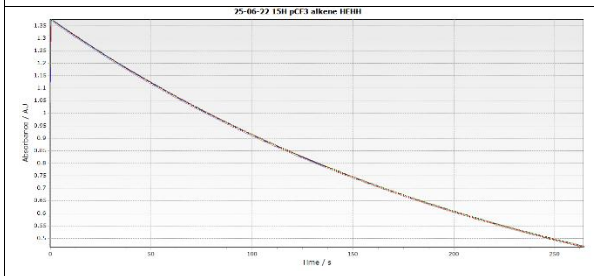	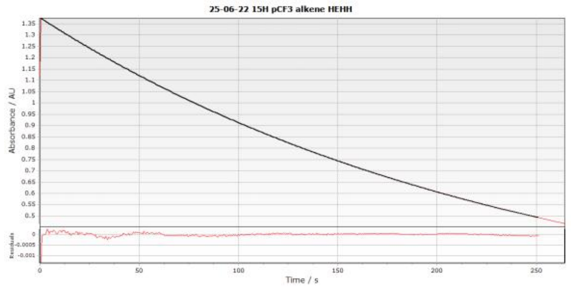
15°C H	
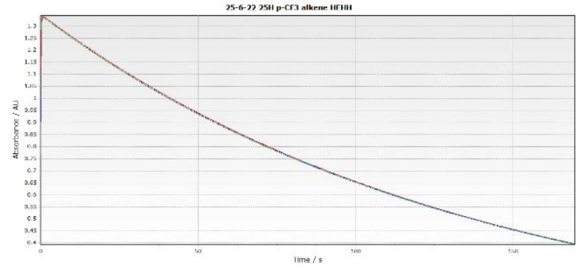	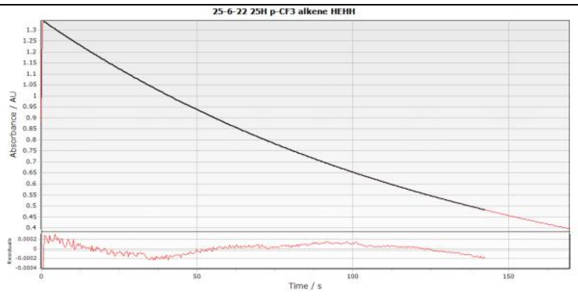
25°C H	

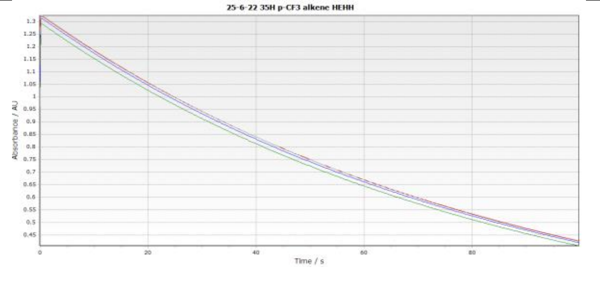

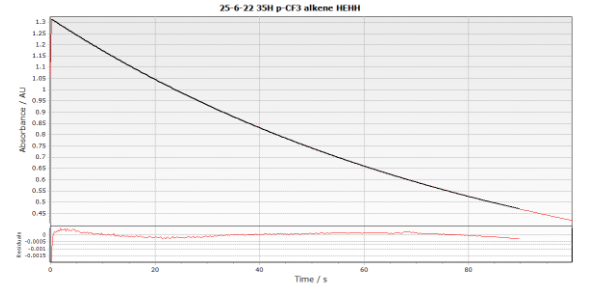

35°C H

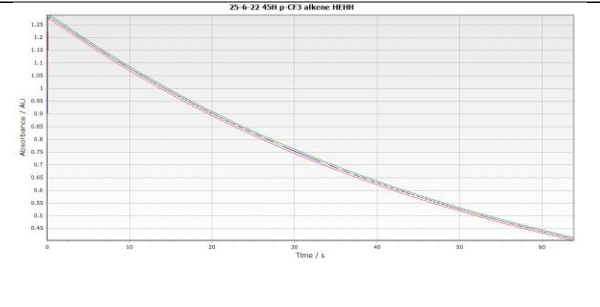

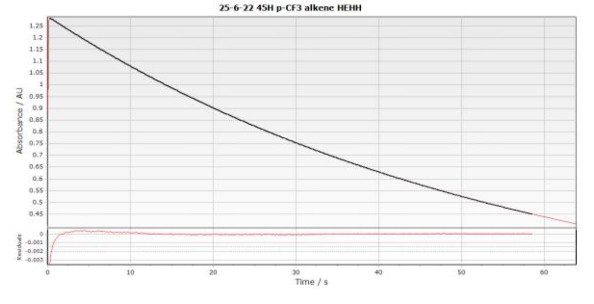

45°C H

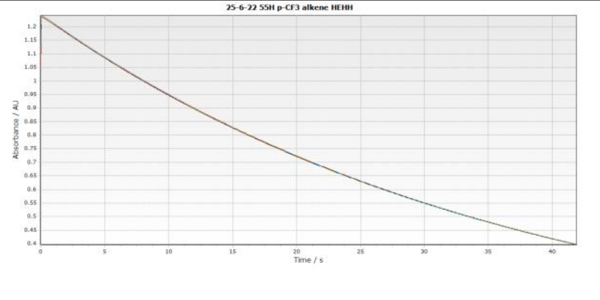

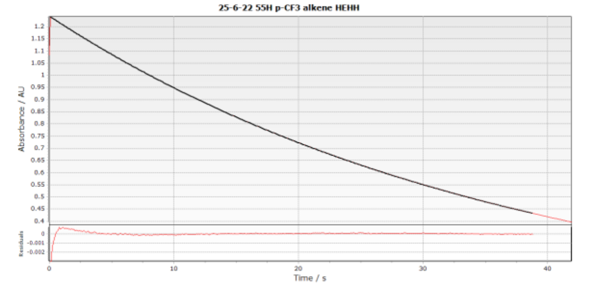

55°C H

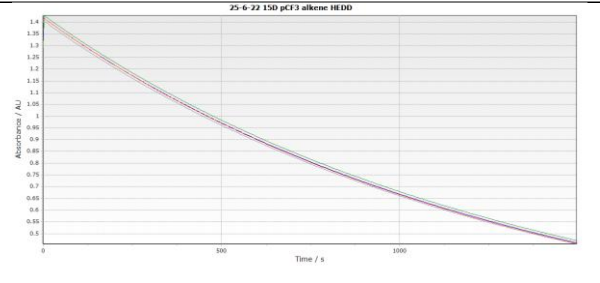

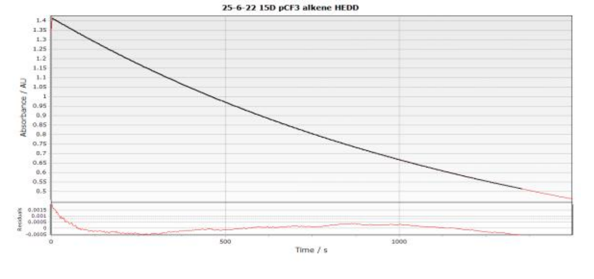

15°C D

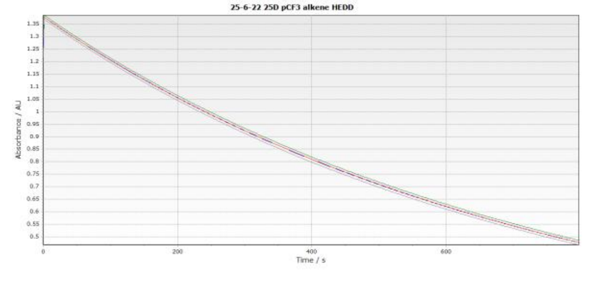

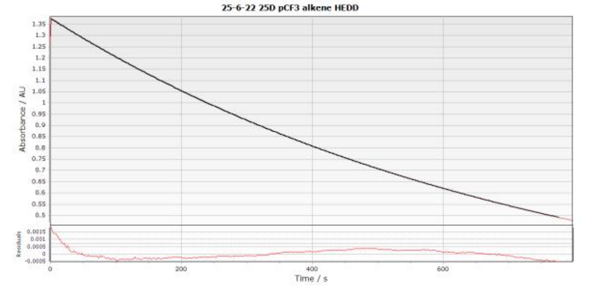

25°C D

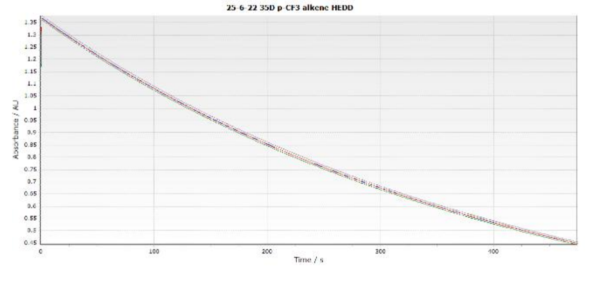

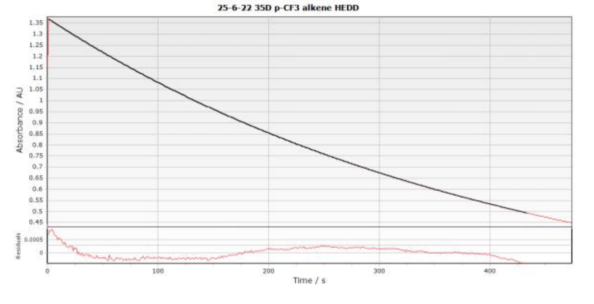

35°C D

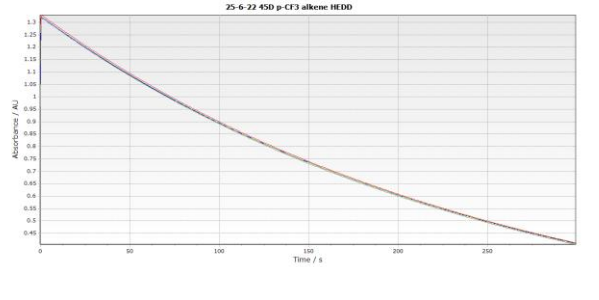

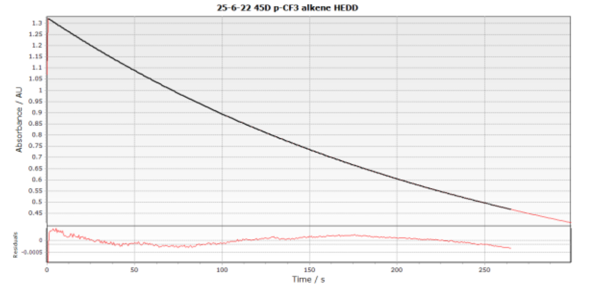

45°C D

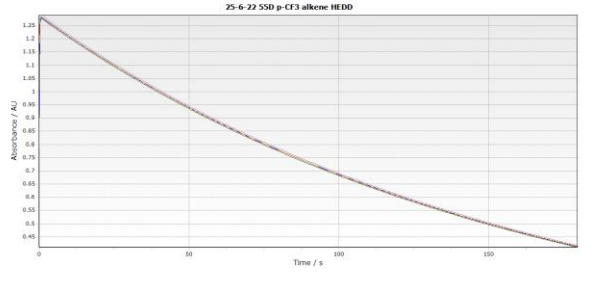

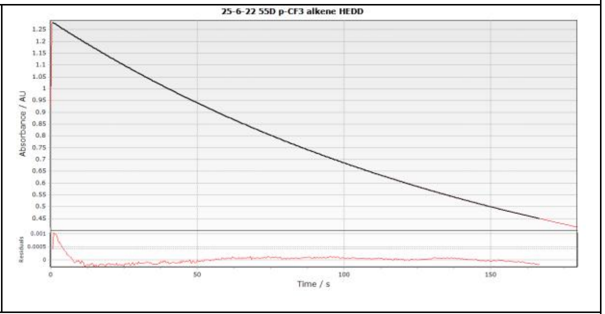

55°C D
